# 8-Hydroxyquinoline Glycoconjugates Containing Sulfur at the Sugar Anomeric Position—Synthesis and Preliminary Evaluation of Their Cytotoxicity

**DOI:** 10.3390/molecules25184174

**Published:** 2020-09-11

**Authors:** Monika Krawczyk, Gabriela Pastuch-Gawołek, Agnieszka Hadasik, Karol Erfurt

**Affiliations:** 1 Department of Organic Chemistry, Bioorganic Chemistry and Biotechnology, Silesian University of Technology, B. Krzywoustego 4, 44-100 Gliwice, Poland; gabriela.pastuch@polsl.pl (G.P.-G.); agnieszkahadasik@gmail.com (A.H.); 2 Biotechnology Centre, Silesian University of Technology, B. Krzywoustego 8, 44-100 Gliwice, Poland; 3 Department of Chemical Organic Technology and Petrochemistry, Silesian University of Technology, B. Krzywoustego 4, 44-100 Gliwice, Poland; karol.erfurt@polsl.pl

**Keywords:** quinoline, glycoconjugates, 1-thiosugars, click reaction, anticancer activity, targeted drug delivery

## Abstract

One of the main factors limiting the effectiveness of many drugs is the difficulty of their delivery to their target site in the cell and achieving the desired therapeutic dose. Moreover, the accumulation of the drug in healthy tissue can lead to serious side effects. The way to improve the selectivity of a drug to the cancer cells seems to be its conjugation with a sugar molecule, which should facilitate its selective transport through GLUT transporters (glucose transporters), whose overexpression is seen in some types of cancer. This was the idea behind the synthesis of 8-hydroxyquinoline (8-HQ) derivative glycoconjugates, for which 1-thiosugar derivatives were used as sugar moiety donors. It was expected that the introduction of a sulfur atom instead of an oxygen atom into the anomeric position of the sugar would increase the stability of the obtained glycoconjugates against untimely hydrolytic cleavage. The anticancer activity of new compounds was determined based on the results of the MTT cytotoxicity tests. Because of the assumption that the activity of this type of compounds was based on metal ion chelation, the effect of the addition of copper ions on cell proliferation was tested for some of them. It turned out that cancer cells treated with glycoconjugates in the presence of Cu^2+^ had a much slower growth rate compared to cells treated with free glycoconjugates in the absence of copper. The highest cytotoxic activity of the compounds was observed against the MCF-7 cell line.

## 1. Introduction

Despite significant progress in the treatment of various types of diseases, cancer is still one of the most considerable clinical problems. Anticancer therapies introduced so far are not effective enough. This is due to the low selectivity profile of approved chemotherapeutics, the use of which leads to numerous undesirable side effects. Moreover, the resistance of cancer cells to available pharmaceuticals results in their rapid proliferation and metastasis [1]. Therefore, it is necessary to develop new safe drugs that target cancer cells directly without damaging healthy cells.

One of the main factors limiting the effectiveness of many drugs is the difficulty of their delivery to their target site in the cell across the phospholipid cell membranes. A better understanding of the processes ruling the delivery of substances to cancer cells can be helpful during planning new treatment strategies. There are several ways to transport into the cell [2,3]. The vast majority of available chemotherapeutics penetrate cell membranes by passive diffusion. This is due to the presence of specific gaps in the blood vessels of cancer cells resulting from chaotic neoangiogenesis. This is commonly known as the effect of enhanced permeability and retention (EPR) [3,4,5]. On the other hand, a variety of membrane proteins are anchored in the structure of lipid bilayer, which can act as carrier proteins or channel proteins (facilitated diffusion). An attractive alternative to transporting chemotherapeutics across a biological membrane may be their modification, which will increase affinity for binding to the appropriate receptor [2,3,4,5].

One of the strategies developed for delivering anticancer drugs is the use of glucose transporting proteins [6,7]. Compared to healthy cells, rapidly proliferating cancer cells have a different energy metabolism, characterized by a high rate of glycolysis process, known as the Warburg effect [8,9]. To ensure a sufficient amount of nutrients up to this process, cancer cells have an increased demand for glucose. The phospholipid membrane surrounding the cells is impermeable to hydrophilic glucose molecules by simple diffusion. Therefore, sugar to the cytosol is transferred by transmembrane proteins-GLUTs or SGLTs (sodium-glucose linked transporters) [10]. In most tumor cells, these transporters are overexpressed, especially GLUT1 [11]. Thanks to these transporters, it has been proven that they even receive over 30 times more glucose than normal cells [12]. GLUT1 transport substrates also include other sugars, such as galactose, mannose, and glucosamine [11]. Therefore, an attractive system for targeted drug delivery directly to cancer cells is their conjugation with sugar derivatives. The sugar fragment can be used as a drug carrier across the cell membrane by adapting to the structure of the GLUT transporter [6]. The crucial question to be answered in the case of such conjugates is whether the sugar-conjugated compound will be transported to the cancer cells by the sugar transporters. The results of research on structural requirements for binding variously substituted sugars to the sugar transporters indicate that β-configuration at the anomeric center of the sugar derivatives is preferred for binding to the GLUT transporters [13,14,15].

**Figure 1:**
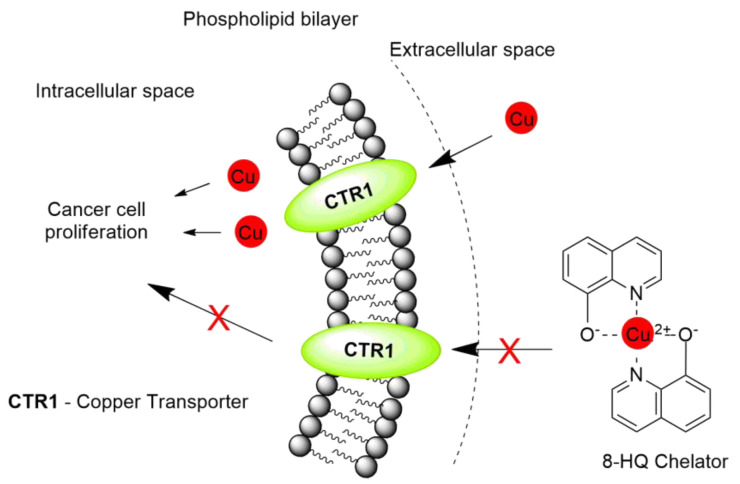
The concept of using metal chelators.

Our research focuses on the use of small quinoline molecules as metal ion chelators and potential anticancer agents [16,17]. The presence of copper ions is important in many biological processes and is essential for cellular physiology [18]. Copper is a co-factor of many enzymes and takes part in a variety of cellular processes in mammals [18]. Changes in the homeostasis of copper ions play an important role in different types of diseases, such as inflammation, neurodegeneration, as well as in carcinogenesis (copper is an essential cofactor for cancer growth and angiogenesis) [18,19,20,21]. Therefore, the use of metal chelating agents seems to be an ideal way to control the level of copper in the organism [21,22,23]. The concept of using metal chelators is shown in Figure 1. The formation of metal complexes by 8-hydroxyquinoline (8-HQ) derivatives has been widely described in the literature and has become a promising therapeutic strategy in clinical practice. Numerous 8-HQ derivatives have been shown to have cytotoxic activity on many cancer cells [16,24,25]. However, a significant limitation of their use is the lack of selectivity for healthy cells. We assumed that the therapeutic effectiveness of 8-HQ derivatives could be improved by attaching a sugar moiety, which should increase the selectivity of the obtained connections by targeting their transport to cancer cells through GLUT transporters. 8-Hydroxyquinoline glycoconjugates are of particular interest due to their simple synthesis procedures, as well as facilitated intermembrane transport and improved solubility.

Our recent studies on the cytotoxic activity of 8-HQ derivatives connected *via* various linkers with d-glucose or d-galactose derivatives containing an oxygen or nitrogen atom at the sugar anomeric position indicated that some of them were able to inhibit the proliferation of tested cell lines [26,27]. Their biological activity depended on the structure of the linker connecting the sugar parts and the quinoline moiety, as well as the type of protective groups in the attached sugar unit. The study of metal complexing properties confirmed that the obtained glycoconjugates were capable of chelating copper ions [27]. In the current research, 1-thiosugars derivatives were used for the synthesis of quinoline glycoconjugates. It was expected that the introduction of a sulfur atom into the anomeric position of the sugar would increase the stability of the obtained glycoconjugates against hydrolytic cleavage. Compared to *O*-glycosyl derivatives, compounds with the *S*-glycosidic binding are less susceptible to enzymatic degradation, especially under the action of glycosylhydrolases [28,29]. 1-Thiosugars, due to their enzymatic stability, exhibit great therapeutic potential [30,31,32]. The thiosugar moiety can be found in many naturally occurring compounds, such as bacteriostatic antibiotics from the group of lincosamides (lincomycin) or d-glucosinolates (sinigrin, gluconasturcin), which have chemopreventive properties [30]. Synthetic thiosugars are effective inhibitors of numerous cellular enzyme pathways. Besides, numerous studies have shown their significant antimicrobial, antiviral, antithrombotic, and anticancer potential [33,34,35,36]. Therefore, increasingly sulfur derivatives of carbohydrates are used for the synthesis of new pharmaceuticals. An example of a 1-thiosugar glycoconjugate that exhibits antiproliferative effects *in vivo* is a gold-based compound 1-phenyl-bis(2-pyridyl)phosphole gold chloride thio-β-d-glucose tetraacetate (GOPI-sugar). It inhibits the proliferation of glioblastoma cell lines (NCH82, NCH89) to a much better extent than the drug used so far-carmustine, making it a promising candidate in cancer therapy. Attachment of a sugar unit to the complex GOPI has improved the solubility and bioavailability of this compound [37]. Previously reported quinolinecarboxylic acid glycoconjugates containing in their structure a fragment derived from 1-thioglycosides have shown a particularly interesting activity profile against colorectal cancer cells (HCT-116) more than 100 times exceeding the activity of the initial quinoline derivatives [38]. Considering the number of reports devoted to the use of thiosugars in the treatment of a wide range of human diseases, it is worth using this potential. In addition, compounds containing a sulfur atom have the ability to complex metal ions, with sulfur preferring the so-called “soft” metals, such as gold, silver, or copper [39,40]. The introduction of sulfur to the designed glycoconjugates may improve their ability to complex copper ions in relation to the previously obtained derivatives. In this article, we presented the results of carried out cytotoxicity tests of new 8-HQ derivatives, allowing us to determine their potential usefulness in anticancer therapy.

## 2. Results and Discussion

### 2.1. Synthesis

**Scheme 1:**
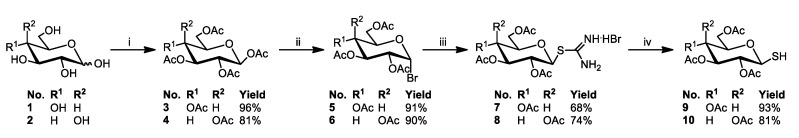
Synthesis of sugar derivatives **3**–**10**. Reagents and Conditions: (**i**) CH_3_COONa, Ac_2_O, b.p., 1 h; (**ii**) CH_3_COOH, 33% HBr/AcOH, 0 °C-r.t., 1 h; (**iii**) thiourea, acetone, b.p., 3 h; (**iv**) K_2_S_2_O_5_, CHCl_3_, H_2_O, b.p., 1 h.

**Scheme 2:**
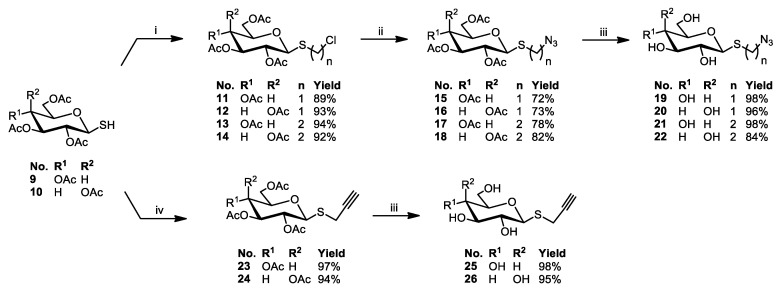
Synthesis of 1-thiosugar derivatives **11**–**26**. Reagents and Conditions: (**i**) DBU, CH_2_Cl_2_ or (CH_2_)_2_Cl_2_, r.t., 1 h; (**ii**) NaN_3_, DMF, r.t., 24 h (**15** and **16**) or 60 °C, 72 h (**17** and **18**); (**iii**) 1. MeONa, MeOH, r.t., 50 min; 2. Amberlyst-15; (**iv**) propargyl bromide, K_2_CO_3_, acetone, r.t., 24 h.

**Scheme 3:**
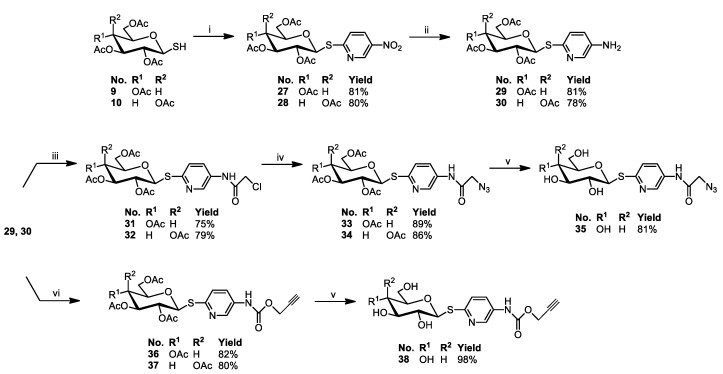
Synthesis of 1-thiosugar derivatives **27**–**38**. Reagents and Conditions: (**i**) 2-chloro-5-nitropyridine, K_2_CO_3_, acetone, r.t., 2 h; (**ii**) Zn, AcOH, CH_2_Cl_2_, r.t., 1 h; (**iii**) chloroacetyl chloride, TEA, DCM, r.t., 1 h; (**iv**) NaN_3_, DMF, r.t., 24 h; (**v**) 1. MeONa, MeOH, r.t., 1 h; 2. Amberlyst-15; (**vi**) propargyl chloroformate, Hünig′s base, DCM, r.t., 2 h.

The structural modification of sugars used in this research concerned the introduction of a sulfur atom into the anomeric position of the sugar, followed by its functionalization by chemical groups involved in the copper(I)-catalyzed alkyne-azide cycloaddition (CuAAC). The synthetic pathways leading to the formation of sugar derivatives are presented in Scheme 1, Scheme 2 and Scheme 3. All substrates were prepared according to the previously published procedures thoroughly or making minor improvements.

2,3,4,6-Tetra-*O*-acetyl-1-thio-β-d-glycopyranoses **9** and **10** were synthesized by carrying out a sequence of reactions in which first glycosyl bromides **5** or **6** reacted with thiourea in acetone, and then such prepared isothiouronium salts **7** and **8** were decomposed in the presence of potassium metabisulfite in a two-phase water/chloroform system [41] (Scheme 1).

1-Thiosugars **9** or **10** were substrates for the preparation of compounds used in the synthesis of final glycoconjugates. Due to the susceptibility of these compounds to oxidation to symmetrical disulfides, they were used immediately after purification by column chromatography for the next stages of synthesis. For the glycoconjugates synthesis, the CuAAC reaction was used, in which the substrates must possess an azide moiety or triple bond. Accordingly, it was necessary to receive different 1-thiosugar derivatives substituted at the anomeric position with a substituent containing either the azide moiety or triple bond.

In the beginning, chloromethyl 2,3,4,6-tetra-*O*-acetyl-1-thio-β-d-glycopyranosides **11** or **12** were obtained in the S_N_2 reaction between corresponding glycosyl thiols **9** or **10** and dichloromethane, in the presence of a strong base, such as DBU (1,8-diazabicyclo[5.4.0]undec-7-ene) [42]. To lengthen the alkyl chain in the structure of chloroalkyl 1-thioglycosides, an analogous reaction of 1-thiosugars **9** or **10** with 1,2-dichloroethane was carried out to obtain 2-chloroethyl 2,3,4,6-tetra-*O*-acetyl-1-thio-β-d-glycopyranosides **13** or **14**. In these reactions, both dichloromethane and dichloroethane acted as a reactant and solvent at the same time. As a result, products **11**–**14** were obtained in a short time and with high yield. In the next step, the substitution of a chlorine atom with an azide moiety was carried out using sodium azide and DMF (*N*,*N*-dimethylformamide) as a solvent, which resulted in azidomethyl **15**, **16** and 2-azidoethyl 2,3,4,6-tetra-*O*-acetyl-1-thio-β-d-glycopyranosides **17**, **18**. Confirmation of chlorine exchange to the azide moiety was the appearance in the ^13^C NMR spectra of the signal of CH_2_N_3_ carbon with a shift of δ = 51.03–51.72 ppm for products **15**–**18**, while the signal of CH_2_Cl carbon was observed at δ = 43.31–45.45 ppm for substrates **11**–**14**.

Propargyl 2,3,4,6-tetra-*O*-acetyl-1-thio-β-d-glycopyranosides **23** and **24** were prepared by the alkylation of 1-thiosugars **9** or **10** with propargyl bromide. Minor modifications of the reaction conditions, compared to the previously reported synthesis method [43], consisting of replacing diisopropylethylamine (base) and dichloromethane (originally used as a solvent) with potassium carbonate and acetone, allowed obtaining the appropriate propargyl 1-thioglycosides **23** and **24** with high yields (97 and 94%, respectively, in comparison to 76 and 83% in cited position).

Removal of the acetyl protecting groups from compounds **15**–**18** and **23**–**24** was performed using a standard Zemplén procedure, involving the use of catalytic amounts of a methanolic solution of sodium methoxide [44]. The reactions were carried out until the complete conversion of the substrate, which was monitored by TLC (thin-layer chromatography). Crude products **19**–**22** and **25**–**26** were obtained with high yields (84–98%), sufficiently pure to be used in the next step.

Another direction of synthesis concerned the introduction of an additional aminopyridyl fragment in the sugar part of the final structure. A simple and efficient synthesis of (5-nitro-2-pyridyl) 2,3,4,6-tetra-*O*-acetyl-1-thio-β-d-glycopyranosides **27** and **28**, followed by reduction to (5-amino-2-pyridyl) 2,3,4,6-tetra-*O*-acetyl-1-thio-β-d-glycopyranoside **29** and **30,** was described a few years ago [45]. The presence of an amino group in the aglycone of 1-thioglycosides **29** and **30** allowed the addition of a fragment containing an azide or propargyl moiety. This was possible as a result of the reaction leading to the formation of an amide or carbamate bond. It is worth emphasizing that the introduction of an amide bond additionally enhanced linker stability. Compounds **29** or **30** were reacted with chloroacetyl chloride or propargyl chloroformate in anhydrous DCM (dichloromethane) (Scheme 3). The hydrogen chloride produced during these reactions was neutralized by the addition to the reaction mixture of a tertiary amine, such as triethylamine or *N,N*-diisopropylethylamine. Substitution of the chlorine atom in compounds **31** and **32,** leading to obtaining compounds **33** and **34**, as well as the removal of the acetyl protecting groups in the sugar part in order to obtain compounds **35** or **38**, was carried out under the same conditions as described above. Structures of sugar derivatives, obtained with high yields, were confirmed by NMR spectra analysis. The spectroscopic data of obtained compounds are presented in the experimental section.

**Scheme 4:**
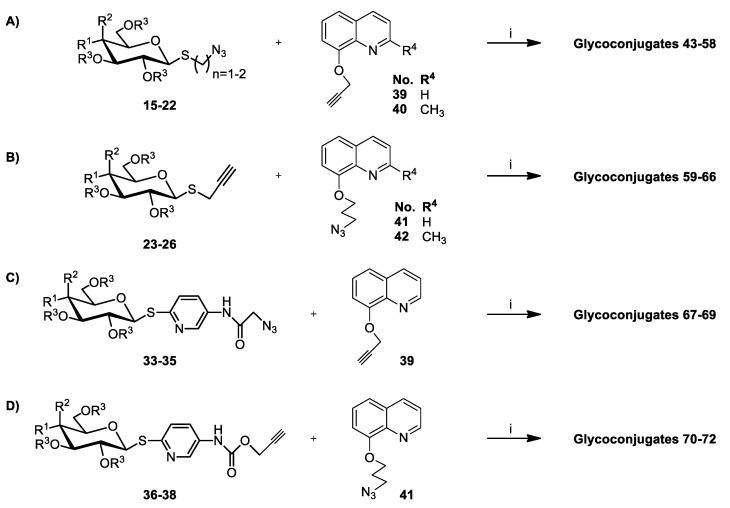
Synthesis of glycoconjugates **43**–**58** (**A**), **59**–**66** (**B**), **67**–**69** (**C**), and **70**–**72** (**D**). Reagents and Conditions: (**i**) CuSO_4_·5H_2_O, NaAsc, i-PrOH, THF, H_2_O, r.t., 24 h.

**Figure 2:**
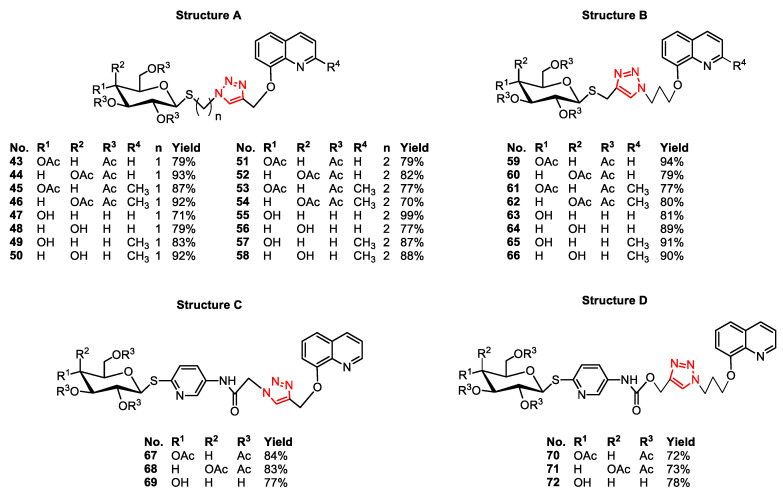
Structures and yields of obtained glycoconjugates **43**–**72**.

For the synthesis of glycoconjugates, derivatives of 1-thioglycosides were used both with the protected and deprotected hydroxyl groups in the sugar part. As the second structural element of the glycoconjugates, corresponding derivatives of 8-hydroxyquinoline **39**–**42** functionalized in the 8-OH position with propargyl or azide groups were used, obtained according to previously published procedures [27,46]. Target glycoconjugates were prepared using the copper(I)-catalyzed 1,3-dipolar azide-alkyne cycloaddition, described by Sharpless [47,48]. In these reactions, equimolar amounts of sugar derivatives and 8-HQ derivatives were combined in the configuration shown in Scheme 4. The use of CuSO_4_·5H_2_O as a catalyst allowed the carrying out of the reaction at room temperature and led to obtaining only 1,4-disubstituted 1,2,3-triazoles. Sodium ascorbate (NaAsc), used as a reducing agent of Cu(II) to Cu(I), avoided the formation of oxidation byproducts. All of the reagents were dissolved in the THF:*i*-PrOH:H_2_O solvent system (tetrahydrofuran:isopropyl alcohol:water). The progress of the reaction was controlled by TLC analysis so that the moment of the total conversion of the substrates could be estimated. After purification of the crude products by column chromatography, in the result, pure glycoconjugates **43**–**72** with high yields were obtained (Figure 2). The structures of all prepared glycoconjugates were confirmed using NMR and HRMS spectroscopy methods. The formation of the glycoconjugate was evidenced by the presence of a singlet at about δ = 7.9 ppm from the H-C(5) proton triazole ring in ^1^H NMR spectra and two characteristic carbon signals at about 123 ppm and 144 ppm for C(4) and C(5) from triazole ring in the ^13^C NMR spectra, as well as the presence of signals characteristic of the sugar and quinoline fragments. The obtained glycoconjugates contained the sugar unit with a binding of the β-configuration at the anomeric center since such orientation is preferred for binding to the GLUT transporters overexpressed in the cancer cells [13]. Such a structure was confirmed by the large coupling constant from the H-1 proton, equal to about *J* = 9.0 Hz, observed in the ^1^H NMR spectra. The NMR spectra of all the synthesized glycoconjugates are presented in the supporting information. Their physicochemical properties, such as melting point and optical rotation, were also determined.

### 2.2. Cytotoxicity Studies

*In vitro* cytotoxicity studies of the obtained building blocks and glycoconjugates were carried out on cell lines characterized by the high expression of GLUT transporters and the strongly changed glucose metabolism (strong Warburg effect) [10,49,50,51]. Based on this, colon cancer cell line (HCT-116) and breast cancer cell line (MCF-7) were selected for the research. Compounds with a high ability to inhibit the proliferation of tumor cells were also tested against the Normal Human Dermal Fibroblast-Neonatal cells (NHDF-Neo) to assess the safety of their use. Cytotoxicity was evaluated using the MTT assay. The results of the screening tests were used to determine the IC_50_ values (the half maximal inhibitory concentration), which are summarized in Table 1 and Table 2. These values represented 50% inhibition of cell growth concerning the control system, which were the cells suspended in the medium supplemented with DMSO in the amount used to dissolve the tested compounds.

The screening tests of sugar substrates used for glycoconjugation indicated no toxicity of compounds **15**–**26** and significant cytotoxicity of compounds **33** and **36** (Table 1). Probably due to the small size, compounds **33** and **36** were able to penetrate through phospholipid membranes into cells by passive diffusion. On the other hand, the presence of additional fragments in their structure capable of coordinating divalent metal ions (an amidopyridyl part), compared to substrates **15**–**26**, affected their ability to inhibit cancer cell growth. As it turned out, these compounds were also highly toxic to healthy cells. Interestingly, the d-galactose derivatives **34** and **37,** as well as the compounds **35** and **38,** without the acetyl protection of the hydroxyl groups were much less toxic and showed no anti-proliferative activity. In the case of the deprotected derivatives **35** and **38**, this result could be explained by the fact that they are too little lipophilic to enter the cell by passive transport and simultaneously, apparently, do not have sufficient affinity for GLUT transporters. However, the results obtained for per-*O*-acetylated galactoconjugates **34** and **37** were surprising. The significant difference in glycoconjugates cytotoxic activity observed with a simple replacement of the d-glucose residue with d-galactose might indicate a significant influence of the type of sugar residue on the biological activity of the whole compound. Cytotoxicity studies of 8-HQ and 2Me8HQ, the second building blocks of glycoconjugates, showed that both compounds were non-toxic to HCT-116 cells and toxic to MCF-7 cells. 8-HQ appeared to be of particular interest as it exhibited high toxicity against the breast cancer cell line, while not being toxic against the NHDF normal cell line. Thus, this compound is a good starting point for structural modifications that should further improve its activity.

Taking into account the obtained results, it seemed necessary to develop a prodrug that would improve the selectivity of a given compound. By starting to design prodrugs by functionalizing the biologically active compound, we focused on differences in the properties of cancer and normal cells. In this case, we paid attention to factors that increase the affinity of the prodrug for the interphase space. It is known that there is a mildly acidic environment in cancer tissues that results from excessive glycolysis in tumors (Warburg effect) [52,53]. In the case of healthy cells, this acidic environment does not exist. Therefore, the presence of the 1,2,3-triazole ring having base properties should increase the concentration of the prodrug at the interphase space of the cancer cells.

Dose-dependent cytotoxicity was observed for glycoconjugates derivatives of 8-HQ containing a 1,2,3-triazole fragment in the structure. The summary of cytotoxicity of tested glycoconjugates **43**–**72** is shown in Table 2. In order to approximate the mechanism of glycoconjugate transport into the cell, the influence of the presence of acetyl protecting groups in the sugar part of the glycoconjugate on the activity was examined. High IC_50_ values of glycoconjugates with a deprotected sugar fragment (**47**–**50**, **55**–**58**, **63**–**66**) indicated that these compounds were not able to bind to glucose transporters and be transported by them across biological membranes. Glycoconjugates with acetyl protecting groups in the sugar part (**43**–**46**, **51**–**54**, **59**–**62**) turned out to be more active. This suggested that due to their higher lipophilicity, they could cross the phospholipid bilayer by passive transport. Derivatives **43**, **44**, and **51**, **52** revealed interesting antiproliferative properties against the tested tumor cell lines. Unfortunately, at the same time, they also turned out to be toxic to healthy cells (NHDF-Neo). This was probably because this type of transport is not preferred in the case of the designed prodrugs, as it does not guarantee selectivity, and, as a result, the tested compounds damage both cancer cells as well as healthy cells.

**Figure 3:**
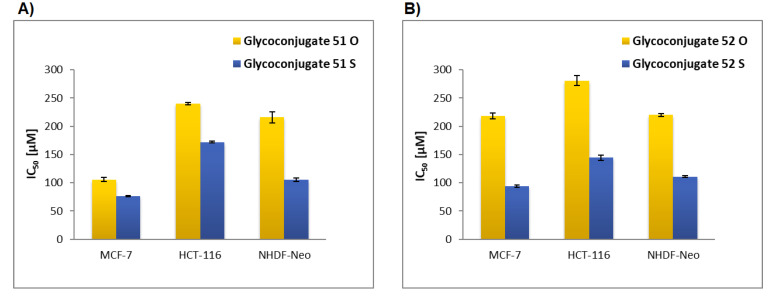
Comparison of IC_50_ activity glycoconjugates **51** (**A**) and **52** (**B**) containing sulfur or oxygen atom at the sugar anomeric position.

As part of the research, the effect of introducing a sulfur atom into the anomeric position of sugar was assessed. Glycoconjugates **51**–**54** derivatives of azidoalkyl 1-thioglycosides showed higher cytotoxic activity than the analogous glycoconjugates obtained earlier containing oxygen atom at the sugar anomeric position [27]. This was particularly evident for the 8-HQ derivatives **51** and **52**, for which the cytotoxic activity was about twice as high as that of their structural counterparts with an anomeric oxygen atom (Figure 3). Unfortunately, an increase in cytotoxicity for glycoconjugates containing anomeric sulfur was observed, both to neoplastic cells and to healthy cells. So, it can be assumed that their stability in the presence of hydrolytic enzymes affects their activity and may suggest that they were not prematurely degraded before entering the cell. In order to verify this assumption, two galactoconjugates **56** and **64** (being the deprotected counterparts of galactoconjugates **52** and **60**) and their two analogs with anomeric oxygen were chosen and subjected to hydrolysis reactions using β-galactosidase from *Aspergillus oryzae* [54]. The conducted experiments, for which the description of the procedure is attached to Supplementary Materials, showed that glycoconjugates **56** and **64**, containing the sulfur atom in the anomeric position, did not undergo hydrolysis in the presence of the enzyme even after 24 h of the reaction, while their counterparts with the anomeric oxygen had completely undergone hydrolysis after just 60 min of the enzymatic reaction.

In the case of glycoconjugates **59**–**62**, their higher cytotoxicity, compared to the oxygen counterparts, was not observed, despite the fact that also for a representative of these compounds, the positive influence of the anomeric sulfur on the hydrolytic stability was confirmed experimentally. For glycoconjugates **59**–**62**, to form a 1,2,3-triazole system in their structure, acetylated derivatives of 1-thiosugars with an anomeric propargyl moiety were used. In this reaction, an “inverted” 1,2,3-triazole system was formed in relation to the one that was in the glycoconjugates **51**–**54**. It can be assumed that for the cytotoxicity of glycoconjugates, not only their hydrolytic stability is important but also the mutual spatial orientation of the atom in the anomeric position of the sugar (sulfur or oxygen) and the 1,2,3-triazole ring, but confirmation of this thesis requires further research.

The length of the aliphatic chain between the sugar and the 1,2,3-triazole ring did not significantly affect the activity of glycoconjugates. It was found that d-glucose derivatives, in most cases, were more active than d-galactose derivatives. Among them, greater cytotoxicity was demonstrated by derivatives whose structure was based on the 8-HQ fragment, compared to derivatives with 2Me8-HQ units. Higher antiproliferative activity of the tested compounds was found against the breast cancer cell line (MCF-7). In this case, high toxicity to the cells of this line was observed for the quinoline aglycones themselves. This indicated the huge sensitivity of the cells of this particular tumor line to 8-HQ.

Previously, we showed that 8-HQ derivative glycoconjugates containing 1,2,3-triazole fragment in the linker structure were able to form complexes with copper ions and potentially inhibit the multiplication of neoplastic cells by eliminating an important factor for their growth [27]. In the current study, we decided to enhance this effect by creating new analogs, with an additional heteroaromatic fragment in the structure **67**–**72**. The presence of sulfur, a pyridine ring, and an additional amide bond in the structure of glycoconjugate should improve their cytotoxic activity as their method of action may be related to metal ion chelation. The protected glycoconjugates **67**, **68**, and **70**, **71** showed good antiproliferative activity against HCT-116 and MCF-7 cancer cells. The unprotected glucose derivative **69** showed moderate activity. Whereas, unprotected glucoconjugate **72** was not active in the concentration range studied. Unfortunately, active compounds showed toxic activity also to the NHDF-Neo cell line. Interestingly, the discussed compounds were created by connecting exhibiting cytotoxic activity substrates **33** and **36** with quinoline derivatives **39** or **41**. The cytotoxicity of the resulting glycoconjugates was expected to increase relative to the substrates themselves, but this did not happen. Perhaps the large size of the resulting glycoconjugates is one of the possible explanations for the observed activity decrease.

Cultured cancer cells, in contrast to *in vivo* environment, are characterized by a low copper level [55,56]. Therefore, additional experiments were carried out for compounds whose expected mechanism of action is based on the chelation of metal ions. The antiproliferative activities of glycoconjugates **67**, **68**, **70**, and **71** were tested in the presence of Cu^2+^ ions. The effect of the interaction of glycoconjugate complexes with metal ions on cell proliferation was measured by adding a copper salt solution to the growth medium and observing the rate of cell growth compared to copper-untreated cells.

First, measurements were made of the effect of different concentrations of copper on the proliferation of cancer and non-cancer cells. Cell cultures under standard conditions were treated for 24 h or 72 h with copper solutions of various concentrations, and their effect on cell proliferation was verified by the MTT assay. Cells with the non-supplemented medium were used as controls. For further experiments, a 100 µM copper acetate solution was used, as it did not affect the viability of any of the tested cell lines in any way.

During the appropriate experiments, the cells (MCF-7, HCT-116, and NHDF-Neo) were treated with solutions of glycoconjugate **67**, **68**, **70**, or **71** in medium culture, supplemented with 100 µM copper acetate, and then their proliferation was measured by the MTT assay. The control system was cells suspended in medium supplemented with DMSO in the amount used to dissolve the compounds and the addition of the same amount of copper. It turned out that cancer cells treated with glycoconjugates in the presence of Cu^2+^ had a much slower growth rate compared to cells treated with free glycoconjugates in the absence of copper. The highest cytotoxic activity of the compounds was observed against the MCF-7 cell line. Their IC_50_ values ranged from 20.51 ± 1.55 µM to 28.94 ± 1.52 µM (Table 2). This confirmed the strong sensitivity of breast cancer cells to 8-HQ derivatives.

**Figure 4:**
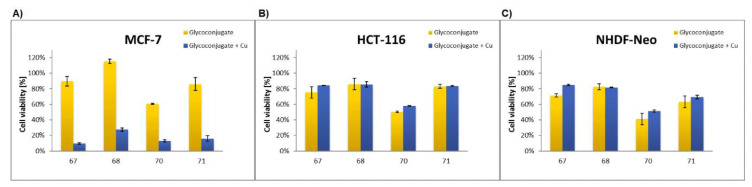
The cell viability treated with free glycoconjugate at 50 µM and glycoconjugates with the addition of Cu^2+^ at 100 µM for MCF-7 (**A**), HCT-116 (**B**), and NHDF-Neo (**C**) cells after 24 h (HCT-116 and NHDF-Neo) or 72 h (MCF-7) incubation. Data are presented as the mean ± standard deviation (*n* = 3).

**Figure 5:**
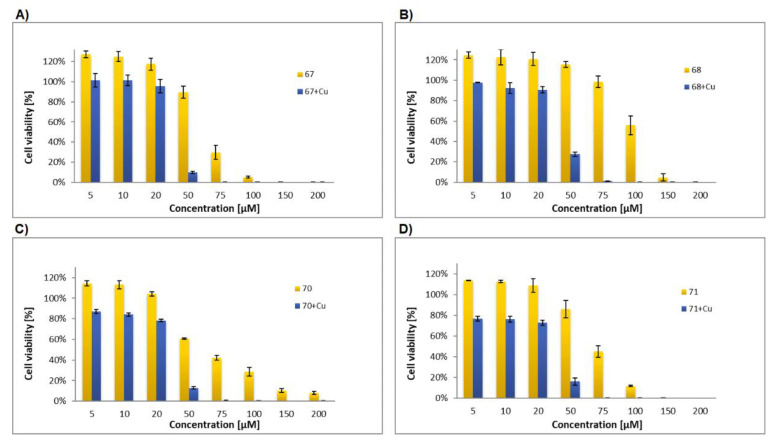
The dependence of MCF-7 cell proliferation on the concentration of the potential inhibitor 67 (**A**), 68 (**B**), 70 (**C**) or 71 (**D**) in the absence and presence of Cu^2+^.

Figure 4 shows the cell viability treated with free glycoconjugate at 50 µM and glycoconjugates with the addition of Cu^2+^ at 100 µM for MCF-7, HCT-116, and NHDF-Neo cells after 24 h (HCT-116 and NHDF-Neo) or 72 h (MCF-7) of incubation. It can be seen that the greatest differences in cell survival occurred in the case of MCF-7 cell lines. The detailed dependence of MCF-7 cells’ proliferation on the concentration of a potential inhibitor is shown in Figure 5. A significant decrease in the proliferation of MCF-7 cells was observed after treatment with glycoconjugates at a concentration of 50 µM in the presence of Cu^2+^ (cell viability 10–28%), compared to experiments with glycoconjugates alone. In this case, the cytotoxicity of glycoconjugates increased about three-fold in the presence of Cu(II) ions. Lower concentrations of glycoconjugates also improved antiproliferative activity in the presence of Cu(II), while concentrations above 50 µM led to complete inhibition of tumor cell growth. For HCT-116 and NHDF-Neo cell lines, only slight differences in cell viability were observed after treatment with free compounds and when Cu(II)(II) ions were added. This suggested that the cells’ viability of these lines was not affected by the concentration of copper ions to the same extent as it was for the breast cancer cell line.

## 3. Materials and Methods

### 3.1. General Information

NMR spectra were recorded with an Agilent spectrometer at a frequency of 400 MHz using TMS (tetramethylsilane) as the internal standards and CDCl_3_, CD_3_OD, or DMSO-d6 as the solvents. NMR solvents were purchased from ACROS Organics (Geel, Belgium). Chemical shifts (δ) were expressed in ppm and the coupling constants (*J*) in Hz. The following abbreviations were used to explain the observed multiplicities: s: singlet, d: doublet, dd: doublet of doublets, ddd: doublet of doublet of doublets, t: triplet, dd~t: doublet of doublets resembling a triplet (with similar values of coupling constants), m: multiplet, p: pentet (quintet), b: broad. High-resolution mass spectra (HRMS) were recorded with a WATERS LCT Premier XE system using the electrospray-ionization (ESI) technique. Optical rotations were measured with a JASCO P-2000 polarimeter using a sodium lamp (589.3 nm) at room temperature. Melting point measurements were performed on OptiMelt (MPA 100) Stanford Research Systems. Reactions were monitored by thin-layer chromatography (TLC) on precoated plates of silica gel 60 F254 (Merck Millipore, Burlington, MA, USA). The TLC plates were visualized under UV light (λ = 254 nm) or by charring the plates after spraying with a 10% solution of sulfuric acid in ethanol. Crude products were purified using column chromatography performed on Silica Gel 60 (70–230 mesh, Fluka, St. Louis, MI, USA), developed using toluene:EtOAc or CHCl_3_:MeOH as solvent systems. All evaporations were performed on a rotary evaporator under diminished pressure at 40 °C. The absorbance on MTT assay was measured spectrophotometrically at the 570 nm wavelength using a plate reader (Epoch, BioTek, Winooski, VT, USA).

All used chemicals were purchased from Sigma-Aldrich (Saint Louis, MO, USA, ACROS Organics (Geel, Belgium), and Avantor (Gliwice, Poland) and were used without purification. d-Glucose, d-galactose, 8-hydroxyquinoline, and 8-hydroxyquinaldine are commercially available (Sigma-Aldrich). 2,3,4,6-Tetra-*O*-acetyl-1-thio-β-d-glycopyranoses **9**, **10 [41]**, chloromethyl- **11, 12** and azidomethyl 2,3,4,6-tetra-*O*-acetyl-1-thio-β-d-glycopyranosides **15**, **16** [42], 2-chloroethyl- **13, 14** and 2-azidoethyl 2,3,4,6-tetra-*O*-acetyl-1-thio-β-d-glycopyranosides **17, 18 [42]**, propargyl 2,3,4,6-tetra-*O*-acetyl-1-thio-β-d-glycopyranosides **23**, **24 [43]**, (5-amino-2-pyridyl) 2,3,4,6-tetra-*O*-acetyl-1-thio-β-d-glycopyranosides **29**, **30** [45], 8-(2-propyn-1-yloxy)quinoline **39** [46], 2-methyl-8-(2-propyn-1-yloxy)quinoline **40** [46], 8-(3-azidopropoxy)quinolone **41** [27], and 2-methyl-8-(3-azidopropoxy)quinolone **42** [27] were prepared according to the respective published procedures.

### 3.2. Chemistry

#### 3.2.1. General Procedure for the Synthesis of Sugar Derivatives **31** and **32**

To a solution of (5-amino-2-pyridyl) 2,3,4,6-tetra-*O*-acetyl-1-thio-β-d-glucopyranoside **29** or (5-amino-2-pyridyl) 2,3,4,6-tetra-*O*-acetyl-1-thio-β-d-galactopyranoside **30** (685 mg, 1.5 mmol) in dry CH_2_Cl_2_ (30 mL), triethylamine (625 µL, 4.5 mmol) was added. The reaction mixture was cooled to 0 °C, and chloroacetyl chloride was added dropwise (175 μL, 2.2 mmol), then stirring was continued at room temperature. After 1 h, the resulting mixture was diluted with dichloromethane (50 mL) and washed with brine (2 × 50 mL). The combined organic phases were dried over anhydrous MgSO_4_, filtered, and concentrated in vacuo. The crude products were purified by column chromatography (toluene:AcOEt; 2:1) to give products **31**–**32**.

*(5-chloroacetamide-2-pyridyl) 2,3,4,6-tetra-O-acetyl-1-thio-β-d-glucopyranoside***31***: Starting from (5-amino-2-pyridyl) 2,3,4,6-tetra-O-acetyl-1-thio-β-d-glucopyranoside***29***, the product was obtained as an orange solid (600 mg, 75%)*, m.p.: 145–146 °C; [α]^25^_D_ = 0.4 (c = 1.0, CHCl_3_); ^1^H NMR (400 MHz, CDCl_3_): δ 2.02, 2.02, 2,04, 2.05 (4s, 12H, CH_3_CO), 3.86 (ddd, 1H, *J* = 2.3 Hz, *J* = 4.6 Hz, *J* = 10.1 Hz, H-5_Glu_), 4.10 (dd, 1H, *J* = 2.3 Hz, *J* = 12.4 Hz, H-6a_Glu_), 4.22 (s, 2H, CH_2_Cl), 4.27 (dd, 1H, *J* = 4.6 Hz, *J* = 12.4 Hz, H-6b_Glu_), 5.15 (dd~t, 1H, *J* = 9.4 Hz, *J* = 10.4 Hz, H-2_Glu_), 5.20 (dd~t, 1H, *J* = 9.3 Hz, *J* = 10.1 Hz, H-4_Glu_), 5.34 (dd~t, 1H, *J* = 9.3 Hz, *J* = 9.4 Hz, H-3_Glu_), 5.72 (d, 1H, *J* = 10.4 Hz, H-1_Glu_), 7.26 (d, 1H, *J* = 8.6 Hz, H-3_Pyr_), 7.97 (dd, 1H, *J* = 2.6 Hz, *J* = 8.6 Hz, H-4_Pyr_), 8.29 (bs, 1H, NH), 8.59 (d, 1H, *J* = 2.6 Hz, H-6_Pyr_); ^13^C NMR (100 MHz, CDCl_3_): δ 20.60, 20.62, 20.69, 20.75 (CH_3_CO), 42.72 (CH_2_Cl), 61.94 (C-6_Glu_), 68.24, 69.47, 74.09, 75.97 (C-2_Glu_, C-3_Glu_, C-4_Glu_, C-5_Glu_), 82.11 (C-1_Glu_), 123.65, 128.60, 131.53, 141.37, 151.09 (C_Pyr_), 164.29 (C=O) 169.42, 169.50, 170.15, 170.66 (CH_3_CO); HRMS (ESI-TOF): calcd for C_21_H_26_N_2_O_10_SCl ([M + H]^+^): *m*/*z* 533.0997; found: *m*/*z* 533.0997.

*(5-chloroacetamide-2-pyridyl) 2,3,4,6-tetra-O-acetyl-1-thio-β-d-galactopyranoside***32***: Starting from (5-amino-2-pyridyl) 2,3,4,6-tetra-O-acetyl-1-thio-β-d-galactopyranoside***30***, the product was obtained as a beige solid (632 mg, 79%)*, m.p.: 49–51 °C; [α]^25^_D_ = 13.6 (c = 1.0, CHCl_3_); ^1^H NMR (400 MHz, CDCl_3_): δ 2.00, 2.01, 2,03, 2.17 (4s, 12H, CH_3_CO), 4.00–4.16 (m, 3H, H-5_Gal_, H-6a_Gal_, H-6b_Gal_), 4.22 (s, 2H, CH_2_Cl), 5.18 (dd, 1H, *J* = 3.4 Hz, *J* = 9.9 Hz, H-3_Gal_), 5.40 (dd~t, 1H, *J* = 9.9 Hz, *J* = 10.4 Hz, H-2_Gal_), 5.49 (dd, 1H, *J* = 0.7 Hz, *J* = 3.4 Hz, H-4_Gal_), 5.69 (d, 1H, *J* = 10.4 Hz, H-1_Gal_), 7.28 (d, 1H, *J* = 8.6 Hz, H-3_Pyr_), 7.97 (dd, 1H, *J* = 2.6 Hz, *J* = 8.6 Hz, H-4_Pyr_), 8.27 (s, 1H, NH), 8.57 (d, 1H, *J* = 2.6 Hz, H-6_Pyr_); ^13^C NMR (100 MHz, CDCl_3_): δ 20.60, 20.68, 20.70, 20.78 (CH_3_CO), 42.72 (CH_2_Cl), 61.29 (C-6_Gal_), 66.82, 67.30, 72.07, 74.58 (C-2_Gal_, C-3_Gal_, C-4_Gal_, C-5_Gal_), 82.67 (C-1_Gal_), 123.59, 128.54, 131.47, 141.47, 151.36 (C_Pyr_), 164.30 (C=O) 169.70, 170.02, 170.26, 170.36 (CH_3_CO); HRMS (ESI-TOF): calcd for C_21_H_26_N_2_O_10_SCl ([M + H]^+^): *m*/*z* 533.0997; found: *m*/*z* 533.1002.

#### 3.2.2. General Procedure for the Synthesis of Sugar Derivatives **33** and **34**

To a solution of compounds **31** or **32** (320 mg, 0.6 mmol) in dry DMF (10 mL), sodium azide (214 mg, 3.3 mmol) was added. The reaction mixture was stirred at room temperature for 24 h. After completion of the reaction, the solvent was evaporated under reduced pressure, and the residue was dissolved in ethyl acetate (50 mL) and extracted with water (2 × 30 mL) and brine (30 mL). The combined organic phases were dried over anhydrous MgSO_4_, filtered, and concentrated in vacuo to afford the corresponding azide **33** and **34**, which were used for the next reaction without further purification.

*(5-azidoacetamide-2-pyridyl) 2,3,4,6-tetra-O-acetyl-1-thio-β-d-glucopyranoside***33***: Starting from (5-chloroacetamide-2-pyridyl) 2,3,4,6-tetra-O-acetyl-1-thio-β-d-glucopyranoside***31***, the product was obtained as an orange solid (288 mg, 89%)*, m.p.: 75–78 °C; [α]^25^_D_ = 11.4 (c = 1.0, CHCl_3_); ^1^H NMR (400 MHz, CDCl_3_): δ 2.02, 2.04, 2,04, 2.05 (4s, 12H, CH_3_CO), 3.86 (ddd, 1H, *J* = 2.3 Hz, *J* = 4.6 Hz, *J* = 10.1 Hz, H-5_Glu_), 4.10 (dd, 1H, *J* = 2.3 Hz, *J* = 12.4 Hz, H-6a_Glu_), 4.19 (s, 2H, CH_2_N_3_), 4.27 (dd, 1H, *J* = 4.6 Hz, *J* = 12.4 Hz, H-6b_Glu_), 5.15 (dd~t, 1H, *J* = 9.3 Hz, *J* = 10.4 Hz, H-2_Glu_), 5.20 (dd~t, 1H, *J* = 9.3 Hz, *J* = 10.1 Hz, H-4_Glu_), 5.34 (dd~t, 1H, *J* = 9.3 Hz, *J* = 9.3 Hz, H-3_Glu_), 5.71 (d, 1H, *J* = 10.4 Hz, H-1_Glu_), 7.25 (d, 1H, *J* = 8.6 Hz, H-3_Pyr_), 7.97 (dd, 1H, *J* = 2.6 Hz, *J* = 8.6 Hz, H-4_Pyr_), 8.07 (bs, 1H, NH), 8.57 (d, 1H, *J* = 2.6 Hz, H-6_Pyr_); ^13^C NMR (100 MHz, CDCl_3_): δ20.60, 20.63, 20.69, 20.75 (CH_3_CO), 52.85 (CH_2_N_3_), 61.95 (C-6_Glu_), 68.26, 69.49, 74.10, 75.96 (C-2_Glu_, C-3_Glu_, C-4_Glu_, C-5_Glu_), 82.15 (C-1_Glu_), 123.59, 128.41, 131.53, 141.33, 150.91 (C_Pyr_), 164.88 (C=O) 169.42, 169.50, 170.15, 170.66 (CH_3_CO); HRMS (ESI-TOF): calcd for C_21_H_26_N_5_O_10_S ([M + H]^+^): *m*/*z* 540.1400; found: *m*/*z* 540.1396.

*(5-azidoacetamide-2-pyridyl) 2,3,4,6-tetra-O-acetyl-1-thio-β-d-galactopyranoside***34***: Starting from (5-chloroacetamide-2-pyridyl) 2,3,4,6-tetra-O-acetyl-1-thio-β-d-galactopyranoside***32***, the product was obtained as a beige solid (278 mg, 86%)*, m.p.: 57–59 °C; [α]^25^_D_ = 17.0 (c = 0.4, CHCl_3_); ^1^H NMR (400 MHz, CDCl_3_): δ 2.00, 2.01, 2,03, 2.17 (4s, 12H, CH_3_CO), 4.04–4.16 (m, 3H, H-5_Gal_, H-6a_Gal_, H-6b_Gal_), 4.19 (s, 2H, CH_2_N_3_), 5.18 (dd, 1H, *J* = 3.4 Hz, *J* = 9.9 Hz, H-3_Gal_), 5.40 (dd~t, 1H, *J* = 9.9 Hz, *J* = 10.2 Hz, H-2_Gal_), 5.48 (dd, 1H, *J* = 0.7 Hz, *J* = 3.4 Hz, H-4_Gal_), 5.68 (d, 1H, *J* = 10.4 Hz, H-1_Gal_), 7.27 (d, 1H, *J* = 8.6 Hz, H-3_Pyr_), 7.98 (dd, 1H, *J* = 2.7 Hz, *J* = 8.6 Hz, H-4_Pyr_), 8.08 (bs, 1H, NH), 8.57 (dd, 1H, *J* = 0.6 Hz, *J* = 2.7 Hz, H-6_Pyr_); ^13^C NMR (100 MHz, CDCl_3_): δ 20.60, 20.68, 20.70, 20.78 (CH_3_CO), 52.84 (CH_2_N_3_), 61.29 (C-6_Gal_), 66.83, 67.30, 72.07, 74.57 (C-2_Gal_, C-3_Gal_, C-4_Gal_, C-5_Gal_), 82.71 (C-1_Gal_), 123.62, 128.43, 131.56, 141.35, 151.05 (C_Pyr_), 164.94 (C=O) 169.69, 170.02, 170.25, 170.35 (CH_3_CO); HRMS (ESI-TOF): calcd for C_21_H_26_N_5_O_10_S ([M + H]^+^): *m*/*z* 540.1400; found: *m*/*z* 540.1403.

#### 3.2.3. General Procedure for the Synthesis of Sugar Derivatives **36** and **37**

To a solution of (5-amino-2-pyridyl) 2,3,4,6-tetra-*O*-acetyl-1-thio-β-d-glucopyranoside **29** or (5-amino-2-pyridyl) 2,3,4,6-tetra-*O*-acetyl-1-thio-β-d-galactopyranoside **30** (274 mg, 0.6 mmol) in dry CH_2_CL_2_ (20 mL), *N,N*-diisopropylethylamine (347 µL, 2.1 mM) was added. The reaction mixture was cooled to 0 °C, and propargyl chloroformate was added dropwise (117 μL, 1.2 mmol), then stirring was continued at room temperature. After 2 h, the resulting mixture was diluted with dichloromethane (60 mL) and washed with 1M HCl (40 mL), saturated NaHCO_3_ (40 mL), and brine (40 mL). The combined organic phases were dried over anhydrous MgSO_4_, filtered, and concentrated in vacuo. The crude products were purified by column chromatography (toluene:AcOEt; 5:1) to give products **36**–**37**.

*(5-(((prop-2-yn-1-yloxy)carbonyl)amino)-2-pyridyl) 2,3,4,6-tetra-O-acetyl-1-thio-β-d-glucopyranoside***36***: Starting from (5-amino-2-pyridyl) 2,3,4,6-tetra-O-acetyl-1-thio-β-d-glucopyranoside***29***, the product was obtained as an orange solid (265 mg, 82%)*, m.p.: 162–165 °C; [α]^26^_D_ = 0.9 (c = 0.46, CHCl_3_); ^1^H NMR (400 MHz, CDCl_3_): δ 2.02, 2.02, 2,03, 2.05 (4s, 12H, CH_3_CO), 2.54 (t, 1H, *J* = 2.5 Hz, CH), 3.85 (ddd, 1H, *J* = 2.3 Hz, *J* = 4.6 Hz, *J* = 10.1 Hz, H-5_Glu_), 4.10 (dd, 1H, *J* = 2.3 Hz, *J* = 12.4 Hz, H-6a_Glu_), 4.26 (dd, 1H, *J* = 4.6 Hz, *J* = 12.4 Hz, H-6b_Glu_), 4.80 (d, 2H, *J* = 2.5 Hz, CH_2_), 5.15 (dd~t, 1H, *J* = 9.3 Hz, *J* = 10.4 Hz, H-2_Glu_), 5.19 (dd~t, 1H, *J* = 9.3 Hz, *J* = 10.1 Hz, H-4_Glu_), 5.33 (dd~t, 1H, *J* = 9.3 Hz, *J* = 9.3 Hz, H-3_Glu_), 5.67 (d, 1H, *J* = 10.4 Hz, H-1_Glu_), 6.86 (bs, 1H, NH), 7.23 (d, 1H, *J* = 8.2 Hz, H-4_Pyr_), 7.84 (d, 1H, *J* = 8.2 Hz, H-3_Pyr_), 8.43 (d, 1H, *J* = 2.5 Hz, H-6_Pyr_); ^13^C NMR (100 MHz, CDCl_3_): δ 20.61, 20.63, 20.70, 20.75 (CH_3_CO), 53.20 (CH_2_), 61.96 (C-6_Glu_), 68.27, 69.55, 74.11, 75.44 (C-2_Glu_, C-3_Glu_, C-4_Glu_, C-5_Glu_), 75.92, 77.40 (C≡CH), 82.37 (C-1_Glu_), 123.95, 127.24, 132.48, 140.31, 149.35 (C_Pyr_), 152.39 (C=O) 169.44, 169.52, 170.17, 170.68 (CH_3_CO); HRMS (ESI-TOF): calcd for C_23_H_27_N_2_O_11_S ([M + H]^+^): *m*/*z* 539.1336; found: *m*/*z* 539.1337.

*(5-(((prop-2-yn-1-yloxy)carbonyl)amino)-2-pyridyl 2,3,4,6-tetra-O-acetyl-1-thio-β-d-galactopyranoside***37***: Starting from (5-amino-2-pyridyl) 2,3,4,6-tetra-O-acetyl-1-thio-β-d-galactopyranoside***30***, the product was obtained as a beige solid (258 mg, 80%)*, m.p.: 53–55 °C; [α]^26^_D_ = 14.2 (c = 1.0, CHCl_3_); ^1^H NMR (400 MHz, CDCl_3_): δ 2.00, 2.00, 2,03, 2.17 (4s, 12H, CH_3_CO), 2.54 (t, 1H, *J* = 2.4 Hz, CH), 4.03–4.16 (m, 3H, H-5_Gal_, H-6a_Gal_, H-6b_Gal_), 4.80 (d, 2H, *J* = 2.4 Hz, CH_2_), 5.17 (dd, 1H, *J* = 3.4 Hz, *J* = 9.9 Hz, H-3_Gal_), 5.39 (dd~t, 1H, *J* = 9.9 Hz, *J* = 10.4 Hz, H-2_Gal_), 5.48 (dd, 1H, *J* = 0.9 Hz, *J* = 3.4 Hz, H-4_Gal_), 5.64 (d, 1H, *J* = 10.4 Hz, H-1_Gal_), 6.91 (bs, 1H, NH), 7.27 (d, 1H, *J* = 8.6 Hz, H-4_Pyr_), 7.85 (d, 1H, *J* = 8.6 Hz, H-3_Pyr_), 8.43 (d, 1H, *J* = 2.6 Hz, H-6_Pyr_); ^13^C NMR (100 MHz, CDCl_3_): δ 20.60, 20.67, 20.70, 20.79 (CH_3_CO), 53.19 (CH_2_), 61.31 (C-6_Gal_), 66.90, 67.31, 72.08, 74.54 (C-2_Gal_, C-3_Gal_, C-4_Gal_, C-5_Gal_), 75.44, 77.41 (C≡CH), 82.93 (C-1_Gal_), 123.98, 127.22, 132.49, 140.30, 149.52 (C_Pyr_), 152.42 (C=O) 169.71, 170.04, 170.28, 170.37 (CH_3_CO); HRMS (ESI-TOF): calcd for C_23_H_27_N_2_O_11_S ([M + H]^+^): *m*/*z* 539.1336; found: *m*/*z* 539.1340.

#### 3.2.4. General Procedure for the Synthesis of Sugar Derivatives **35** and **38**

(5-azidoacetamide-2-pyridyl) 2,3,4,6-tetra-*O*-acetyl-1-thio-β-d-glucopyranoside **33** or (5-(((prop-2-yn-1-yloxy)carbonyl)amino)-2-pyridyl 2,3,4,6-tetra-*O*-acetyl-1-thio-β-d-glucopyranoside **36** (0.5 mmol) was dissolved in MeOH (20 mL), and then 1 M solution of NaOMe in MeOH (200 µL, 0.2 mmol) was added. The reaction mixture was stirred for 1 h at room temperature. After the reaction mixture was neutralized with Amberlyst-15 ion exchange resin, the mixture was filtered off, and the filtrate was concentrated in vacuo to give compounds **35** and **38**, pure enough for further reactions.

*(5-azidoacetamide-2-pyridyl) 1-thio-β-d-glucopyranoside***35***: Starting from (5-azidoacetamide-2-pyridyl) 2,3,4,6-tetra-O-acetyl-1-thio-β-d-glucopyranoside***33***, the product was obtained as an orange solid (150 mg, 81%)*, m.p.: 74–75 °C; [α]^26^_D_ = -59.6 (c = 0.45, CH_3_OH); ^1^H NMR (400 MHz, CD_3_OD): δ 3.32–3.42 (m, 3H, H-2_Glu_, H-4_Glu_, H-5_Glu_), 3.44 (dd~t, 1H, *J* = 8.6 Hz, *J* = 8.7 Hz, H-3_Glu_), 3.66 (dd, 1H, *J* = 5.6 Hz, *J* = 12.1 Hz, H-6a_Glu_), 3.85 (dd, 1H, *J* = 2.2 Hz, *J* = 12.1 Hz, H-6b_Glu_), 4.05 (s, 2H, CH_2_N_3_), 5.11 (d, 1H, *J* = 9.9 Hz, H-1_Glu_), 7.48 (dd, 1H, *J* = 0.6 Hz, *J* = 8.7 Hz, H-3_Pyr_), 8.00 (dd, 1H, *J* = 2.6 Hz, *J* = 8.7 Hz, H-4_Pyr_), 8.63 (d, 1H, *J* = 2.6 Hz, H-6_Pyr_); ^13^C NMR (100 MHz, CD_3_OD): δ 53.21 (CH_2_N_3_), 62.81 (C-6_Glu_), 71.35, 73.92, 79.77, 82.23 (C-2_Glu_, C-3_Glu_, C-4_Glu_, C-5_Glu_), 86.83 (C-1_Glu_), 125.20, 130.13, 134.33, 142.20, 153.64 (C_Pyr_), 168.90 (C=O); HRMS (ESI-TOF): calcd for C_13_H_18_N_5_O_6_S ([M + H]^+^): *m*/*z* 372.0978; found: *m*/*z* 372.0979.

*(5-(((prop-2-yn-1-yloxy)carbonyl)amino)-2-pyridyl 1-thio-β-d-glucopyranoside***38***: Starting from (5-(((prop-2-yn-1-yloxy)carbonyl)amino)-2-pyridyl 2,3,4,6-tetra-O-acetyl-1-thio-β-d-glucopyranoside***36***, the product was obtained as an orange solid (181 mg, 98%)*, m.p.: 69–71 °C; [α]^26^_D_ = −52.8 (c = 1.0, CH_3_OH); ^1^H NMR (400 MHz, CD_3_OD): δ 2.94 (t, 1H, *J* = 2.5 Hz, CH), 3.31–3.35 (m, 2H, H-2_Glu_, H-4_Glu_), 3.39 (ddd, 1H, *J* = 2.3 Hz, *J* = 5.7 Hz, *J* = 9.7 Hz, H-5_Glu_), 3.43 (dd~t, 1H, *J* = 8.8 Hz, *J* = 8.8 Hz, H-3_Glu_), 3.66 (dd, 1H, *J* = 5.7 Hz, *J* = 12.2 Hz, H-6a_Glu_), 3.85 (dd, 1H, *J* = 2.3 Hz, *J* = 12.2 Hz, H-6b_Glu_), 4.78 (d, 2H, *J* = 2.5 Hz, CH_2_), 5.03 (d, 1H, *J* = 9.9 Hz, H-1_Glu_), 7.48 (d, 1H, *J* = 8.6 Hz, H-4_Pyr_), 7.89 (d, 1H, *J* = 8.6 Hz, H-3_Pyr_), 8.50 (d, 1H, *J* = 2.3 Hz, H-6_Pyr_); ^13^C NMR (100 MHz, CD_3_OD): δ 53.63 (CH_2_), 62.83 (C-6_Glu_), 71.36, 73.94, 76.32, 79.03 (C-2_Glu_, C-3_Glu_, C-4_Glu_, C-5_Glu_), 79.74, 82.22 (C≡CH), 87.15 (C-1_Glu_), 125.86, 128.62, 135.43, 140.94, 151.80 (C_Pyr_), 154.82 (C=O); HRMS (ESI-TOF): calcd for C_15_H_19_N_2_O_7_S ([M + H]^+^): *m*/*z* 371.0913; found: *m*/*z* 371.0911.

#### 3.2.5. General Procedure for the Synthesis of Glycoconjugates **43**–**72**

The appropriate sugar derivatives **15**–**26, 33**–**38** (1 eq.) and 8-hydroxyquinoline derivatives **39**–**41** (1 eq.) were dissolved in a dry solvent system: THF (2 mL) and *i*-PrOH (2 mL). The catalyst systems were prepared: sodium ascorbate (0.4 eq.) dissolved in H_2_O (1 mL) and CuSO_4_·5H_2_O (0.2 eq.) dissolved in H_2_O (1 mL), mixed, and immediately added to the reaction mixture. The reaction mixture was stirred for 24 h at room temperature. Then, the solvents were evaporated in vacuo, and the crude products were purified by column chromatography (dry loading; toluene:AcOEt, 2:1 and CHCl_3_:MeOH, 100:1 for fully protected glycoconjugates or CHCl_3_:MeOH, gradient: 50:1 to 2:1 for glycoconjugates with unprotected sugar part) to give products **43**–**72**.

*Glycoconjugate***43***: Starting from sugar derivative***15***and 8-HQ derivative***39***, the product was obtained as a beige solid (79% yield)*, m.p.: 191–196 °C; [α]^23^_D_ = −70.4 (c = 1.0, CHCl_3_); ^1^H NMR (400 MHz, CDCl_3_): δ 1.94, 1.99, 2.02, 2.05 (4s, 12H, CH_3_CO), 3.61 (ddd, 1H, *J* = 2.2 Hz, *J* = 4.8 Hz, *J* = 10.0 Hz, H-5_Glu_), 4.05 (dd, 1H, *J* = 2.2 Hz, *J* = 12.5 Hz, H-6a_Glu_), 4.17 (dd, 1H, *J* = 4.8 Hz, *J* = 12.5 Hz, H-6b_Glu_), 4.55 (d, 1H, *J* = 10.1 Hz, H-1_Glu_), 5.02 (dd~t, 1H, *J* = 9.2 Hz, *J* = 10.0 Hz, H-4_Glu_), 5.06 (dd~t, 1H, *J* = 9.4 Hz, *J* = 10.1 Hz, H-2_Glu_), 5.15 (dd~t, 1H, *J* = 9.2 Hz, *J* = 9.4 Hz, H-3_Glu_), 5.29 i 5.71 (qAB, 2H, *J* = 14.5 Hz, CH_2_N_3_), 5.58 (s, 2H, CH_2_O), 7.29 (dd, 1H, *J* = 1.9 Hz, *J* = 6.9 Hz, H-7_Quin_), 7.40–7.49 (m, 3H, H-3_Quin_, H-5_Quin_, H-6_Quin_), 7.94 (s, 1H, H-5_Triaz_), 8.14 (dd, 1H, *J* = 1.6 Hz, *J* = 8.3 Hz, H-4_Quin_), 8.93 (dd, 1H, *J* = 1.6 Hz, 4.1 Hz, H-2_Quin_); ^13^C NMR (100 MHz, CDCl_3_): δ 20.51, 20.55, 20.57, 20.74 (CH_3_CO), 48.05 (CH_2_N_3_), 61.58, 62.85 (C-6_Glu_, CH_2_O), 67.91, 69.77, 73.56, 76.20 (C-2_Glu_, C-3_Glu_, C-4_Glu_, C-5_Glu_), 81.48 (C-1_Glu_), 109.93 (C-7_Quin_), 120.44 (C-5_Quin_), 121.71 (C-3_Quin_), 123.20 (C-5_Triaz_), 126.68 (C-6_Quin_), 129.52 (C-4a_Quin_), 136.00 (C-4_Quin_), 140.32 (C-8a_Quin_), 145.12 (C-4_Triaz_), 149.42 (C-2_Quin_), 155.63 (C-8_Quin_), 169.35, 169.40, 169.98, 170.54 (CH_3_CO); HRMS (ESI-TOF): calcd for C_27_H_31_N_4_O_10_S ([M + H]^+^): *m*/*z* 603.1761; found: *m*/*z* 603.1766.

*Glycoconjugate***44***: Starting from sugar derivative***16***and 8-HQ derivative***39***, the product was obtained as a beige solid (93% yield)*, m.p.: 92–96 °C; [α]^23^_D_ = −55.8 (c = 1.0, CHCl_3_); ^1^H NMR (400 MHz, CDCl_3_): δ 1.95, 1.97, 2.03, 2.15 (4s, 12H, CH_3_CO), 3.67 (ddd, 1H, *J* = 0.8 Hz, *J* = 6.0 Hz, *J* = 6.9 Hz, H-5_Gal_), 3.93 (dd, 1H, *J* = 6.0 Hz, *J* = 11.4 Hz, H-6a_Gal_), 3.98 (dd, 1H, *J* = 6.9 Hz, *J* = 11.4 Hz, H-6b_Gal_), 4.46 (d, 1H, *J* = 10.0 Hz, H-1_Gal_), 4.95 (dd, 1H, *J* = 3.4 Hz, *J* = 10.0 Hz, H-3_Gal_), 5.21 (dd~t, 1H, *J* = 10.0 Hz, *J* = 10.0 Hz, H-2_Gal_), 5.31 and 5.71 (qAB, 2H, *J* = 14.6 Hz, CH_2_N_3_), 5.33 (dd, 1H, *J* = 0.8 Hz, *J* = 3.4 Hz, H-4_Gal_), 5.60 (s, 2H, CH_2_O), 7.28 (dd, 1H, *J* = 2.7 Hz, *J* = 6.4 Hz, H-7_Quin_), 7.41–7.48 (m, 3H, H-3_Quin_, H-5_Quin_, H-6_Quin_), 7.94 (s, 1H, H-5_Triaz_), 8.15 (dd, 1H, *J* = 1.6 Hz, *J* = 8.3 Hz, H-4_Quin_), 8.93 (dd, 1H, *J* = 1.6 Hz, 4.1 Hz, H-2_Quin_); ^13^C NMR (100 MHz, CDCl_3_): δ20.52, 20.60, 20.65, 20.69 (CH_3_CO), 47.93 (CH_2_N_3_), 61.36, 62.85 (C-6_Gal_, CH_2_O), 66.98, 67.13, 71.54, 74.92 (C-2_Gal_, C-3_Gal_, C-4_Gal_, C-5_Gal_), 81.81 (C-1_Gal_), 109.96 (C-7_Quin_), 120.40 (C-5_Quin_), 121.74 (C-3_Quin_), 123.25 (C-5_Triaz_), 126.69 (C-6_Quin_), 129.53 (C-4a_Quin_), 135.98 (C-4_Quin_), 140.34 (C-8a_Quin_), 144.99 (C-4_Triaz_), 149.45 (C-2_Quin_), 153.71 (C-8_Quin_), 169.60, 169.81, 170.12, 170.26 (CH_3_CO); HRMS (ESI-TOF): calcd for C_27_H_31_N_4_O_10_S ([M + H]^+^): *m*/*z* 603.1761; found: *m*/*z* 603.1760.

*Glycoconjugate***45***: Starting from sugar derivative***15***and 8-HQ derivative***40***, the product was obtained as a white solid (87% yield)*, m.p.: 159–163 °C; [α]^23^_D_ = −84.8 (c = 1.0, CHCl_3_); ^1^H NMR (400 MHz, CDCl_3_): δ 1.94, 1.99, 2.02, 2.07 (4s, 12H, CH_3_CO), 2.78 (s, 3H, CH_3_), 3.55 (ddd, 1H, *J* = 1.9 Hz, *J* = 4.8 Hz, *J* = 10.1 Hz, H-5_Glu_), 4.01 (dd, 1H, *J* = 1.9 Hz, *J* = 12.5 Hz, H-6a_Glu_), 4.15 (dd, 1H, *J* = 4.8 Hz, *J* = 12.5 Hz, H-6b_Glu_), 4.52 (d, 1H, *J* = 10.1 Hz, H-1_Glu_), 5.01 (dd~t, 1H, *J* = 9.3 Hz, *J* = 10.1 Hz, H-4_Glu_), 5.04 (dd~t, 1H, *J* = 9.3 Hz, *J* = 10.1 Hz, H-2_Glu_), 5.14 (dd~t, 1H, *J* = 9.3 Hz, *J* = 9.3 Hz, H-3_Glu_), 5.28 i 5.71 (qAB, 2H, *J* = 14.6 Hz, CH_2_N_3_), 5.59 i 5.62 (qAB, 2H, *J* = 13.4 Hz, CH_2_O), 7.22 (dd, 1H, *J* = 1.4 Hz, *J* = 7.2 Hz, H-7_Quin_), 7.31 (d, 1H, *J* = 8.4 Hz, H-3_Quin_), 7.34–7.40 (m, 2H, H-5_Quin_, H-6_Quin_), 7.92 (s, 1H, H-5_Triaz_), 8.01 (d, 1H, *J* = 8.4 Hz, H-4_Quin_); ^13^C NMR (100 MHz, CDCl_3_): δ 20.51, 20.55, 20,57, 20.74 (CH_3_CO), 25.74 (CH_3_), 47.97 (CH_2_N), 61.57, 63.21 (C-6_Glu_, CH_2_O), 67.90, 69.79, 73.55, 76.17 (C-2_Glu_, C-3_Glu_, C-4_Glu_, C-5_Glu_), 81.33 (C-1_Glu_), 110.48 (C-7_Quin_), 120.41 (C-5_Quin_), 122.62 (C-3_Quin_), 123.04 (C-5_Triaz_), 125.61 (C-6_Quin_), 127.76 (C-4a_Quin_), 136.17 (C-4_Quin_), 139.89 (C-8a_Quin_), 145.46 (C-4_Triaz_), 153.23 (C-2_Quin_), 158.29 (C-8_Quin_), 169.33, 169.40, 169.96, 170.51 (CH_3_CO); HRMS (ESI-TOF): calcd for C_28_H_33_N_4_O_10_S ([M + H]^+^): *m*/*z* 617.1917; found: *m*/*z* 617.1915.

*Glycoconjugate***46***: Starting from sugar derivative***16***and 8-HQ derivative***40***, the product was obtained as a beige solid (92% yield)*, m.p.: 65–68 °C; [α]^23^_D_ = −34.8 (c = 1.0, CHCl_3_); ^1^H NMR (400 MHz, CDCl_3_): δ 1.95, 1.97, 2.06, 2.14 (4s, 12H, CH_3_CO), 2.78 (s, 3H, CH_3_), 3.49 (ddd, 1H, *J* = 0.8 Hz, *J* = 5.7 Hz, *J* = 7.1 Hz, H-5_Gal_), 3.83 (dd, 1H, *J* = 5.7 Hz, *J* = 11.5 Hz, H-6a_Gal_), 3.93 (dd, 1H, *J* = 7.1 Hz, *J* = 11.5 Hz, H-6b_Gal_), 4.38 (d, 1H, *J* = 10.0 Hz, H-1_Gal_), 4.93 (dd, 1H, *J* = 3.4 Hz, *J* = 10.0 Hz, H-3_Gal_), 5.19 (dd~t, 1H, *J* = 10.0 Hz, *J* = 10.0 Hz, H-2_Gal_), 5.28 (dd, 1H, *J* = 0.8 Hz, *J* = 3.4 Hz, H-4_Gal_), 5.30 and 5.70 (qAB, 2H, *J* = 14.5 Hz, CH_2_N_3_), 5.61 (dd, 2H, *J* = 2.5 Hz, *J* = 16.5 Hz, CH_2_O), 7.20 (dd, 1H, *J* = 1.4 Hz, *J* = 7.4 Hz, H-7_Quin_), 7.31 (d, 1H, *J* = 8.4 Hz, H-3_Quin_), 7.34–7.43 (m, 2H, H-5_Quin_, H-6_Quin_), 7.91 (s, 1H, H-5_Triaz_), 8.02 (d, 1H, *J* = 8.4 Hz, H-4_Quin_); ^13^C NMR (100 MHz, CDCl_3_): δ 20.52, 20.60, 20.63, 20.69 (CH_3_CO), 25.71 (CH_3_) 47.77 (CH_2_N_3_), 61.49, 63.13 (C-6_Gal_, CH_2_O), 67.03, 67.16, 71.51, 74.85 (C-2_Gal_, C-3_Gal_, C-4_Gal_, C-5_Gal_), 81.51 (C-1_Gal_), 110.47 (C-7_Quin_), 120.38 (C-5_Quin_), 122.70 (C-3_Quin_), 123.20 (C-5_Triaz_), 125.62 (C-6_Quin_), 127.77 (C-4a_Quin_), 136.15 (C-4_Quin_), 139.90 (C-8a_Quin_), 145.27 (C-4_Triaz_), 153.14 (C-2_Quin_), 158.37 (C-8_Quin_), 169.60, 169.80, 170.10, 170.24 (CH_3_CO); HRMS (ESI-TOF): calcd for C_28_H_33_N_4_O_10_S ([M + H]^+^): *m*/*z* 617.1917; found: *m*/*z* 617.1917.

*Glycoconjugate***47**: *Starting from sugar derivative***19***and 8-HQ derivative***39***, the product was obtained as a white solid (71% yield)*, m.p.: 123–126 °C; [α]^23^_D_ = −127.0 (c = 1.0, DMSO); ^1^H NMR (400 MHz, DMSO): δ 3.02–3.10 (m, 2H, H-2_Glu_, H-4_Glu_), 3.16 (m, 1H, H-5_Glu_), 3.26 (m, 1H, H-3_Glu_), 3.48 (m, 1H, H-6a_Glu_), 3.78 (m, 1H, H-6b_Glu_), 4.47 (d, 1H, *J* = 9.7 Hz, H-1_Glu_), 4.96 (t, 1H, *J* = 5.4 Hz, 6-OH), 5.02 (d, 1H, *J* = 5.5 Hz, OH), 5.10 (d, 1H, *J* = 4.9 Hz, OH), 5.29 (d, 1H, *J* = 6.2 Hz, OH), 5.36 (s, 2H, CH_2_O), 5.65 and 5.82 (qAB, 2H, *J* = 14.5 Hz, CH_2_N_3_), 7.42 (dd, 1H, *J* = 3.4 Hz, *J* = 5.4 Hz, H-7_Quin_), 7.51–7.58 (m, 3H, H-3_Quin_, H-5_Quin_, H-6_Quin_), 8.34 (dd, 1H, *J* = 1.4 Hz, *J* = 8.3 Hz, H-4_Quin_), 8.56 (s, 1H, H-5_Triaz_), 8.83 (d, 1H, *J* = 2.4 Hz, H-2_Quin_); ^13^C NMR (100 MHz, DMSO): δ 47.81 (CH_2_N_3_), 61.39, 61.97 (C-6_Glu_, CH_2_O), 70.16, 73.25, 78.05, 81.25 (C-2_Glu_, C-3_Glu_, C-4_Glu_, C-5_Glu_), 83.88 (C-1_Glu_), 110.01 (C-7_Quin_), 120.04 (C-5_Quin_), 121.88 (C-3_Quin_), 125.28 (C-5_Triaz_), 126.79 (C-6_Quin_), 129.08 (C-4a_Quin_), 135.97 (C-4_Quin_), 139.55 (C-8a_Quin_), 142.95 (C-4_Triaz_), 148.96 (C-2_Quin_), 153.74 (C-8_Quin_); HRMS (ESI-TOF): calcd for C_19_H_23_N_4_O_6_S ([M + H]^+^): *m*/*z* 435.1338; found: *m*/*z* 435.1339.

*Glycoconjugate***48***: Starting from sugar derivative***20***and 8-HQ derivative***39***, the product was obtained as a beige solid (79% yield)*, m.p.: 142–145 °C; [α]^23^_D_ = −58.7 (c = 1.0, DMSO); ^1^H NMR (400 MHz, DMSO): δ 3.29 (m, 1H, H-3_Gal_), 3.39 (m, 1H, H-5_Gal_), 3.45 (m, 1H, H-2_Gal_), 3.53 (m, 1H, H-6a_Gal_), 3.60 (m, 1H, H-6b_Gal_), 3.69 (m, 1H, H-4_Gal_), 4.40 (d, 1H, *J* = 9.6 Hz, H-1_Gal_), 4.52 (d, 1H, *J* = 4.5 Hz, OH), 4.84 (d, 1H, *J* = 5.2 Hz, OH), 4.91 (t, 1H, *J* = 5.2 Hz, 6-OH), 5.12 (d, 1H, *J* = 6.1 Hz, OH), 5.36 (s, 2H, CH_2_O), 5.63 and 5.79 (qAB, 2H, *J* = 14.5 Hz, CH_2_N_3_), 7.41 (dd, 1H, *J* = 3.6, *J* = 5.4 Hz, H-7_Quin_), 7.50–7.58 (m, 3H, H-3_Quin_, H-5_Quin_, H-6_Quin_), 8.33 (dd, 1H, *J* = 1.7 Hz, *J* = 8.3 Hz, H-4_Quin_), 8.52 (s, 1H, H-5_Triaz_), 8.83 (dd, 1H, *J* = 1.7 Hz, *J* = 4.1 Hz, H-2_Quin_); ^13^C NMR (100 MHz, DMSO): δ 47.88 (CH_2_N), 60.89, 61.98 (C-6_Gal_, CH_2_O), 68.61, 70.01, 74.55, 79.68 (C-2_Gal_, C-3_Gal_, C-4_Gal_, C-5_Gal_), 84.39 (C-1_Gal_), 110.03 (C-7_Quin_), 120.04 (C-5_Quin_), 121.88 (C-3_Quin_), 125.20 (C-5_Triaz_), 126.79 (C-6_Quin_), 129.08 (C-4a_Quin_), 135.95 (C-4_Quin_), 139.57 (C-8a_Quin_), 142.91 (C-4_Triaz_), 148.96 (C-2_Quin_), 153.72 (C-8_Quin_); HRMS (ESI-TOF): calcd for C_19_H_23_N_4_O_6_S ([M + H]^+^): *m*/*z* 435.1338; found: *m*/*z* 435.1333.

*Glycoconjugate***49***: Starting from sugar derivative***19***and 8-HQ derivative***40***, the product was obtained as a white solid (83% yield)*, m.p.: 140–144 °C; [α]^23^_D_ = −64.0 (c = 1.0, DMSO); ^1^H NMR (400 MHz, DMSO): δ 2.64 (s, 3H, CH_3_), 3.02–3.10 (m, 2H, H-2_Glu_, H-4_Glu_), 3.15 (m, 1H, H-5_Glu_), 3.24 (m, 1H, H-3_Glu_), 3.45 (m, 1H, H-6a_Glu_)_,_ 3.75 (m, 1H, H-6b_Glu_), 4.46 (d, 1H, *J* = 9.7 Hz, H-1_Glu_), 4.77 (t, 1H, *J* = 5.5 Hz, 6-OH), 5.01 (d, 1H, *J* = 5.4 Hz, OH), 5.10 (d, 1H, *J* = 4.8 Hz, OH), 5.28 (d, 1H, *J* = 6.2 Hz, OH), 5.35 (s, 2H, CH_2_O), 5.64 and 5.81 (qAB, 2H, *J* = 14.5 Hz, CH_2_N_3_), 7.37 (dd, 1H, *J* = 1.4 Hz, *J* = 7.6, H-7_Quin_), 7.42 (d, 1H, *J* = 8.3 Hz, H-3_Quin_), 7.44 (dd~t, 1H, *J* = 7.3 Hz, *J* = 7.6 Hz, H-6_Quin_), 7.47 (dd, 1H, *J* = 1.2 Hz, *J* = 8.1 Hz, H-5_Quin_), 8.19 (d, 1H, *J* = 8.4 Hz, H-4_Quin_), 8.49 (s, 1H, H-5_Triaz_); ^13^C NMR (100 MHz, DMSO): δ 24.88 (CH_3_), 47.69 (CH_2_N_3_), 61.31, 61.65 (C-6_Glu_, CH_2_O), 70.10, 73.24, 78.04, 81.27 (C-2_Glu_, C-3_Glu_, C-4_Glu_, C-5_Glu_), 83.74 (C-1_Glu_), 110.22 (C-7_Quin_), 119.85 (C-5_Quin_), 122.49 (C-3_Quin_), 125.40, 125.66 (C-5_Triaz_, C-6_Quin_), 127.35 (C-4a_Quin_), 136.03 (C-4_Quin_), 139.14 (C-8a_Quin_), 142.91 (C-4_Triaz_), 153.29 (C-2_Quin_), 157.33 (C-8_Quin_); HRMS (ESI-TOF): calcd for C_20_H_25_N_4_O_6_S ([M + H]^+^): *m*/*z* 449.1495; found: *m*/*z* 449.1494.

*Glycoconjugate***50***: Starting from sugar derivative***20***and 8-HQ derivative***40***, the product was obtained as a beige solid (92% yield)*, m.p.: 134–138 °C; [α]^23^_D_ = −90.0 (c = 1.0, DMSO); ^1^H NMR (400 MHz, DMSO): δ 2.64 (s, 3H, CH_3_), 3.27 (m, 1H, H-3_Gal_), 3.34–3.44 (m, 2H, H-2_Gal_, H-5_Gal_), 3.49 (m, 1H, H-6a_Gal_), 3.55 (m, 1H, H-6b_Gal_), 3.67 (m, 1H, H-4_Gal_), 4.37 (d, 1H, *J* = 9.6 Hz, H-1_Gal_), 4.48 (d, 1H, *J* = 4.6 Hz, OH), 4.74 (t, 1H, *J* = 5.4 Hz, 6-OH), 4.84 (d, 1H, *J* = 4.9 Hz, OH), 5.11 (d, 1H, *J* = 6.1 Hz, OH), 5.36 (s, 2H, CH_2_O), 5.62 and 5.78 (qAB, 2H, *J* = 14.5 Hz, CH_2_N_3_), 7.36 (dd, 1H, *J* = 1.7 Hz, *J* = 7.4 Hz, H-7_Quin_), 7.39–7.44 (m, 2H, H-3_Quin_, H-6_Quin_), 7.47 (dd, 1H, *J* = 1.6 Hz, *J* = 8.1 Hz, H-5_Quin_), 8.19 (d, 1H, *J* = 8.4 Hz, H-4_Quin_), 8.44 (s, 1H, H-5_Triaz_); ^13^C NMR (100 MHz, DMSO): δ 24.89 (CH_3_), 47.73 (CH_2_N_3_), 60.77, 61.65 (C-6_Gal_, CH_2_O), 68.53, 70.01, 74.54, 79.66 (C-2_Gal_, C-3_Gal_, C-4_Gal_, C-5_Gal_), 84.21 (C-1_Gal_), 110.26 (C-7_Quin_), 119.84 (C-5_Quin_), 122.49 (C-3_Quin_), 125.32, 125.66 (C-5_Triaz_, C-6_Quin_), 127.35 (C-4a_Quin_), 136.01 (C-4_Quin_), 139.17 (C-8a_Quin_), 142.87 (C-4_Triaz_), 153.25 (C-2_Quin_), 157.33 (C-8_Quin_); HRMS (ESI-TOF): calcd for C_20_H_25_N_4_O_6_S ([M + H]^+^): *m*/*z* 449.1495; found: *m*/*z* 449.1496.

*Glycoconjugate***51***: Starting from sugar derivative***17***and 8-HQ derivative***39***, the product was obtained as a brown solid (79% yield)*, m.p.: 150–152 °C; [α]^23^_D_ = −31.2 (c = 1.0, CHCl_3_); ^1^H NMR (400 MHz, CDCl_3_): δ 2.00, 2.01, 2.03, 2.03 (4s, 12H, CH_3_CO), 3.05 (m, 1H, CHS), 3.25 (m, 1H, CHS), 3.70 (ddd, 1H, *J* = 2.8 Hz, *J* = 4.6 Hz, *J* = 10.1 Hz, H-5_Glu_), 4.13 (dd, 1H, *J* = 2.8 Hz, *J* = 12.5 Hz, H-6a_Glu_), 4.19 (dd, 1H, *J* = 4.6 Hz, *J* = 12.5 Hz, H-6b_Glu_), 4.47 (d, 1H, *J* = 10.0 Hz, H-1_Glu_), 4.50–4.67 (m, 2H, CH_2_N_3_), 5.02 (dd~t, 1H, *J* = 9.4 Hz, *J* = 10.1 Hz, H-4_Glu_), 5.04 (dd~t, 1H, *J* = 9.4 Hz, *J* = 10.0 Hz, H-2_Glu_), 5.21 (dd~t, 1H, *J* = 9.4 Hz, *J* = 9.4 Hz, H-3_Glu_), 5.58 (s, 2H, CH_2_O), 7.32 (dd, 1H, *J* = 1.3 Hz, *J* = 7.2 Hz, H-7_Quin_), 7.38–7.49 (m, 3H, H-3_Quin_, H-5_Quin_, H-6_Quin_), 7.85 (s, 1H, H-5_Triaz_), 8.13 (dd, 1H, *J* = 1.4 Hz, *J* = 8.3 Hz, H-4_Quin_), 8.93 (d, 1H, *J* = 2.7 Hz, H-2_Quin_); ^13^C NMR (100 MHz, CDCl_3_): δ 20.56, 20.57, 20.63, 20.66 (CH_3_CO), 30.28 (CH_2_S) 50.61 (CH_2_N_3_), 61.85, 62.97 (C-6_Glu_, CH_2_O), 68.11, 69.50, 73.56, 76.25 (C-2_Glu_, C-3_Glu_, C-4_Glu_, C-5_Glu_), 83.73 (C-1_Glu_), 110.07 (C-7_Quin_), 120.27 (C-5_Quin_), 121.65 (C-3_Quin_), 124.05 (C-5_Triaz_), 126.74 (C-6_Quin_), 129.51 (C-4a_Quin_), 135.98 (C-4_Quin_), 140.37 (C-8a_Quin_), 144.03 (C-4_Triaz_), 149.37 (C-2_Quin_), 153.87 (C-8_Quin_), 169.35, 169.38, 170.04, 170.49 (CH_3_CO); HRMS (ESI-TOF): calcd for C_28_H_33_N_4_O_10_S ([M + H]^+^): *m*/*z* 617.1917; found: *m*/*z* 617.1918.

*Glycoconjugate***52***: Starting from sugar derivative***18***and 8-HQ derivative***39***, the product was obtained as a yellow solid (82% yield)*, m.p.: 90–95 °C; [α]^23^_D_ = −9.4 (c = 1.0, CHCl_3_); ^1^H NMR (400 MHz, CDCl_3_): δ 1.98, 1.99, 2.04, 2.15 (4s, 12H, CH_3_CO), 3.06 (m, 1H, CHS), 3.28 (m, 1H, CHS), 3.92 (ddd, 1H, *J* = 0.8 Hz, *J* = 6.0 Hz, *J* = 6.9 Hz, H-5_Gal_), 4.08 (dd, 1H, *J* = 6.0 Hz, *J* = 11.7 Hz, H-6a_Gal_), 4.12 (dd, 1H, *J* = 6.9 Hz, *J* = 11.7 Hz, H-6b_Gal_), 4.46 (d, 1H, *J* = 9.9 Hz, H-1_Gal_), 4.53–4.69 (m, 2H, CH_2_N_3_), 5.03 (dd, 1H, *J* = 3.3 Hz, *J* = 10.0 Hz, H-3_Gal_), 5.24 (dd~t, 1H, *J* = 9.9 Hz, *J* = 10.0 Hz, H-2_Gal_), 5.43 (dd, 1H, *J* = 0.8 Hz, *J* = 3.3 Hz, H-4_Gal_), 5.58 (s, 2H, CH_2_O), 7.34 (d, 1H, *J* = 7.1 Hz, H-7_Quin_), 7.38–7.49 (m, 3H, H-3_Quin_, H-5_Quin_, H-6_Quin_), 7.85 (s, 1H, H-5_Triaz_), 8.13 (d, 1H, *J* = 8.0 Hz, H-4_Quin_), 8.94 (d, 1H, *J* = 1.9 Hz, H-2_Quin_); ^13^C NMR (100 MHz, CDCl_3_): δ 20.56, 20.63, 20.68, 20.74 (CH_3_CO), 30.29 (CH_2_S) 50.66 (CH_2_N), 61.61, 62.93 (C-6_Gal_, CH_2_O), 66.76, 67.24, 71.64, 74.90 (C-2_Gal_, C-3_Gal_, C-4_Gal_, C-5_Gal_), 84.17 (C-1_Gal_), 109.99 (C-7_Quin_), 120.27 (C-5_Quin_), 121.66 (C-3_Quin_), 123.86 (C-5_Triaz_), 126.74 (C-6_Quin_), 129.51 (C-4a_Quin_), 135.96 (C-4_Quin_), 140.36 (C-8a_Quin_), 144.09 (C-4_Triaz_), 149.37 (C-2_Quin_), 153.89 (C-8_Quin_), 169.61, 169.94, 170.13, 170.33 (CH_3_CO); HRMS (ESI-TOF): calcd for C_28_H_33_N_4_O_10_S ([M + H]^+^): *m*/*z* 617.1917; found: *m*/*z* 617.1920.

*Glycoconjugate***53***: Starting from sugar derivative***17***and 8-HQ derivative***40***, the product was obtained as a beige solid (77% yield)*, m.p.: 139–142 °C; [α]^23^_D_ = −29.8 (c = 1.0, CHCl_3_); ^1^H NMR (400 MHz, CDCl_3_): δ 2.00, 2.01, 2.02, 2.03 (4s, 12H, CH_3_CO), 2.79 (s, 3H, CH_3_), 3.05 (m, 1H, CHS), 3.25 (m, 1H, CHS), 3.70 (ddd, 1H, *J* = 2.9 Hz, *J* = 4.5 Hz, *J* = 10.1 Hz, H-5_Glu_), 4.12 (dd, 1H, *J* = 2.9 Hz, *J* = 12.5 Hz, H-6a_Glu_), 4.18 (dd, 1H, *J* = 4.5 Hz, *J* = 12.5 Hz, H-6b_Glu_), 4.47 (d, 1H, *J* = 10.0 Hz, H-1_Glu_), 4.50–4.67 (m, 2H, CH_2_N_3_), 5.01 (dd~t, 1H, *J* = 9.4 Hz, *J* = 10.1 Hz, H-4_Glu_), 5.04 (dd~t, 1H, *J* = 9.4 Hz, *J* = 10.0 Hz, H-2_Glu_), 5.20 (dd~t, 1H, *J* = 9.4 Hz, *J* = 9.4 Hz, H-3_Glu_), 5.59 (s, 2H, CH_2_O), 7.26 (dd, 1H, *J* = 3.3 Hz, *J* = 5.6 Hz, H-7_Quin_), 7.31 (d, 1H, *J* = 8.4 Hz, H-3_Quin_), 7.33–7.39 (m, 2H, H5_Quin_, H-6_Quin_), 7.83 (s, 1H, H-5_Triaz_), 8.01 (d, 1H, *J* = 8.4 Hz, H-4_Quin_); ^13^C NMR (100 MHz, CDCl_3_): δ 20.56, 20.57, 20.63, 20.66 (CH_3_CO), 25.73 (CH_3_), 30.33 (CH_2_S) 50.60 (CH_2_N_3_), 61.86, 63.33 (C-6_Glu_, CH_2_O), 68.12, 69.50, 73.57, 76.24 (C-2_Glu_, C-3_Glu_, C-4_Glu_, C-5_Glu_), 83.74 (C-1_Glu_), 110.65 (C-7_Quin_), 120.26 (C-5_Quin_), 122.59 (C-3_Quin_), 123.91 (C-5_Triaz_), 125.70 (C-6_Quin_), 127.76 (C-4a_Quin_), 136.17 (C-4_Quin_), 139.93 (C-8a_Quin_), 144.37 (C-4_Triaz_), 153.37 (C-2_Quin_), 158.19 (C-8_Quin_), 169.36, 169.40, 170.04, 170.50 (CH_3_CO); HRMS (ESI-TOF): calcd for C_29_H_35_N_4_O_10_S ([M + H]^+^): *m*/*z* 631.2074; found: *m*/*z* 631.2075.

*Glycoconjugate***54***: Starting from sugar derivative***18***and 8-HQ derivative***40***, the product was obtained as a white solid (70% yield)*, m.p.: 56–61 °C; [α]^23^_D_ = −9.3 (c = 1.0, CHCl_3_); ^1^H NMR (400 MHz, CDCl_3_): δ 1.98, 1.99, 2.04, 2.15 (4s, 12H, CH_3_CO), 2.79 (s, 3H, CH_3_), 3.06 (m, 1H, CHS), 3.28 (m, 1H, CHS), 3.90 (ddd, 1H, *J* = 0.7 Hz, *J* = 6.0 Hz, *J* = 7.0 Hz, H-5_Gal_), 4.07 (dd, 1H, *J* = 6.0 Hz, *J* = 11.9 Hz, H-6a_Gal_), 4.11 (dd, 1H, *J* = 7.0 Hz, *J* = 11.9 Hz, H-6b_Gal_), 4.45 (d, 1H, *J* = 9.9 Hz, H-1_Gal_), 4.53–4.69 (m, 2H, CH_2_N_3_), 5.02 (dd, 1H, *J* = 3.3 Hz, *J* = 10.0 Hz, H-3_Gal_), 5.24 (dd~t, 1H, *J* = 9.9 Hz, *J* = 10.0 Hz, H-2_Gal_), 5.42 (dd, 1H, *J* = 0.7 Hz, *J* = 3.3 Hz, H-4_Gal_), 5.60 (s, 2H, CH_2_O), 7.28 (m, 1H, H-7_Quin_), 7.31 (d, 1H, *J* = 8.4 Hz, H-3_Quin_), 7.33–7.39 (m, 2H, H-5_Quin_, H-6_Quin_), 7.83 (s, 1H, H-5_Triaz_), 8.01 (d, 1H, *J* = 8.4 Hz, H-4_Quin_); ^13^C NMR (100 MHz, CDCl_3_): δ 20.56, 20.63, 20.67, 20.74 (CH_3_CO), 25.79 (CH_3_), 30.30 (CH_2_S) 50.65 (CH_2_N), 61.62, 63.33 (C-6_Gal_, CH_2_O), 66.75, 67.24, 71.64, 74.89 (C-2_Gal_, C-3_Gal_, C-4_Gal_, C-5_Gal_), 84.15 (C-1_Gal_), 110.59 (C-7_Quin_), 120.25 (C-5_Quin_), 122.58 (C-3_Quin_), 123.69 (C-5_Triaz_), 125.70 (C-6_Quin_), 127.75 (C-4a_Quin_), 136.17 (C-4_Quin_), 139.90 (C-8a_Quin_), 144.44 (C-4_Triaz_), 153.36 (C-2_Quin_), 158.20 (C-8_Quin_), 169.61, 169.93, 170.12, 170.33 (CH_3_CO); HRMS (ESI-TOF): calcd for C_29_H_35_N_4_O_10_S ([M + H]^+^): *m*/*z* 631.2074; found: *m*/*z* 631.2070.

*Glycoconjugate***55***: Starting from sugar derivative***21***and 8-HQ derivative***39***, the product was obtained as a brown solid (99% yield)*, m.p.: 65–69 °C; [α]^23^_D_ = −4.0 (c = 1.0, CH_3_OH); ^1^H NMR (400 MHz, DMSO): δ 2.97–3.24 (m, 6H, H-2_Glu_, H-3_Glu_, H-4_Glu_ H-5_Glu_, CH_2_S), 3.45 (m, 1H, 6a_Glu_), 3.72 (m, 1H, 6b_Glu_), 4.37 (d, 1H, *J* = 9.6 Hz, H-1_Glu_), 4.57–4.73 (m, 3H, CH_2_N_3_, 6-OH), 4.95 (d, 1H, *J* = 5.3 Hz, OH), 5.03 (d, 1H, *J* = 4.8 Hz, OH), 5.21 (d, 1H, *J* = 5.8 Hz, OH), 5.35 (s, 2H, CH_2_O), 7.41 (dd, 1H, *J* = 3.8 Hz, *J* = 5.3 Hz, H-7_Quin_), 7.50–7.57 (m, 3H, H-3_Quin_, H-5_Quin_, H-6_Quin_), 8.32 (dd, 1H, *J* = 1.8 Hz, *J* = 8.3 Hz, H-4_Quin_), 8.34 (s, 1H, H-5_Triaz_), 8.83 (dd, 1H, *J* = 1.8 Hz, *J* = 4.1 Hz, H-2_Quin_); ^13^C NMR (100 MHz, DMSO): δ 29.78 (CH_2_S), 49.91 (CH_2_N_3_), 61.28, 61.91 (C-6_Glu_, CH_2_O), 70.06, 72.95, 78.10, 81.00 (C-2_Glu_, C-3_Glu_, C-4_Glu_, C-5_Glu_), 85.36 (C-1_Glu_), 110.06 (C-7_Quin_), 120.01 (C-5_Quin_), 121.84 (C-3_Quin_), 125.39 (C-5_Triaz_), 126.75 (C-6_Quin_), 129.06 (C-4a_Quin_), 135.81 (C-4_Quin_), 139.70 (C-8a_Quin_), 142.21 (C-4_Triaz_), 148.95 (C-2_Quin_), 153.86 (C-8_Quin_); HRMS (ESI-TOF): calcd for C_20_H_25_N_4_O_6_S ([M + H]^+^): *m*/*z* 449.1495; found: *m*/*z* 449.1492.

*Glycoconjugate***56***: Starting from sugar derivative***22***and 8-HQ derivative***39***, the product was obtained as a beige solid (77% yield)*, m.p.: 125–128 °C; [α]^23^_D_ = 6.2 (c = 1.0, CH_3_OH); ^1^H NMR (400 MHz, DMSO): δ 3.06 (m, 1H, CHS), 3.19 (m, 1H, CHS), 3.28 (m, 1H, H-3_Gal_), 3.36–3.46 (m, 2H, 2_Gal_, 5_Gal_), 3.47–3.58 (m, 2H, 6a_Gal_, 6b_Gal_), 3.69 (m, 1H, H-4_Gal_), 4.30 (d, 1H, *J* = 9.4 Hz Hz, H-1_Gal_), 4.45 (d, 1H, *J* = 4.5 Hz, OH), 4.58–4.73 (m, 3H, CH_2_N_3_, 6-OH), 4.82 (d, 1H, *J* = 5.4 Hz, OH), 5.06 (d, 1H, *J* = 5.7 Hz, OH), 5.35 (s, 2H, CH_2_O), 7.42 (dd, 1H, *J* = 4.2 Hz, *J* = 4.9 Hz, H-7_Quin_), 7.50–7.57 (m, 3H, H-3_Quin_, H-5_Quin_, H-6_Quin_), 8.32 (dd, 1H, *J* = 1.8 Hz, *J* = 8.3 Hz, H-4_Quin_), 8.34 (s, 1H, H-5_Triaz_), 8.83 (dd, 1H, *J* = 1.8 Hz, *J* = 4.1 Hz, H-2_Quin_); ^13^C NMR (100 MHz, DMSO): δ 29.66 (CH_2_S), 50.04 (CH_2_N_3_), 60.82, 61.91 (C-6_Gal_, CH_2_O), 68.57, 69.65, 74.64, 79.36 (C-2_Gal_, C-3_Gal_, C-4_Gal_, C-5_Gal_), 85.80 (C-1_Gal_), 110.07 (C-7_Quin_), 120.06 (C-5_Quin_), 121.92 (C-3_Quin_), 125.37 (C-5_Triaz_), 126.83 (C-6_Quin_), 129.11 (C-4a_Quin_), 135.89 (C-4_Quin_), 139.71 (C-8a_Quin_), 142.31 (C-4_Triaz_), 149.03 (C-2_Quin_), 153.88 (C-8_Quin_); HRMS (ESI-TOF): calcd for C_20_H_25_N_4_O_6_S ([M + H]^+^): *m*/*z* 449.1495; found: *m*/*z* 449.1494.

*Glycoconjugate***57***: Starting from sugar derivative***21***and 8-HQ derivative***40***, the product was obtained as a brown solid (87% yield)*, m.p.: 91–95 °C; [α]^23^_D_ = 5.0 (c = 1.0, CH_3_OH); ^1^H NMR (400 MHz, DMSO): δ 2.63 (s, 3H, CH_3_), 2.98–3.23 (m, 6H, H-2_Glu_, H-3_Glu_, H-4_Glu_ H-5_Glu_, CH_2_S), 3.44 (m, 1H, 6a_Glu_), 3.72 (m, 1H, 6b_Glu_), 4.38 (d, 1H, *J* = 9.6 Hz, H-1_Glu_), 4.55–4.73 (m, 3H, CH_2_N_3_, 6-OH), 4.95 (d, 1H, *J* = 5.3 Hz, OH), 5.03 (d, 1H, *J* = 4.8 Hz, OH), 5.20 (d, 1H, *J* = 5.8 Hz, OH), 5.35 (s, 2H, CH_2_O), 7.37 (dd, 1H, *J* = 1.7 Hz, *J* = 8.1 Hz, H-7_Quin_), 7.39–7.44 (m, 2H, H-3_Quin_, H-6_Quin_), 7.47 (dd, 1H, *J* = 1.7 Hz, *J* = 7.4 Hz, H-5_Quin_), 8.19 (d, 1H, *J* = 8.4 Hz, H-4_Quin_), 8.33 (s, 1H, H-5_Triaz_); ^13^C NMR (100 MHz, DMSO): δ 24.83 (CH_3_), 29.70 (CH_2_S), 49.82 (CH_2_N), 61.19, 61.68 (C-6_Glu_, CH_2_O), 69.96, 72.85, 78.01, 80.91 (C-2_Glu_, C-3_Glu_, C-4_Glu_, C-5_Glu_), 85.30 (C-1_Glu_), 110.21 (C-7_Quin_), 119.74 (C-5_Quin_), 122.36 (C-3_Quin_), 125.34 (C-5_Triaz_), 125.57 (C-6_Quin_), 127.24 (C-4a_Quin_), 135.88 (C-4_Quin_), 139.10 (C-8a_Quin_), 142.21 (C-4_Triaz_), 153.28 (C-2_Quin_), 157.16 (C-8_Quin_); HRMS (ESI-TOF): calcd for C_21_H_27_N_4_O_6_S ([M + H]^+^): *m*/*z* 463.1651; found: *m*/*z* 463.1651.

*Glycoconjugate***58***: Starting from sugar derivative***22***and 8-HQ derivative***40***, the product was obtained as a brown solid (88% yield)*, m.p.: 83–87 °C; [α]^23^_D_ = 10.2 (c = 1.0, CH_3_OH); ^1^H NMR (400 MHz, DMSO): δ 2.63 (s, 3H, CH_3_), 3.05 (m, 1H, CHS), 3.18 (m, 1H, CHS), 3.28 (m, 1H, H-3_Gal_), 3.36–3.46 (m, 2H, H-2_Gal_, H-5_Gal_), 3.48–3.58 (m, 2H, 6a_Gal_, 6b_Gal_), 3.68 (m, 1H, H-4_Gal_), 4.30 (d, 1H, *J* = 9.5 Hz, H-1_Gal_), 4.47 (d, 1H, *J* = 4.6 Hz, OH), 4.57–4.72 (m, 3H, CH_2_N_3_, 6-OH), 4.86 (d, 1H, *J* = 5.6 Hz, OH), 5.09 (d, 1H, *J* = 5.8 Hz, OH), 5.34 (s, 2H, CH_2_O), 7.37 (dd, 1H, *J* = 1.8 Hz, *J* = 7.4 Hz, H-7_Quin_), 7.40–7.46 (m, 2H, H-3_Quin_, H-6_Quin_), 7.47 (dd, 1H, *J* = 1.8 Hz, *J* = 8.1 Hz, H-5_Quin_), 8.19 (d, 1H, *J* = 8.4 Hz, H-4_Quin_), 8.33 (s, 1H, H-5_Triaz_); ^13^C NMR (100 MHz, DMSO): δ 24.97 (CH_3_), 29.67 (CH_2_S), 50.01 (CH_2_N_3_), 60.77, 61.71 (C-6_Gal_, CH_2_O), 68.52, 69.60, 74.63, 79.36 (C-2_Gal_, C-3_Gal_, C-4_Gal_, C-5_Gal_), 85.84 (C-1_Gal_), 110.23 (C-7_Quin_), 119.85 (C-5_Quin_), 122.52 (C-3_Quin_), 125.43, 125.72 (C-5_Triaz_, C-6_Quin_), 127.36 (C-4a_Quin_), 136.03 (C-4_Quin_), 139.19 (C-8a_Quin_), 142.34 (C-4_Triaz_), 153.39 (C-2_Quin_), 157.32 (C-8_Quin_); HRMS (ESI-TOF): calcd for C_21_H_27_N_4_O_6_S ([M + H]^+^): *m*/*z* 463.1651; found: *m*/*z* 463.1654.

*Glycoconjugate***59***: Starting from sugar derivative***23***and 8-HQ derivative***41***, the product was obtained as a brown oil (94% yield)*, [α]^23^_D_ = 36.8 (c = 1.0, CHCl_3_); ^1^H NMR (400 MHz, CDCl_3_): δ 1.98, 2.00, 2.02, 2.06 (4s, 12H, CH_3_CO), 2.63 (p, 2H, *J* = 6.3 Hz, CH_2_), 3.67 (ddd, 1H, *J* = 2.2 Hz, *J* = 4.6 Hz, *J* = 10.0 Hz, H-5_Glu_), 3.85 and 4.05 (qAB, 2H, *J* = 14.3 Hz, CH_2_S), 4.07 (dd, 1H, *J* = 2.2 Hz, *J* = 12.4 Hz, H-6a_Glu_), 4.20 (dd, 1H, *J* = 4.6 Hz, *J* = 12.4 Hz, H-6b_Glu_), 4.24 (t, 2H, *J* = 5.9 Hz, CH_2_O), 4.60 (d, 1H, *J* = 10.1 Hz, H-1_Glu_), 4.73 (t, 2H, *J* = 6.7 Hz, CH_2_N), 5.02 (dd~t, 1H, *J* = 9.3 Hz, *J* = 10.1 Hz, H-2_Glu_), 5.08 (dd~t, 1H, *J* = 9.4 Hz, *J* = 10.0 Hz, H-4_Glu_), 5.19 (dd~t, 1H, *J* = 9.3 Hz, *J* = 9.4 Hz, H-3_Glu_), 7.05 (dd, 1H, *J* = 2.1 Hz, *J* = 6.7 Hz, H-7_Quin_), 7.39–7.52 (m, 3H, H-3_Quin_, H-5_Quin_, H-6_Quin_), 7.67 (s, 1H, H-5_Triaz_), 8.17 (dd, 1H, *J* = 1.6 Hz, *J* = 8.3 Hz, H-4_Quin_), 8.96 (dd, 1H, *J* = 1.6 Hz, *J* = 4.0 Hz, H-2_Quin_); ^13^C NMR (100 MHz, CDCl_3_): δ 20.60, 20.62, 20.66, 20.77 (CH_3_CO), 24.39 (CH_2_), 29.61 (CH_2_S) 47.28 (CH_2_N), 61.85 (C-6_Glu_), 65.19 (CH_2_O), 68.15, 69.89, 73.82, 75.76 (C-2_Glu_, C-3_Glu_, C-4_Glu_, C-5_Glu_), 82.75 (C-1_Glu_), 109.40 (C-7_Quin_), 120.34 (C-5_Quin_), 121.74 (C-3_Quin_), 123.03 (C-5_Triaz_), 126.71 (C-6_Quin_), 129.56 (C-4a_Quin_), 136.08 (C-4_Quin_), 140.30 (C-8a_Quin_), 144.53 (C-4_Triaz_), 149.41 (C-2_Quin_), 154.20 (C-8_Quin_), 169.40, 169.81, 170.14, 170.65 (CH_3_CO); HRMS (ESI-TOF): calcd for C_29_H_35_N_4_O_10_S ([M + H]^+^): *m*/*z* 631.2074; found: *m*/*z* 631.2070.

*Glycoconjugate***60***: Starting from sugar derivative***24***and 8-HQ derivative***41***, the product was obtained as a beige solid (79% yield)*, m.p.: 56–58 °C; [α]^25^_D_ = −27.0 (c = 1.0, CHCl_3_); ^1^H NMR (400 MHz, CDCl_3_): δ 1.97, 1.99, 2.02, 2.14 (4s, 12H, CH_3_CO), 2.63 (p, 2H, *J* = 6.3 Hz, CH_2_), 3.80 (m, 1H, H-5_Gal_), 3.88 and 4.07 (qAB, 2H, *J* = 14.2 Hz, CH_2_S), 3.96–4.07 (m, 2H, CH_2_O), 4.16–4.31 (m, 2H, H-6a_Gal_, H-6b_Gal_), 4.55 (d, 1H, *J* = 10.0 Hz, H-1_Gal_), 4.73 (t, 2H, *J* = 6.7 Hz, CH_2_N), 5.00 (dd~t, 1H, *J* = 3.4 Hz, *J* = 10.0 Hz, H-3_Gal_), 5.22 (dd~t, 1H, *J* = 10.0 Hz, *J* = 10.0 Hz, H-2_Gal_), 5.38 (d, 1H, *J* = 2.9 Hz, H-4_Gal_), 7.06 (dd, 1H, *J* = 1.6 Hz, *J* = 7.0 Hz, H-7_Quin_), 7.40–7.51 (m, 3H, H-3_Quin_, H-5_Quin_, H-6_Quin_), 7.66 (s, 1H, H-5_Triaz_), 8.16 (dd, 1H, *J* = 1.5 Hz, *J* = 8.3 Hz, H-4_Quin_), 8.96 (dd, 1H, *J* = 1.4 Hz, *J* = 4.0 Hz, H-2_Quin_); ^13^C NMR (100 MHz, CDCl_3_): δ 20.58, 20.67, 20.69, 20.74 (CH_3_CO), 24.24 (CH_2_), 29.65 (CH_2_S) 47.25 (CH_2_N), 61.42 (C-6_Gal_), 65.19 (CH_2_O), 67.30, 71.78, 74.45, 77.23 (C-2_Gal_, C-3_Gal_, C-4_Gal_, C-5_Gal_), 83.04 (C-1_Gal_), 109.44 (C-7_Quin_), 120.38 (C-5_Quin_), 121.77 (C-3_Quin_), 123.04 (C-5_Triaz_), 126.74 (C-6_Quin_), 129.59 (C-4a_Quin_), 136.06 (C-4_Quin_), 140.35 (C-8a_Quin_), 144.38 (C-4_Triaz_), 149.43 (C-2_Quin_), 154.26 (C-8_Quin_), 169.58, 169.97, 170.21, 170.31 (CH_3_CO); HRMS (ESI-TOF): calcd for C_29_H_35_N_4_O_10_S ([M + H]^+^): *m*/*z* 631.2074; found: *m*/*z* 631.2076.

*Glycoconjugate***61***: Starting from sugar derivative***23***and 8-HQ derivative***42***, the product was obtained as a brown solid (77% yield)*, m.p.: 48–49 °C; [α]^23^_D_ = 36.0 (c = 1.0, CHCl_3_); ^1^H NMR (400 MHz, CDCl_3_): δ 1.97, 2.00, 2.02, 2.06 (4s, 12H, CH_3_CO), 2.56 (p, 2H, *J* = 6.3 Hz, CH_2_), 2.79 (s, 3H, CH_3_), 3.65 (ddd, 1H, *J* = 2.1 Hz, *J* = 4.5 Hz, *J* = 10.0 Hz, H-5_Glu_), 3.85 and 4.05 (qAB, 2H, *J* = 14.3 Hz, CH_2_S), 4.06 (dd, 1H, *J* = 2.1 Hz, *J* = 12.4 Hz, H-6a_Glu_), 4.19 (dd, 1H, *J* = 4.5 Hz, *J* = 12.4 Hz, H-6b_Glu_), 4.24 (t, 2H, *J* = 5.4 Hz, CH_2_O), 4.60 (d, 1H, *J* = 10.1 Hz, H-1_Glu_), 4.74 (t, 2H, *J* = 6.7 Hz, CH_2_N), 5.01 (dd~t, 1H, *J* = 9.3 Hz, *J* = 10.1 Hz, H-2_Glu_), 5.08 (dd~t, 1H, *J* = 9.4 Hz, *J* = 10.0 Hz, H-4_Glu_), 5.18 (dd~t, 1H, *J* = 9.3 Hz, *J* = 9.4 Hz, H-3_Glu_), 7.04 (dd, 1H, *J* = 2.8 Hz, *J* = 6.1 Hz, H-7_Quin_), 7.30–7.43 (m, 3H, H-3_Quin_, H-5_Quin_, H-6_Quin_), 7.74 (s, 1H, H-5_Triaz_), 8.04 (d, 1H, *J* = 8.4 Hz, H-4_Quin_); ^13^C NMR (100 MHz, CDCl_3_): δ 20.60, 20.61, 20.65, 20.77 (CH_3_CO), 24.42 (CH_2_), 25.75 (CH_3_), 29.63 (CH_2_S) 47.20 (CH_2_N), 61.84 (C-6_Glu_), 65.34 (CH_2_O), 68.14, 69.91, 73.83, 75.72 (C-2_Glu_, C-3_Glu_, C-4_Glu_, C-5_Glu_), 82.74 (C-1_Glu_), 110.06 (C-7_Quin_), 120.32 (C-5_Quin_), 122.63 (C-3_Quin_), 123.08 (C-5_Triaz_), 125.70 (C-6_Quin_), 127.76 (C-4a_Quin_), 136.19 (C-4_Quin_), 139.91 (C-8a_Quin_), 144.47 (C-4_Triaz_), 153.64 (C-2_Quin_), 158.23 (C-8_Quin_), 169.40, 169.77, 170.14, 170.65 (CH_3_CO); HRMS (ESI-TOF): calcd for C_30_H_37_N_4_O_10_S ([M + H]^+^): *m*/*z* 645.2230; found: *m*/*z* 645.2233.

*Glycoconjugate***62***: Starting from sugar derivative***24***and 8-HQ derivative***42***, the product was obtained as a beige solid (80% yield)*, m.p.: 56–58 °C; [α]^24^_D_ = −26.0 (c = 1.0, CHCl_3_); ^1^H NMR (400 MHz, CDCl_3_): δ 1.97, 1.98, 2.02, 2.14 (4s, 12H, CH_3_CO), 2.60 (p, 2H, *J* = 6.3 Hz, CH_2_), 2.79 (s, 3H, CH_3_), 3.78 (m, 1H, H-5_Gal_), 3.88 and 4.07 (qAB, 2H, *J* = 14.1 Hz, CH_2_S), 3.96–4.10 (m, 2H, CH_2_O), 4.15–4.31 (m, 2H, H-6a_Gal_, H-6b_Gal_), 4.54 (d, 1H, *J* = 10.0 Hz, H-1_Gal_), 4.75 (t, 2H, *J* = 6.7 Hz, CH_2_N), 5.00 (dd~t, 1H, *J* = 3.2 Hz, *J* = 10.0 Hz, H-3_Gal_), 5.22 (dd~t, 1H, *J* = 10.0 Hz, *J* = 10.0 Hz, H-2_Gal_), 5.37 (d, 1H, *J* = 3.2 Hz, H-4_Gal_), 7.04 (m, 1H, H-7_Quin_), 7.33 (d, 1H, *J* = 8.4 Hz, H-3_Quin_), 7.36–7.43 (m, 2H, H-5_Quin_, H-6_Quin_), 7.72 (s, 1H, H-5_Triaz_), 8.04 (d, 1H, *J* = 8.4 Hz, H-4_Quin_); ^13^C NMR (100 MHz, CDCl_3_): δ 20.58, 20.62, 20.68, 20.74 (CH_3_CO), 24.26 (CH_2_), 25.70 (CH_3_), 29.67 (CH_2_S) 47.18 (CH_2_N), 61.41 (C-6_Gal_), 65.32 (CH_2_O), 67.30, 67.32, 71.78, 74.42 (C-2_Gal_, C-3_Gal_, C-4_Gal_, C-5_Gal_), 83.02 (C-1_Gal_), 110.07 (C-7_Quin_), 120.34 (C-5_Quin_), 122.65 (C-3_Quin_), 123.07 (C-5_Triaz_), 125.73 (C-6_Quin_), 127.80 (C-4a_Quin_), 136.20 (C-4_Quin_), 139.93 (C-8a_Quin_), 144.31 (C-4_Triaz_), 153.70 (C-2_Quin_), 158.25 (C-8_Quin_), 169.58, 169.97, 170.22, 170.30 (CH_3_CO); HRMS (ESI-TOF): calcd for C_30_H_37_N_4_O_10_S ([M + H]^+^): *m*/*z* 645.2230; found: *m*/*z* 645.2233.

*Glycoconjugate***63***: Starting from sugar derivative***25***and 8-HQ derivative***41***, the product was obtained as a beige solid (81% yield)*, m.p.: 59–62 °C; [α]^25^_D_ = −51.0 (c = 1.0, CH_3_OH); ^1^H NMR (400 MHz, DMSO): δ 2.41 (p, 2H, *J* = 6.5 Hz, CH_2_), 3.02–3.10 (m, 2H, H-3_Glu_, H-5_Glu_), 3.11–3.19 (m, 2H, H-2_Glu_, H-6a_Glu_), 3.45 (m, 1H, H-6b_Glu_), 3.73 (m, 1H, H-4_Glu_), 3.85 and 3.98 (qAB, 2H, *J* = 14.2, CH_2_S), 4.19 (t, 2H, *J* = 6.1 Hz CH_2_O), 4.27 (d, 1H, *J* = 9.6 Hz, H-1_Glu_), 4.61 (t, 2H, *J* = 7.0 Hz, CH_2_N), 4.75 (t, 1H, *J* = 5.7 Hz, 6-OH), 4.97 (d, 1H, *J* = 5.3 Hz, OH), 5.00 (d, 1H, *J* = 4.9 Hz, OH), 5.17 (d, 1H, *J* = 5.9 Hz, OH), 7.20 (dd, 1H, *J* = 1.8 Hz, *J* = 7.1 Hz, H-7_Quin_), 7.48–7.54 (m, 2H, H-5_Quin_, H-6_Quin_), 7.59 (dd, 1H, *J* = 4.1 Hz, *J* = 8.3 Hz, H-3_Quin_), 8.20 (s, 1H, H-5_Triaz_), 8.33 (dd, 1H, *J* = 1.7 Hz, *J* = 8.3 Hz, H-4_Quin_), 8.90 (dd, 1H, *J* = 1.7 Hz, *J* = 4.1 Hz, H-2_Quin_); ^13^C NMR (100 MHz, DMSO): δ 23.16 (CH_2_), 29.61 (CH_2_S), 46.59 (CH_2_N), 61.35 (C-6_Glu_), 65.38 (CH_2_O), 70.14, 73.08, 78.16, 81.06 (C-2_Glu_, C-3_Glu_, C-4_Glu_, C-5_Glu_), 84.26 (C-1_Glu_), 109.80 (C-7_Quin_), 119.89 (C-5_Quin_), 121.86 (C-3_Quin_), 123.52 (C-5_Triaz_), 126.82 (C-6_Quin_), 129.05 (C-4a_Quin_), 135.85 (C-4_Quin_), 139.72 (C-8a_Quin_), 144.39 (C-4_Triaz_), 149.07 (C-2_Quin_), 154.15 (C-8_Quin_); HRMS (ESI-TOF): calcd for C_21_H_27_N_4_O_6_S ([M + H]^+^): *m*/*z* 463.1651; found: *m*/*z* 463.1653.

*Glycoconjugate***64**: *Starting from sugar derivative***26***and 8-HQ derivative***41***, the product was obtained as a white solid (89% yield)*, m.p.: 150–153 °C; [α]^26^_D_ = −30.0 (c = 1.0, DMSO); ^1^H NMR (400 MHz, DMSO): δ 2.42 (p, 2H, *J* = 6.5 Hz, CH_2_), 3.25 (m, 1H, H-3_Gal_), 3.34–3.46 (m, 2H, H-2_Gal,_ H-5_Gal_), 3.45–3.58 (m, 2H, H-6a_Gal,_ H-6b_Gal_), 3.65 (m, 1H, H-4_Gal_), 3.84 and 3.96 (qAB, 2H, *J* = 14.1 Hz, CH_2_S), 4.13–4.24 (m, 2H, CH_2_O), 4.20 (d, 1H, *J* = 9.5 Hz, H-1_Gal_), 4.42 (d, 1H, *J* = 3.8 Hz, OH), 4.61 (t, 2H, *J* = 7.0 Hz, CH_2_N), 4.72 (bs, 1H, 6-OH), 4.78 (d, 1H, *J* = 4.4 Hz, OH), 5.00 (d, 1H, *J* = 5.5 Hz, OH), 7.19 (dd, 1H, *J* = 2.2 Hz, *J* = 6.8 Hz, H-7_Quin_), 7.47–7.54 (m, 2H, H-5_Quin_, H-6_Quin_), 7.59 (dd, 1H, *J* = 4.2 Hz, *J* = 8.3 Hz, H-3_Quin_), 8.18 (s, 1H, H-5_Triaz_), 8.32 (dd, 1H, *J* = 1.7 Hz, *J* = 8.3 Hz, H-4_Quin_), 8.90 (dd, 1H, *J* = 1.7 Hz, *J* = 4.2 Hz, H-2_Quin_); ^13^C NMR (100 MHz, DMSO): δ 23.08 (CH_2_), 29.59 (CH_2_S), 46.58 (CH_2_N), 60.86 (C-6_Gal_), 65.34 (CH_2_O), 68.60, 69.84, 74.64, 79.38 (C-2_Gal_, C-3_Gal_, C-4_Gal_, C-5_Gal_), 84.70 (C-1_Gal_), 109.78 (C-7_Quin_), 119.90 (C-5_Quin_), 121.87 (C-3_Quin_), 123.53 (C-5_Triaz_), 126.82 (C-6_Quin_), 129.05 (C-4a_Quin_), 135.85 (C-4_Quin_), 139.73 (C-8a_Quin_), 144.41 (C-4_Triaz_), 149.09 (C-2_Quin_), 154.16 (C-8_Quin_); HRMS (ESI-TOF): calcd for C_21_H_27_N_4_O_6_S ([M + H]^+^): *m*/*z* 463.1651; found: *m*/*z* 463.1651.

*Glycoconjugate***65***: Starting from sugar derivative***25***and 8-HQ derivative***42***, the product was obtained as a brown solid (91% yield)*, m.p.: 55–58 °C; [α]^26^_D_ = −40.0 (c = 1.0, DMSO); ^1^H NMR (400 MHz, DMSO): δ 2.40 (p, 2H, *J* = 6.6 Hz, CH_2_), 2.67 (s, 3H, CH_3_), 2.99–3.19 (m, 4H, H-2_Glu_, H-3_Glu_, H-4_Glu_, H-5_Glu_), 3.43 (m, 1H, H-6a_Glu_), 3.71 (m, 1H, H-6b_Glu_), 3.84 and 3.98 (qAB, 2H, *J* = 14.1 Hz, CH_2_S), 4.19 (t, 2H, *J* = 6.2 Hz, CH_2_O), 4.26 (d, 1H, *J* = 9.6 Hz, H-1_Glu_), 4.61 (t, 2H, *J* = 7.0 Hz, CH_2_N), 4.69 (t, 1H, *J* = 5.8 Hz, 6-OH), 4.94 (d, 1H, *J* = 5.1 Hz, OH), 5.01 (d, 1H, *J* = 4.7 Hz, OH), 5.15 (d, 1H, *J* = 5.8 Hz, OH), 7.15 (dd, 1H, *J* = 1.3 Hz, *J* = 7.5 Hz, H-7_Quin_), 7.38–7.49 (m, 3H, H-3_Quin_, H-5_Quin_, H-6_Quin_), 8.17 (s, 1H, H-5_Triaz_), 8.19 (d, 1H, *J* = 8.4 Hz, H-4_Quin_); ^13^C NMR (100 MHz, DMSO): δ 23.10 (CH_2_), 25.07 (CH_3_), 29.54 (CH_2_S), 46.58 (CH_2_N), 61.31 (C-6_Glu_), 65.54 (CH_2_O), 70.13, 73.09, 78.16, 81.06 (C-2_Glu_, C-3_Glu_, C-4_Glu_, C-5_Glu_), 84.21 (C-1_Glu_), 110.33 (C-7_Quin_), 119.83 (C-5_Quin_), 122.47 (C-3_Quin_), 123.45 (C-5_Triaz_), 125.75 (C-6_Quin_), 127.33 (C-4a_Quin_), 136.00 (C-4_Quin_), 139.31 (C-8a_Quin_), 144.36 (C-4_Triaz_), 153.67 (C-2_Quin_), 157.32 (C-8_Quin_); HRMS (ESI-TOF): calcd for C_22_H_29_N_4_O_6_S ([M + H]^+^): *m*/*z* 477.1808; found: *m*/*z* 477.1806.

*Glycoconjugate***66**: *Starting from sugar derivative***26***and 8-HQ derivative***42***, the product was obtained as a beige solid (90% yield)*, m.p.: 83–87 °C; [α]^23^_D_ = 10.2 (c = 1.0, DMSO); ^1^H NMR (400 MHz, DMSO): δ 2.41 (p, 2H, *J* = 6.3 Hz, CH_2_), 3.43 (s, 3H, CH_3_), 3.23 (m, 1H, H-3_Gal_), 3.34–3.42 (m, 2H, H-2_Gal,_ H-5_Gal_), 3.43–3.58 (m, 2H, H-6a_Gal,_ H-6b_Gal_), 3.65 (m, 1H, H-4_Gal_), 3.83 and 3.96 (qAB, 2H, *J* = 14.1 Hz, CH_2_S), 4.12–4.24 (m, 2H, CH_2_O), 4.19 (d, 1H, *J* = 9.4 Hz, H-1_Gal_), 4.41 (d, 1H, *J* = 4.0 Hz, OH), 4.61 (t, 2H, *J* = 7.0 Hz, CH_2_N), 4.68 (bs, 1H, 6-OH), 4.77 (d, 1H, *J* = 4.7 Hz, OH), 4.99 (d, 1H, *J* = 5.6 Hz, OH), 7.16 (dd, 1H, *J* = 1.3 Hz, *J* = 7.6 Hz, H-7_Quin_), 7.38–7.45 (m, 2H, H-3_Quin_, H-6_Quin_), 7.47 (dd, 1H, *J* = 1.4 Hz, *J* = 8.2 Hz, H-5_Quin_), 8.16 (s, 1H, H-5_Triaz_), 8.19 (d, 1H, *J* = 8.4 Hz, H-4_Quin_); ^13^C NMR (100 MHz, DMSO): δ 23.02 (CH_2_), 25.08 (CH_3_), 29.52 (CH_2_S), 46.56 (CH_2_N), 60.81 (C-6_Gal_), 65.50 (CH_2_O), 68.57, 69.83, 74.64, 79.36 (C-2_Gal_, C-3_Gal_, C-4_Gal_, C-5_Gal_), 84.65 (C-1_Gal_), 110.31 (C-7_Quin_), 119.83 (C-5_Quin_), 122.47 (C-3_Quin_), 123.43 (C-5_Triaz_), 125.73 (C-6_Quin_), 127.33 (C-4a_Quin_), 135.99 (C-4_Quin_), 139.31 (C-8a_Quin_), 144.37 (C-4_Triaz_), 153.67 (C-2_Quin_), 157.33 (C-8_Quin_); HRMS (ESI-TOF): calcd for C_22_H_29_N_4_O_6_S ([M + H]^+^): *m*/*z* 477.1808; found: *m*/*z* 477.1807.

*Glycoconjugate***67***: Starting from sugar derivative***33***and 8-HQ derivative***39***, the product was obtained as a brown solid (84% yield)*, m.p.: 114–115 °C; [α]^20^_D_ = −2.2 (c = 1.0, CHCl_3_); ^1^H NMR (400 MHz, CDCl_3_): δ 1.97, 2.00, 2.00, 2.02 (4s, 12H, CH_3_CO), 3.80 (ddd, 1H, *J* = 2.2 Hz, *J* = 4.4 Hz, *J* = 10.1 Hz, H-5_Glu_), 4.07 (dd, 1H, *J* = 2.2 Hz, *J* = 12.4 Hz, H-6a_Glu_), 4.22 (dd, 1H, *J* = 4.4 Hz, *J* = 12.4 Hz, H-6b_Glu_), 5.05–5.20 (m, 4H, H-2_Glu_, H-4_Glu_, CH_2_O), 5.29 (dd~t, 1H, *J* = 9.3 Hz, *J* = 9.3 Hz, H-3_Glu_), 5.40 (s, 2H, CH_2_O), 5.59 (d, 1H, *J* = 10.4 Hz, H-1_Glu_), 6.89 (d, 1H, *J* = 8.4 Hz, H-3_Pyr_), 7.34 (d, 1H, *J* = 6.6 Hz, H-7_Quin_), 7.44–7.59 (m, 3H, H-3_Quin_, H-5_Quin_, H-6_Quin_), 7.76 (d, 1H, *J* = 8.4 Hz, H-4_Pyr_), 7.97 (s, 1H, H-5_Triaz_), 8.25 (d, 1H, *J* = 7.7 Hz, H-4_Quin_), 8.41 (s, 1H, H-6_Pyr_), 8.84 (bs, 1H, H-2_Quin_), 10.84 (bs, 1H, NH); ^13^C NMR (100 MHz, CDCl_3_): δ 20.60, 20.62, 20.71, 20.71 (CH_3_CO), 52.80 (CH_2_N), 61. 87, 62.04 (CH_2_O, C-6_Glu_), 68.20, 69.47, 74.11, 75.83 (C-2_Glu_, C-3_Glu_, C-4_Glu_, C-5_Glu_), 82.22 (C-1_Glu_), 109.56 (C-7_Quin_), 120.45 (C-5_Quin_), 122.10 (C-3_Quin_), 123.27 (C-5_Triaz_), 125.92 (C_Pyr_), 127.29 (C_Pyr_), 128.09 (C-6_Quin_), 129.73 (C-4a_Quin_), 132.80 (C-7_Quin_), 136.99 (C-4_Quin_), 139.54 (C-7_Quin_), 141.30 (C-8a_Quin_), 142.92 (C-4_Triaz_), 148.63 (C-7_Quin_), 149.61 (C-2_Quin_), 153.69 (C-8_Quin_), 163.96 (C=O), 169.44, 169.53, 170.11, 170.65 (CH_3_CO); HRMS (ESI-TOF): calcd for C_33_H_35_N_6_O_11_S ([M + H]^+^): *m*/*z* 732.2085; found: *m*/*z* 732.2084.

*Glycoconjugate***68***: Starting from sugar derivative***34***and 8-HQ derivative***39***, the product was obtained as a beige solid (83% yield)*, m.p.: 128–129 °C; [α]^21^_D_ = 9.6 (c = 1.0, CHCl_3_); ^1^H NMR (400 MHz, CDCl_3_): δ 1.94, 1.98, 2,01, 2.15 (4s, 12H, CH_3_CO), 4.01 (m, 1H, H-5_Gal_), 4.05–4.10 (m, 2H, H-6a_Gal_, H-6b_Gal_), 5.11 (s, 2H, CH_2_O), 5.14 (dd, 1H, *J* = 3.4 Hz, *J* = 10.1 Hz, H-3_Gal_), 5.36 (dd~t, 1H, *J* = 10.1 Hz, *J* = 10.2 Hz, H-2_Gal_), 5.40 (s, 2H, CH_2_N), 5.45 (dd, 1H, *J* = 0.7 Hz, *J* = 3.4 Hz, H-4_Gal_), 5.58 (d, 1H, *J* = 10.2 Hz, H-1_Gal_), 7.00 (d, 1H, *J* = 8.7 Hz, H-3_Pyr_), 7.33 (d, 1H, *J* = 7.4 Hz, H-7_Quin_), 7.44–7.58 (m, 3H, H-3_Quin_, H-5_Quin_, H-6_Quin_), 7.81 (dd, 1H, *J* = 2.3 Hz, *J* = 8.7 Hz, H-4_Pyr_), 7.97 (s, 1H, H-5_Triaz_), 8.24 (d, 1H, *J* = 8.3 Hz, H-4_Quin_), 8.43 (d, 1H, *J* = 1.9 Hz, H-6_Pyr_), 8.32 (d, 1H, *J* = 3.0 Hz, H-2_Quin_), 10.77 (bs, 1H, NH); ^13^C NMR (100 MHz, CDCl_3_): δ 20.60, 20.64, 20.68, 20.80 (CH_3_CO), 52.83 (CH_2_N), 61. 23, 62.07 (CH_2_O, C-6_Gal_), 66.84, 67.29, 72.04, 74.46 (C-2_Gal_, C-3_Gal_, C-4_Gal_, C-5_Gal_), 82.75 (C-1_Gal_), 109.57 (C-7_Quin_), 120.49 (C-5_Quin_), 122.08 (C-3_Quin_), 123.29 (C-5_Triaz_), 125.85 (C_Pyr_), 127.24 (C_Pyr_), 128.21 (C-6_Quin_), 129.73 (C-4a_Quin_), 132.76 (C-7_Quin_), 136.96 (C-4_Quin_), 139.55 (C-7_Quin_), 141.36 (C-8a_Quin_), 143.02 (C-4_Triaz_), 148.65 (C-7_Quin_), 149.93 (C-2_Quin_), 153.67 (C-8_Quin_), 163.91 (C=O), 169.72, 169.98, 170.24, 170.33 (CH_3_CO); HRMS (ESI-TOF): calcd for C_33_H_35_N_6_O_11_S ([M + H]^+^): *m*/*z* 723.2085; found: *m*/*z* 723.2086.

*Glycoconjugate***69***: Starting from sugar derivative***35***and 8-HQ derivative***39***, the product was obtained as a brown solid (77% yield)*, m.p.: 143–144 °C; [α]^22^_D_ = −24.0 (c = 0.5, H_2_O); ^1^H NMR (400 MHz, DMSO): δ 3.13 (m, 1H, H-2_Glu_), 3.21–3.29 (m, 2H, H-3_Glu_, H-4_Glu_), 3.37–3.50 (m, 2H, H-6a_Glu_, H-6b_Glu_), 3.66 (m, 1H, H-5_Glu_), 4.51 (t, 1H, *J* = 5.7 Hz, 6-OH), 5.00 (d, 1H, *J* = 5.3 Hz, OH), 5.07 (d, 1H, *J* = 9.9 Hz, H-1_Glu_),5.13 (d, 1H, *J* = 4.9 Hz, OH), 5.36 (d, 1H, *J* = 6.1 Hz, OH), 5.39 (t, 2H, *J* = 6.0 Hz, CH_2_O), 5.42 (t, 2H, *J* = 6.9 Hz, CH_2_N), 7.40 (d, 1H, *J* = 8.6 Hz, H-3_Pyr_), 7.44 (dd, 1H, *J* = 4.4 Hz, *J* = 8.9 Hz, H-7_Quin_), 7.50–7.58 (m, 3H, H-3_Quin_, H-5_Quin_, H-6_Quin_), 7.92 (dd, 1H, *J* = 2.6 Hz, *J* = 8.6 Hz, H-4_Pyr_), 8.32 (dd, 1H, *J* = 1.8 Hz, *J* = 8.3 Hz, H-4_Quin_), 8.35 (s, 1H, H-5_Triaz_), 8.63 (d, 1H, *J* = 2.6 Hz, H-6_Pyr_), 8.84 (dd, 1H, *J* = 1.7 Hz, *J* = 4.1 Hz, H-2_Quin_), 10.73 (s, 1H, NH); ^13^C NMR (100 MHz, DMSO): δ 52.10 (CH_2_N), 60. 83, 61.77 (CH_2_O, C-6_Glu_), 69.71, 72.39, 78.28, 81.05 (C-2_Glu_, C-3_Glu_, C-4_Glu_, C-5_Glu_), 84.60 (C-1_Glu_), 109.94 (C-7_Quin_), 119.98 (C-5_Quin_), 121.85 (C-3_Quin_), 122.17 (C-5_Triaz_), 126.51 (C_Pyr_), 126.74 (C_Pyr_), 127.76 (C-6_Quin_), 129.06 (C-4a_Quin_), 132.35 (C-7_Quin_), 135.77 (C-4_Quin_), 139.73 (C-7_Quin_), 140.29 (C-8a_Quin_), 142.49 (C-4_Triaz_), 148.98 (C-7_Quin_), 152.52 (C-2_Quin_), 153.87 (C-8_Quin_), 164.69 (C=O); HRMS (ESI-TOF): calcd for C_25_H_27_N_6_O_7_S ([M + H]^+^): *m*/*z* 555.1662; found: *m*/*z* 555.1667.

*Glycoconjugate***70***: Starting from sugar derivative***36***and 8-HQ derivative***41***, the product was obtained as a white solid (72% yield)*, m.p.: 93–95 °C; [α]^21^_D_ = −3.0 (c = 1, CHCl_3_); ^1^H NMR (400 MHz, DMSO): δ 1.96, 1.97, 1.98, 2.00 (4s, 12H, CH_3_CO), 2.43 (bs, 2H, CH_2_), 4.02 (m, 1H, H-5_Glu_), 4.10–4.17 (m, 2H, H-6a_Glu_, H-6b_Glu_), 4.21 (bs, 2H, CH_2_O), 4.67 (t, 2H, *J* = 6.0 Hz, CH_2_N), 4.93–5.02 (m, 2H, H-2_Glu_, H-4_Glu_), 5.22 (s, 2H, CH_2_OCO), 5.41 (dd~t, 1H, *J* = 9.4 Hz, *J* = 9.4 Hz, H-3_Glu_), 5.69 (d, 1H, *J* = 10.3 Hz, H-1_Glu_), 7.21 (d, 1H, *J* = 8.0 Hz, H-7_Quin_), 7.37 (d, 1H, *J* = 8.7 Hz, H-3_Pyr_), 7.50 (dd~t, 1H, *J* = 7.5 Hz, *J* = 8.0 Hz, H-3_Quin_), 7.47–7.64 (m, 2H, H-5_Quin_, H-6_Quin_), 7.83 (dd, 1H, *J* = 2.4 Hz, *J* = 8.7 Hz, H-4_Pyr_), 8.32–8.40 (m, 2H, H-4_Quin_, H-6_Pyr_), 8.54 (s, 1H, H-5_Triaz_), 8.91 (bs, 1H, H-2_Quin_), 10.00 (s, 1H, NH); ^13^C NMR (100 MHz, DMSO): δ 20.19, 20.24, 20.28, 20.24 (CH_3_CO), 29.51 (CH_2_), 46.63 (CH_2_N), 57.63 (CH_2_OCO), 61.73 (C-6_Glu_), 65.36 (CH_2_O), 67.94, 69.33, 72.91, 74.41 (C-2_Glu_, C-3_Glu_, C-4_Glu_, C-5_Glu_), 81.26 (C-1_Glu_), 109.82 (C-7_Quin_), 119.92 (C-5_Quin_), 121.81 (C-3_Quin_), 123.05 (C-5_Triaz_), 125.00 (C_Pyr_), 126.60 (C_Pyr_), 126.78 (C-6_Quin_), 128.98 (C-4a_Quin_), 133.84 (C_Pyr_), 135.92 (C-4_Quin_), 139.43 (C_Pyr_), 139.68 (C-8a_Quin_), 141.91 (C-4_Triaz_), 148.01 (C_Pyr_), 148.84 (C-2_Quin_), 153.10 (C-8_Quin_), 153.99 (C=O), 168.99, 169.18, 169.38, 169.80 (CH_3_CO); HRMS (ESI-TOF): calcd for C_35_H_39_N_6_O_12_S ([M + H]^+^): *m*/*z* 767.2347; found: *m*/*z* 767.2344.

*Glycoconjugate***71***: Starting from sugar derivative***37***and 8-HQ derivative***41***, the product was obtained as a white solid (73% yield)*, m.p.: 97–99 °C; [α]^21^_D_ = 9.8 (c = 1.0, CHCl_3_); ^1^H NMR (400 MHz, DMSO): δ 1.94, 1.95, 2.00, 2.14 (4s, 12H, CH_3_CO), 2.43 (bs, 2H, CH_2_), 3.97–4.07 (m, 2H, H-6a_Gal_, H-6b_Gal_), 4.21 (bs, 2H, CH_2_O), 4.35 (m, 1H, H-5_Gal_), 4.67 (t, 2H, *J* = 6.5 Hz, CH_2_N), 5.13 (dd~t, 1H, *J* = 9.9 Hz, *J* = 10.2 Hz, H-2_Gal_), 5.22 (s, 2H, CH_2_OCO), 5.30–5.38 (m, 2H, H-3_Gal_, H-4_Gal_), 5.65 (d, 1H, *J* = 10.2 Hz, H-1_Gal_), 7.21 (d, 1H, *J* = 8.4 Hz, H-7_Quin_), 7.38 (d, 1H, *J* = 8.7 Hz, H-3_Pyr_), 7.50 (dd~t, 1H, *J* = 7.5 Hz, *J* = 8.1 Hz, H-3_Quin_), 7.50–7.65 (m, 2H, H-5_Quin_, H-6_Quin_), 7.84 (dd, 1H, *J* = 2.4 Hz, *J* = 8.7 Hz, H-4_Pyr_), 8.32–8.40 (m, 2H, H-4_Quin_, H-6_Pyr_), 8.54 (s, 1H, H-5_Triaz_), 8.90 (bs, 1H, H-2_Quin_), 10.00 (s, 1H, NH); ^13^C NMR (100 MHz, DMSO): δ 20.33, 20.39, 20.41, 20.43 (CH_3_CO), 29.60 (CH_2_), 46.72 (CH_2_N), 57.72 (CH_2_OCO), 61.51 (C-6_Gal_), 65.44 (CH_2_O), 66.80, 67.55, 71.01, 73.66 (C-2_Gal_, C-3_Gal_, C-4_Gal_, C-5_Gal_), 81.77 (C-1_Gal_), 109.89 (C-7_Quin_), 120.00 (C-5_Quin_), 121.91 (C-3_Quin_), 123.04 (C-5_Triaz_), 125.08 (C_Pyr_), 126.67 (C_Pyr_), 126.85 (C-6_Quin_), 129.08 (C-4a_Quin_), 133.87 (C_Pyr_), 135.95 (C-4_Quin_), 139.36 (C_Pyr_), 139.78 (C-8a_Quin_), 142.00 (C-4_Triaz_), 148.29 (C_Pyr_), 148.90 (C-2_Quin_), 153.19 (C-8_Quin_), 154.18 (C=O), 169.31, 169.39, 169.75, 169.94 (CH_3_CO); HRMS (ESI-TOF): calcd for C_35_H_39_N_6_O_12_S ([M + H]^+^): *m*/*z* 767.2347; found: *m*/*z* 767.2345.

*Glycoconjugate***72***: Starting from sugar derivative***38***and 8-HQ derivative***41***, the product was obtained as a white solid (78% yield)*, m.p.: 152–154 °C; [α]^22^_D_ = −36.0 (c = 1.0, CH_3_OH); ^1^H NMR (400 MHz, DMSO): δ 2.43 (p, 2H, *J* = 6.3 Hz, CH_2_),3.09–3.19 (m, 2H, H-2_Glu_, H-4_Glu_), 3.21–3.29 (m, 2H, H-3_Glu_, H-5_Glu_), 3.44 (m, 1H, H-6a_Glu_), 3.66 (m, 1H, H-6b_Glu_), 4.19 (t, 2H, *J* = 6.0 Hz, CH_2_O), 4.51 (t, 1H, *J* = 5.7 Hz, 6-OH), 4.67 (t, 2H, *J* = 6.9 Hz, CH_2_N), 4.99 (d, 1H, *J* = 5.3 Hz, OH), 5.03 (d, 1H, *J* = 9.9 Hz, H-1_Glu_), 5.11 (d, 1H, *J* = 4.8 Hz, OH), 5.21 (s, 2H, CH_2_OCO), 5.34 (d, 1H, *J* = 6.1 Hz, OH), 7.19 (dd, 1H, *J* = 1.7 Hz, *J* = 7.1 Hz, H-7_Quin_), 7.36 (d, 1H, *J* = 8.7 Hz, H-3_Pyr_), 7.45–7.60 (m, 2H, H-5_Quin_, H-6_Quin_), 7.56 (dd, 1H, *J* = 4.0 Hz, *J* = 8.3 Hz, H-3_Quin_), 7.76 (dd, 1H, *J* = 2.1 Hz, *J* = 8.7 Hz, H-4_Pyr_), 8.32 (dd, 1H, *J* = 1.3 Hz, *J* = 7.0 Hz, H-4_Quin_), 8.37 (s, 1H, H-5_Triaz_), 8.49 (d, 1H, *J* = 2.1 Hz, H-6_Pyr_), 8.89 (bs, 1H, H-2_Quin_), 9.92 (bs, 1H, NH); ^13^C NMR (100 MHz, DMSO): δ 29.51 (CH_2_), 46.62 (CH_2_N), 57.56 (CH_2_OCO), 60.76 (C-6_Glu_), 65.32 (CH_2_O), 69.65, 72.30, 78.20, 80.97 (C-2_Glu_, C-3_Glu_, C-4_Glu_, C-5_Glu_), 84.71 (C-1_Glu_), 109.75 (C-7_Quin_), 119.86 (C-5_Quin_), 121.77 (C-3_Quin_), 122.19 (C-5_Triaz_), 124.99 (C_Pyr_), 126.58 (C_Pyr_), 126.68 (C-6_Quin_), 128.96 (C-4a_Quin_), 132.91 (C-7_Quin_), 135.71 (C-4_Quin_), 139.38 (C-7_Quin_), 139.68 (C-8a_Quin_), 141.96 (C-4_Triaz_), 148.95 (C-7_Quin_), 150.91 (C-2_Quin_), 153.12 (C-8_Quin_), 154.09 (C=O); HRMS (ESI-TOF): calcd for C_27_H_31_N_6_O_8_S ([M + H]^+^): *m*/*z* 599.1924; found: *m*/*z* 599.1919.

### 3.3. Biological Assays

#### 3.3.1. Cell Lines

The human colon adenocarcinoma cell line HCT-116 was obtained from American Type Culture Collection (ATCC, Manassas, VA, USA). The human cell line MCF-7 was obtained from collections at the Maria Sklodowska-Curie Memorial Cancer Center and Institute of Oncology, branch in Gliwice, Poland. The Normal Human Dermal Fibroblasts-Neonatal (NHDF-Neo) was purchased from LONZA (Cat. No. CC-2509, NHDF-Neo, Dermal Fibroblasts, Neonatal, Lonza, Poland). The culture media consisted of RPMI 1640 or DMEM+F12 medium, supplemented with 10% fetal bovine serum and 1% of standard antibiotics (penicillin and streptomycin). The culture media were purchased from EuroClone and HyClone. Fetal bovine serum (FBS) was delivered by EURx, Poland, and Antibiotic Antimycotic Solution (100×) by Sigma-Aldrich, Germany. The cells were cultured under standard conditions at 37 °C in a humidified atmosphere at 5% CO_2_.

#### 3.3.2. MTT Assay

The viability of the cells was determined using an MTT (3-[4,5–dimethylthiazol-2-yl]-2,5-diphenyltetrazolium bromide) test (Sigma-Aldrich). Stock solutions of tested compounds were prepared in DMSO and diluted with the appropriate volumes of the growth medium directly before the experiment. The cells were seeded into 96-well plates at concentration 1 × 10^4^ (HCT 116, NHDF-Neo) or 5 × 10^3^ (MCF-7) per well. The cell cultures were incubated for 24 h at 37 °C in a humidified atmosphere of 5% CO_2_. Then, the culture medium was removed, replaced with the solution of the tested compounds in medium with varying concentrations, and incubated for further 24 h or 72 h. After that, the medium was removed, and the MTT solution (50 µL, 0.5 mg/mL in PBS) was added. After 3 h of incubation, the MTT solution was removed, and the precipitated formazan was dissolved in DMSO. Finally, the absorbance at the 570 nm wavelength was measured spectrophotometrically with the plate reader. The experiment was conducted in at least three independent iterations with four technical repetitions. The IC_50_ values were calculated using CalcuSyn software (version 2.0, Biosoft, Cambridge, UK).

#### 3.3.3. Influence of Metal Ions on Cellular Proliferation

The cells were seeded into 96-well plates at concentration 1 × 10^4^ (HCT 116, NHDF-Neo) or 5 × 10^3^ (MCF-7) per well. The cell cultures were incubated for 24 h at 37 °C in a humidified atmosphere of 5% CO_2_. Then, the culture medium was removed, replaced with the solution of the tested compounds in medium with varying concentrations. Additionally, 100 µM solution of Cu(CH_3_COO)_2_ was added into wells with tested compounds. After 24 h or 72 h incubation, the MTT assay was performed.

## 4. Conclusions

Glycoconjugation of quinoline derivatives with appropriately functionalized 1-sugar derivatives was supposed to increase the selectivity of such obtained prodrugs in relation to the cancer cells, thanks to the facilitation of its transport through GLUT transporters, whose overexpression is seen in some types of cancer. The cytotoxic activity *in vitro* of the new quinoline glycoconjugates was tested against the MCF-7, HCT-116, and NHDF-Neo cell lines. In order to approximate the mechanism of glycoconjugates transport into the cell, both types of glycoconjugates: Protected in the sugar part and their unprotected counterparts were investigated. Based on the obtained results, it can be stated that only compounds with acetyl protection of hydroxyl groups in the sugar part have the ability to inhibit the proliferation of tumor cells. Thus, lipophilicity has a significant influence on the biological activity of the tested compounds, which can be explained by the facilitation of passive transport through biological membranes into the cells. Derivatives with an unprotected sugar fragment showed low activity, probably due to their high hydrophilicity and low affinity for the cell membrane, as well as the inability to bind to GLUT transporters. It is likely that the functionalization of the anomeric position in sugar and the use of the compounds thus obtained to binding with 8-HQ derivatives reduces the affinity of the sugar residue for GLUT transporters. In this situation, the improvement of the hydrolytic stability of the obtained glycoconjugates by introducing a sulfur atom into the anomeric position of the sugar did not affect their selectivity, but only in some cases (compounds **51**–**54**), increased cytotoxicity compared to their oxygen counterparts. This can be explained by the fact that they were not prematurely degraded before entering the cell, and, as a consequence of facilitated passive transport, their concentration in the cells was higher.

Among the tested compounds, the glycoconjugates **67**–**71** containing an additional heteroaromatic (5-amine-2-pyridyl) moiety in the linker structure turned out to be the most active. For these compounds, the additional experiments of antiproliferative activity in the presence of Cu^2+^ ions were carried out. It was observed that the activity of glycoconjugates increased significantly in the presence of copper compared to cells treated with alone glycoconjugates in the absence of Cu^2+^. The highest cytotoxicity of the compounds was observed against the MCF-7 cell line. This confirmed the strong sensitivity of breast cancer cells to the presence of copper ions as well as their sensitivity to compounds capable of complexing these ions, such as 8-HQ or sugar derivatives containing 2-thio-5-amino-pyridine moiety. Unfortunately, in the case of the latter, cytotoxicity to non-cancer cells was also observed. In such a case, the research on the dependence of glycoconjugates’ cytotoxic activity on their structure should be extended in the direction that allows better matching of glycoconjugates to GLUT transporters, which should improve their selectivity. According to some literature reports, the 6-OH group is the position in the sugar that is least involved in binding to the GLUT transporter. Perhaps the use of this position for binding with quinoline derivatives will prove effective and allow to increase the selectivity of the obtained glycoconjugates.

## Figures and Tables

**Table 1:** Summary of cytotoxicity of substrates used for glycoconjugation.

Compound	Activity IC_50_ [µM] ^a^		
	HCT-116 ^b^	MCF-7 ^c^	NHDF-Neo ^b^
**15**–**26**	>800	>800	-
**33**	13.39 ± 0.10	6.39 ± 0.07	11.20 ± 0.31
**34**	541.37 ± 3.73	382.69 ± 4.23	-
**35**	>800	>800	-
**36**	124.79 ± 8.98	38.33 ± 2.44	41.69 ± 3.40
**37**	456.70 ± 3.85	392.41 ± 5.29	-
**38**	>800	>800	-
8HQ	>800	0.24 ± 0.01	>800
2Me8HQ	>800	43.18 ± 1.78	346.77 ± 2.23
Doxorubicin	5.59 ± 0.14	0.67 ± 0.01	>20

^a^ Cytotoxicity was evaluated using the MTT assay; ^b^ Incubation time 24 h; ^c^ Incubation time 72 h. Data are presented as the mean ± standard deviation (*n* = 3).

**Table 2:** Summary of cytotoxicity of tested glycoconjugates **43**–**72**.

Compound	Activity IC_50_ [µM] ^a^		
	HCT-116 ^b^	MCF-7 ^c^	NHDF-Neo ^b^
**43**	106.71 ± 4.10	59.12 ± 1.46	54.62 ± 0.74
**44**	162.34 ± 1.35	85.65 ± 3.28	140.41 ± 7.25
**45**	268.54 ± 5.55	218.40 ± 6.24	-
**46**	789.98 ± 2.33	235.24 ± 6.77	-
**47**	791.73 ± 2.30	428.16 ± 8.84	-
**48**	376.07 ± 7.16	204.88 ± 5.85	-
**49**	>800	>800	-
**50**	>800	>800	-
**51**	127.05 ± 1.75	76.30 ± 1.33	105.32 ± 3.40
**52**	144.59 ± 4.84	93.97 ± 1.66	111.08 ± 1.24
**53**	338.97 ± 4.61	135.55 ± 1.58	-
**54**	519.18 ± 0.90	204.79 ± 2.53	-
**55**	394.77 ± 6.44	315.93 ± 1.66	-
**56**	431.84 ± 5.10	172.46 ± 5.43	-
**57**	>800	>800	-
**58**	>800	332.85 ± 1.39	-
**59**	172.83 ± 3.48	153.34 ± 0.25	229.12 ± 2.06
**60**	304.80 ± 1.34	212.15 ± 3.46	421.20 ± 0.14
**61**	107.68 ± 1.62	130.45 ± 0.20	260.43 ± 7.65
**62**	324.00 ± 1.61	243.65 ± 3.04	667.65 ± 6.43
**63**	>800	>800	-
**64**	>800	>800	-
**65**	>800	>800	-
**66**	>800	>800	-
**67**	146.16 ± 3.49	69.72 ± 3.50	71.81 ± 6.70
**67** + Cu^2+ d^	74.50 ± 0.16	28.94 ± 1.52	71.43 ± 1.35
**68**	130.31 ± 1.31	96.32 ± 5.99	110.60 ± 2.78
**68** + Cu^2+ d^	96.01 ± 0.59	23.77 ± 0.48	83.60 ± 3.03
**69**	210.39 ± 0.55	144.42 ± 2.68	163.81 ± 0.01
**70**	63.49 ± 2.37	67.50 ± 1.58	64.00 ± 5.34
**70** + Cu^2+ d^	73.87 ± 2.99	25.90 ± 1.76	68.43 ± 1.11
**71**	90.68 ± 0.52	68.32 ± 3.68	75.26 ± 0.38
**71** + Cu^2+ d^	82.99 ± 1.37	20.51 ± 1.55	69.31 ± 2.90
**72**	>800	739.20 ± 3.79	>800

^a^ Cytotoxicity was evaluated using the MTT assay; ^b^ Incubation time 24 h; ^c^ Incubation time 72 h; ^d^ Addition of cooper acetate at 100 µM. Data are presented as the mean ± standard deviation (*n* = 3).

## Supplementary Materials

Determination of glycoconjugates stability under the action of β-galactosidase from *Aspergillus oryzae*, and the ^1^H NMR and ^13^C NMR spectra of all obtained compounds.
