# Supplementary material for: 8-Hydroxyquinoline Glycoconjugates Containing Sulfur at the Sugar Anomeric Position—Synthesis and Preliminary Evaluation of Their Cytotoxicity
Source: Molecules. 2020 Sep 11;25(18):4174. doi: 10.3390/molecules25184174 (PMC7570910; doi:10.3390/molecules25184174)
Supplement: Supplementary file 1 [file molecules-25-04174-s001.pdf]

Supplementary Materials

8-Hydroxyquinoline Glycoconjugates Containing Sulfur at the Sugar Anomeric Position - Synthesis and Preliminary Evaluation of Their Cytotoxicity

Monika Krawczyk [1,2,*], Gabriela Pastuch-Gawołek [1,2], Agnieszka Hadasik [1], Karol Erfurt [3]

[1] Department of Organic Chemistry, Bioorganic Chemistry and Biotechnology, Silesian University of Technology, B. Krzywoustego 4, 44-100 Gliwice, Poland; gabriela.pastuch@polsl.pl (G.P.-G.), agnieszkahadasik@gmail.com (A.H.)
[2] Biotechnology Centre, Silesian University of Technology, B. Krzywoustego 8, 44-100 Gliwice, Poland
[3] Department of Chemical Organic Technology and Petrochemistry, Silesian University of Technology, B. Krzywoustego 4, 44-100 Gliwice, Poland; karol.erfurt@polsl.pl (K.E.)
* Correspondence: monika.krawczyk@polsl.pl (M.K.); Tel.: +48-32-237-1759

1. Determination of glycoconjugates stability under the action of β-galactosidase from *Aspergillus oryzae*

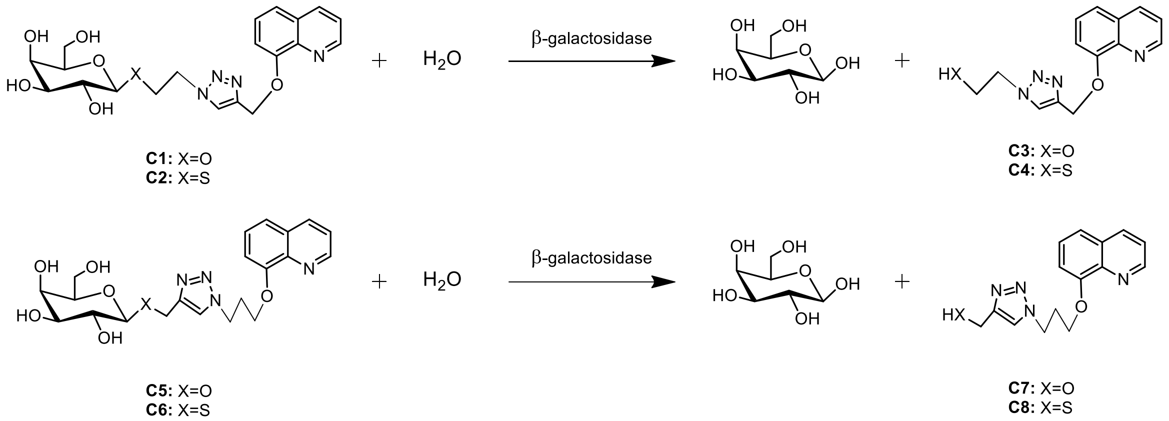

Scheme S1. Scheme of the enzymatic reactions.

1.1. Procedure for the hydrolysis reactions using β-galactosidase (EC 3.2.1.23)

β-Galactosidase from *Aspergillus oryzae* was purchased from Sigma-Aldrich. The experiment was conducted according to the procedure provided by the enzyme supplier [54].

Preparation of test solutions:

- 10 mM Citric Acid Solution (**A**) was prepared by dissolving 210 mg (1 mmol) of Citric Acid Monohydrate in 100 mL of deionized water.
- 20 mM Sodium Phosphate Solution (**B**) was prepared by dissolving 716 mg (2 mmol) of Sodium Phosphate Dibasic Dodecahydrate in 100 mL of deionized water.
- 20 mM Phosphate-Citrate Buffer (**C**) was prepared using 100 mL of solution **B** and adjusted to pH 4.5 at 30 °C with solution **A**.

- 10 mM Substrate Solutions (**D1-D4**) were prepared by dissolving 0.025 mmol of **C1**, **C2**, **C5,** or **C6** in 2.5 mL of Buffer **C.**
- β-Galactosidase Solution (**E**) was prepared by dissolving 1 mg of the enzyme in 1 mL of cold deionized water, obtained a solution (**E1**) with an activity of 8.9 unit/ml. Then 100 μl of the solution (**E1**) was diluted to a volume of 1 mL with cold deionized water, obtaining a solution (**E2**) with an activity of 0.89 unit/ml.

Procedure:

The following reagents were pipetted into a test tube: 0.4 mL of 20 mM Phosphate-Citrate Buffer (**C**) and 0.5 mL of the appropriate 10 mM Substrate Solution (**D1-D4**), which then thoroughly mixed and equilibrated to 30 °C with a thermoblock. Then 0.1 mL of β-Galactosidase Solution (**E2**) was added to the mixture, all immediately were mixed and incubated at 30 °C for 24 h. The progress of the reactions was monitored by thin-layer chromatography (TLC) in a $CHCl_3$:CH_3OH (5:1) eluents system. Analysis was performed immediately before the addition of enzyme and then after 10 min, 30 min, 60 min, 180 min, and 24 h. The TLC plates were visualized under UV light (λ = 254 nm). The resulting TLC plates are shown in Figure S1.

The 1 mL reaction mixtures contained reagents in the following final concentrations: 18 mM Phosphate-Citrate Buffer (**C**), 5 mM Substrate Solution (**D**), and 0.089 unit of β-Galactosidase.

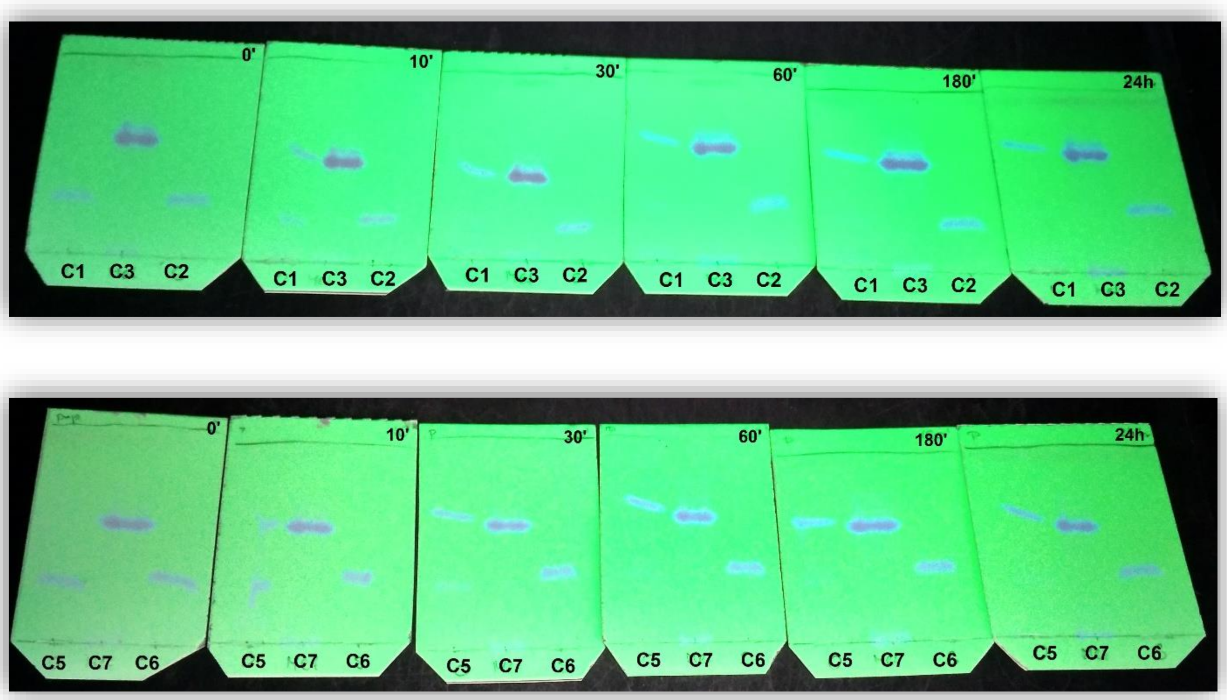

Figure S1. Thin-layer chromatography (TLC) plates.

1.2. General Procedure for the Synthesis of Metabolite ***C3***

8-(2-Propyn-1-yloxy)quinoline (68 mg, 0.37 mmol) and 2-azidoethanol (70 mg, 0.80 mmol) were dissolved in dry THF (2 mL) and *i*-PrOH (2 mL). The catalysts system were prepared: $CuSO_4{\cdot}5H_2O$ (19 mg, 0.07 mmol) dissolved in H_2O (1 mL) and sodium ascorbate (29 mg, 0.15 mmol) dissolved in H_2O (1 mL) mixed and immediately added to the solution of substrates. The reaction mixture was stirred for 24 h at room temperature. The progress of the reaction was monitored on TLC in a $CHCl_3$:CH_3OH (5:1) eluents system. After completion, the reaction mixture was concentrated in vacuo and purified using column chromatography ($CHCl_3$:MeOH, gradient: 100:1 to 20:1).

Product **C3** was obtained as a brown solid (81 mg, 81%); m.p.: 126–128 °C; $[\alpha]^{27}_{D}$ = -0.6 (c = 1.0, $CHCl_3$); ^{1}H NMR (400 MHz, $CDCl_3$): δ 4.01 (t, 2H, *J* = 5.0 Hz, CH_2), 4.45 (t, 2H, *J* = 5.0 Hz, CH_2), 5.46 (s, 2H, CH_2O_{Quin}), 7.28 (dd, 1H, *J* = 1.4 Hz, *J* = 8.2 Hz, H-7_{Quin}), 7.35-7.48 (m, 3H, H-3_{Quin}, H-5_{Quin}, H-6_{Quin}), 7.94 (s, 1H, H-5_{Triaz}), 8.12 (d, 1H, *J* = 8.3 Hz, H-4_{Quin}), 8.83 (bs, 1H, H-2_{Quin}); ^{13}C NMR (100 MHz, $CDCl_3$): δ 52.82 (CH_2N), 61.02 (CH_2O), 62.75 (CH_2O), 109.78 (C-7_{Quin}), 120.19 (C-5_{Quin}), 121.68 (C-3_{Quin}), 124.65 (C-5_{Triaz}), 126.82 (C-6_{Quin}), 129.50 (C-$4a_{Quin}$), 136.19 (C-4_{Quin}), 140.00 (C-$8a_{Quin}$), 143.72 (C-4_{Triaz}), 149.12 (C-2_{Quin}), 153.76 (C-8_{Quin}); HRMS (ESI-TOF): calcd for $C_{14}H_{15}N_4O_2$ ($[M + H]^+$): *m*/*z* 271.1195; found: *m*/*z* 271.1199.

1.3. General Procedure for the Synthesis of Metabolite ***C7***

8-(3-Azidopropoxy)quinolone (109 mg, 0.48 mmol) and propargyl alcohol (35 µL, 0.61 mmol) were dissolved in dry THF (2 mL) and *i*-PrOH (2 mL). The catalysts system were prepared: $CuSO_4{\cdot}5H_2O$ (24 mg, 0.10 mmol) dissolved in H_2O (1 mL) and sodium ascorbate (38 mg, 0.19 mmol) dissolved in H_2O (1 mL) mixed and immediately added to the solution of substrates. The reaction mixture was stirred for 24 h at room temperature. The progress of the reaction was monitored on TLC in a $CHCl_3$:CH_3OH (5:1) eluents system. After completion, the reaction mixture was concentrated in vacuo and purified using column chromatography ($CHCl_3$:MeOH, gradient: 100:1 to 20:1).

Product **C7** was obtained as a brown oil (117 mg, 86%); $[\alpha]^{25}_{D}$ = 23.0 (c = 1.0, $CHCl_3$); ^{1}H NMR (400 MHz, DMSO): δ 2.41 (p, 2H, *J* = 6.5 Hz, CH_2), 4.19 (t, 2H, *J* = 6.1 Hz, CH_2N), 4.51 (s, 2H, CH_2OH), 4.62 (t, 2H, *J* = 6.9 Hz, CH_2O), 7.19 (dd, 1H, *J* = 2.0 Hz, *J* = 7.0 Hz, H-7_{Quin}), 7.47–7.59 (m, 3H, H-3_{Quin}, H-5_{Quin}, H-6_{Quin}), 8.10 (s, 1H, H-5_{Triaz}), 8.32 (dd, 1H, *J* = 1.6 Hz, *J* = 8.2 Hz, H-4_{Quin}), 8.89 (bs, 1H, H-2_{Quin}); ^{13}C NMR (100 MHz, DMSO): δ 29.56 (CH_2), 46.37 (CH_2N), 54.97 (CH_2OH), 65.31 (CH_2O), 109.76 (C-7_{Quin}), 119.83 (C-5_{Quin}), 121.76 (C-3_{Quin}), 122.76 (C-5_{Triaz}), 126.70 (C-6_{Quin}), 128.97 (C-$4a_{Quin}$), 135.71 (C-4_{Quin}), 139.70 (C-$8a_{Quin}$), 147.96 (C-4_{Triaz}), 148.95 (C-2_{Quin}), 154.13 (C-8_{Quin}); HRMS (ESI-TOF): calcd for $C_{15}H_{17}N_4O_2$ ($[M + H]^+$): *m*/*z* 285.1352; found: *m*/*z* 285.1354.

2. Spectra

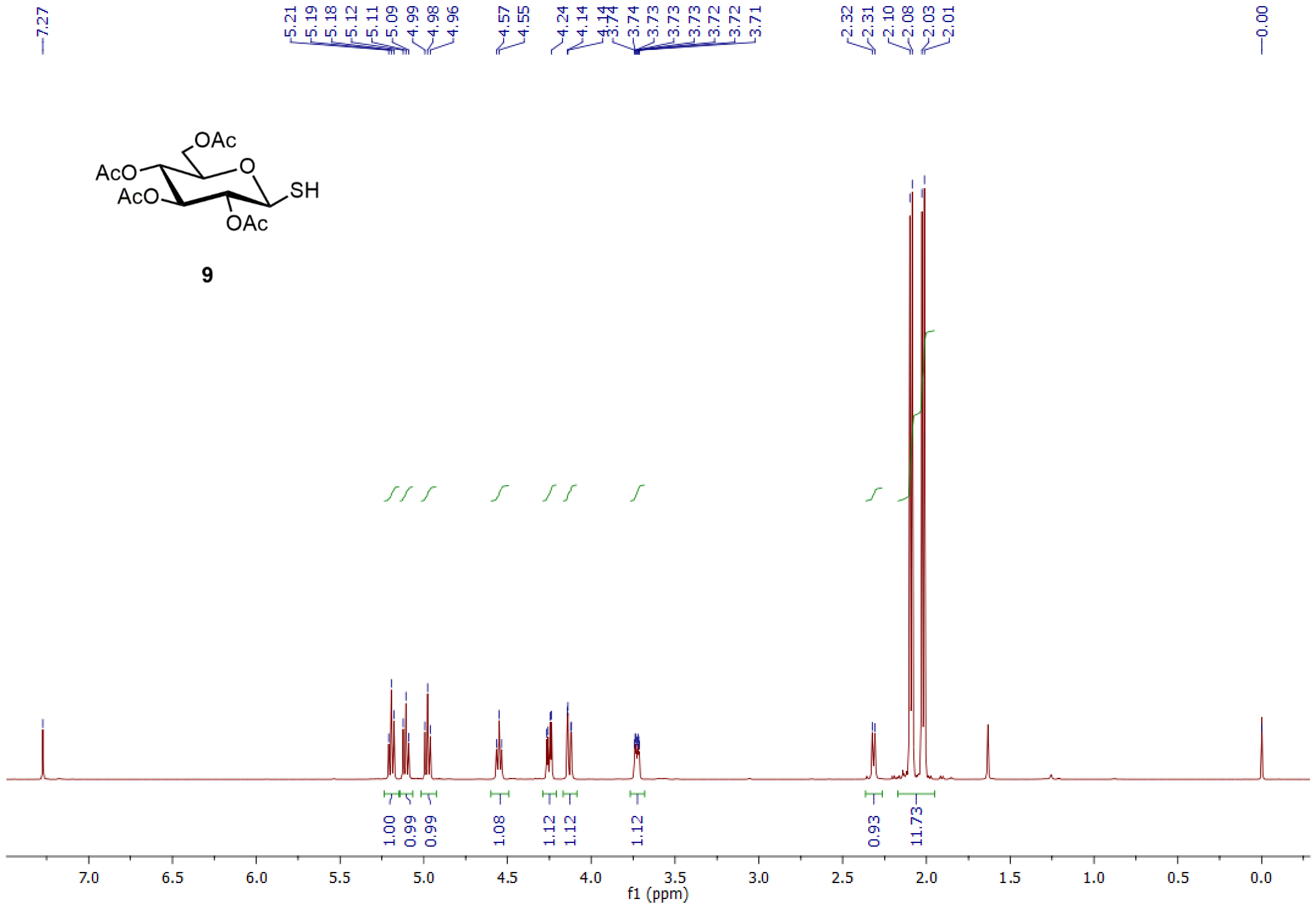

Fig. S2: ^{1}H NMR spectrum of compound **9** (400 MHz/$CDCl_3$/TMS; δ (ppm)).

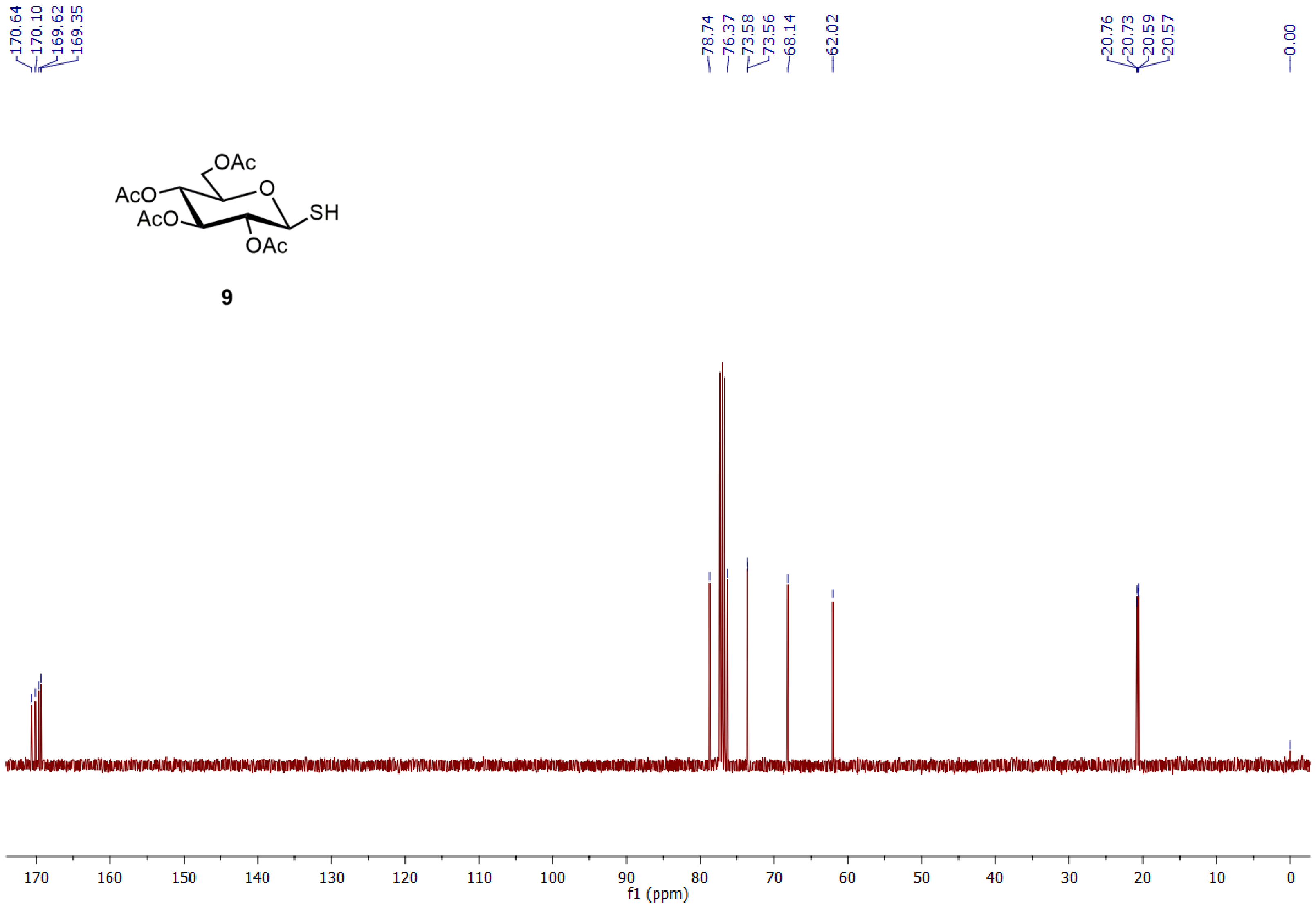

Fig. S3: ^{13}C NMR spectrum of compound **9** (100 MHz/$CDCl_3$/TMS; δ (ppm)).

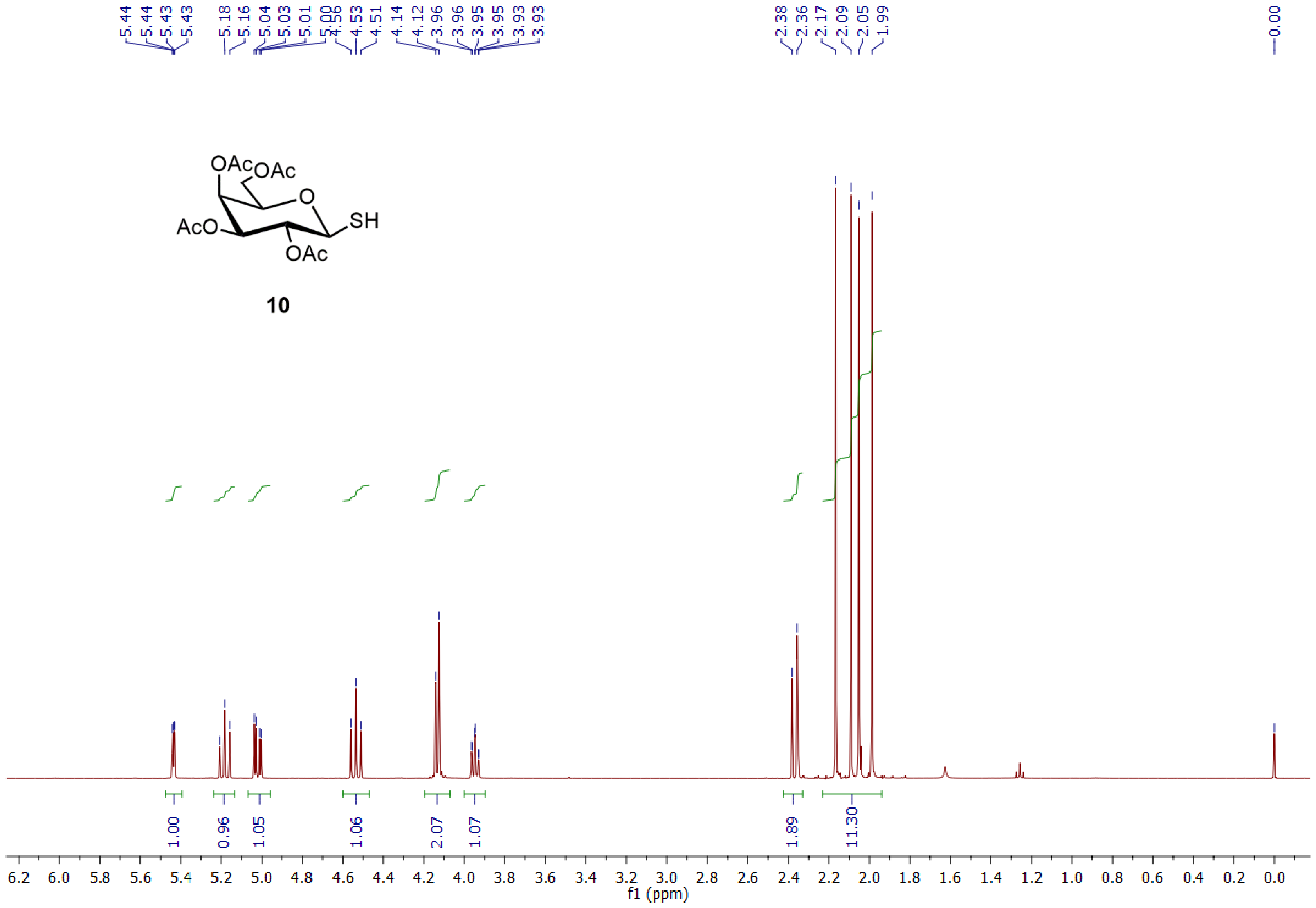

Fig. S4: ^{1}H NMR spectrum of compound **10** (400 MHz/$CDCl_3$/TMS; δ (ppm)).

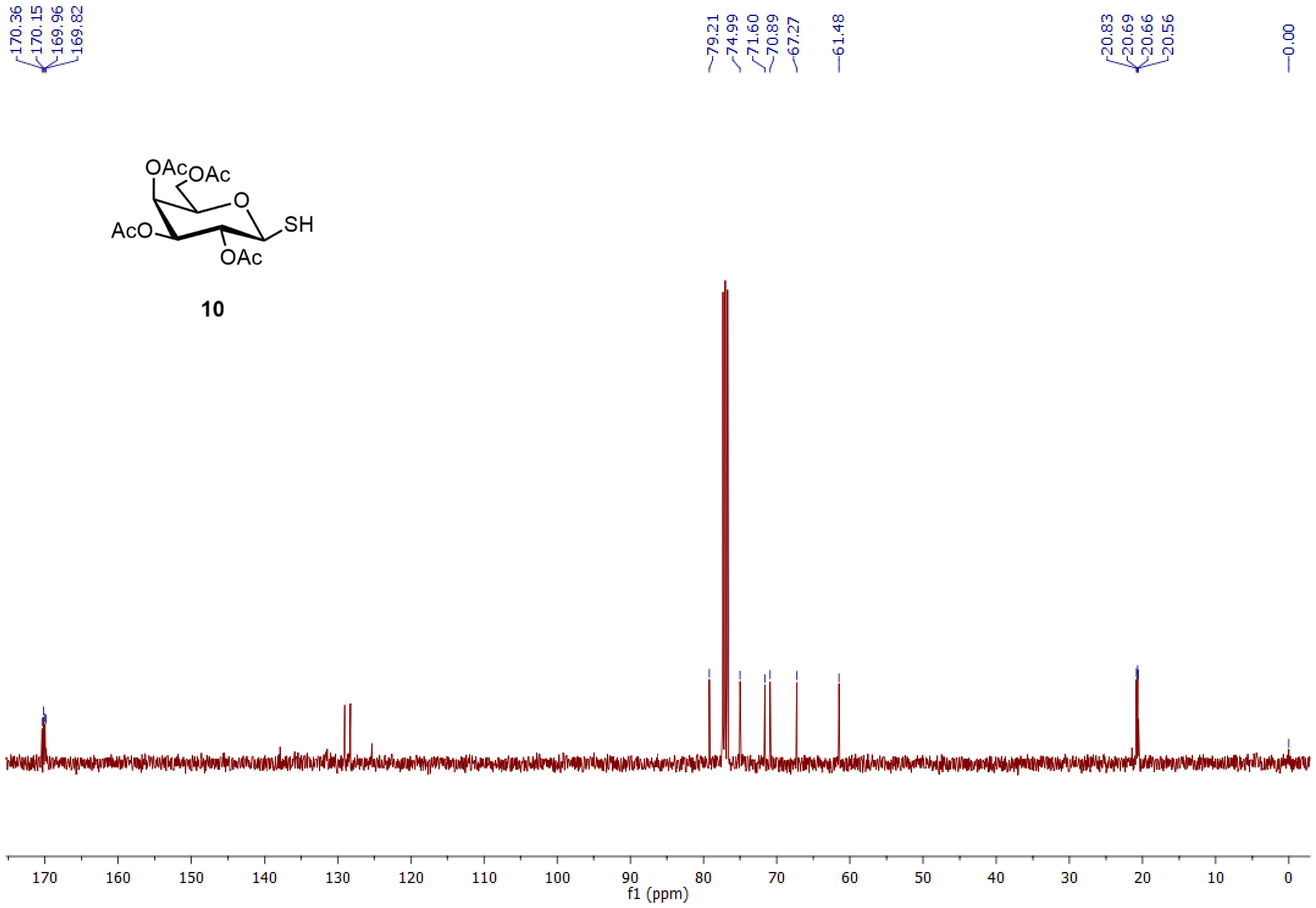

Fig. S5: ^{13}C NMR spectrum of compound **10** (100 MHz/$CDCl_3$/TMS; δ (ppm)).

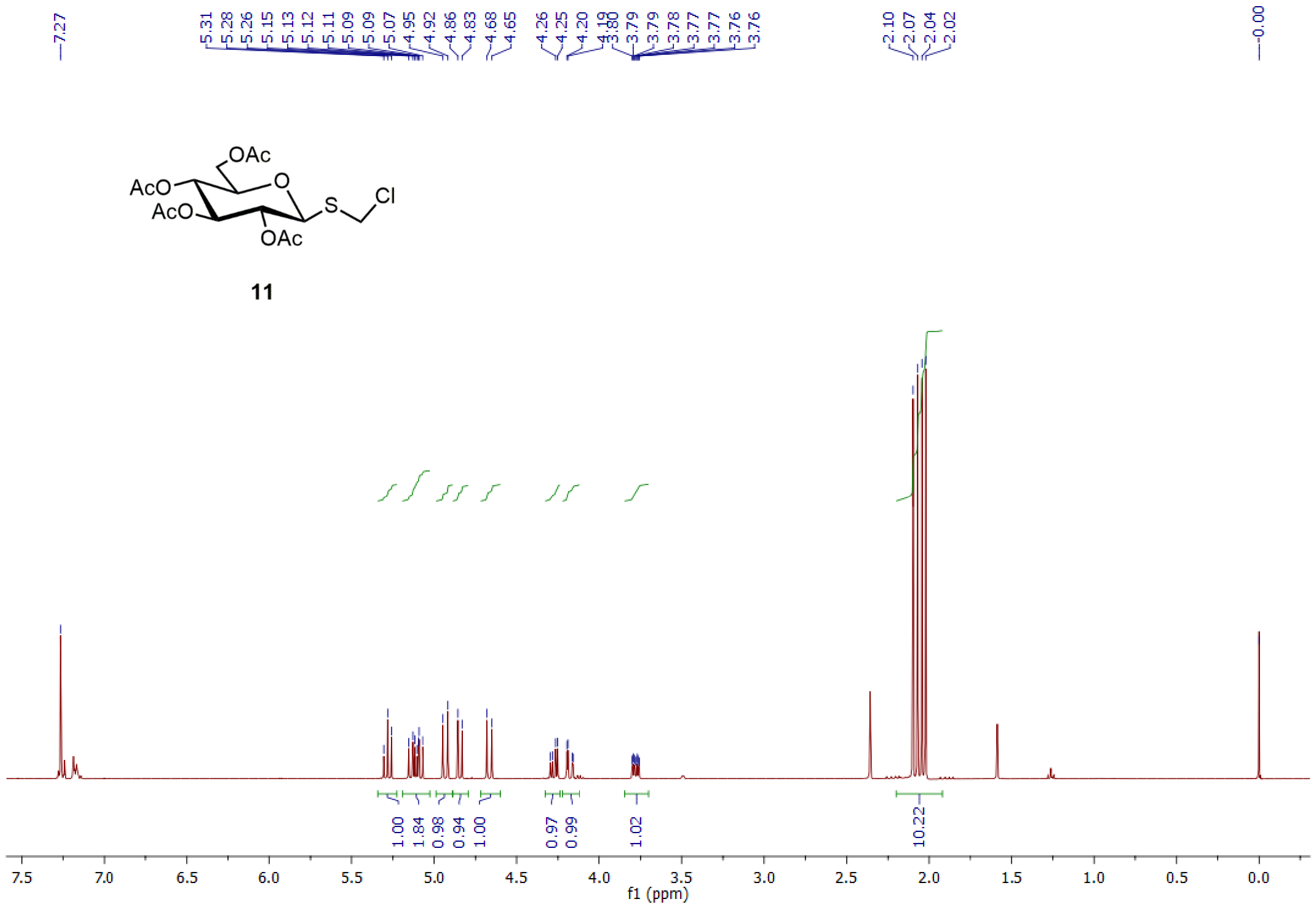

Fig. S6: ^{1}H NMR spectrum of compound **11** (400 MHz/$CDCl_3$/TMS; δ (ppm)).

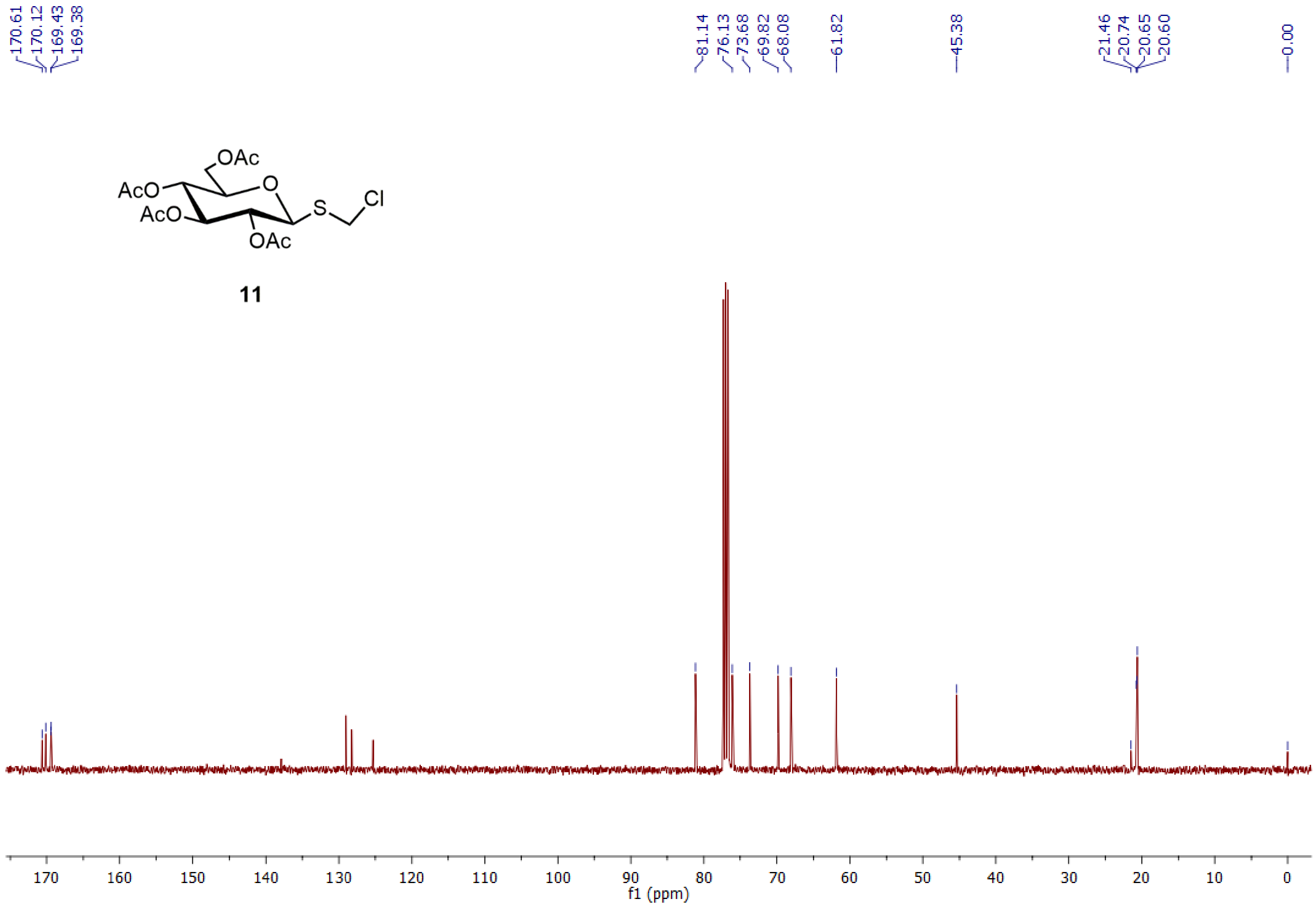

Fig. S7: ^{13}C NMR spectrum of compound **11** (100 MHz/$CDCl_3$/TMS; δ (ppm)).

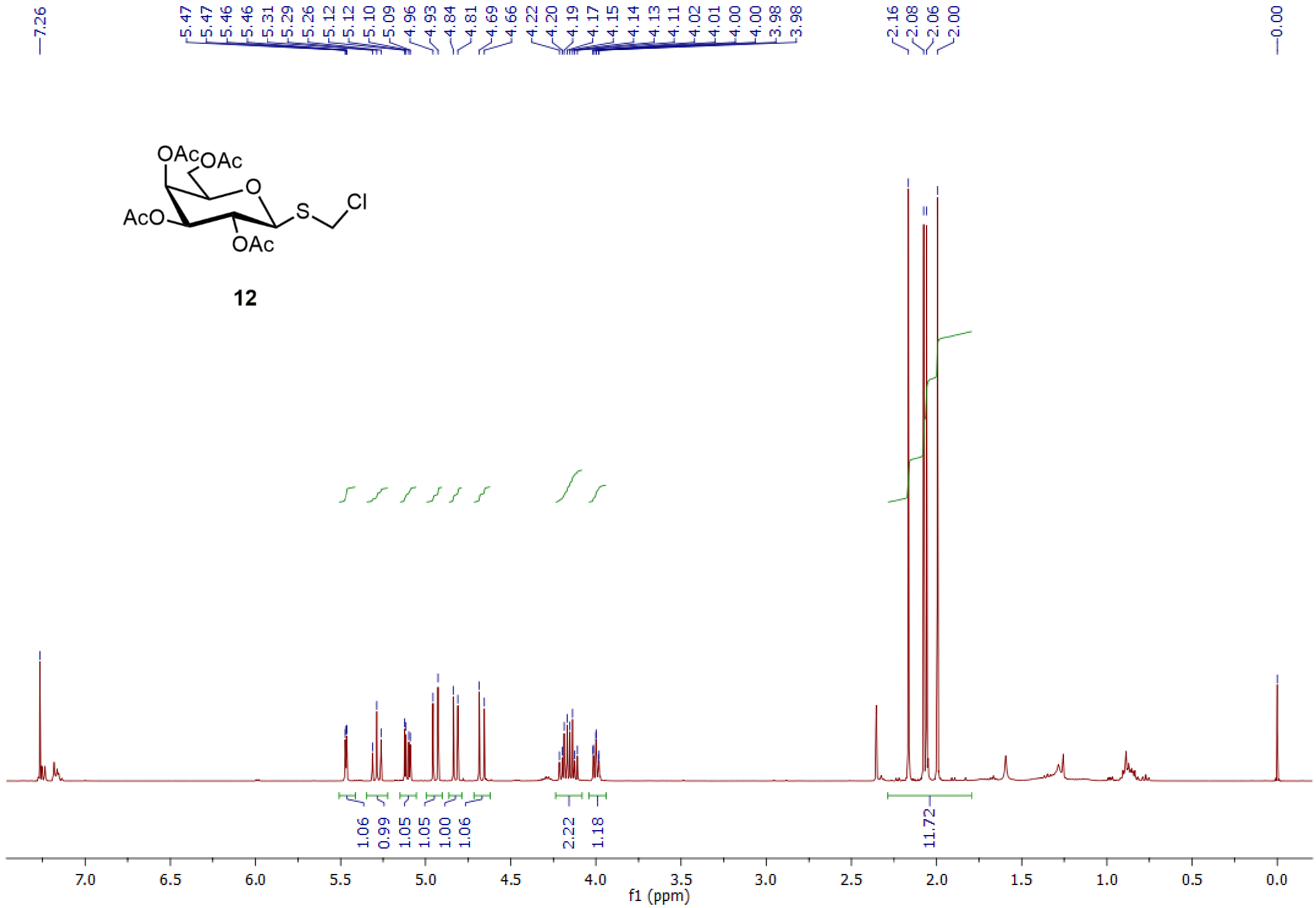

Fig. S8: ^{1}H NMR spectrum of compound **12** (400 MHz/$CDCl_3$/TMS; δ (ppm)).

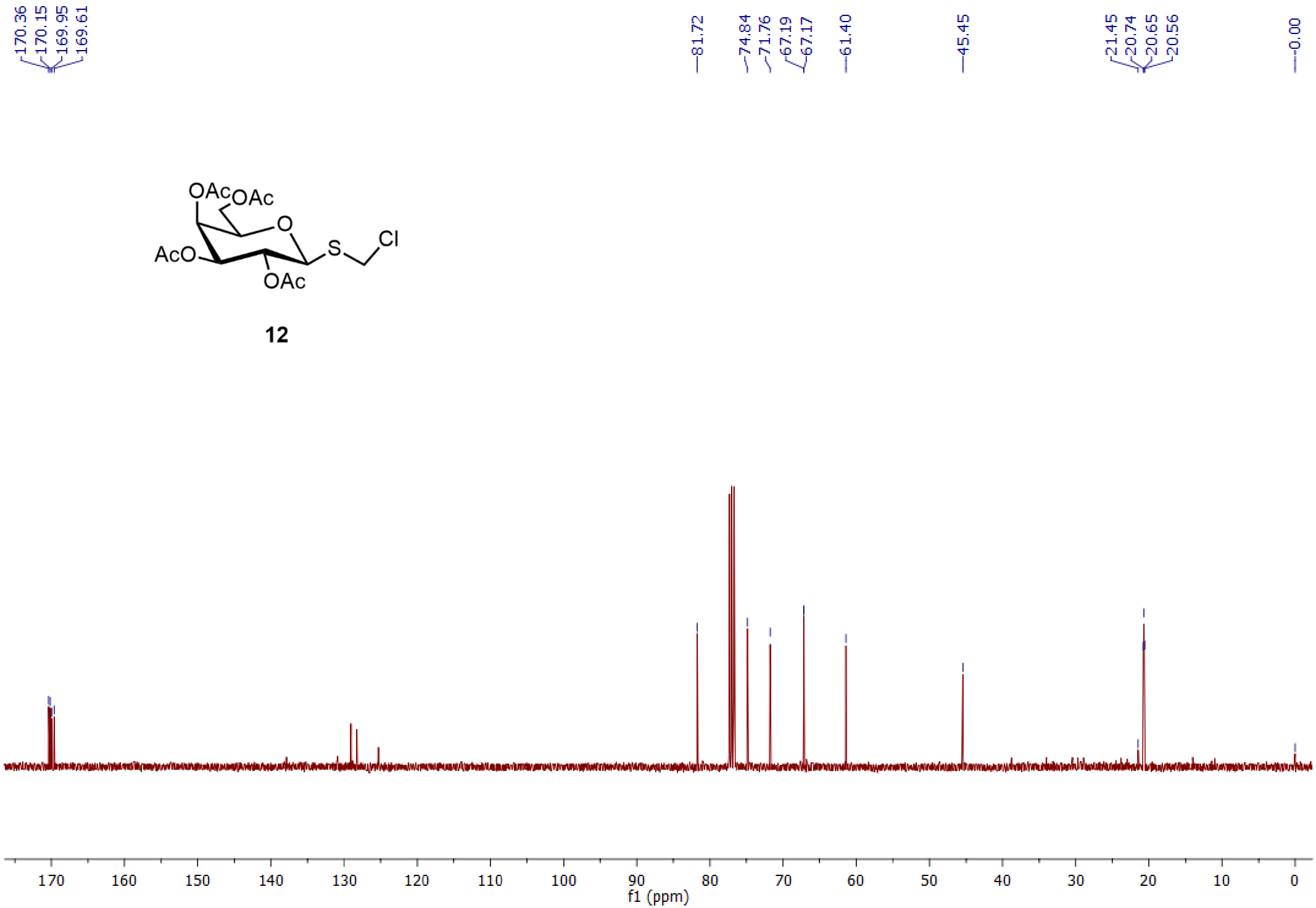

Fig. S9: ^{13}C NMR spectrum of compound **12** (100 MHz/$CDCl_3$/TMS; δ (ppm)).

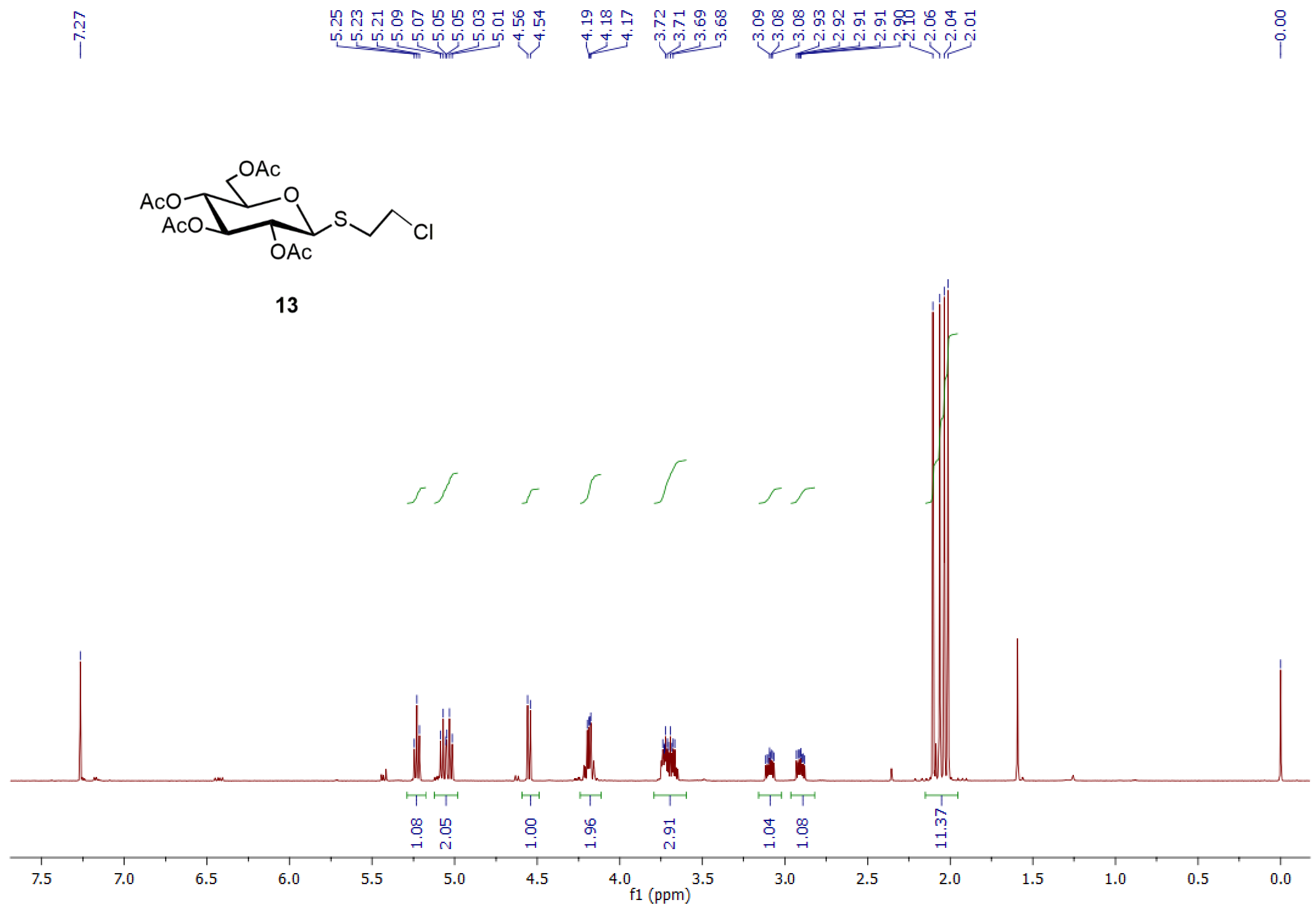

Fig. S10: ^{1}H NMR spectrum of compound **13** (400 MHz/$CDCl_3$/TMS; δ (ppm)).

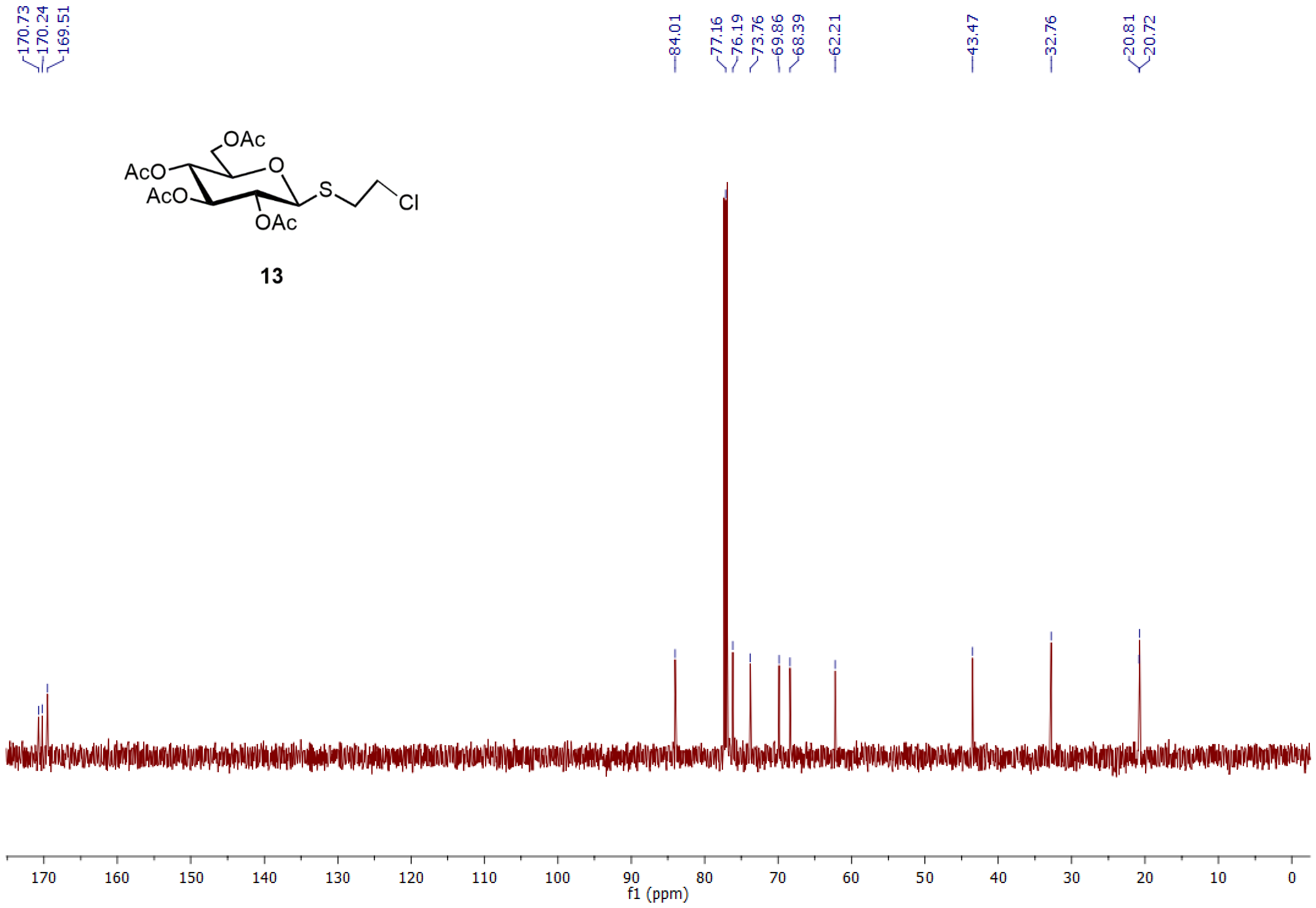

Fig. S11: ^{13}C NMR spectrum of compound **13** (100 MHz/$CDCl_3$/TMS; δ (ppm)).

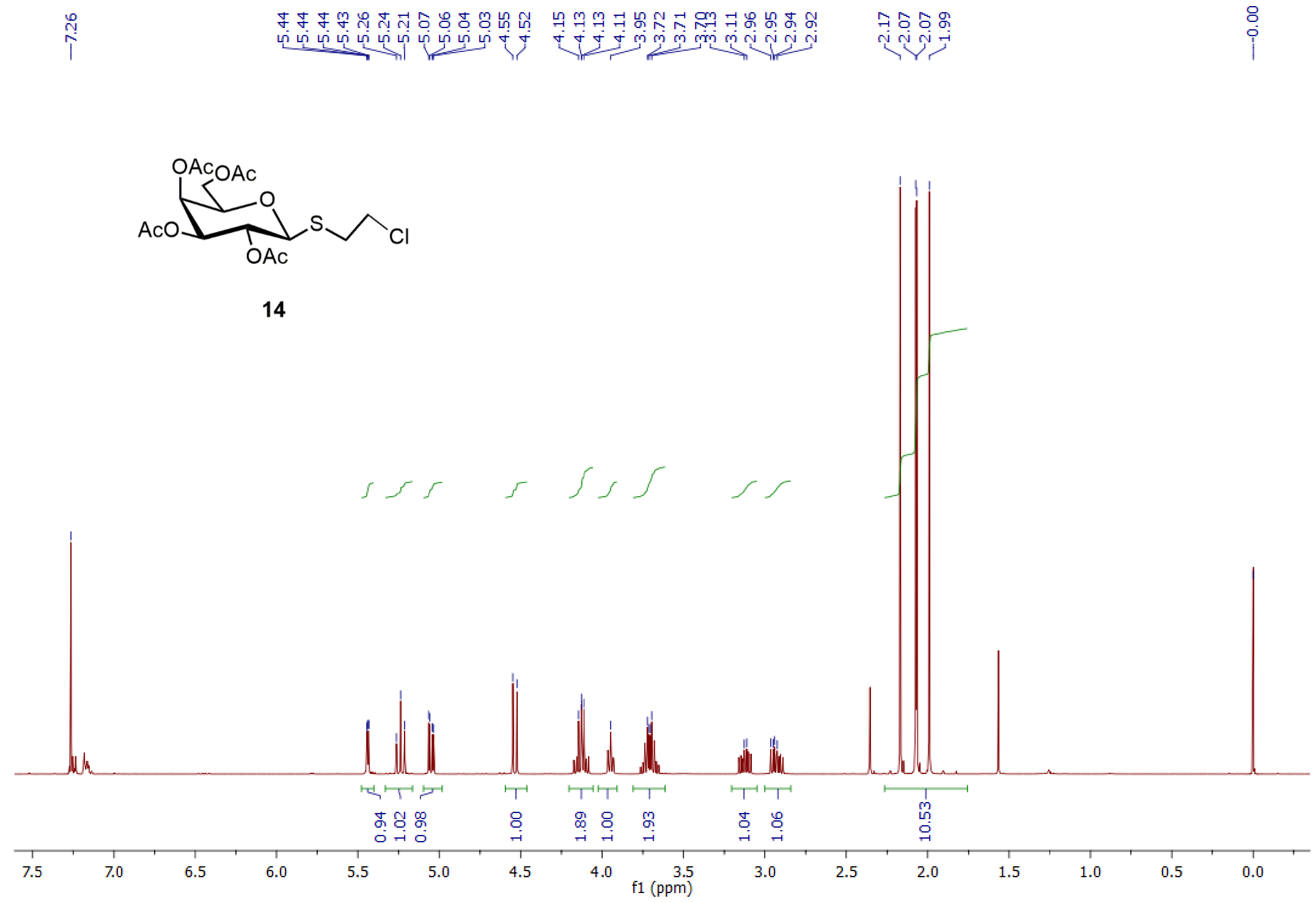

Fig. S12: ^{1}H NMR spectrum of compound **14** (400 MHz/$CDCl_3$/TMS; δ (ppm)).

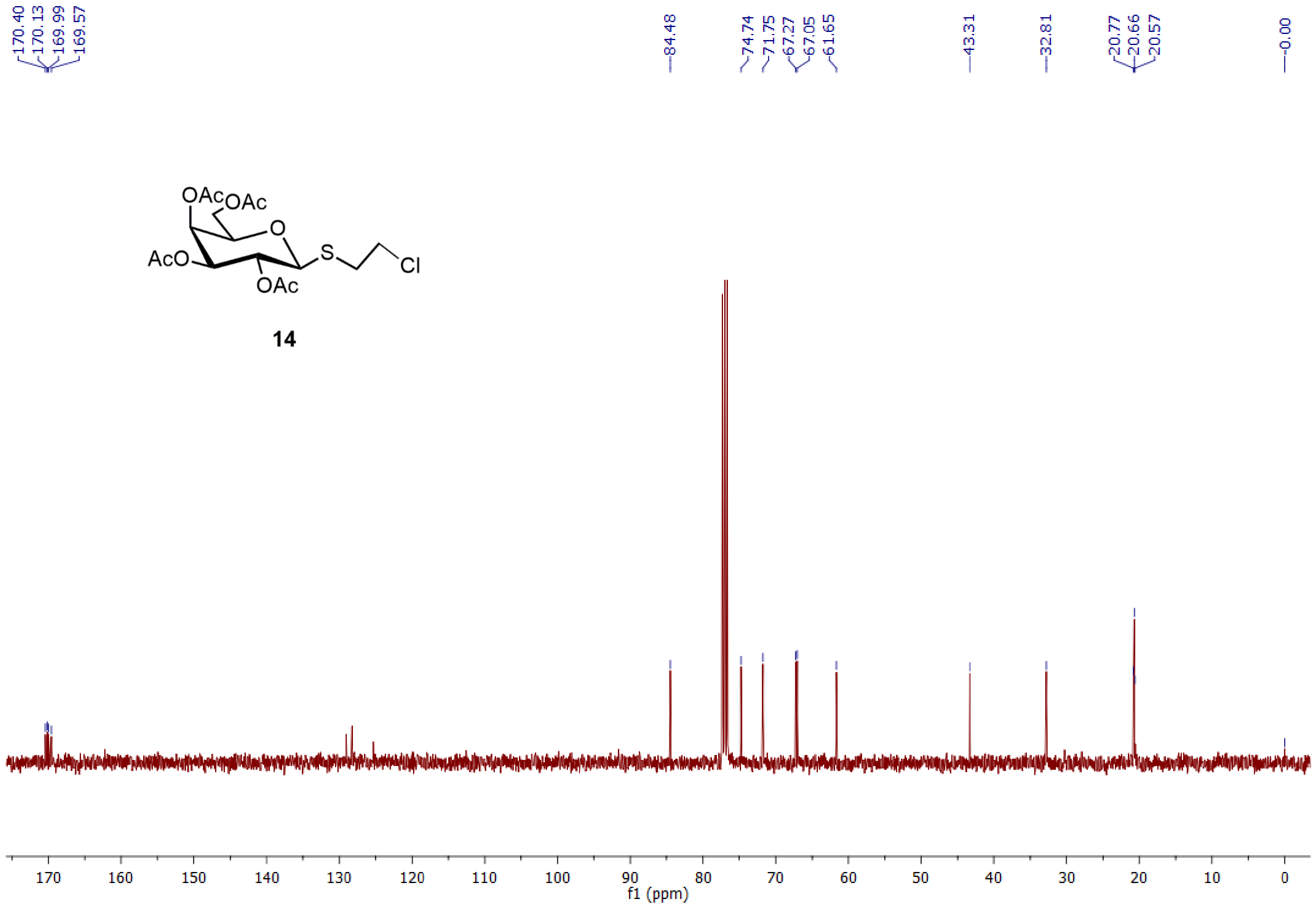

Fig. S13: ^{13}C NMR spectrum of compound **14** (100 MHz/$CDCl_3$/TMS; δ (ppm)).

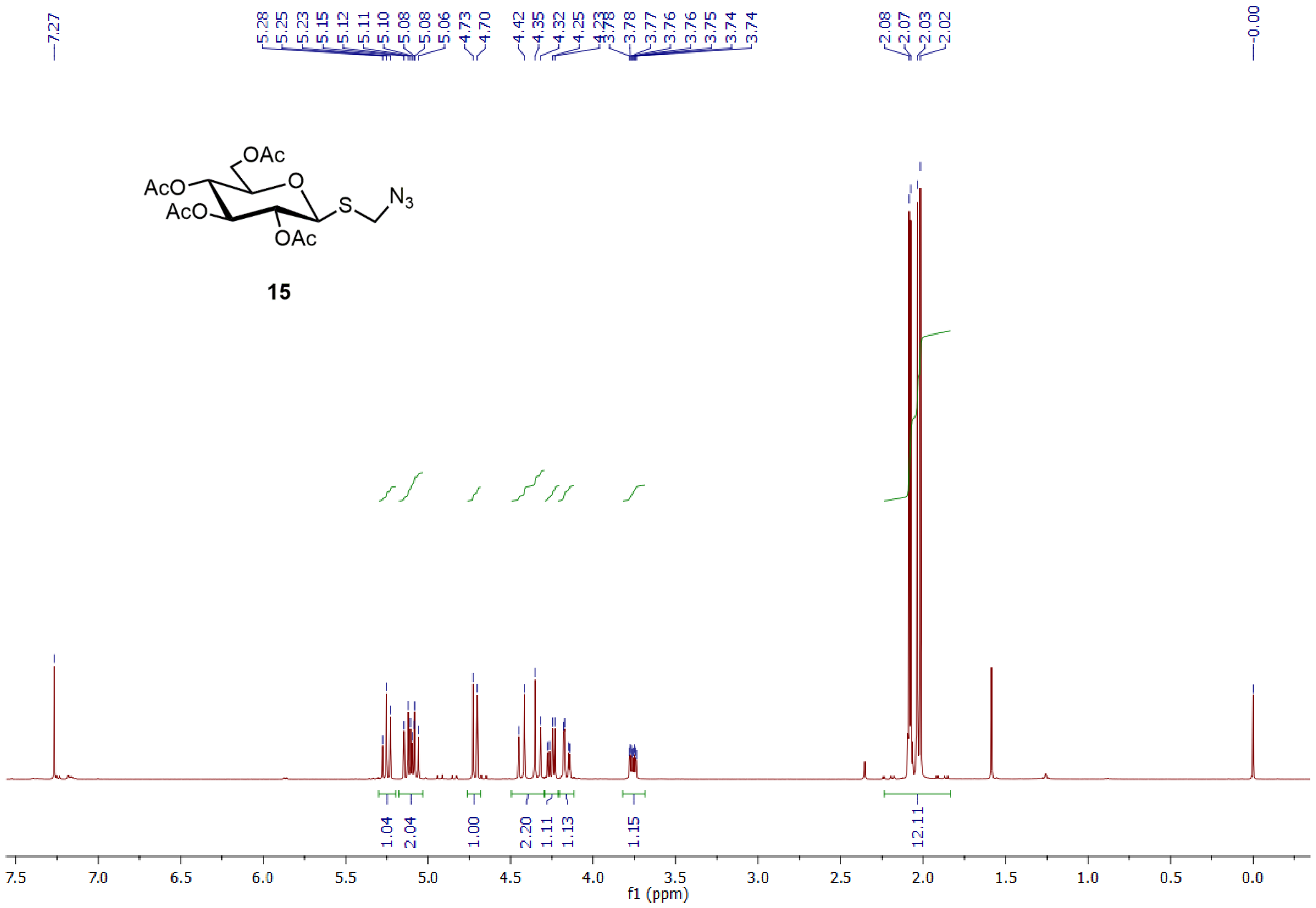

Fig. S14: ^1H NMR spectrum of compound **15** (400 MHz/$CDCl_3$/TMS; δ (ppm)).

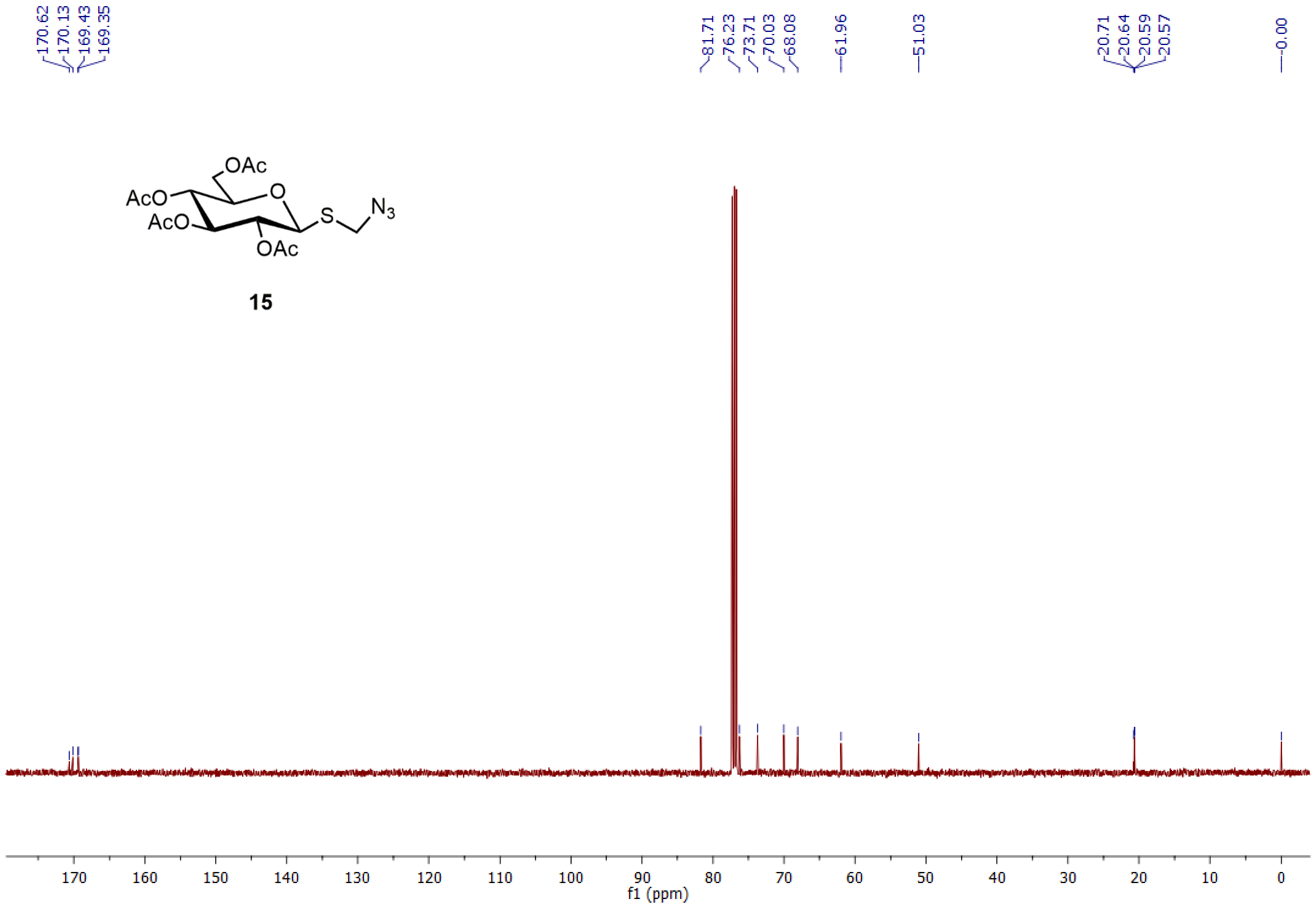

Fig. S15: ^{13}C NMR spectrum of compound **15** (100 MHz/$CDCl_3$/TMS; δ (ppm)).

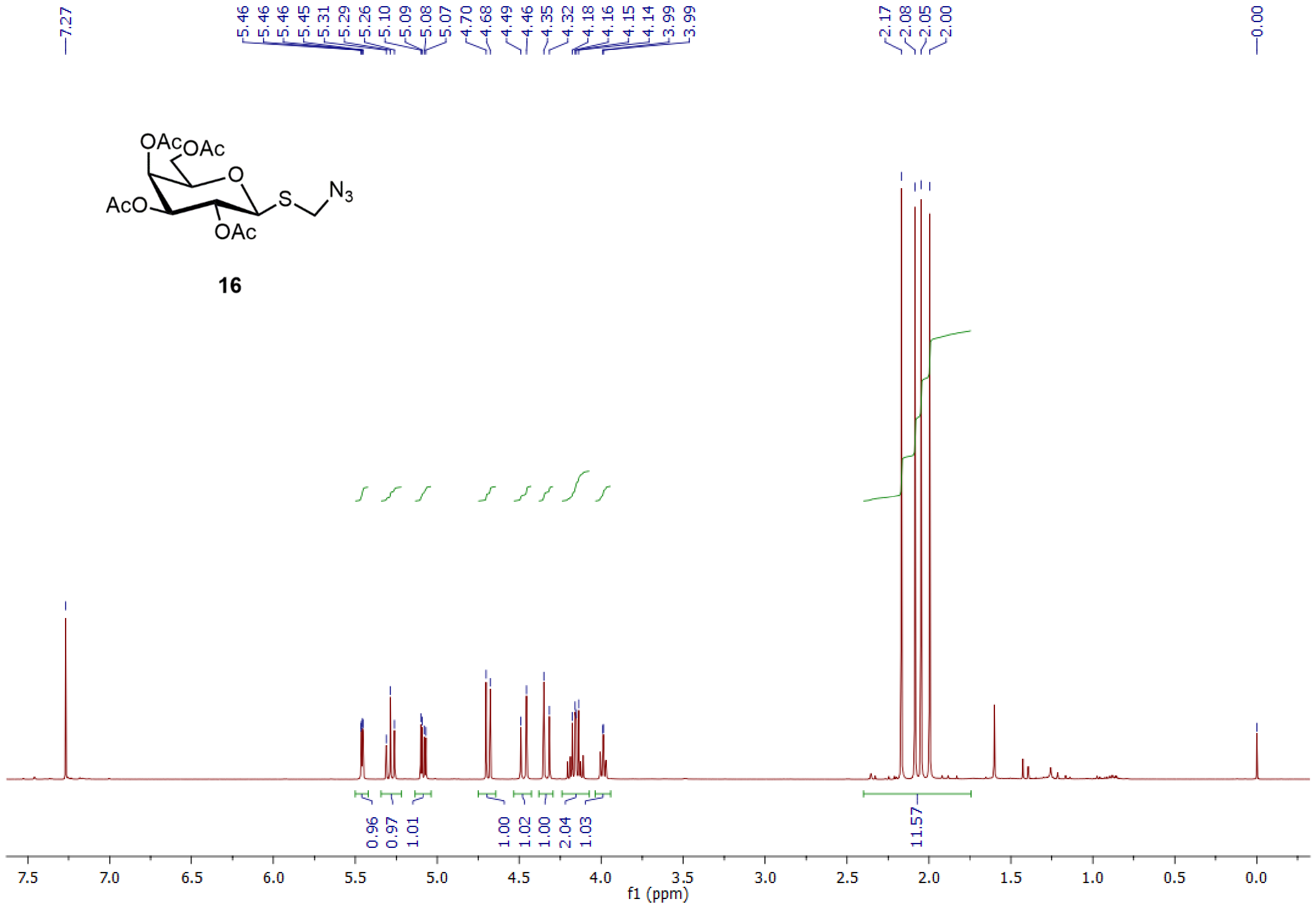

Fig. S16: ^{1}H NMR spectrum of compound **16** (400 MHz/$CDCl_3$/TMS; δ (ppm)).

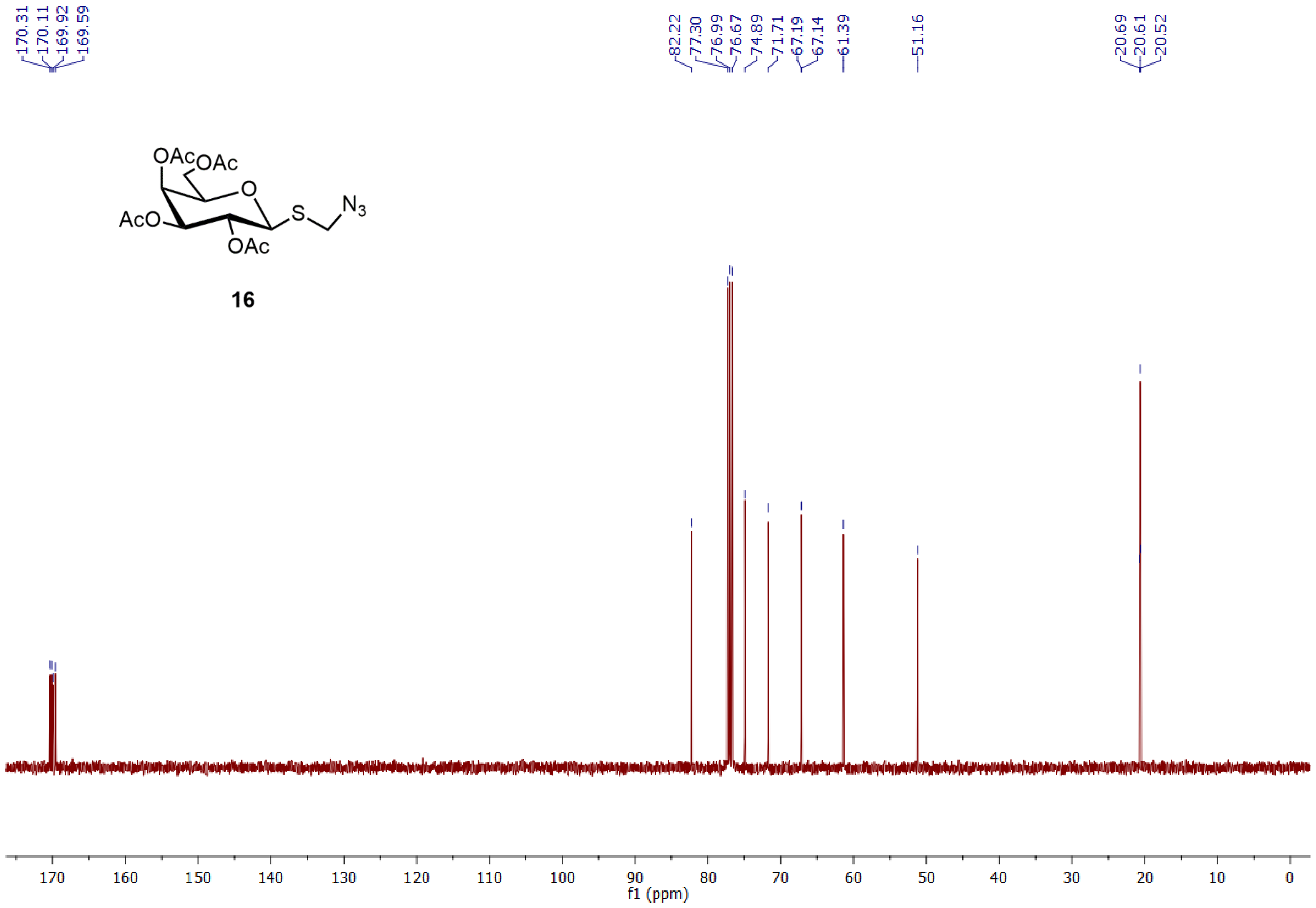

Fig. S17: ^{13}C NMR spectrum of compound **16** (100 MHz/$CDCl_3$/TMS; δ (ppm)).

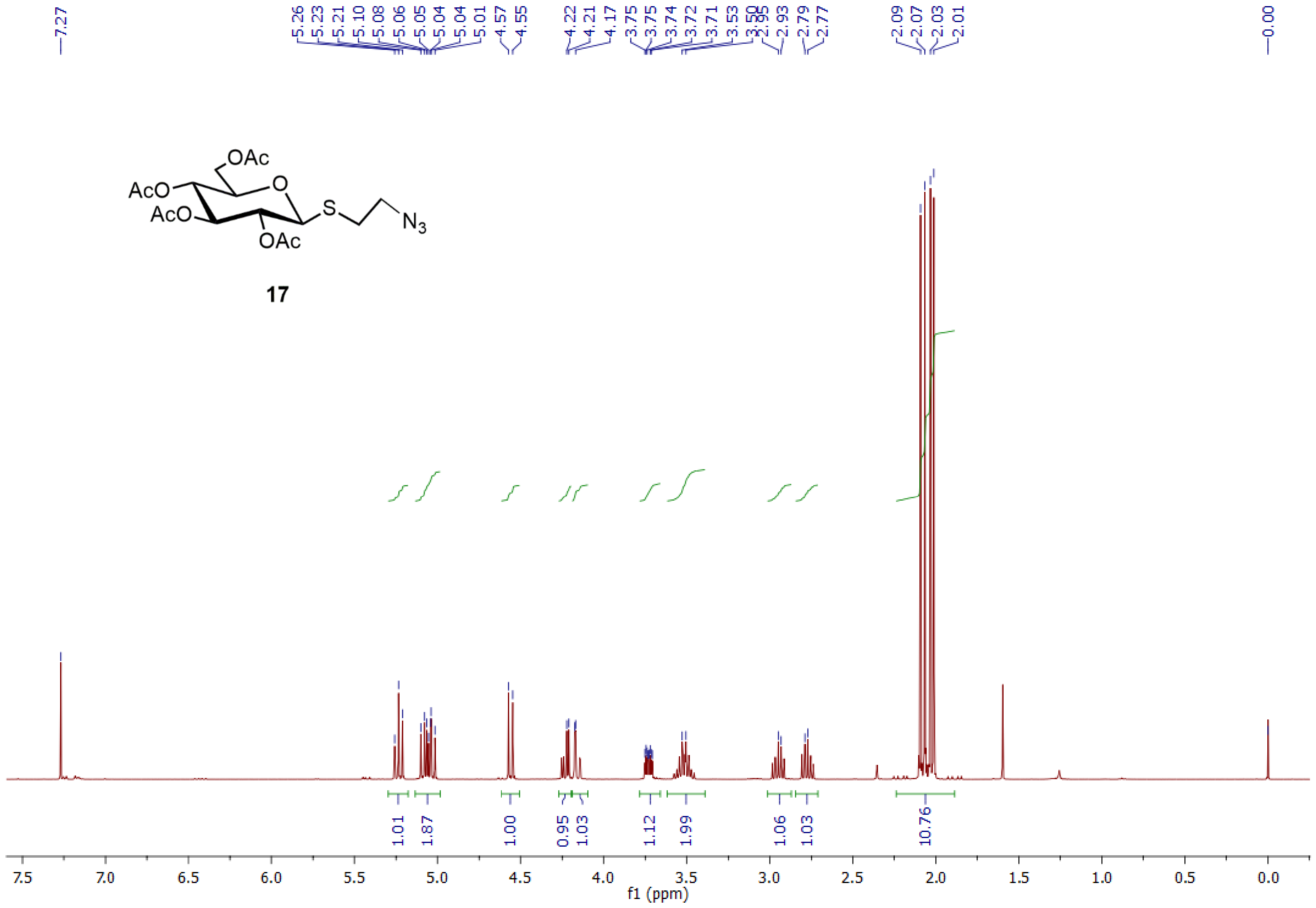

Fig. S18: ^{1}H NMR spectrum of compound **17** (400 MHz/$CDCl_3$/TMS; δ (ppm)).

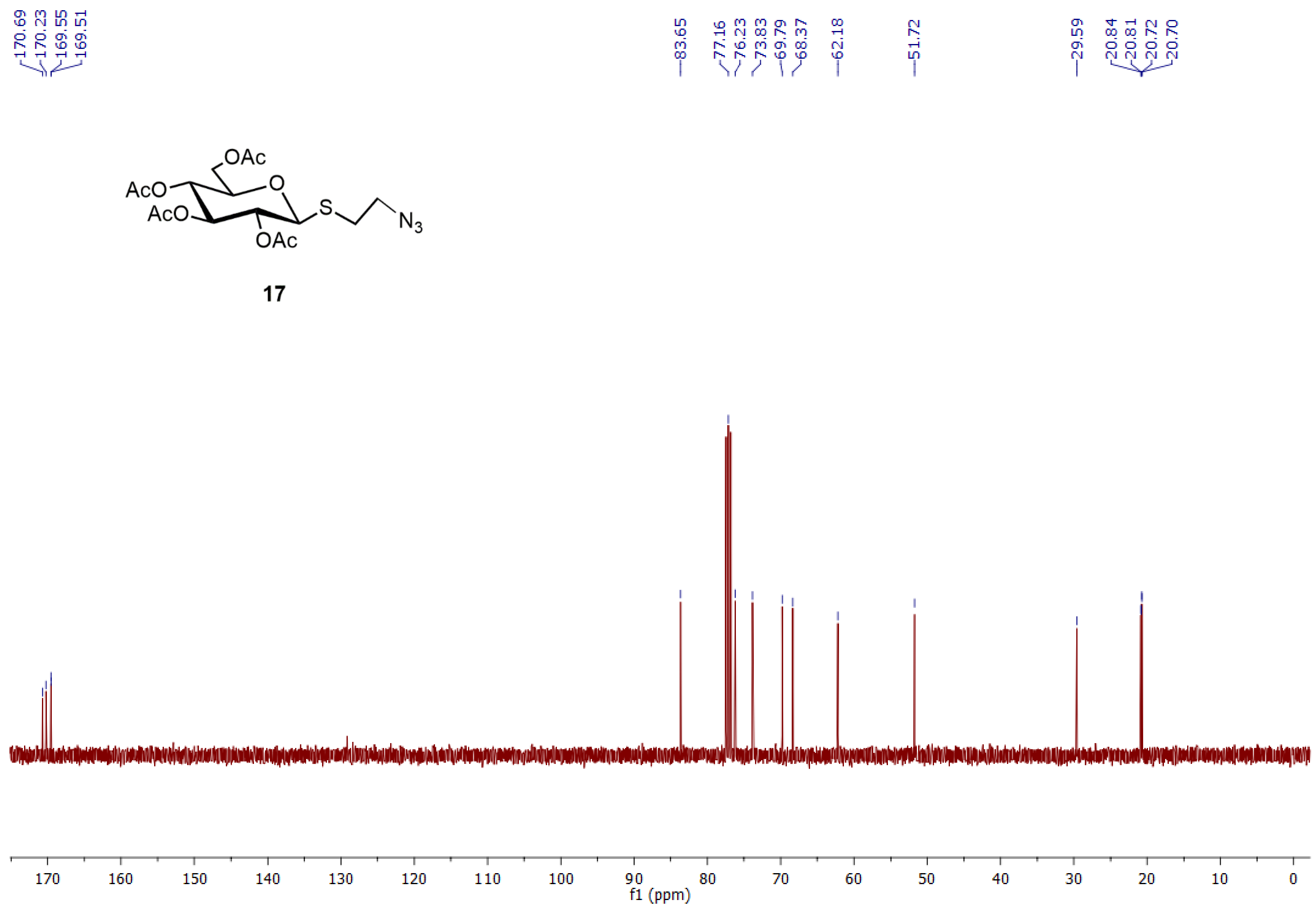

Fig. S19: ^{13}C NMR spectrum of compound **17** (100 MHz/$CDCl_3$/TMS; δ (ppm)).

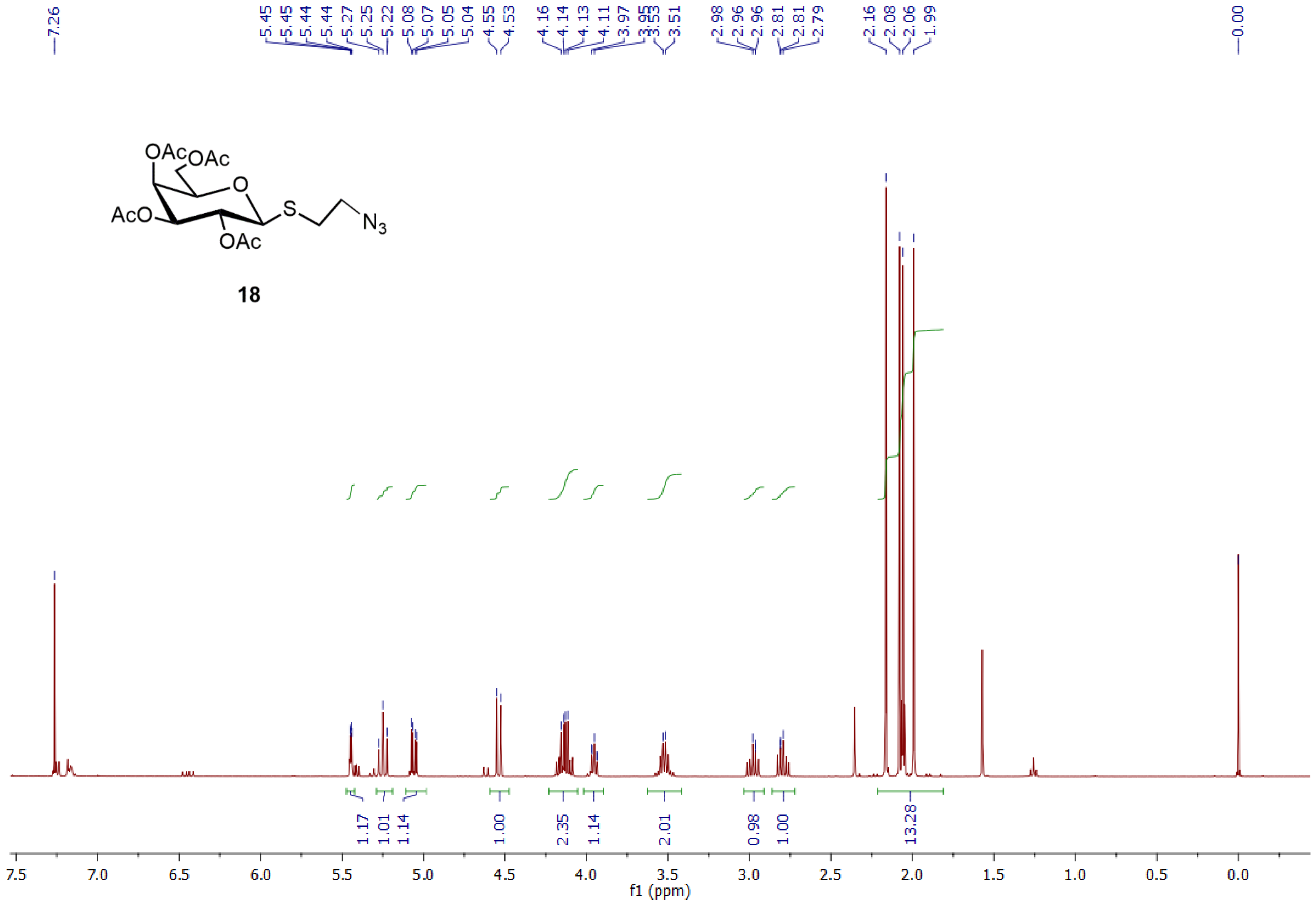

Fig. S20: ¹H NMR spectrum of compound **18** (400 MHz/$CDCl_3$/TMS; δ (ppm)).

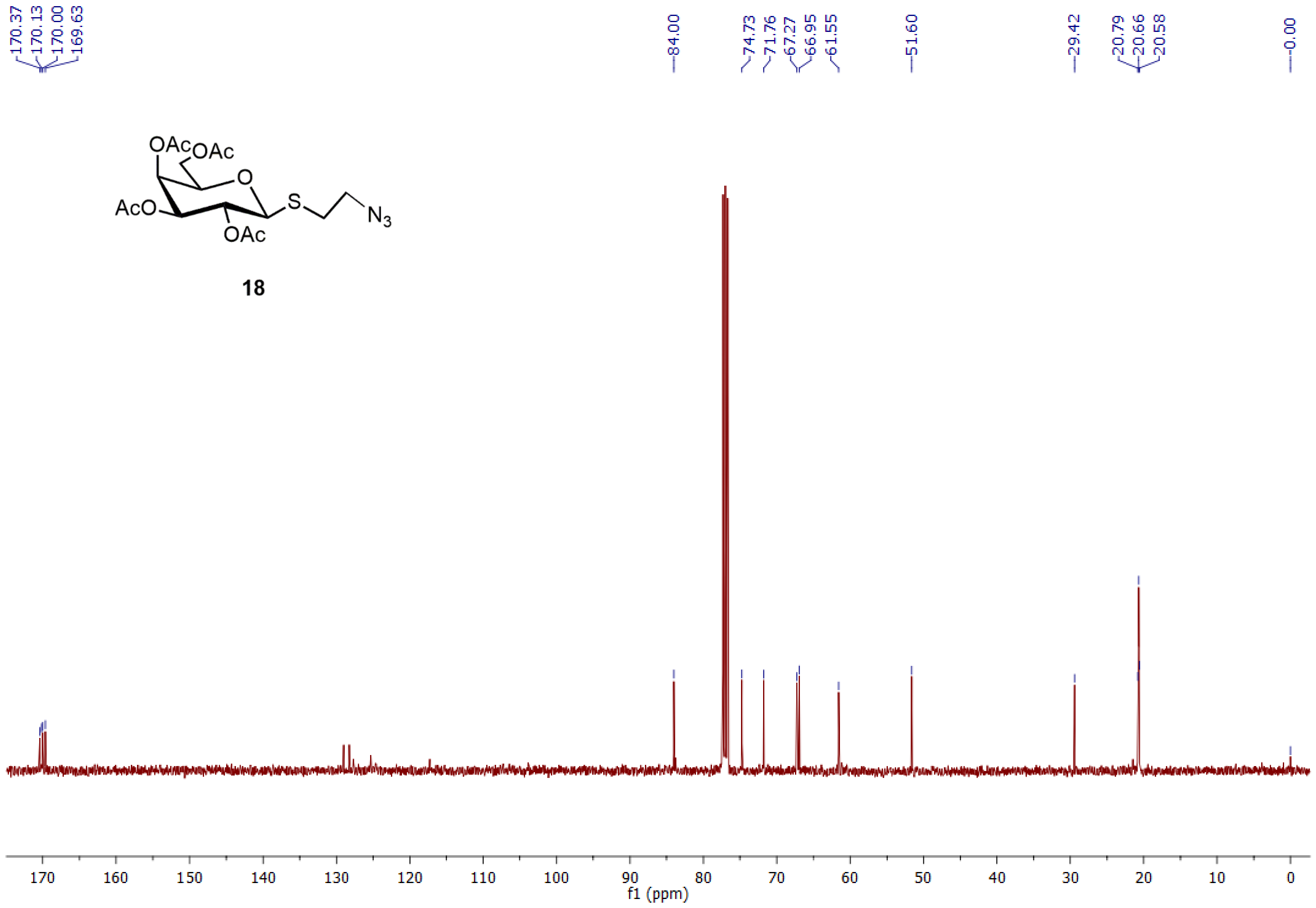

Fig. S21: ^{13}C NMR spectrum of compound **18** (100 MHz/$CDCl_3$/TMS; δ (ppm)).

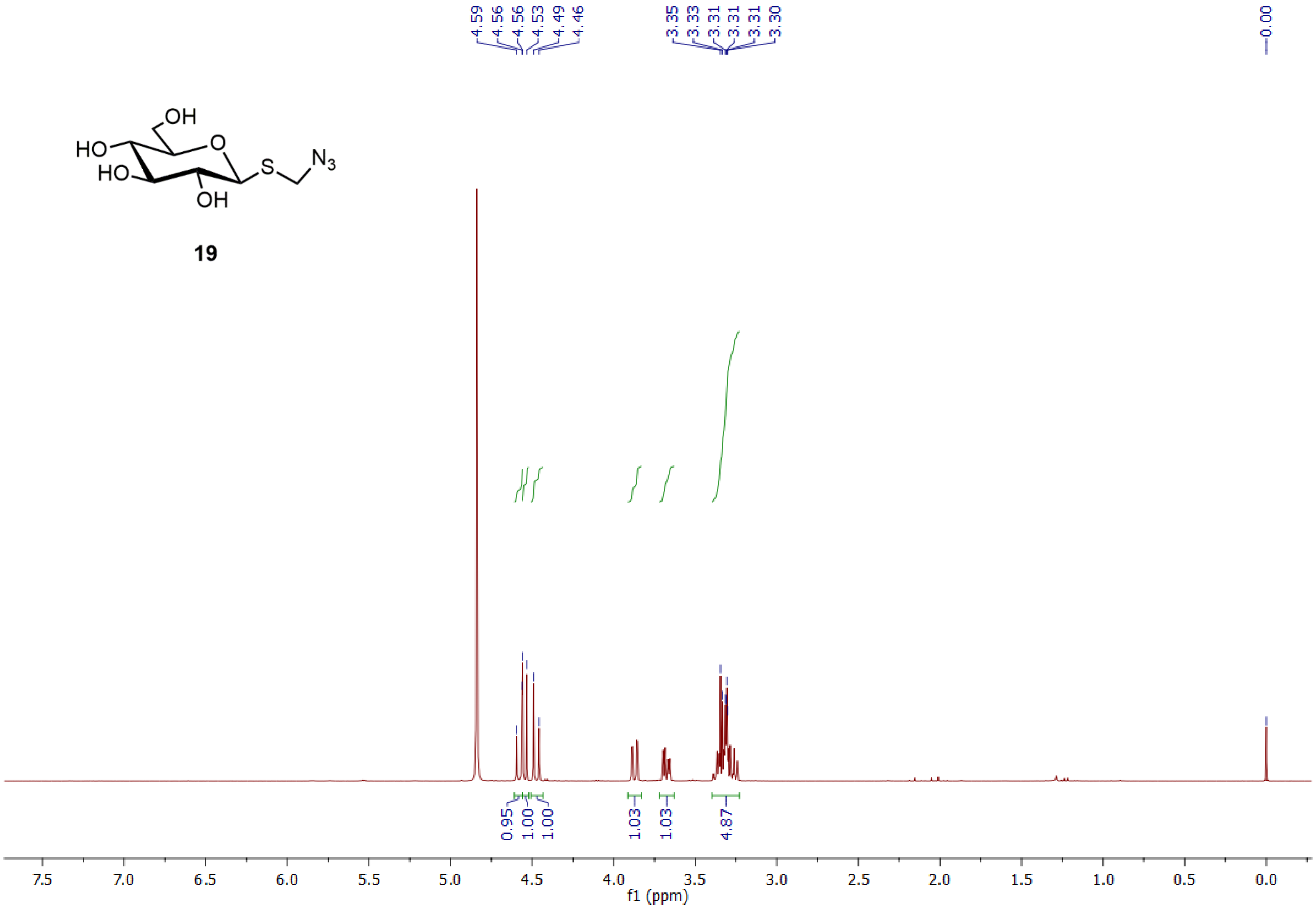

Fig. S22: ^{1}H NMR spectrum of compound **19** (400 MHz/CD_3OD/TMS; δ (ppm)).

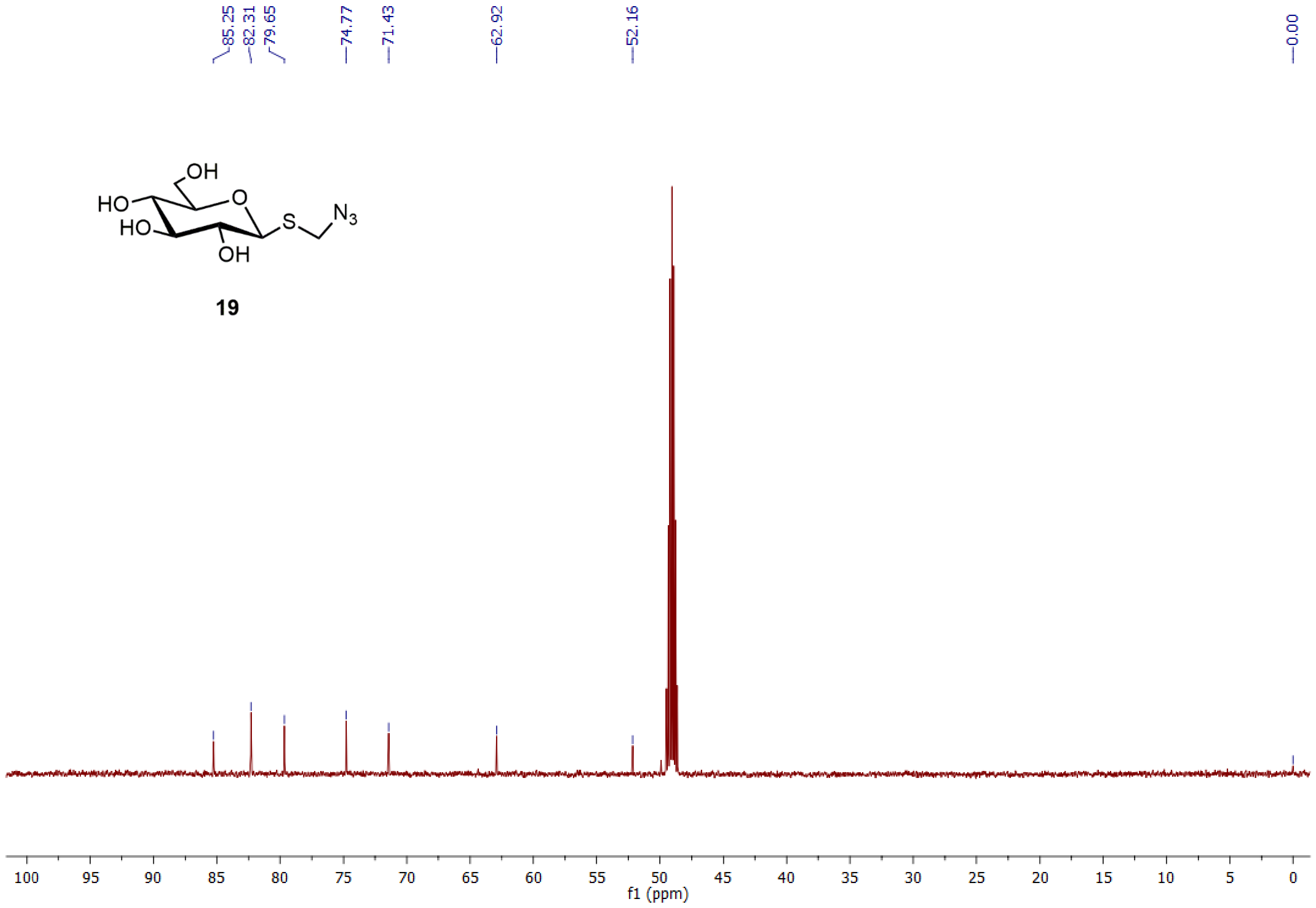

Fig. S23: ^{13}C NMR spectrum of compound **19** (100 MHz/CD_3OD/TMS; δ (ppm)).

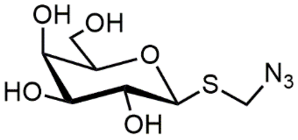

20

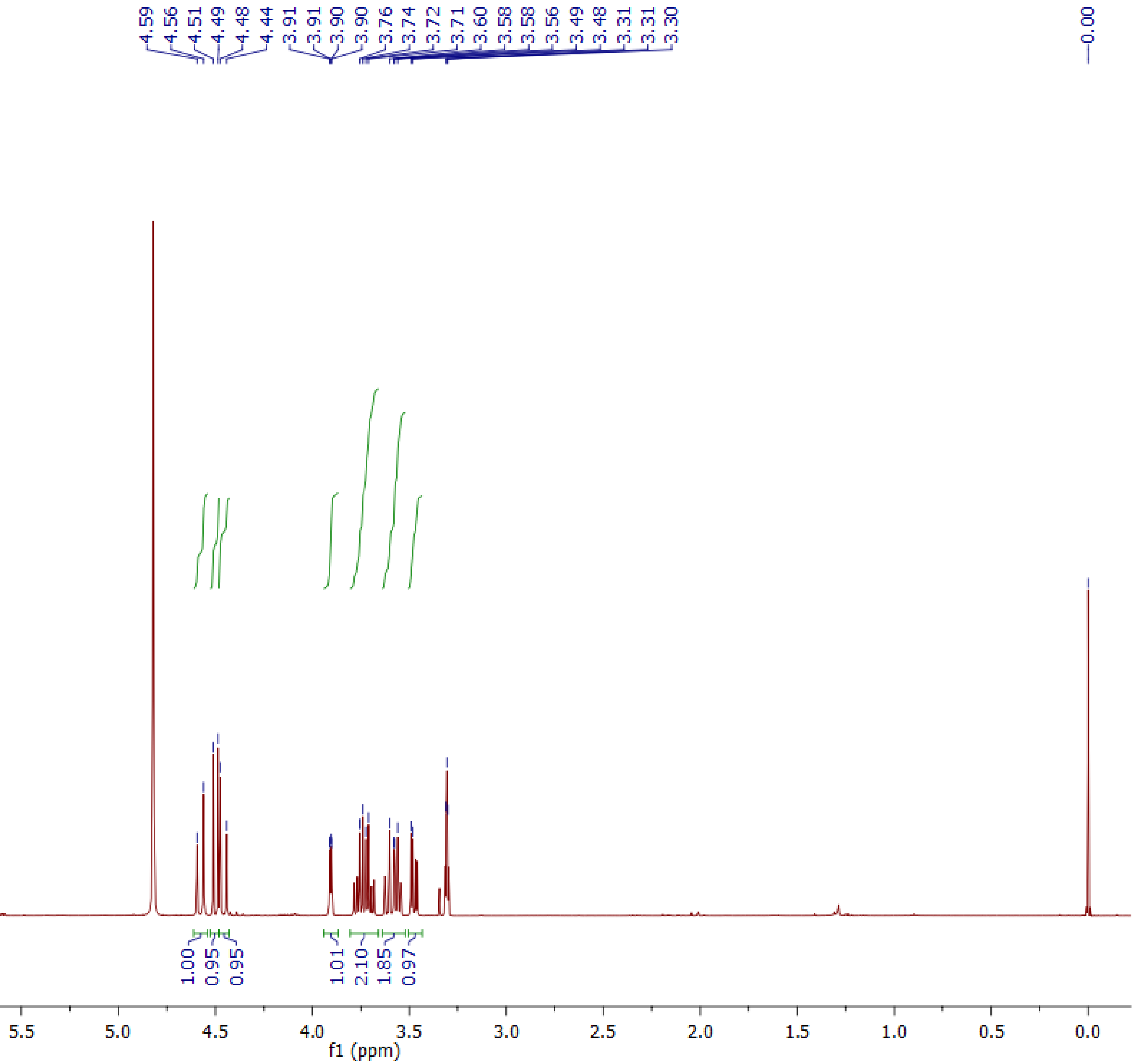

Fig. S24: ^{1}H NMR spectrum of compound **20** (400 MHz/CD_3OD/TMS; δ (ppm)).

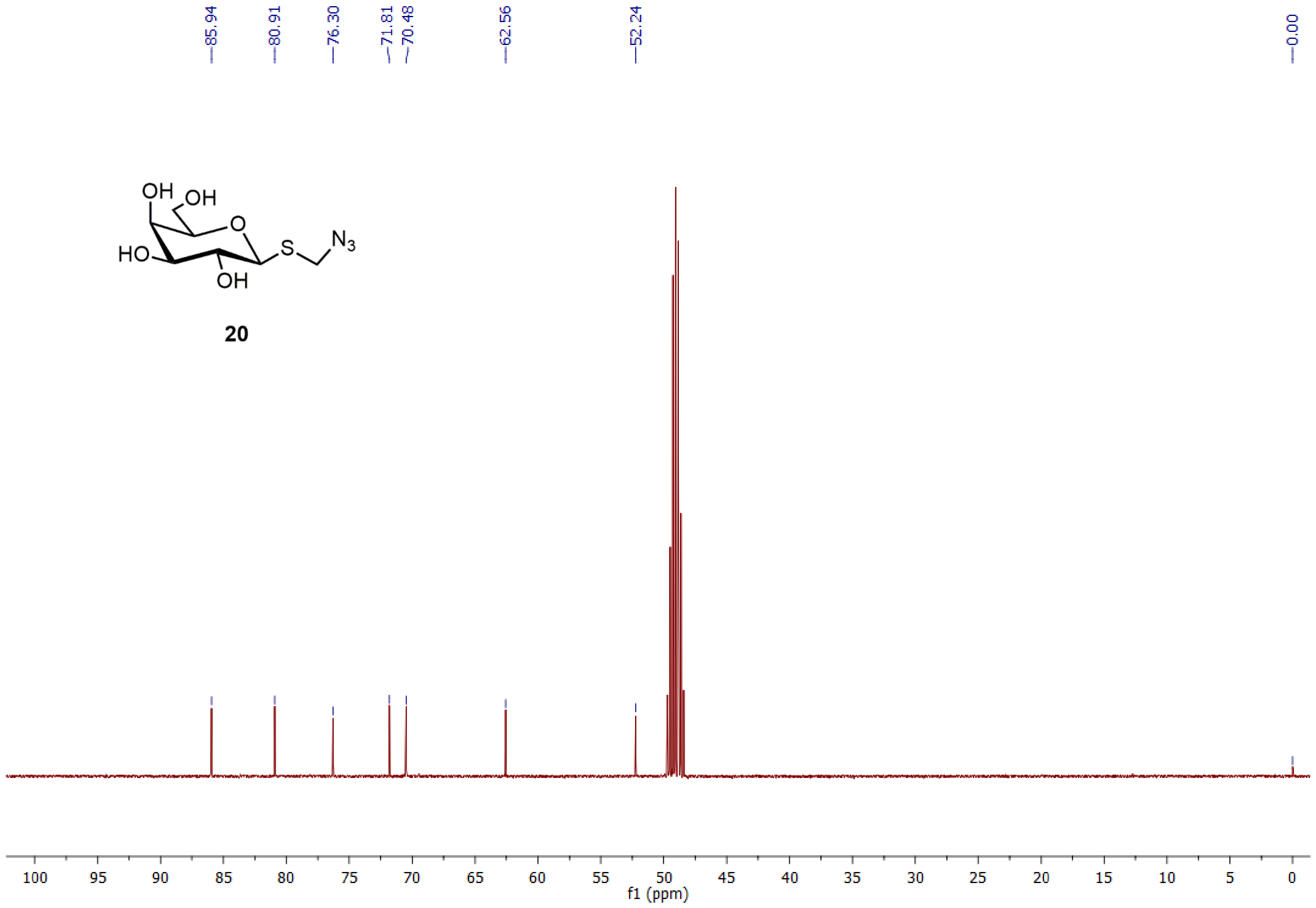

Fig. S25: ^{13}C NMR spectrum of compound **20** (100 MHz/CD_3OD/TMS; δ (ppm)).

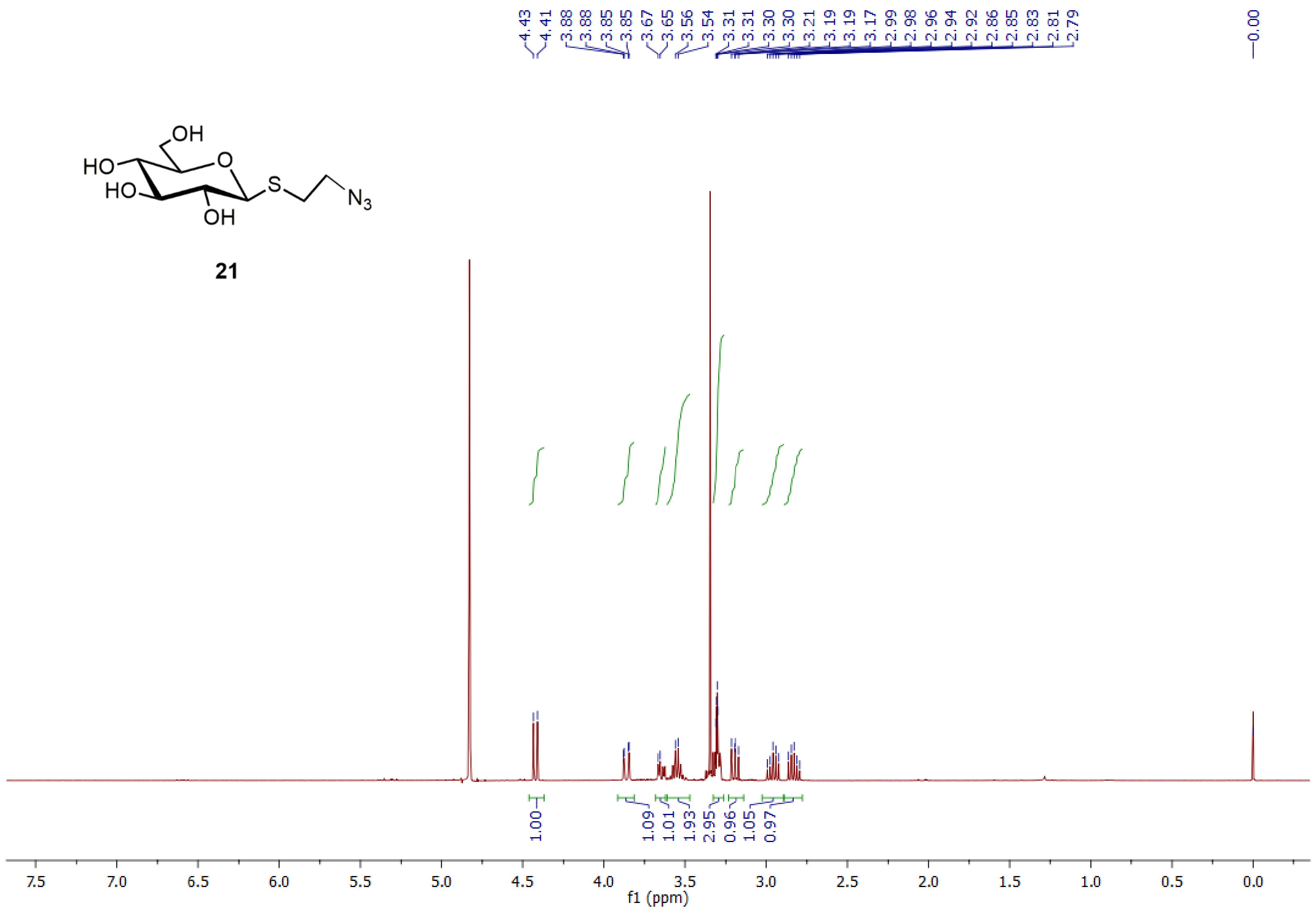

Fig. S26: ^{1}H NMR spectrum of compound **21** (400 MHz/CD_3OD/TMS; δ (ppm)).

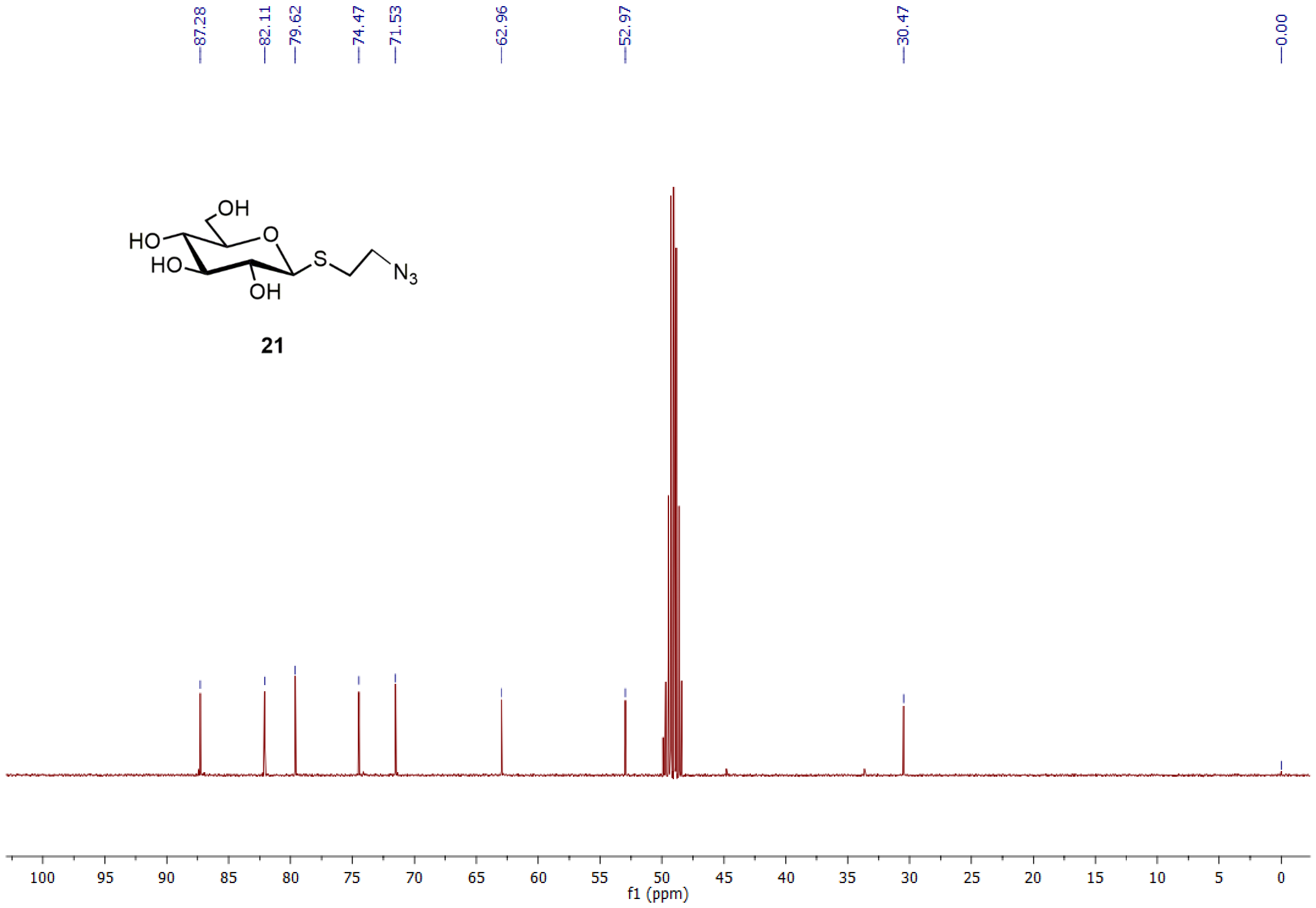

Fig. S27: ^{13}C NMR spectrum of compound **21** (100 MHz/CD_3OD/TMS; δ (ppm)).

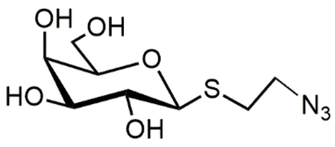

22

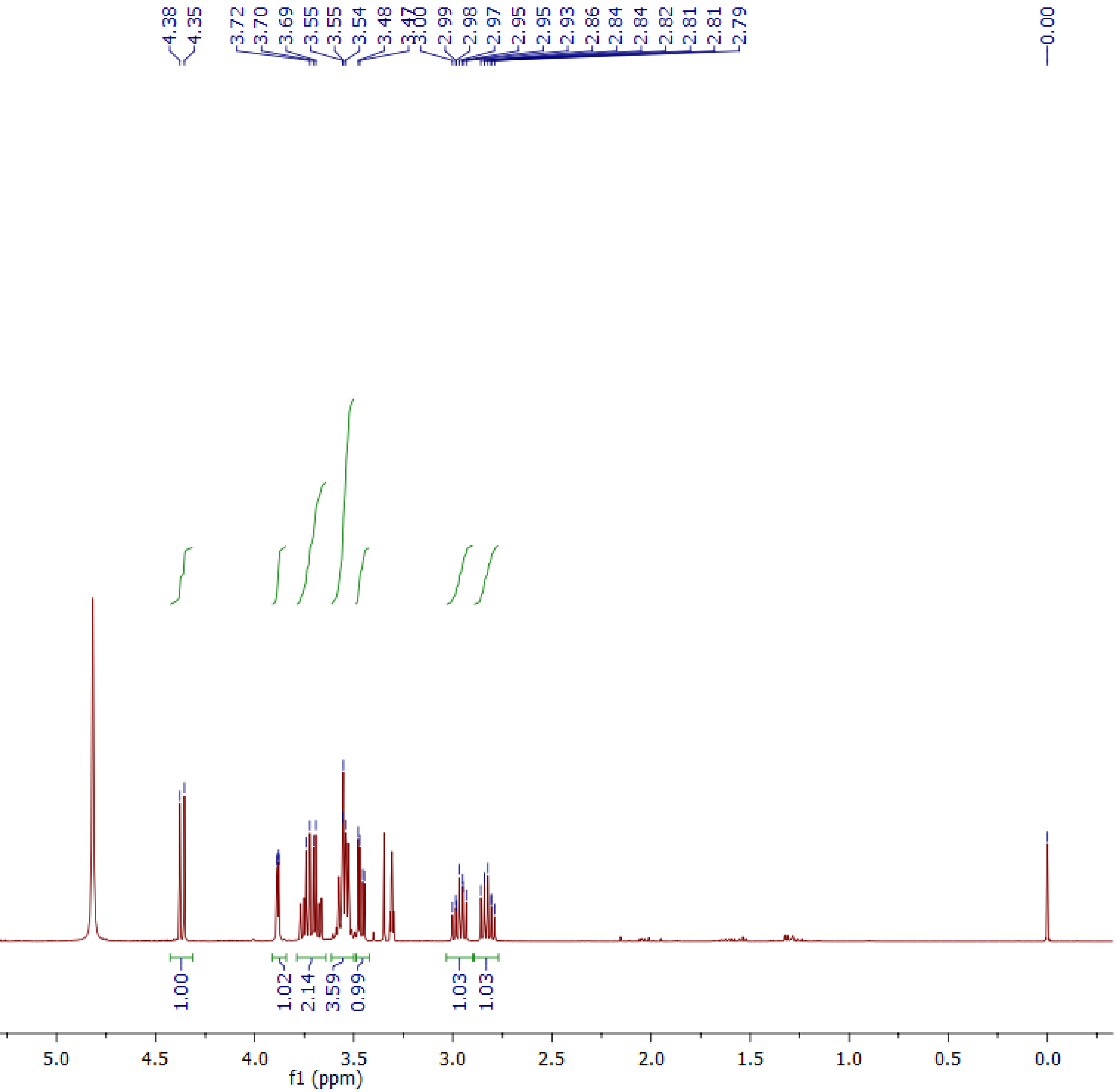

Fig. S28: ^{1}H NMR spectrum of compound **22** (400 MHz/CD_3OD/TMS; δ (ppm)).

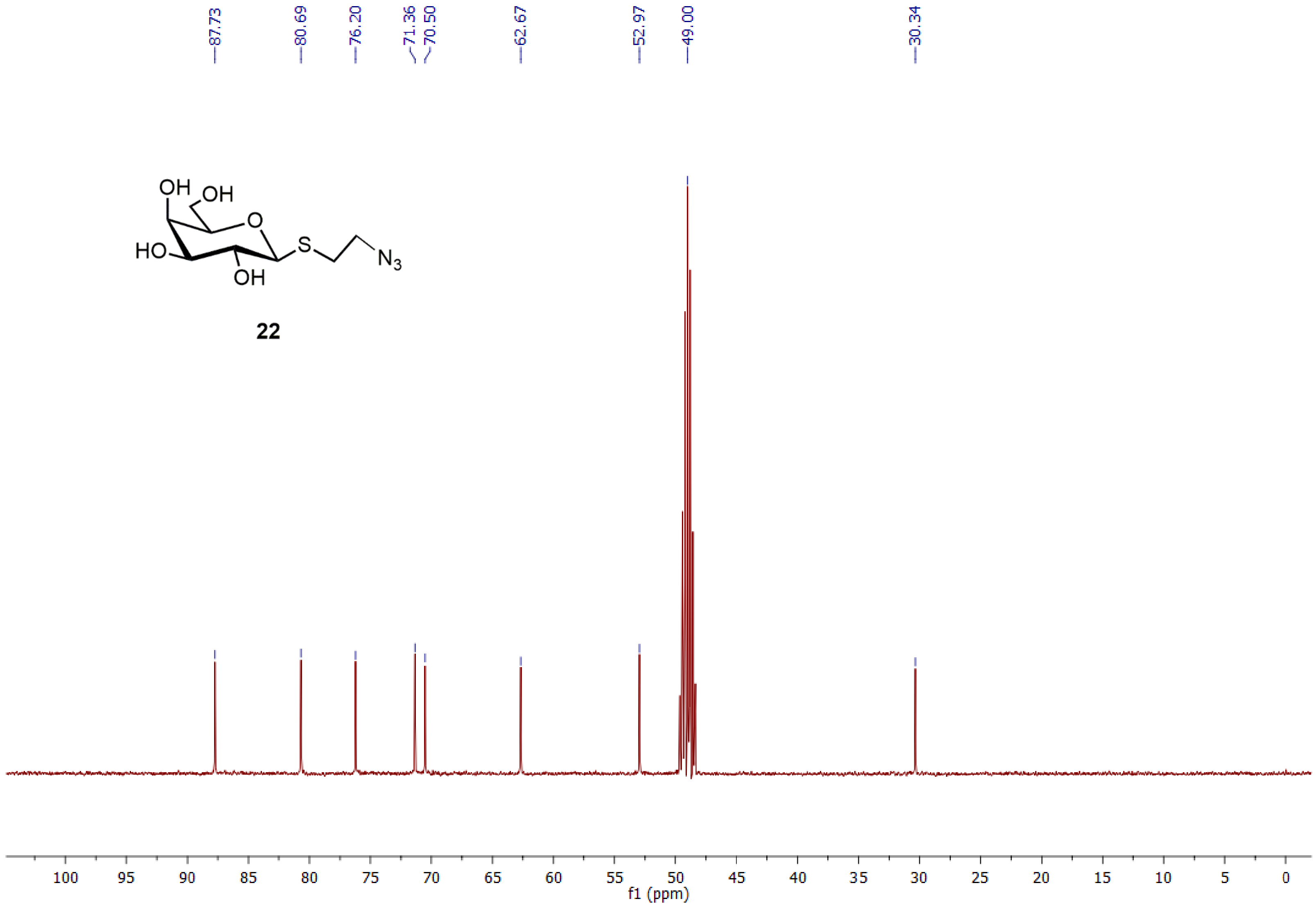

Fig. S29: ^{13}C NMR spectrum of compound **22** (100 MHz/CD_3OD/TMS; δ (ppm)).

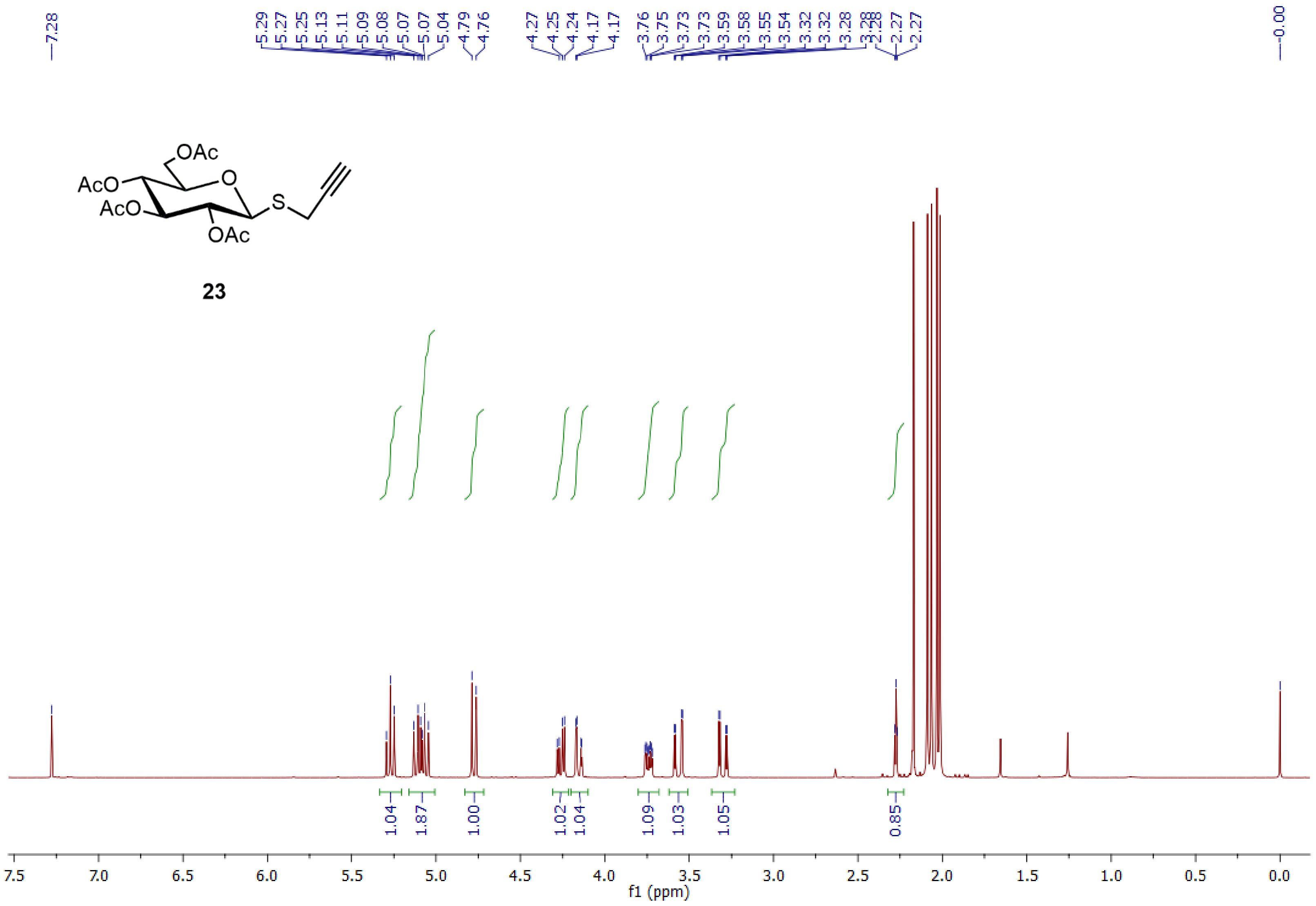

Fig. S30: ^{1}H NMR spectrum of compound **23** (400 MHz/$CDCl_3$/TMS; δ (ppm)).

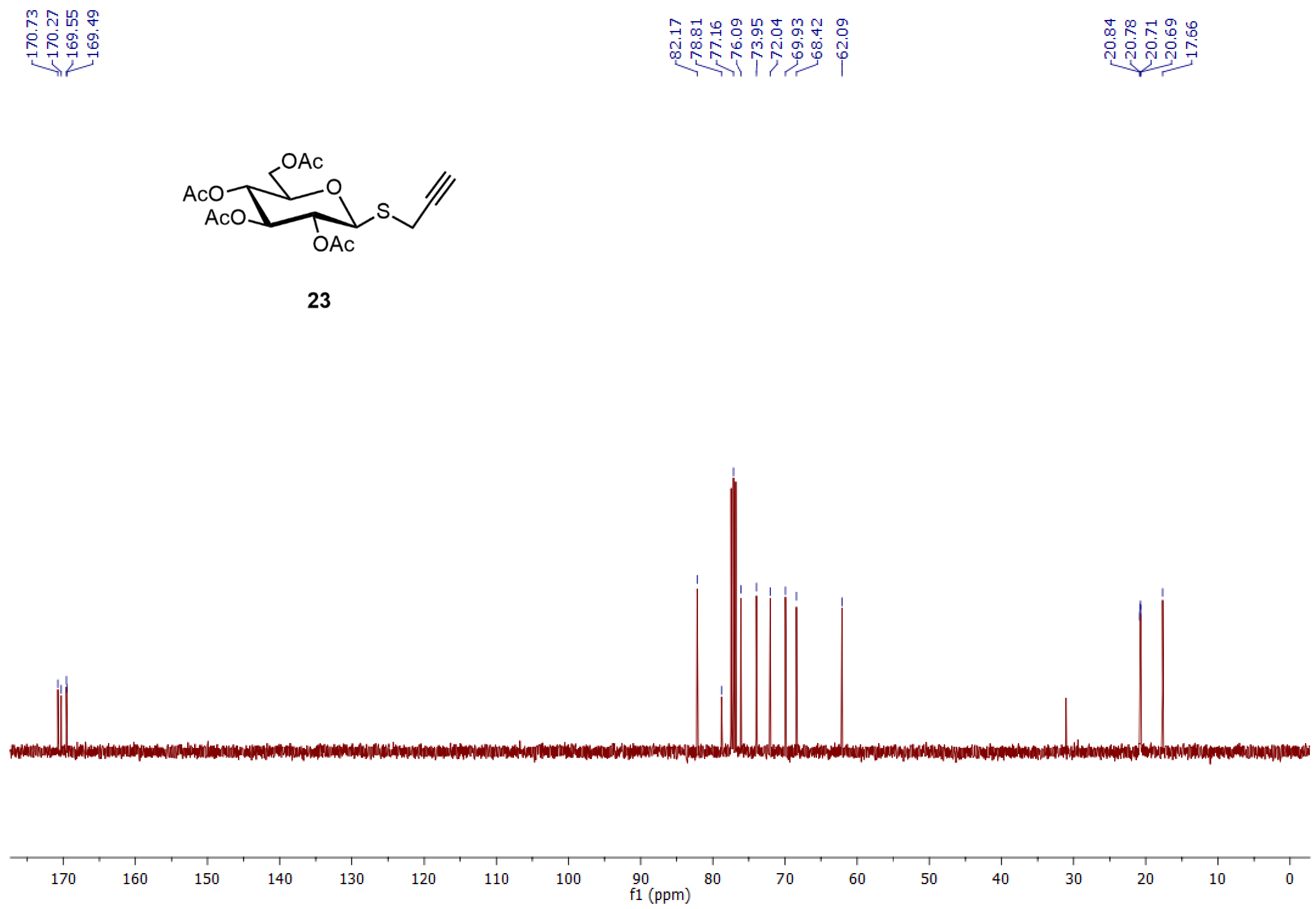

Fig. S31: ^{13}C NMR spectrum of compound **23** (100 MHz/$CDCl_3$/TMS; δ (ppm)).

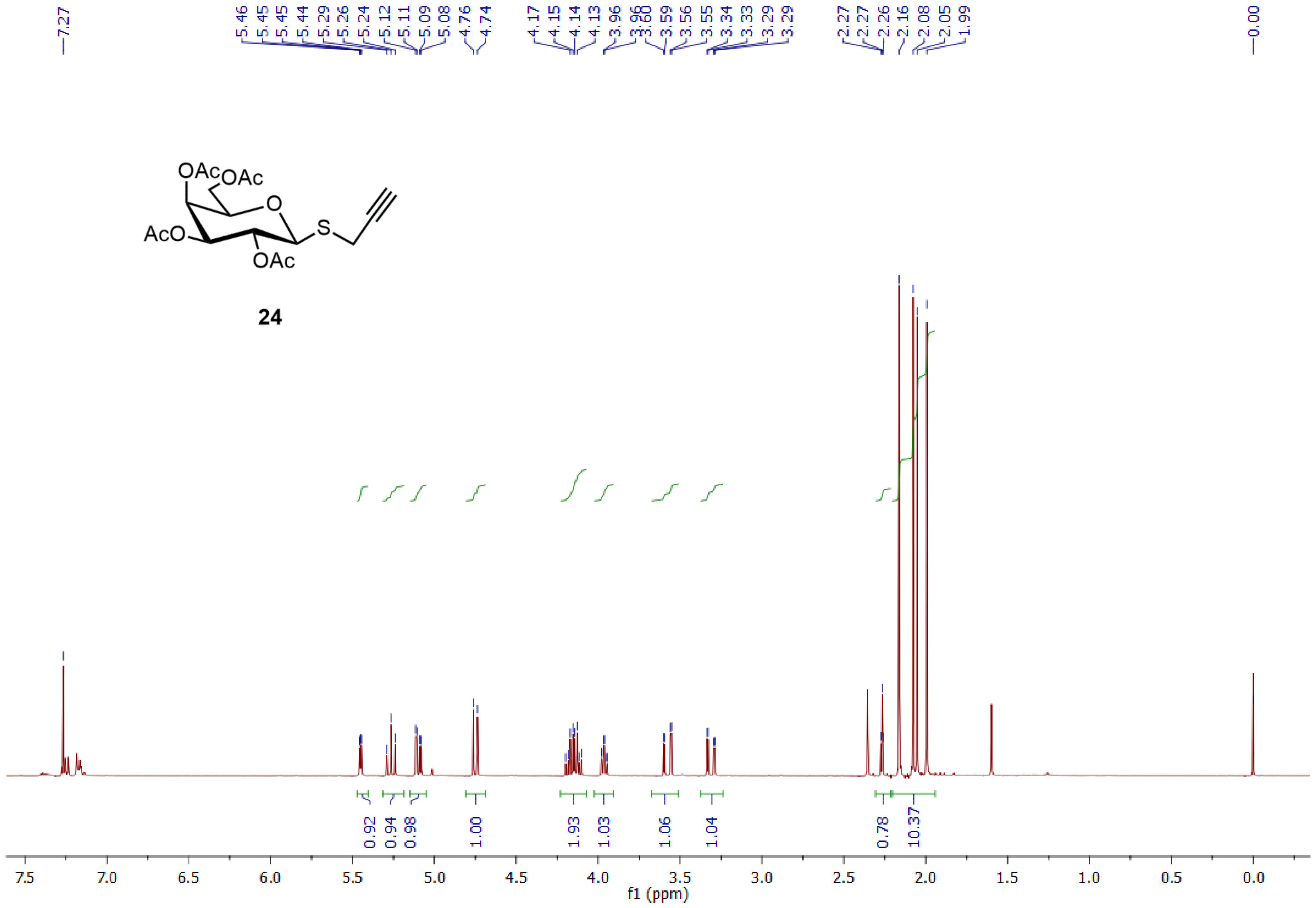

24

Fig. S32: ^{1}H NMR spectrum of compound **24** (400 MHz/$CDCl_3$/TMS; δ (ppm)).

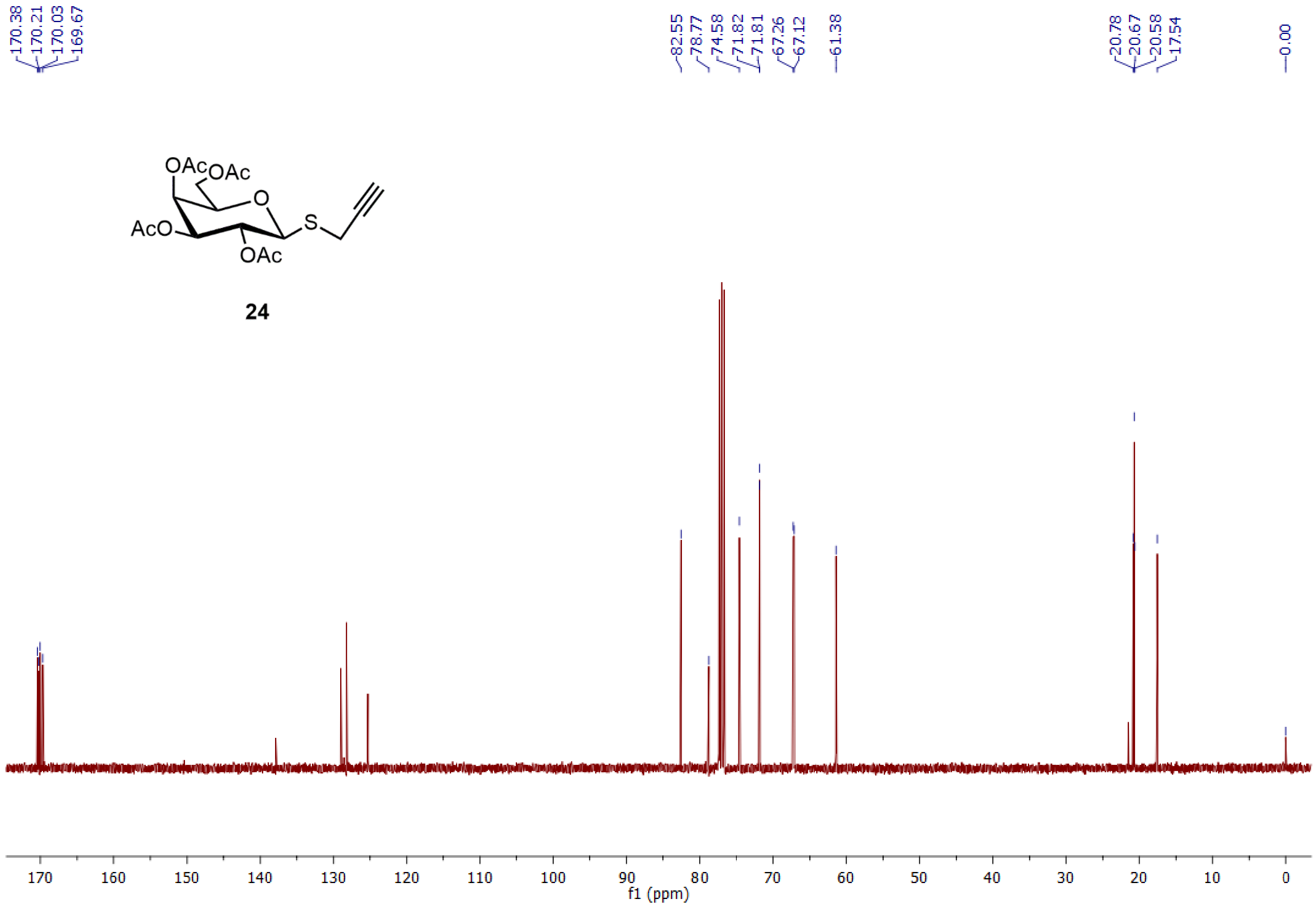

Fig. S33: ^{13}C NMR spectrum of compound **24** (100 MHz/$CDCl_3$/TMS; δ (ppm)).

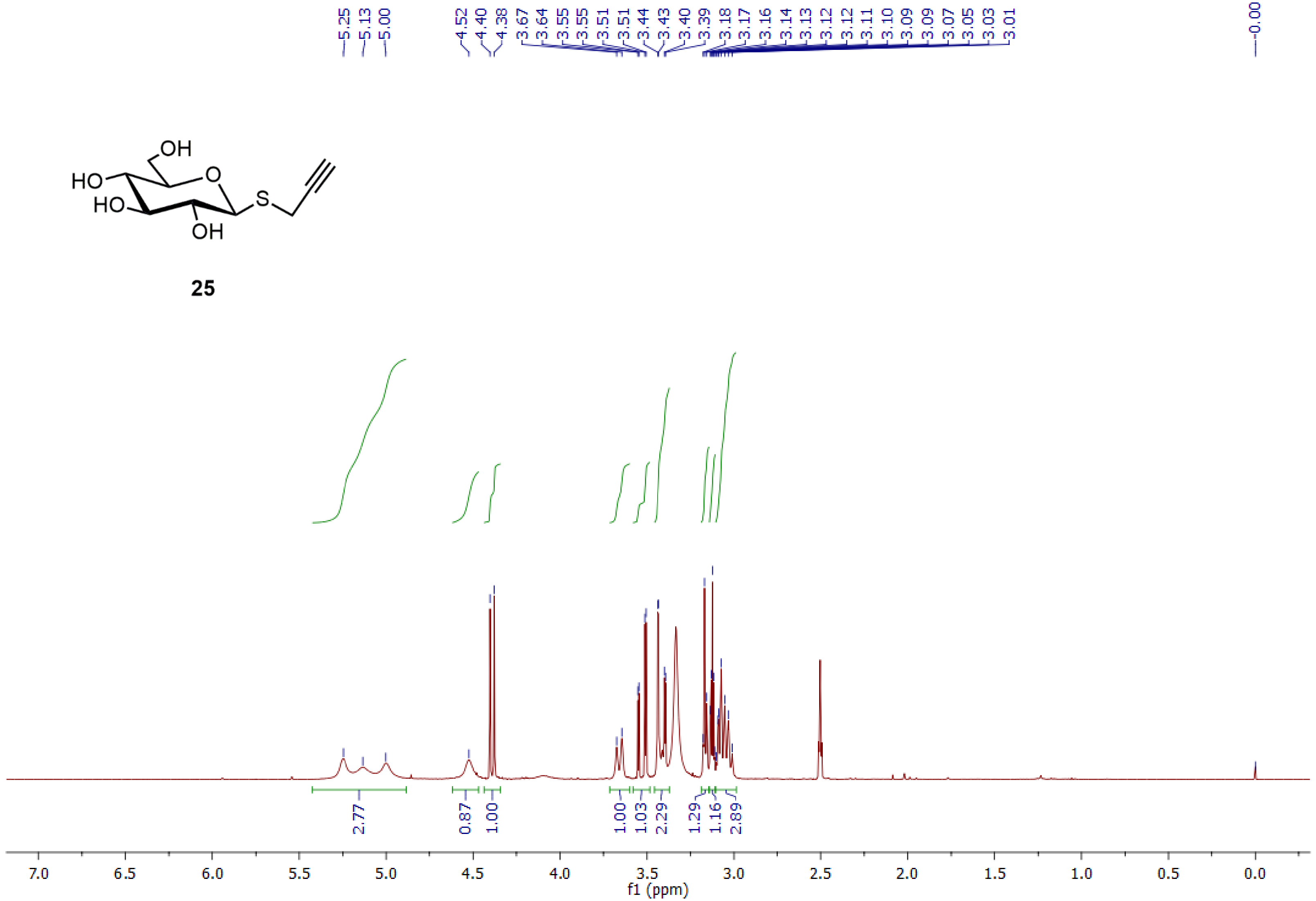

Fig. S34: ^{1}H NMR spectrum of compound **25** (400 MHz/DMSO/TMS; δ (ppm)).

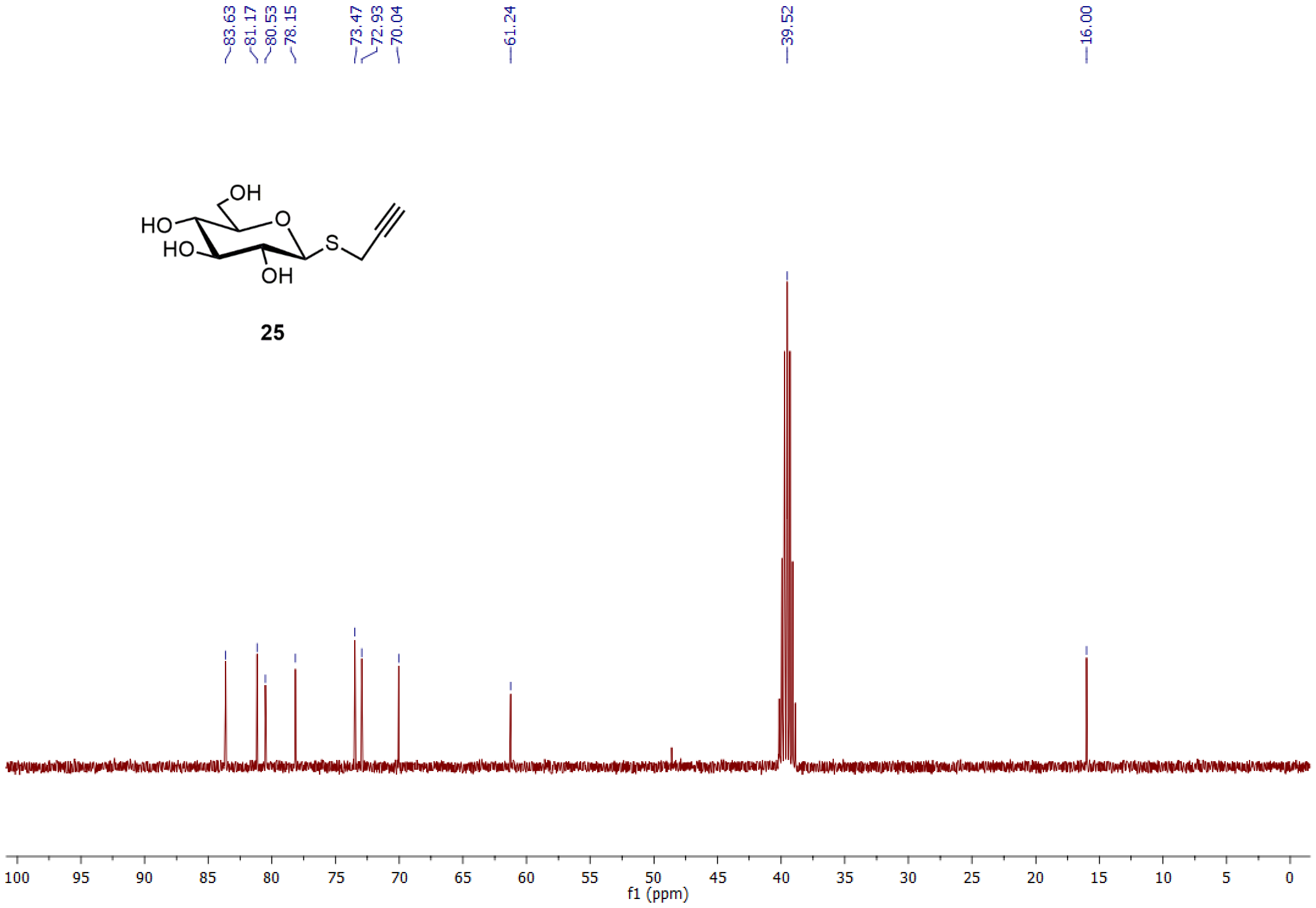

Fig. S35: ^{13}C NMR spectrum of compound **25** (100 MHz/DMSO/TMS; δ (ppm)).

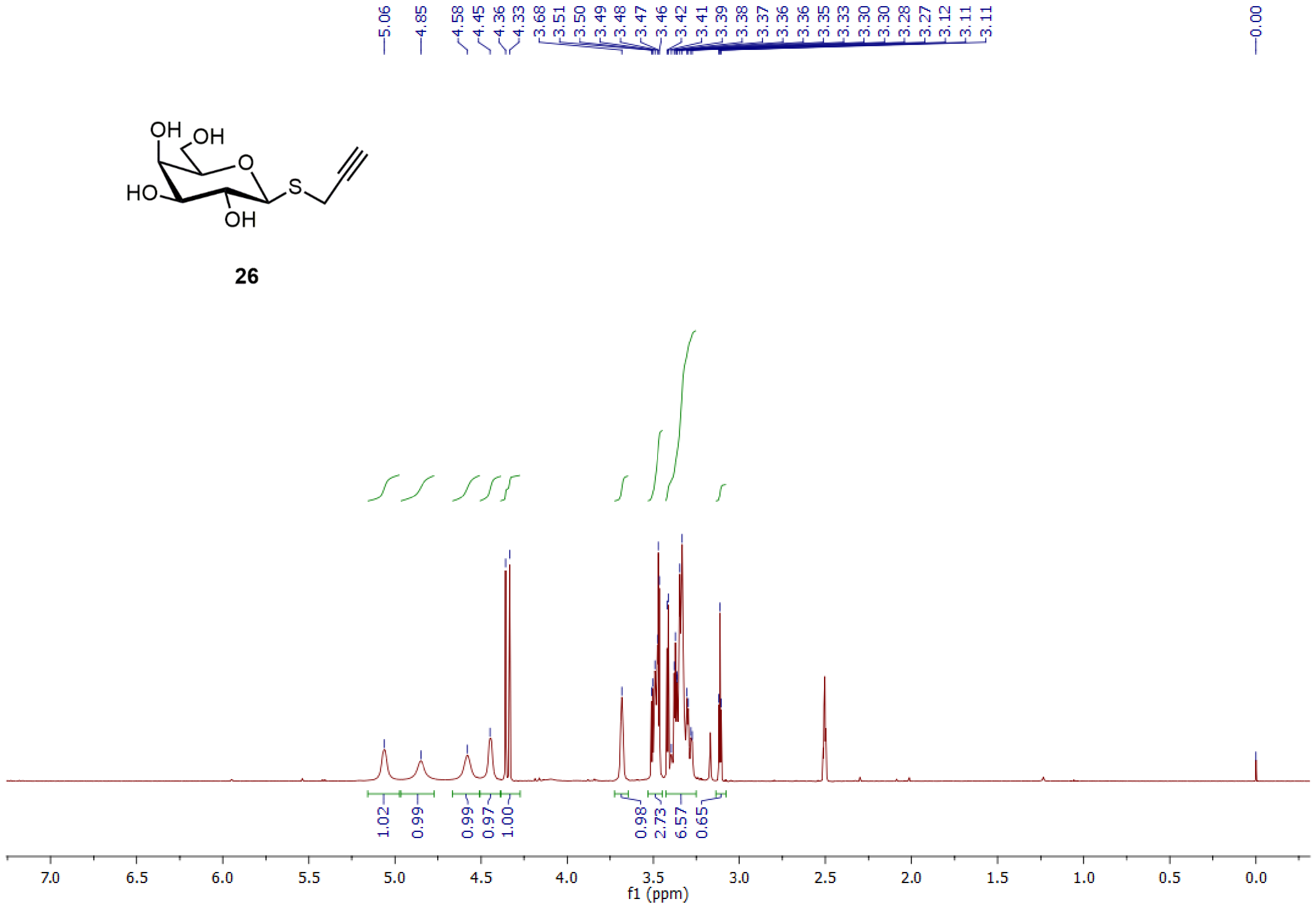

Fig. S36: ^{1}H NMR spectrum of compound **26** (400 MHz/DMSO/TMS; δ (ppm)).

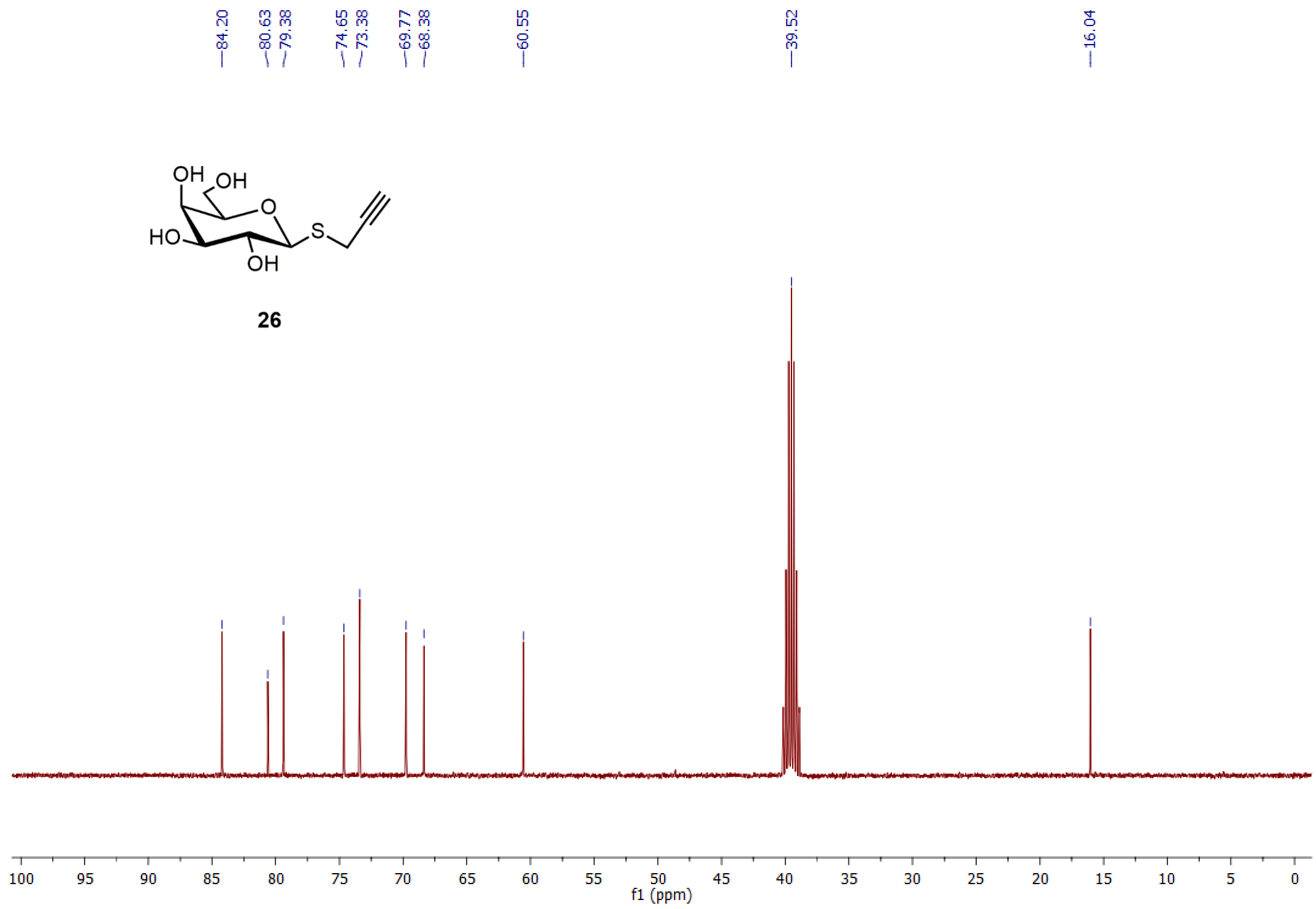

Fig. S37: ^{13}C NMR spectrum of compound **26** (100 MHz/DMSO/TMS; δ (ppm)).

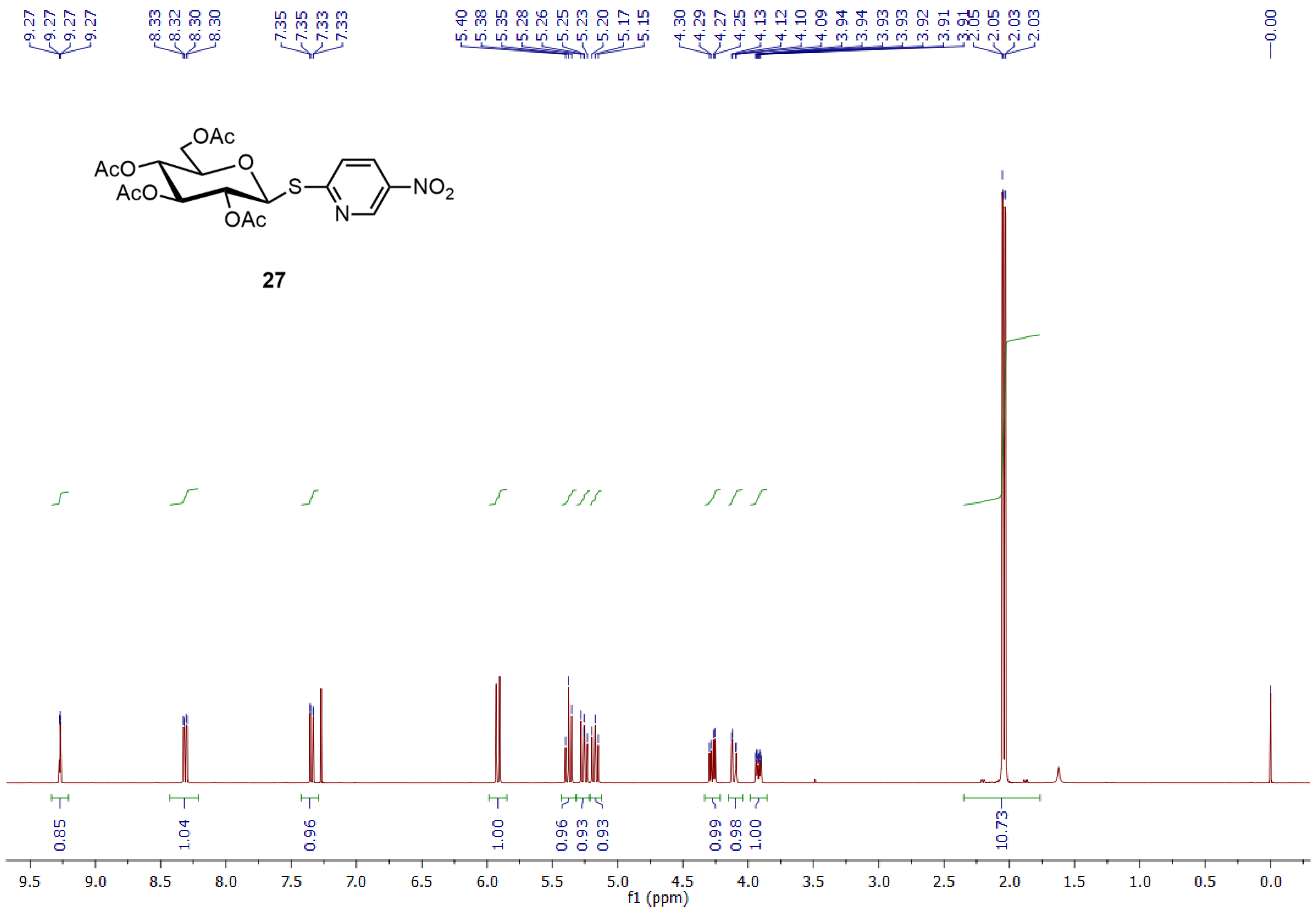

Fig. S38: ^{1}H NMR spectrum of compound **27** (400 MHz/$CDCl_3$/TMS; δ (ppm)).

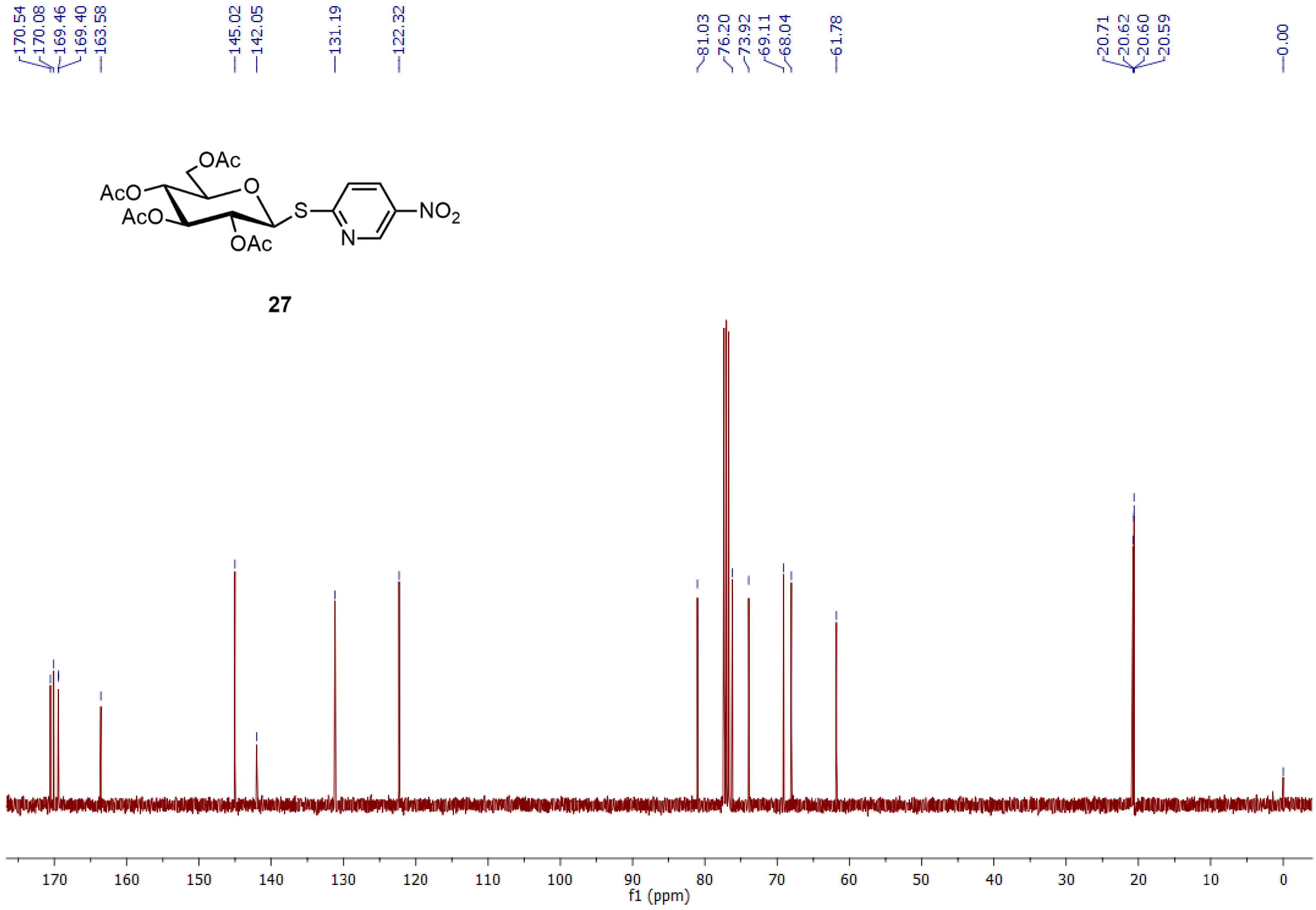

Fig. S39: ^{13}C NMR spectrum of compound **27** (100 MHz/$CDCl_3$/TMS; δ (ppm)).

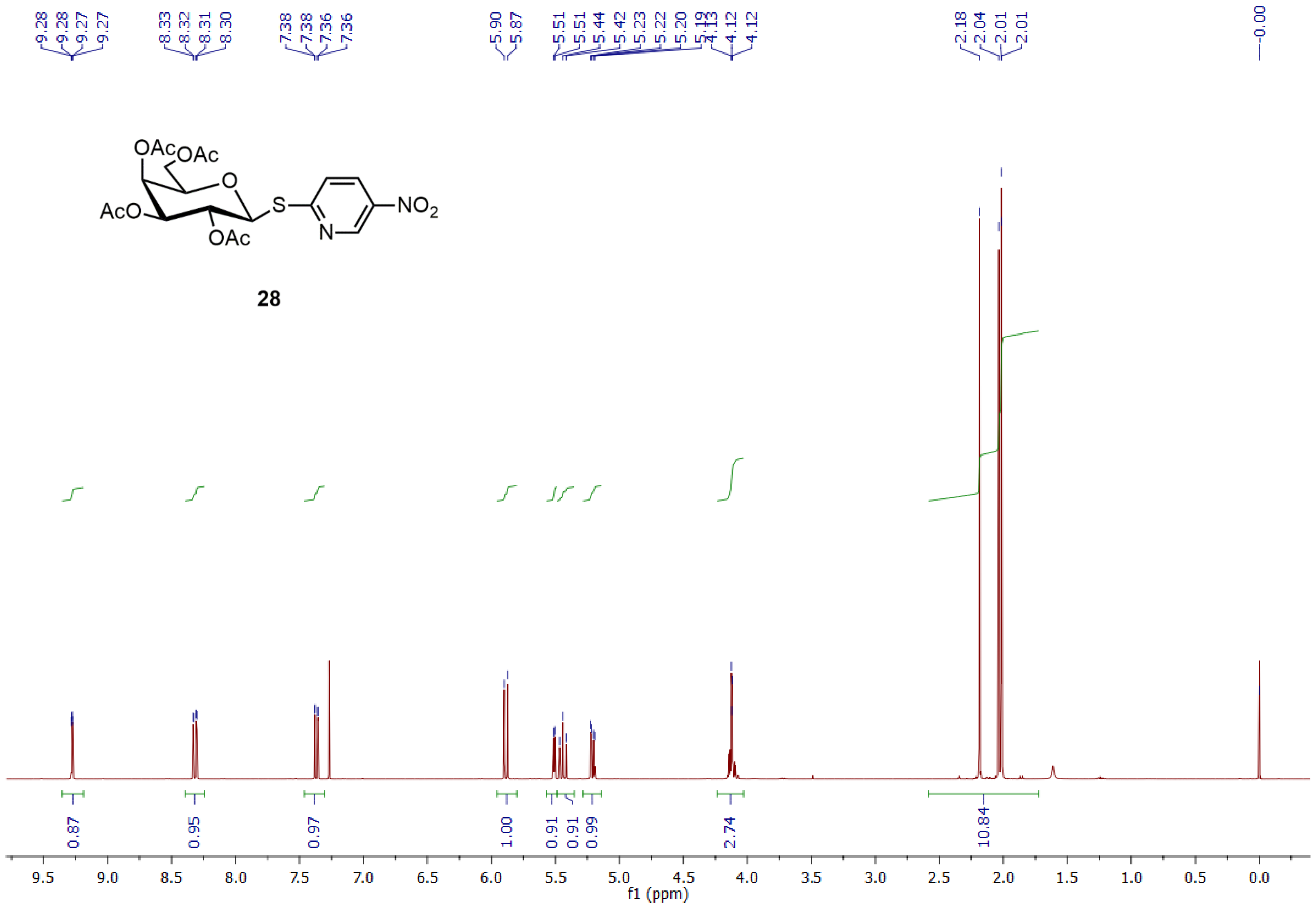

Fig. S40: ^{1}H NMR spectrum of compound **28** (400 MHz/$CDCl_3$/TMS; δ (ppm)).

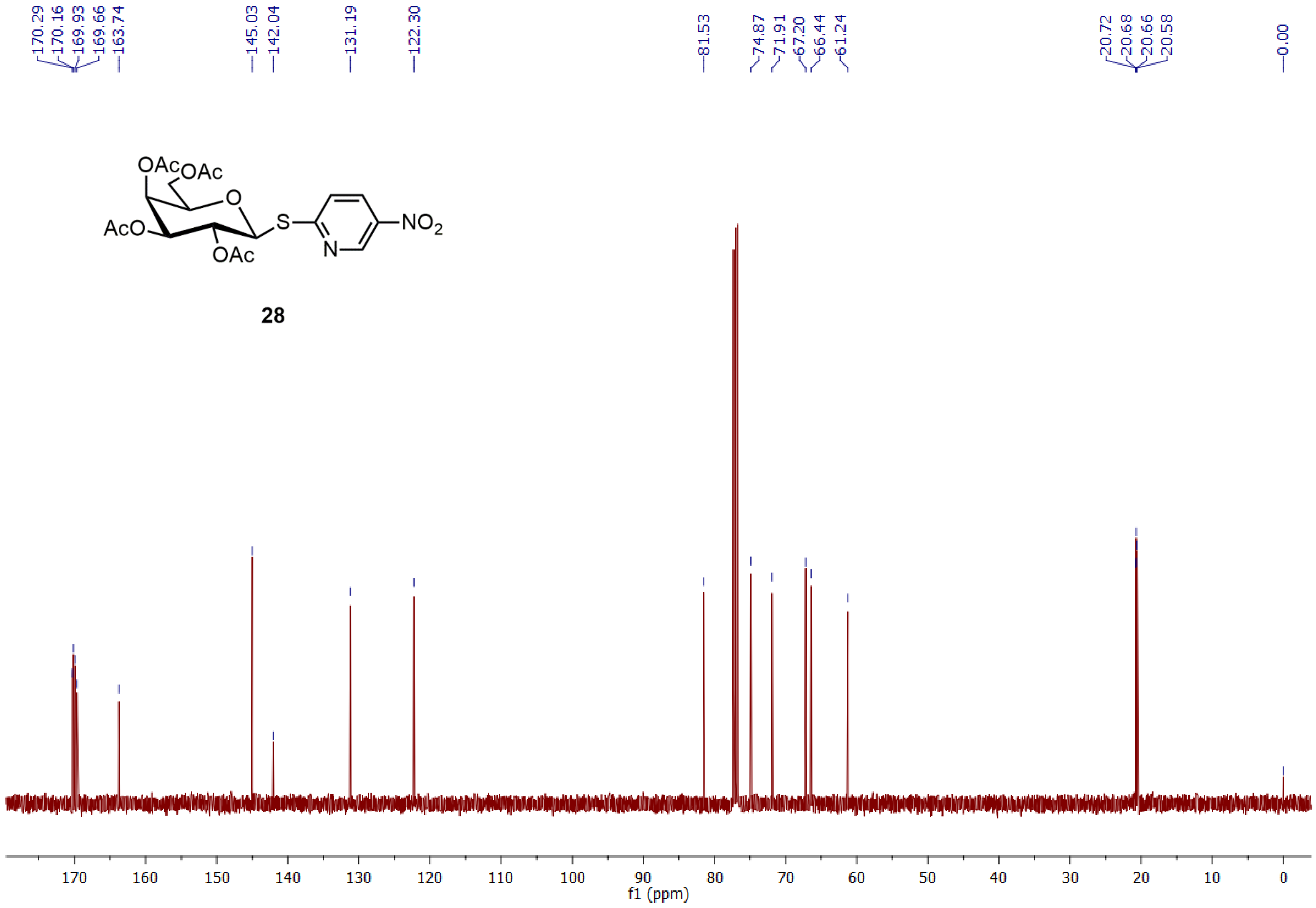

Fig. S41: ^{13}C NMR spectrum of compound **28** (100 MHz/$CDCl_3$/TMS; δ (ppm)).

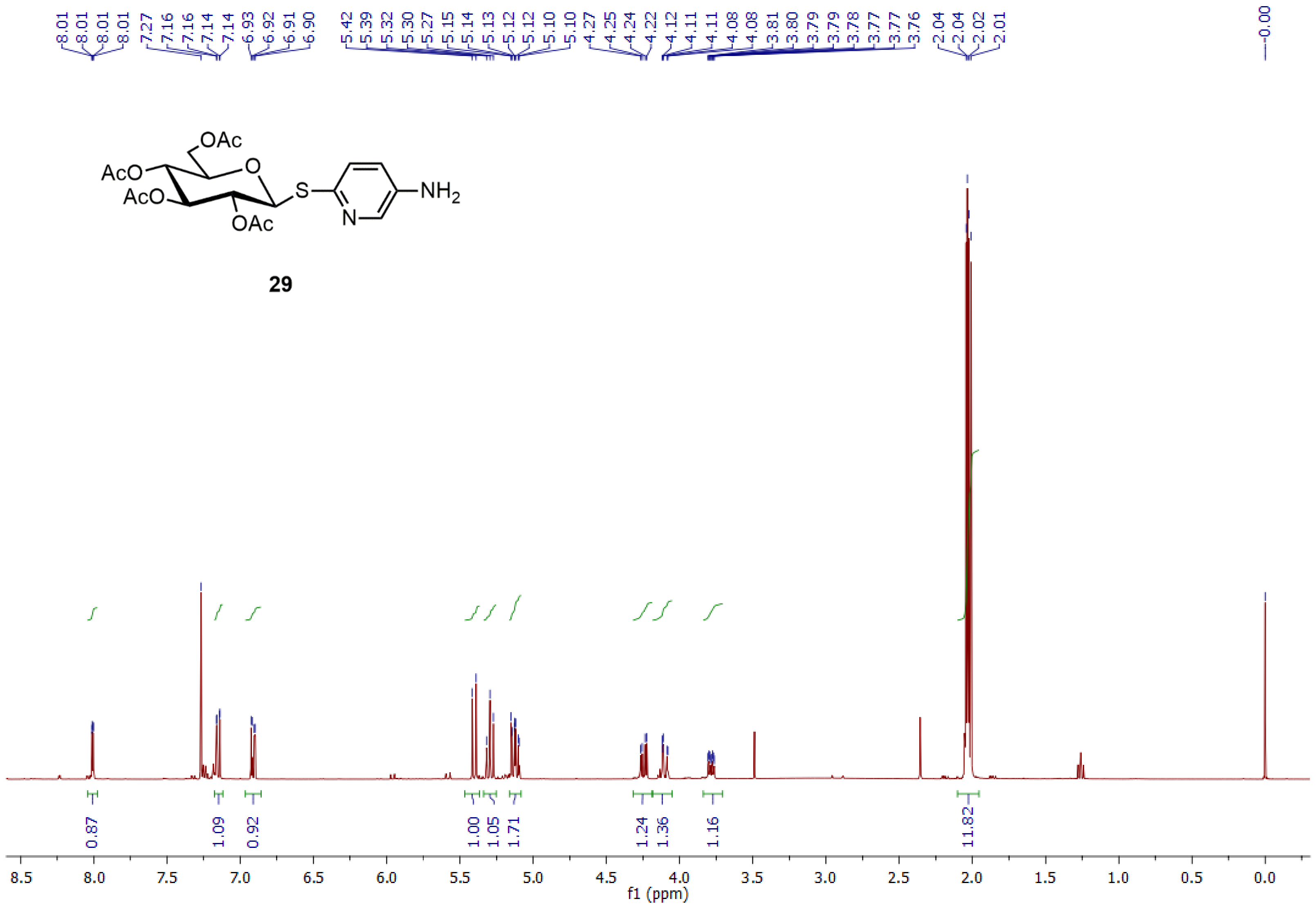

Fig. S42: ^{1}H NMR spectrum of compound **29** (400 MHz/$CDCl_3$/TMS; δ (ppm)).

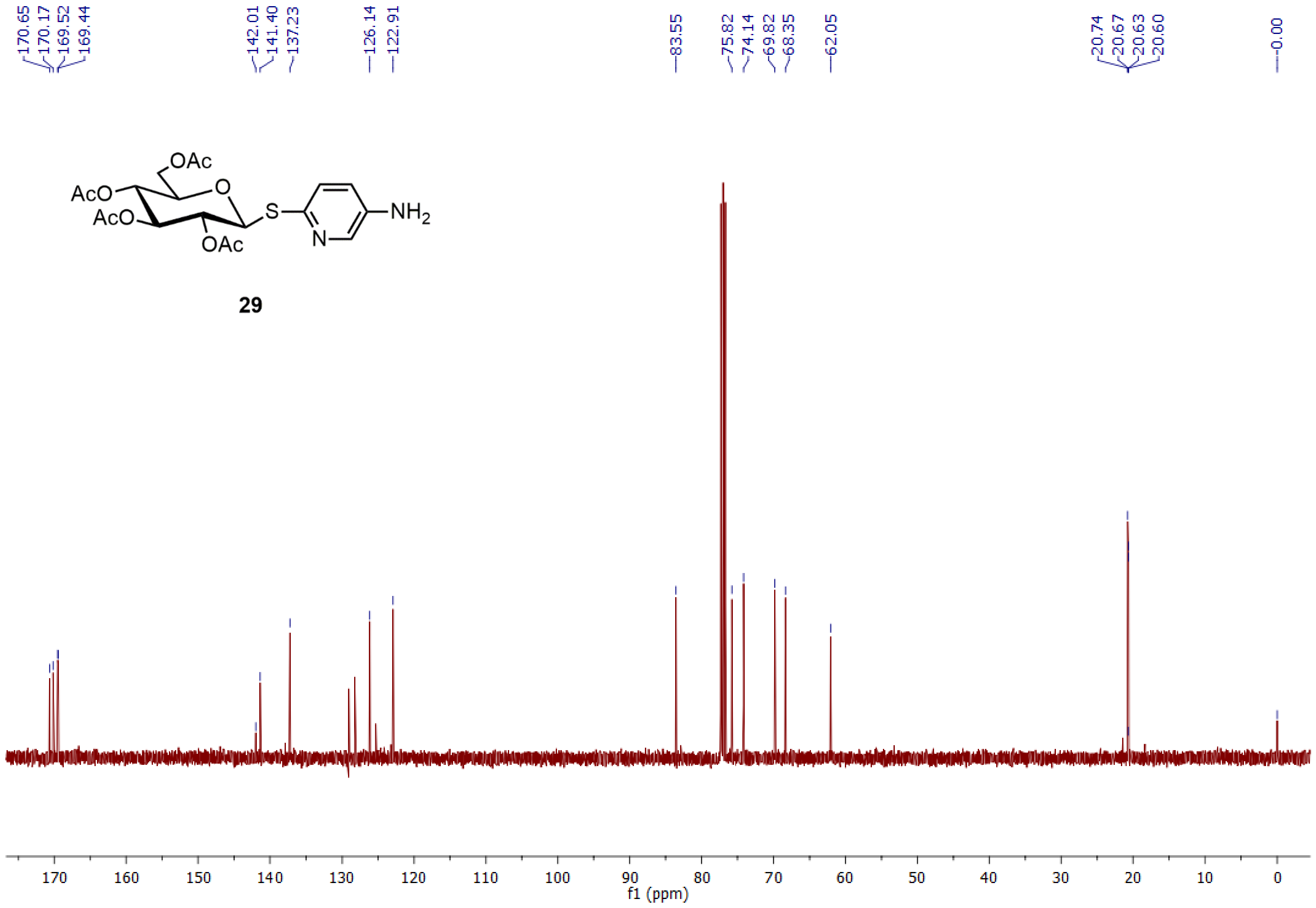

Fig. S43: ^{13}C NMR spectrum of compound **29** (100 MHz/$CDCl_3$/TMS; δ (ppm)).

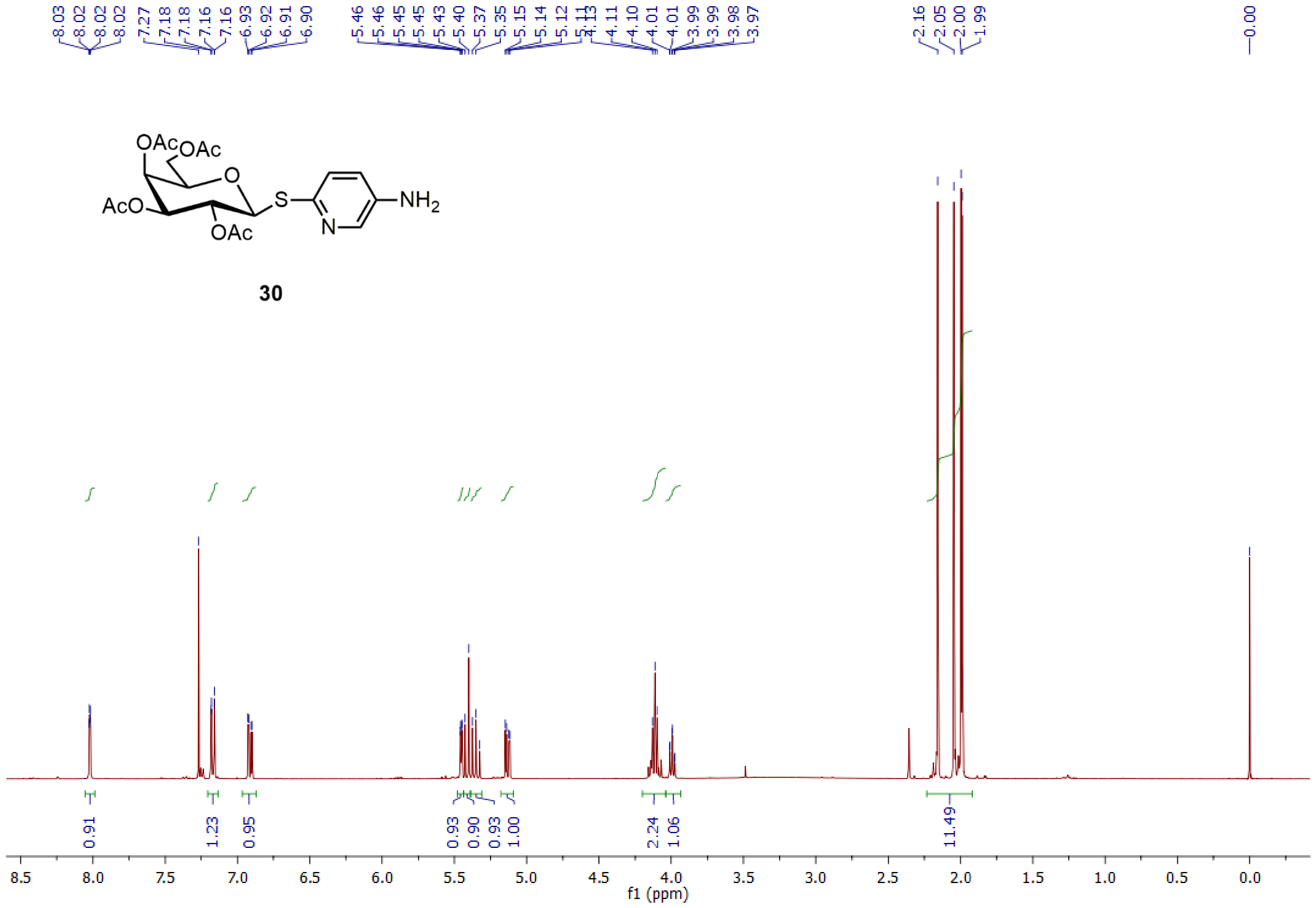

Fig. S44: ^{1}H NMR spectrum of compound **30** (400 MHz/$CDCl_3$/TMS; δ (ppm)).

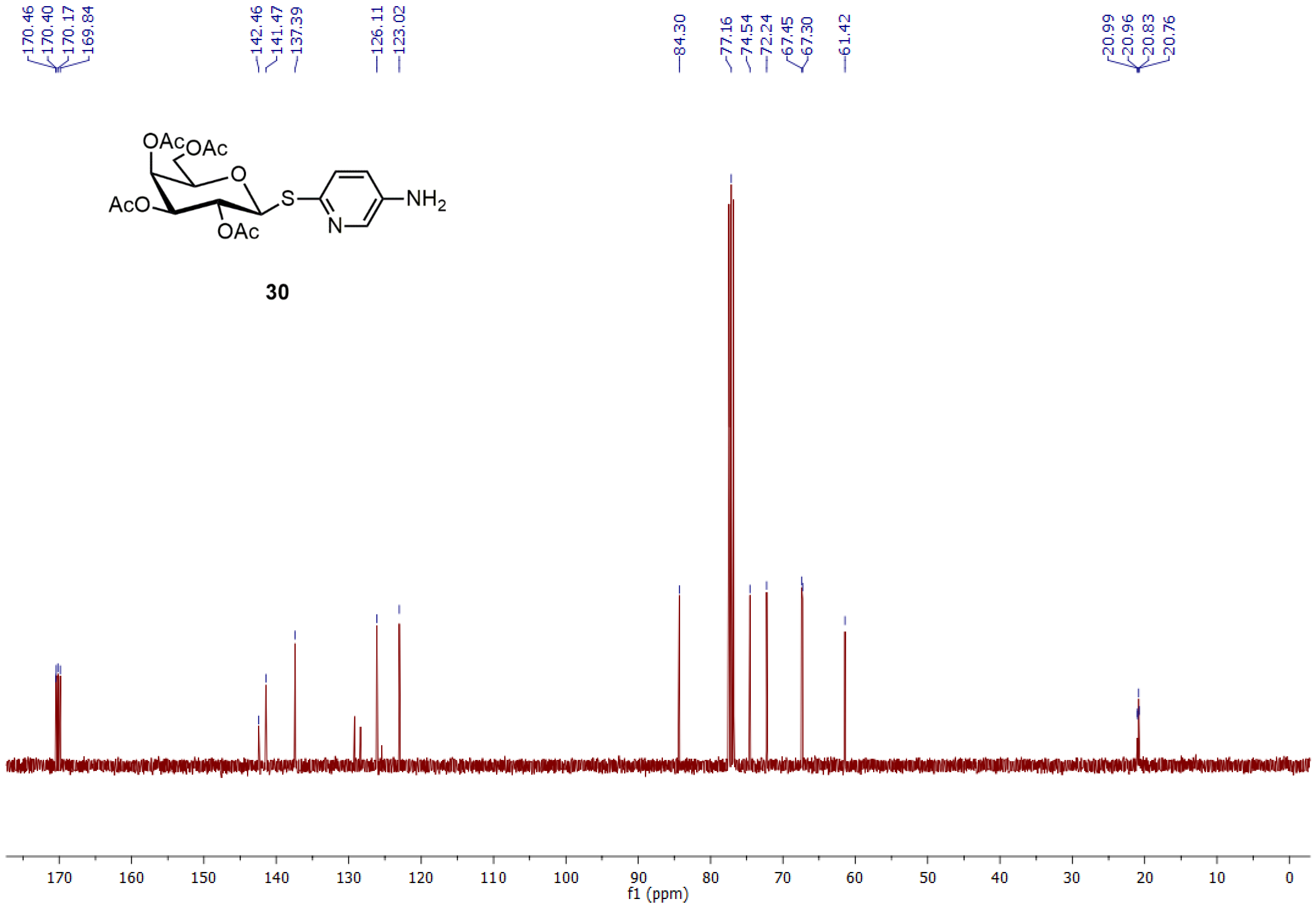

Fig. S45: ^{13}C NMR spectrum of compound **30** (100 MHz/$CDCl_3$/TMS; δ (ppm)).

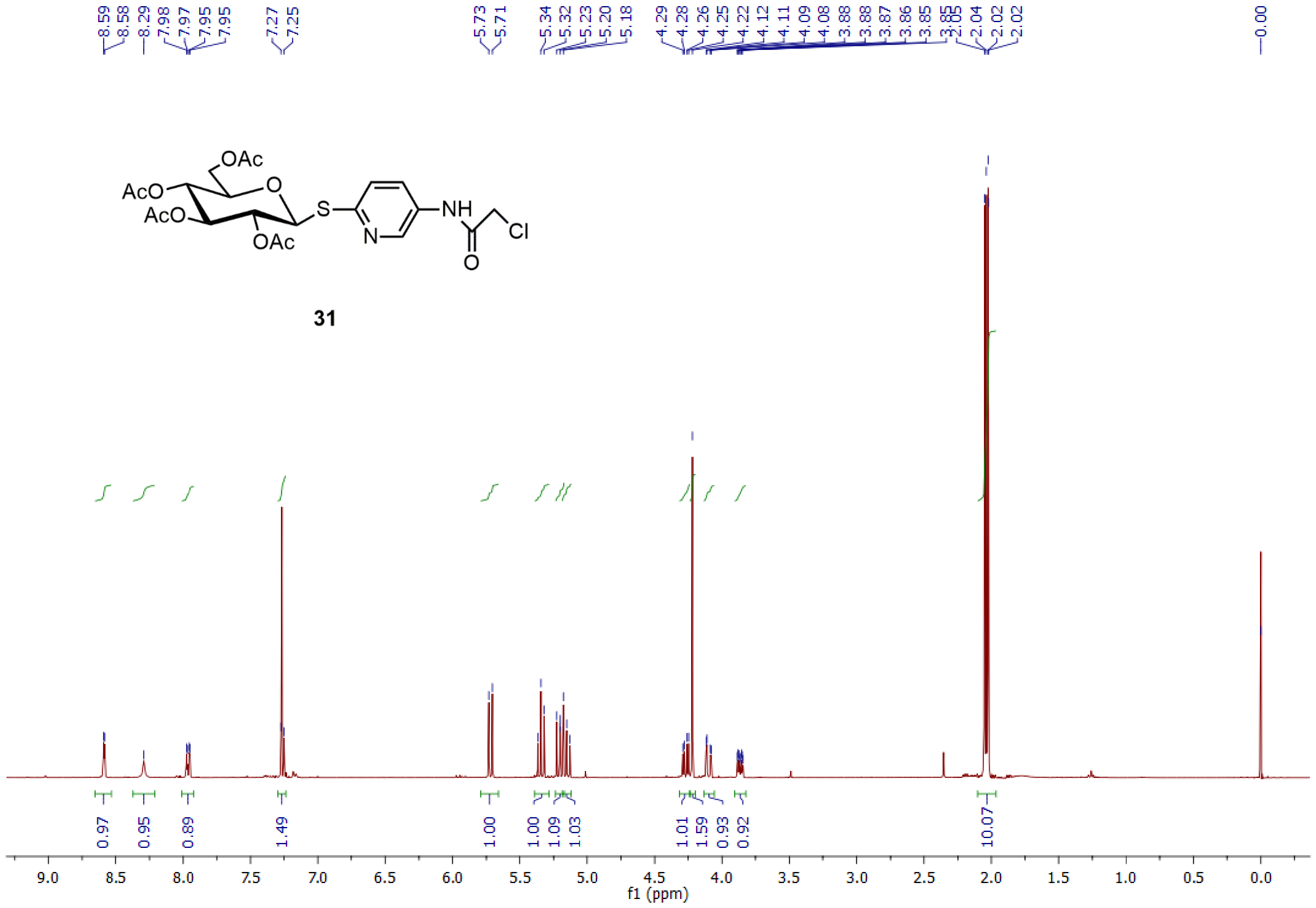

Fig. S46: ^{1}H NMR spectrum of compound **31** (400 MHz/$CDCl_3$/TMS; δ (ppm)).

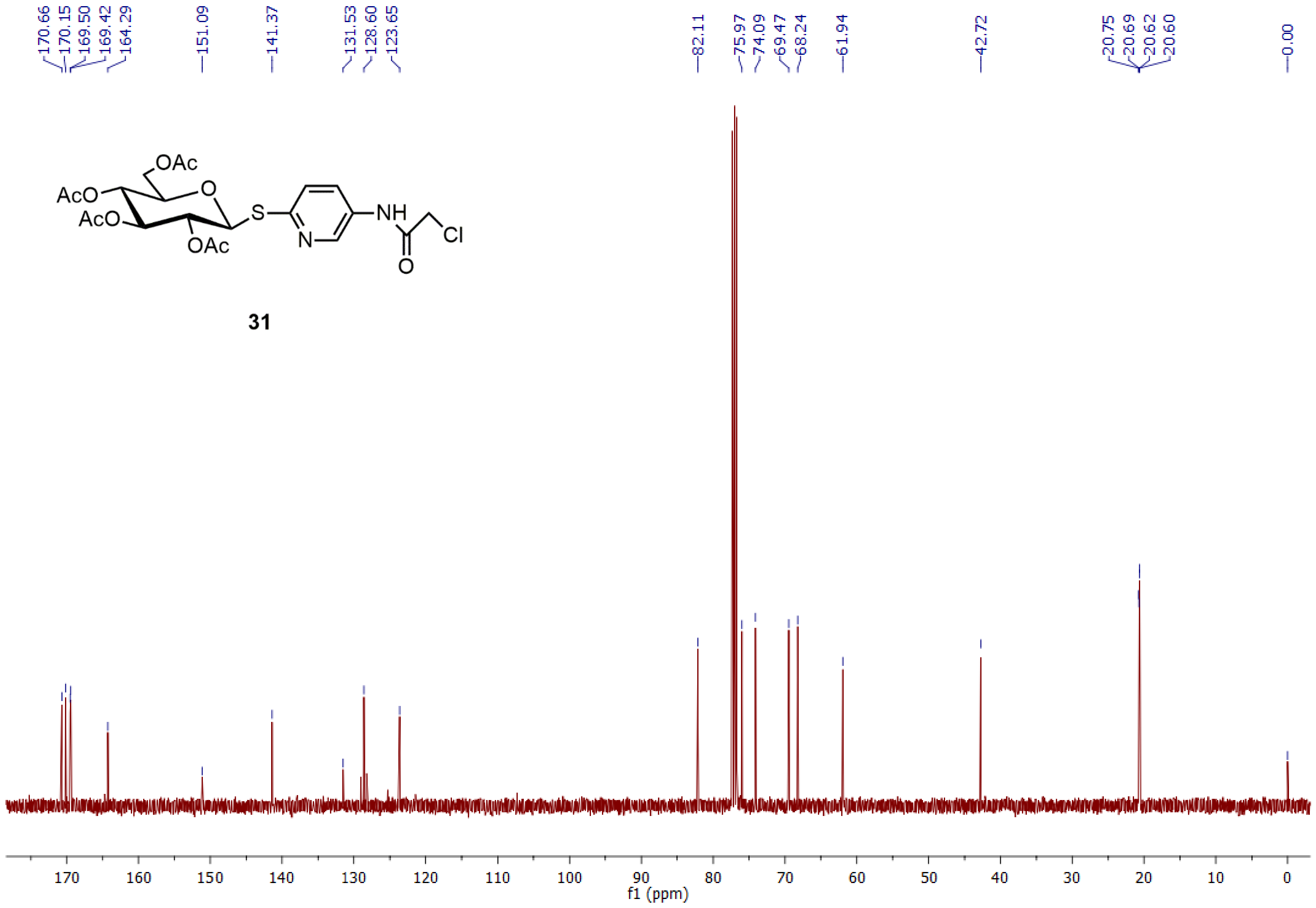

Fig. S47: ^{13}C NMR spectrum of compound **31** (100 MHz/$CDCl_3$/TMS; δ (ppm)).

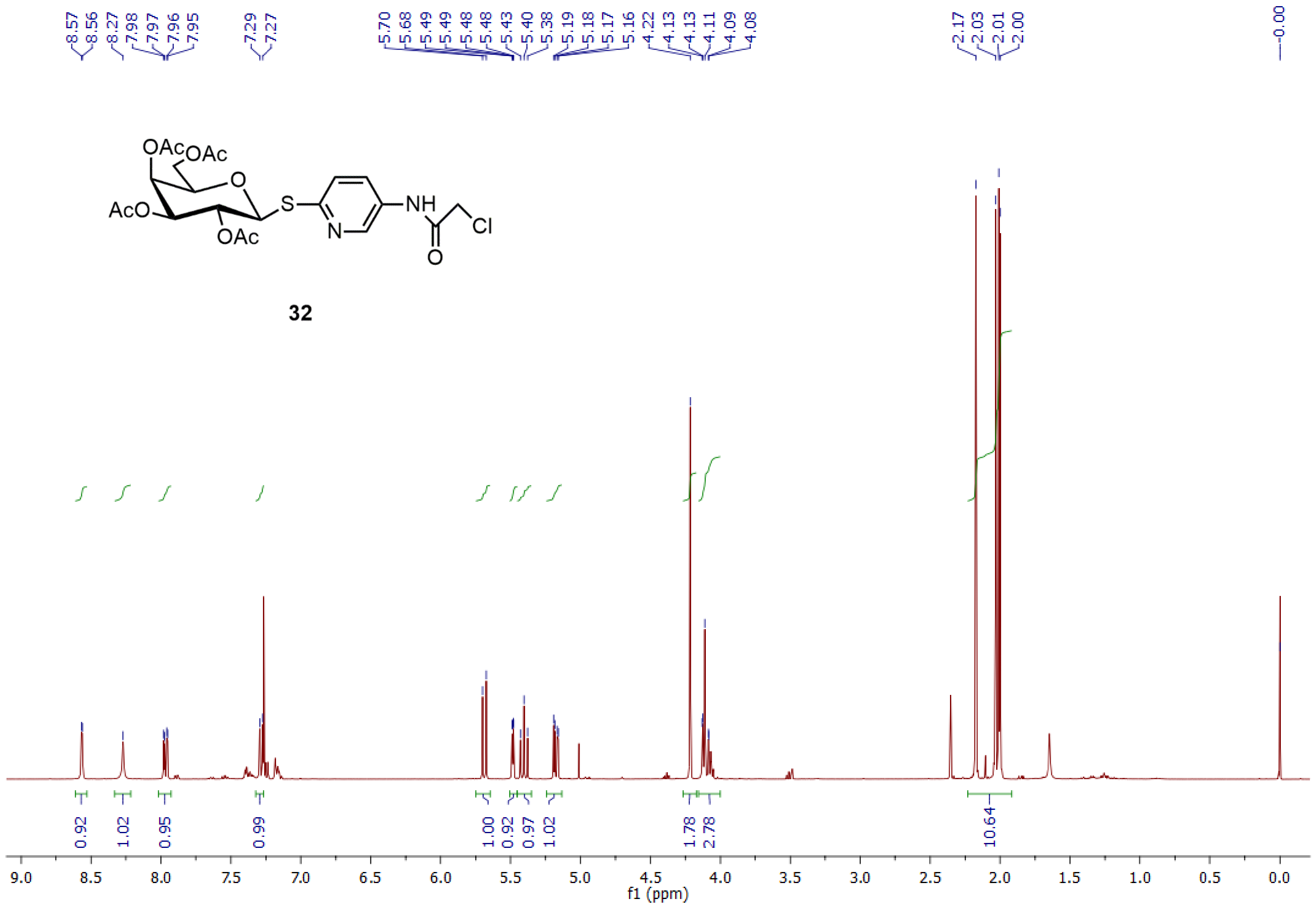

Fig. S48: ^{1}H NMR spectrum of compound **32** (400 MHz/$CDCl_3$/TMS; δ (ppm)).

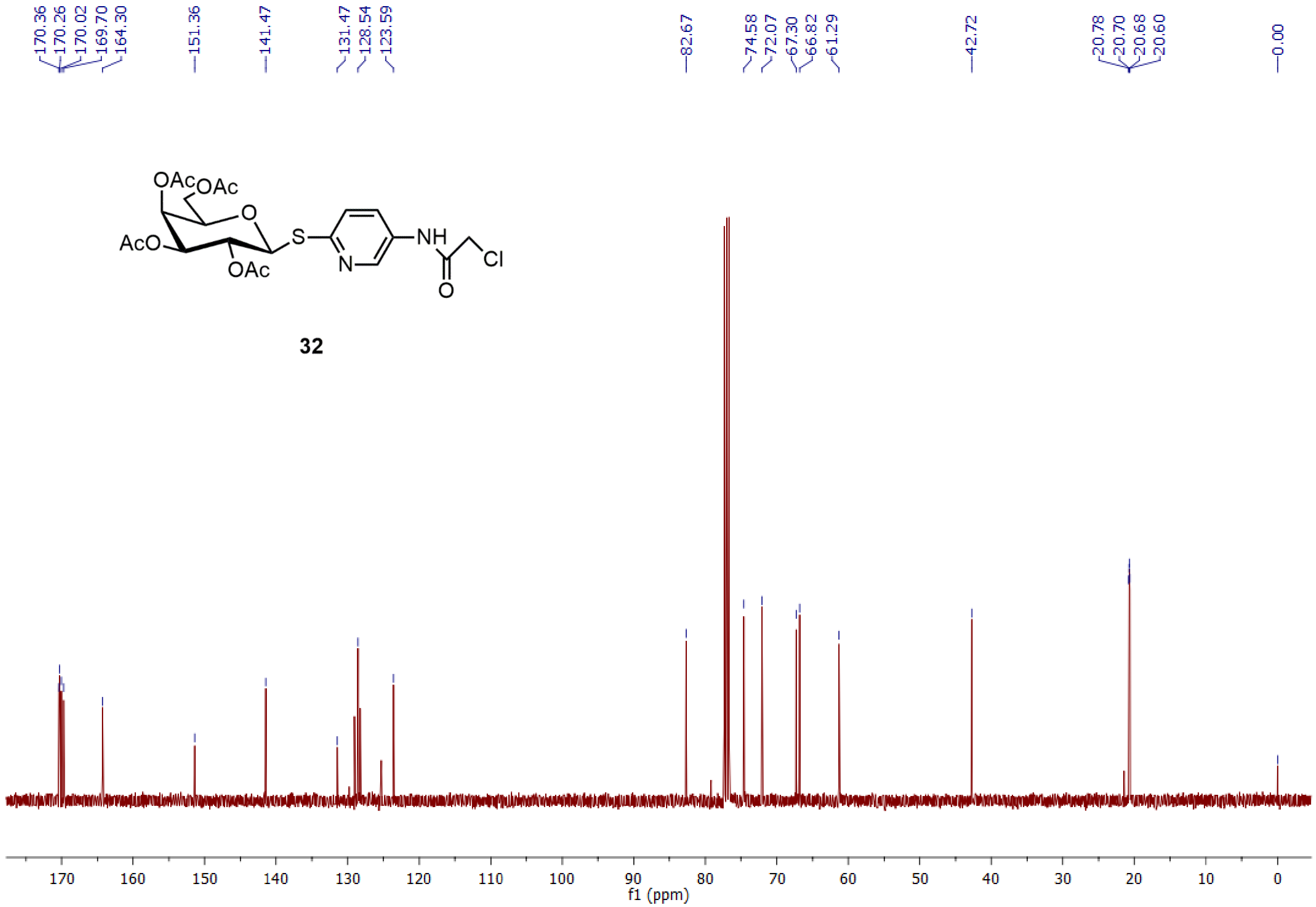

Fig. S49: ^{13}C NMR spectrum of compound **32** (100 MHz/$CDCl_3$/TMS; δ (ppm)).

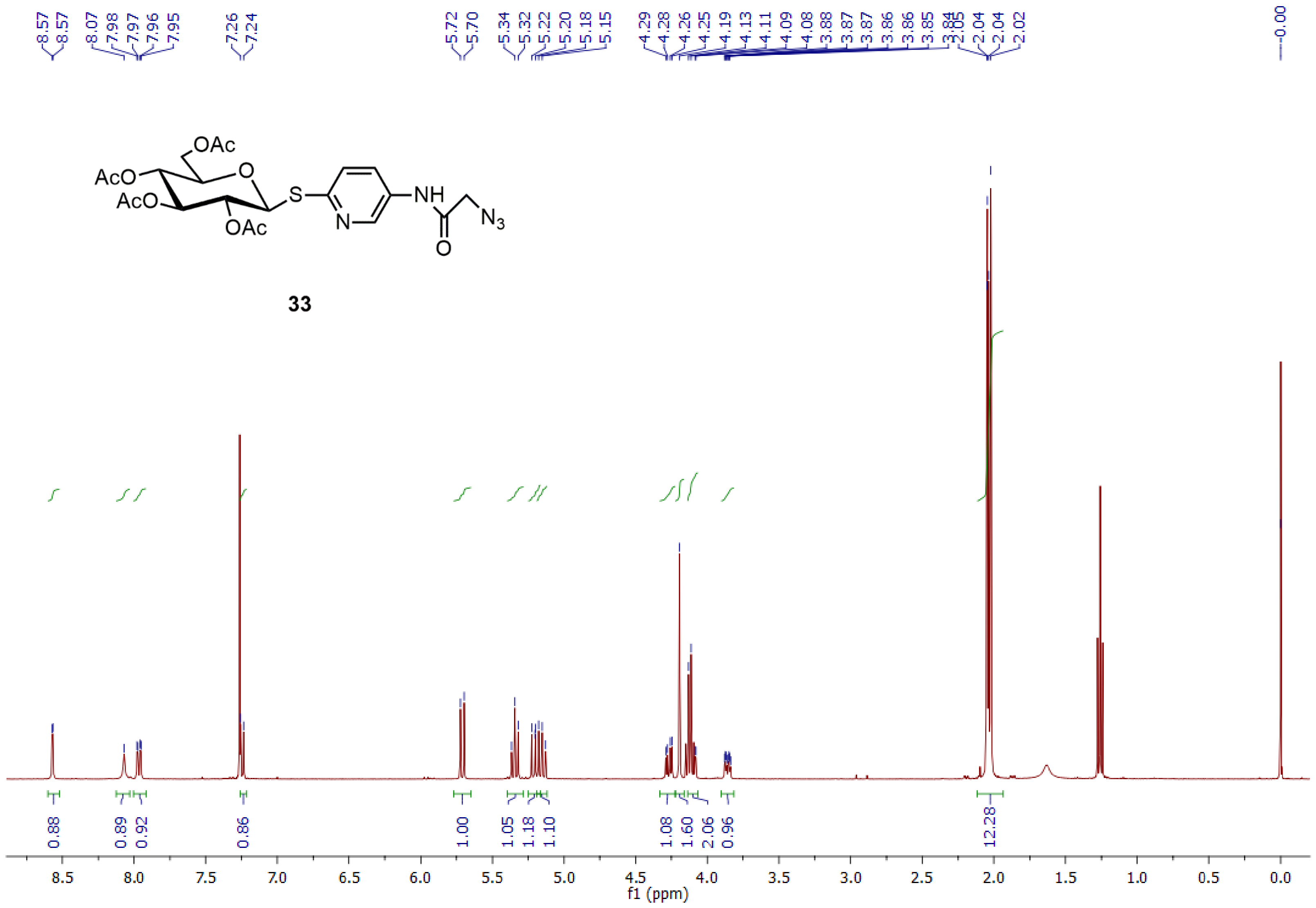

Fig. S50: ^{1}H NMR spectrum of compound **33** (400 MHz/$CDCl_3$/TMS; δ (ppm)).

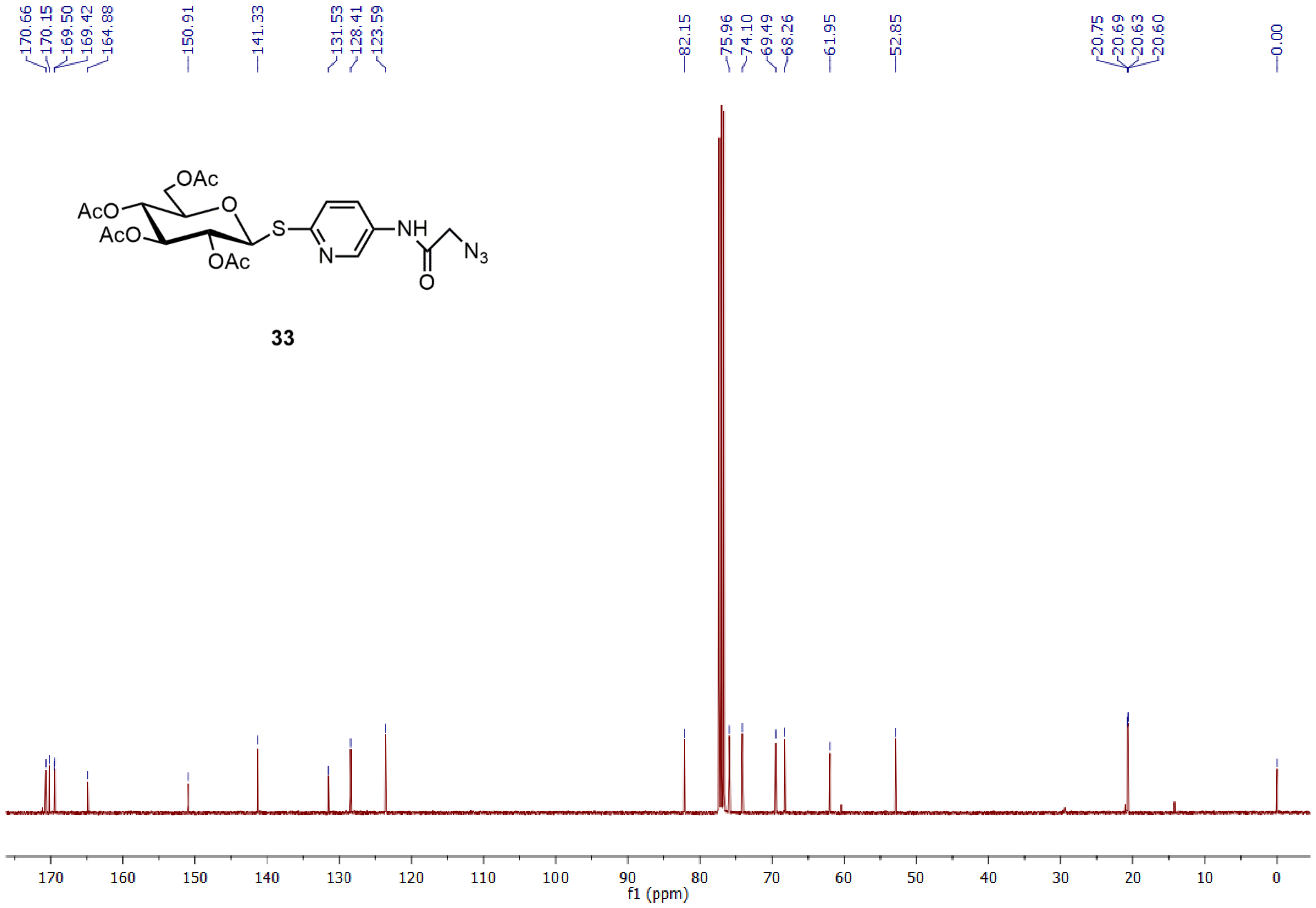

Fig. S51: ^{13}C NMR spectrum of compound **33** (100 MHz/$CDCl_3$/TMS; δ (ppm)).

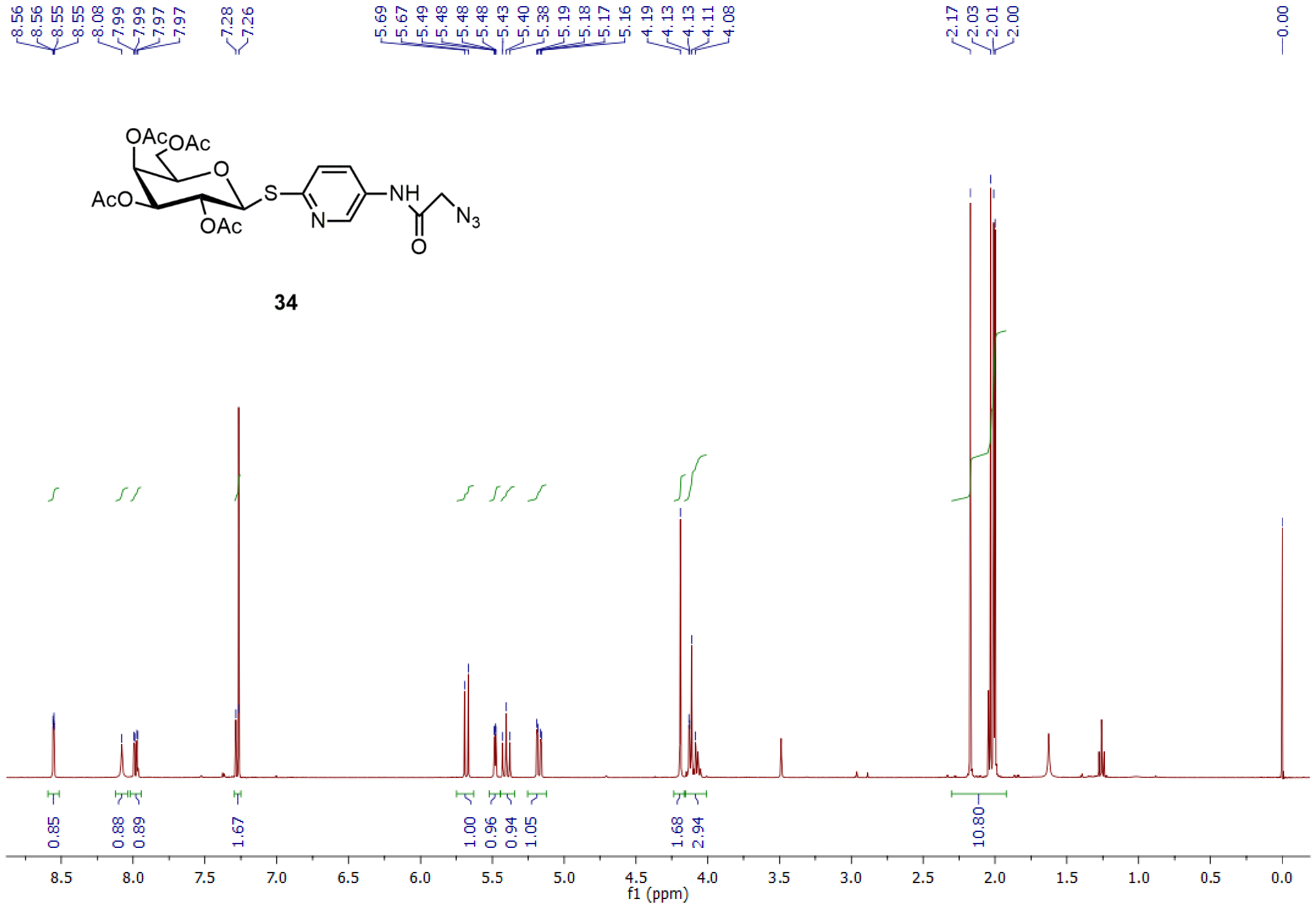

Fig. S52: ^{1}H NMR spectrum of compound **34** (400 MHz/$CDCl_3$/TMS; δ (ppm)).

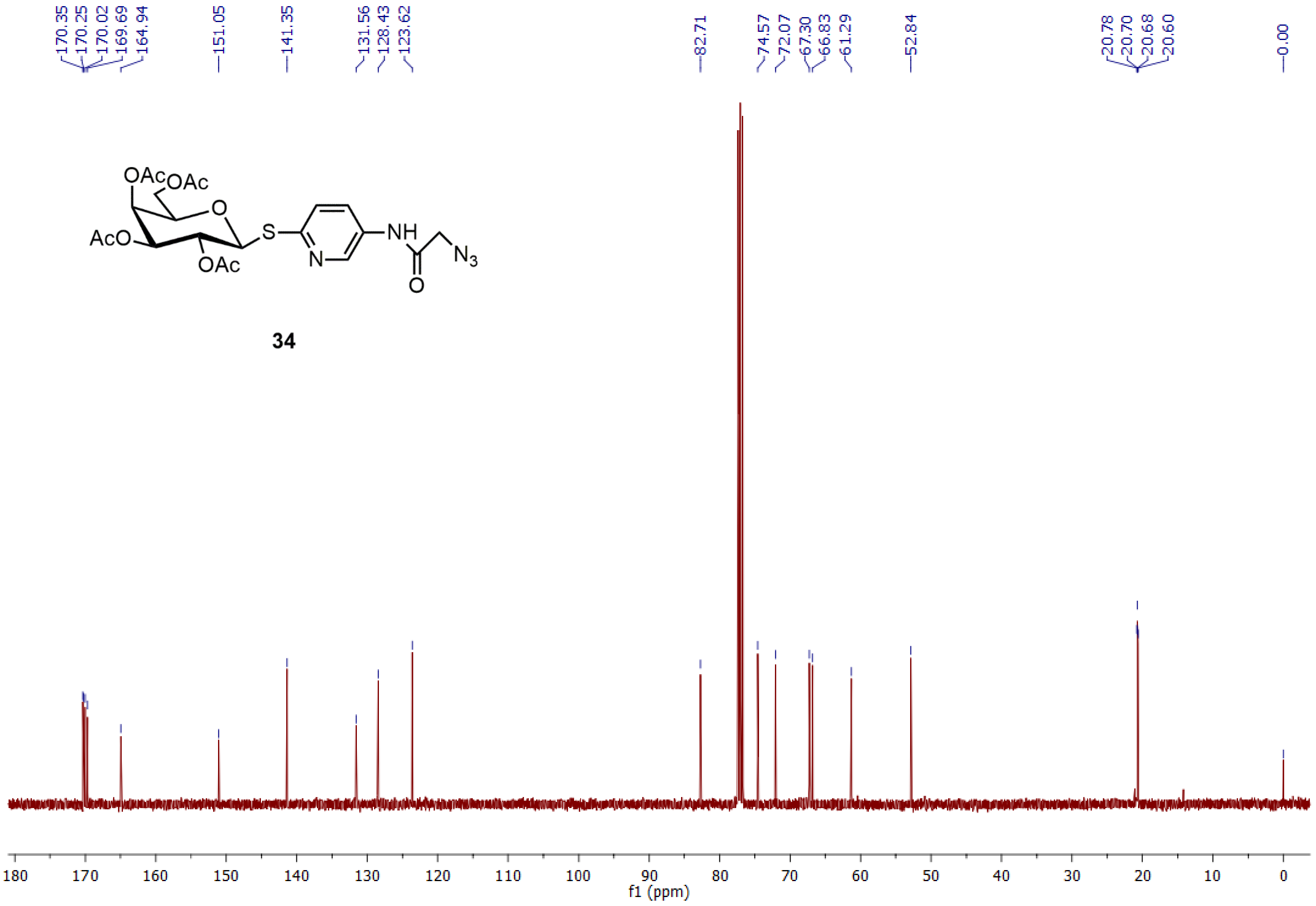

Fig. S53: ^{13}C NMR spectrum of compound **34** (100 MHz/$CDCl_3$/TMS; δ (ppm)).

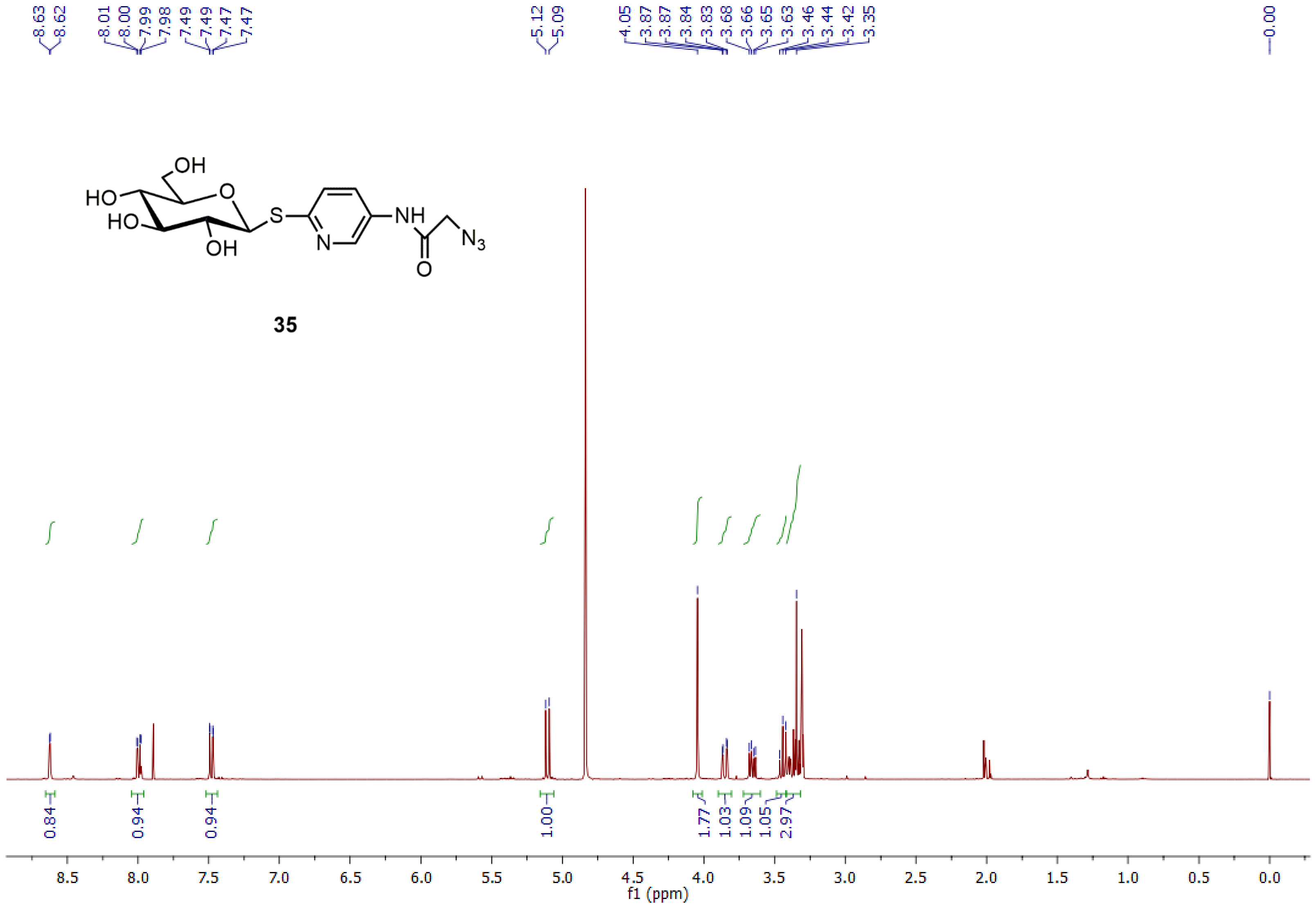

Fig. S54: ^{1}H NMR spectrum of compound **35** (400 MHz/CD_3OD/TMS; δ (ppm)).

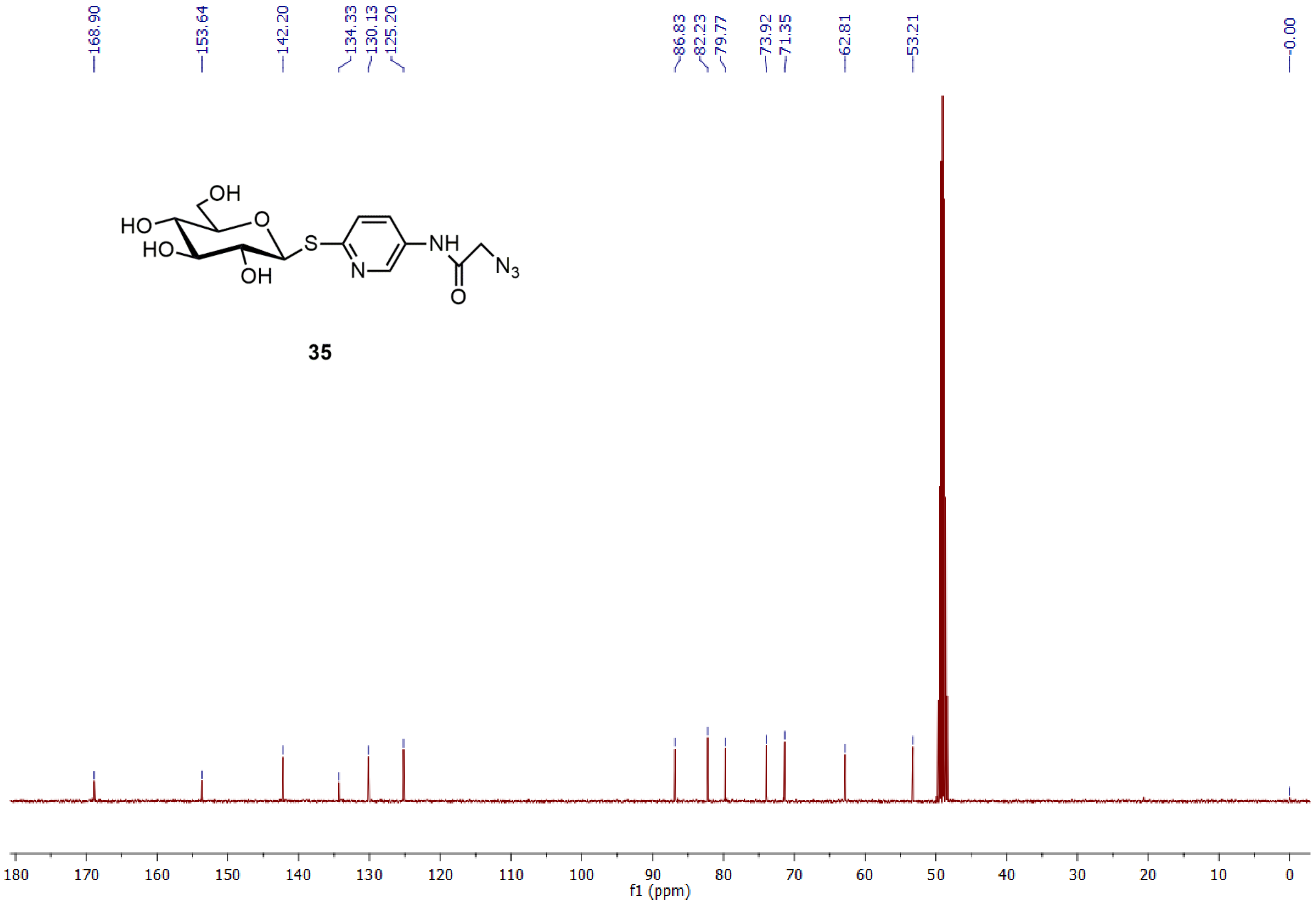

Fig. S55: ^{13}C NMR spectrum of compound **35** (100 MHz/CD_3OD/TMS; δ (ppm)).

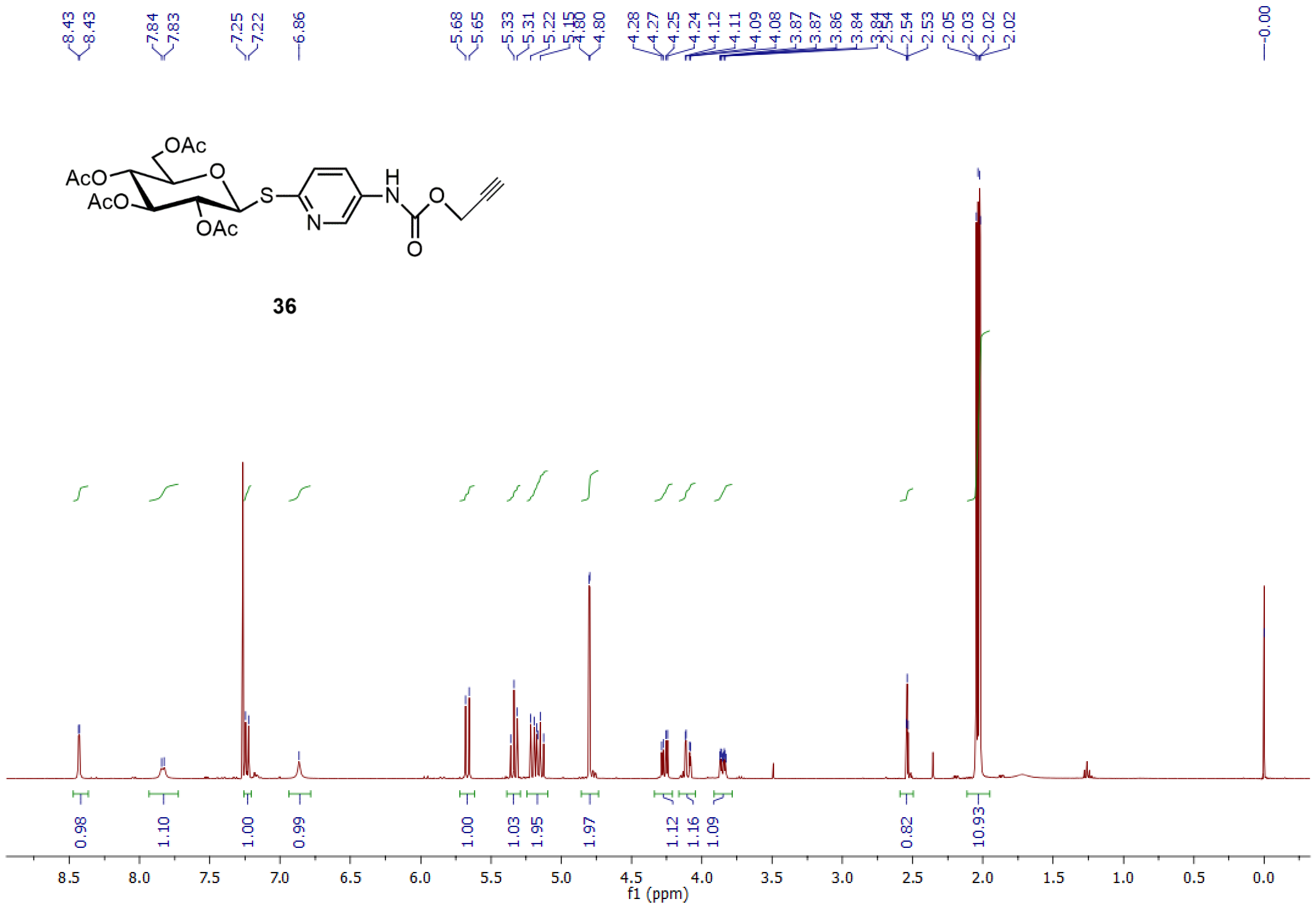

Fig. S56: ^{1}H NMR spectrum of compound **36** (400 MHz/$CDCl_3$/TMS; δ (ppm)).

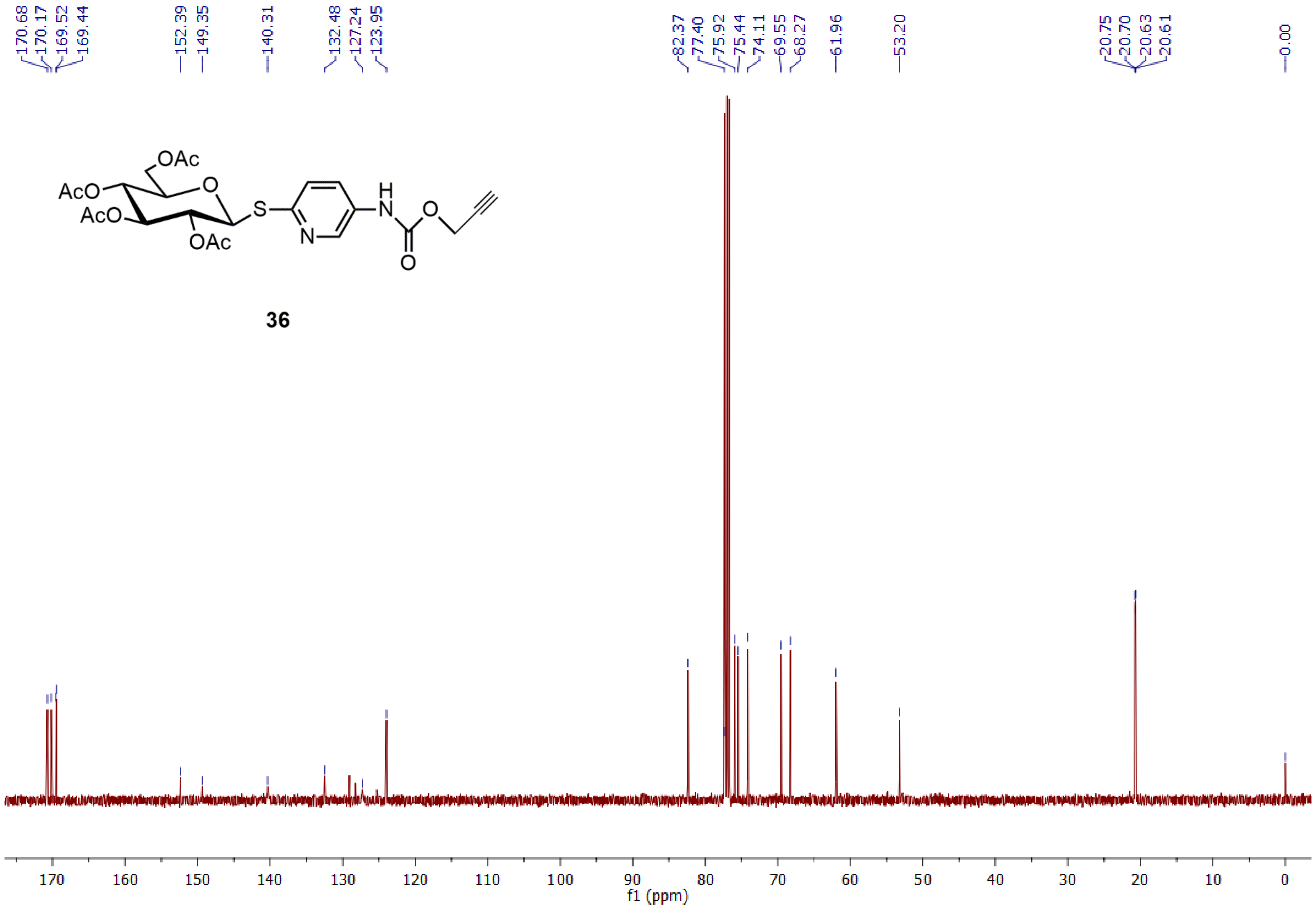

Fig. S57: ^{13}C NMR spectrum of compound **36** (100 MHz/$CDCl_3$/TMS; δ (ppm)).

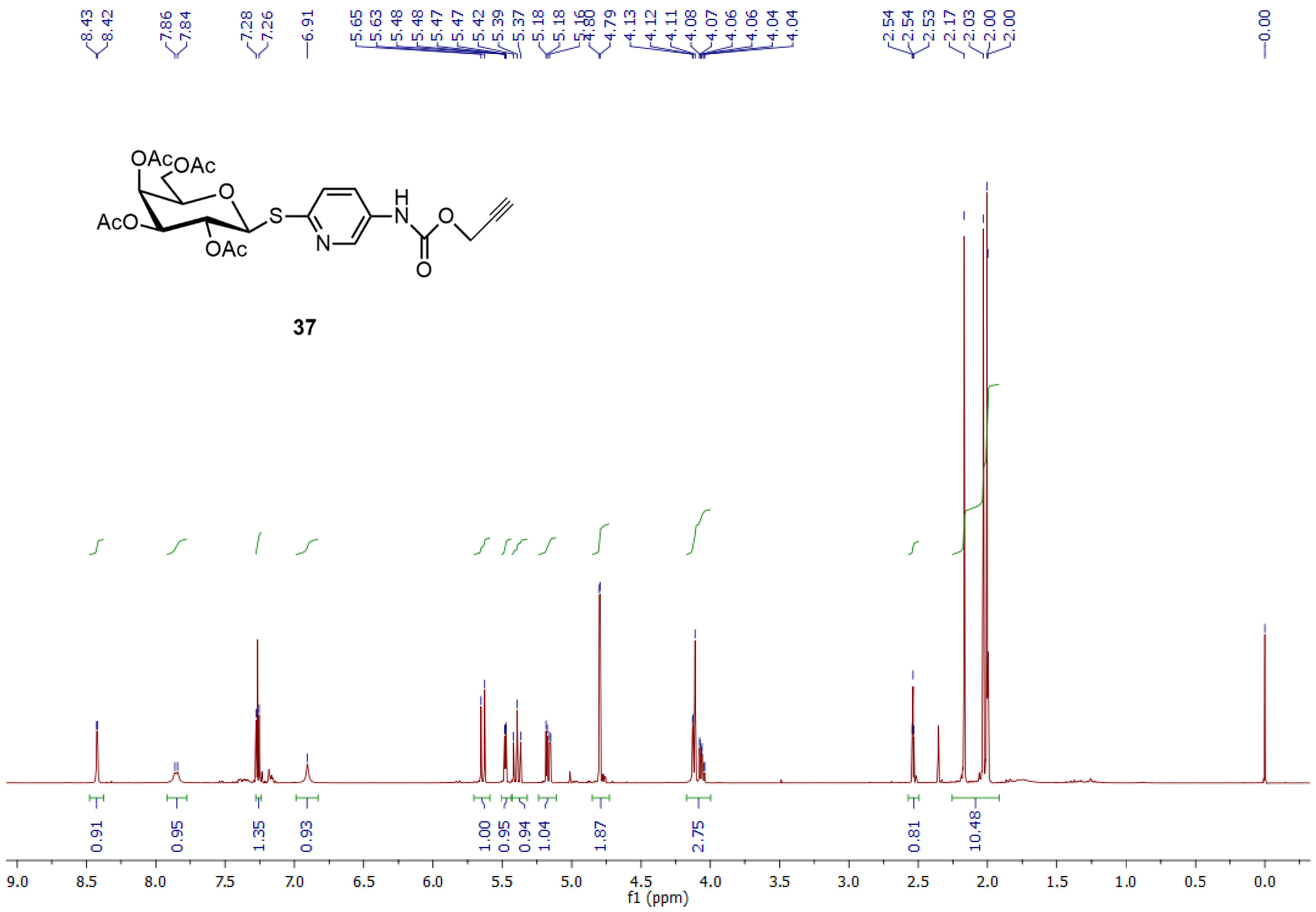

Fig. S58: ^{1}H NMR spectrum of compound **37** (400 MHz/$CDCl_3$/TMS; δ (ppm)).

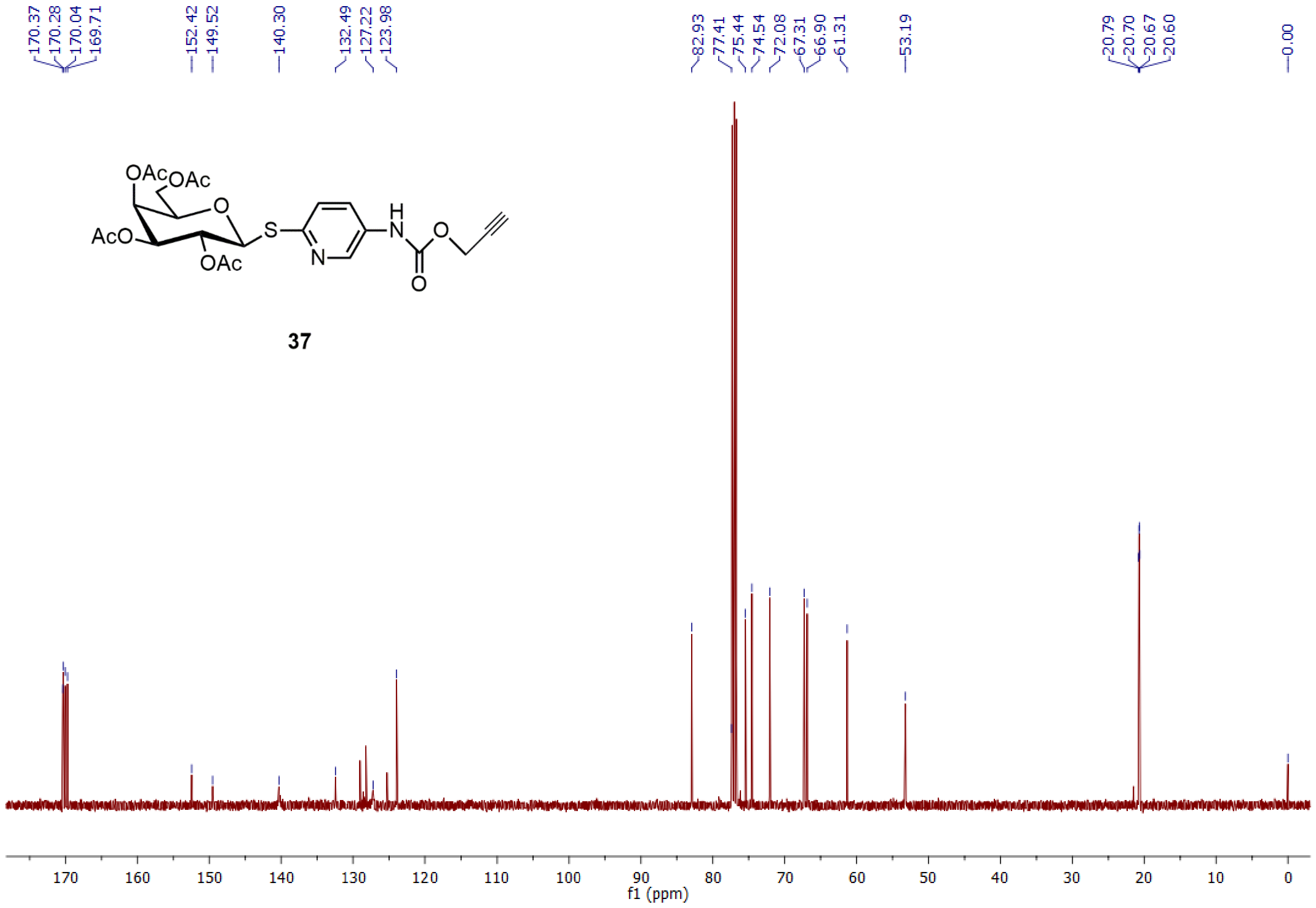

Fig. S59: ^{13}C NMR spectrum of compound **37** (100 MHz/$CDCl_3$/TMS; δ (ppm)).

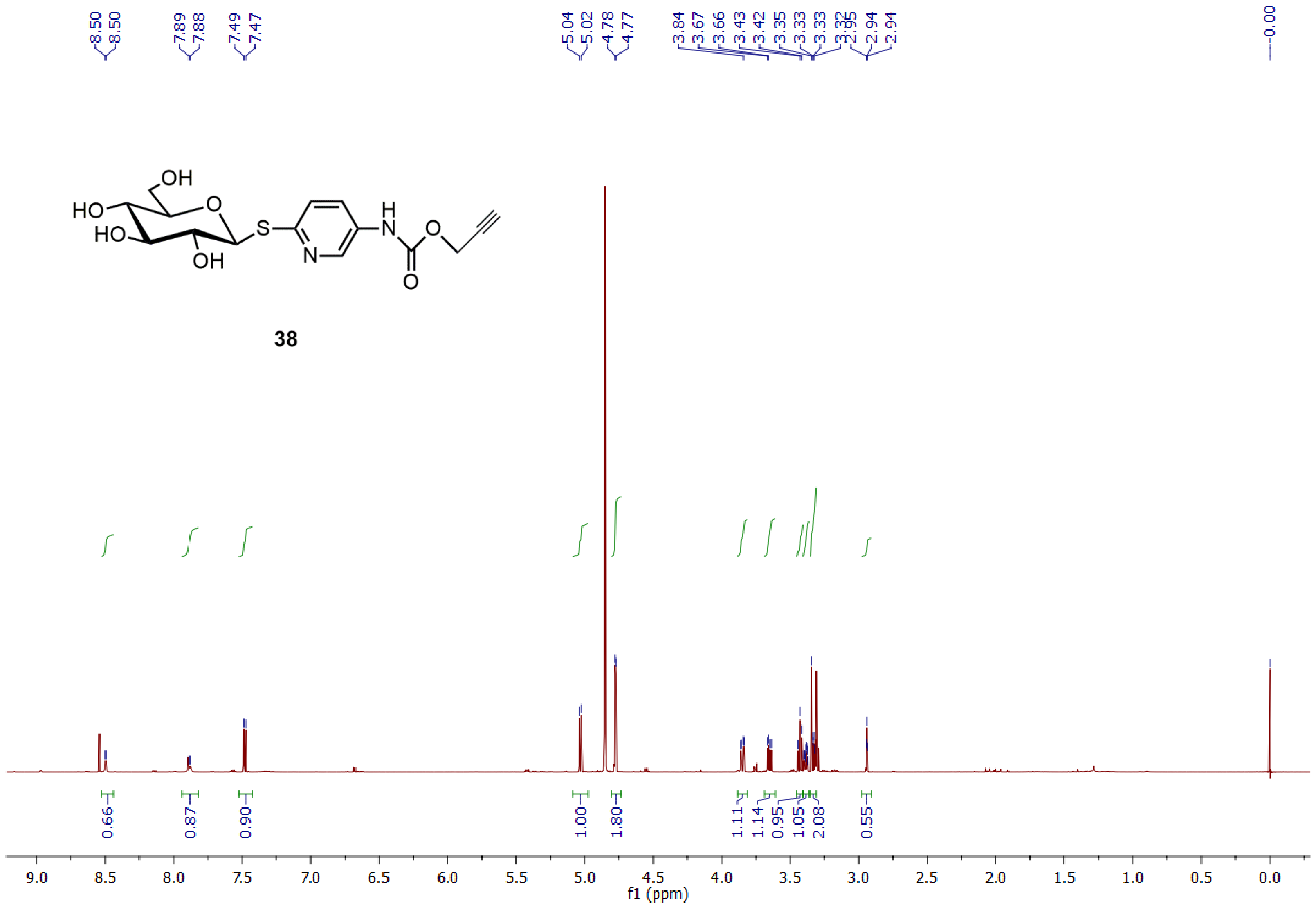

Fig. S60: ^{1}H NMR spectrum of compound **38** (400 MHz/CD_3OD/TMS; δ (ppm)).

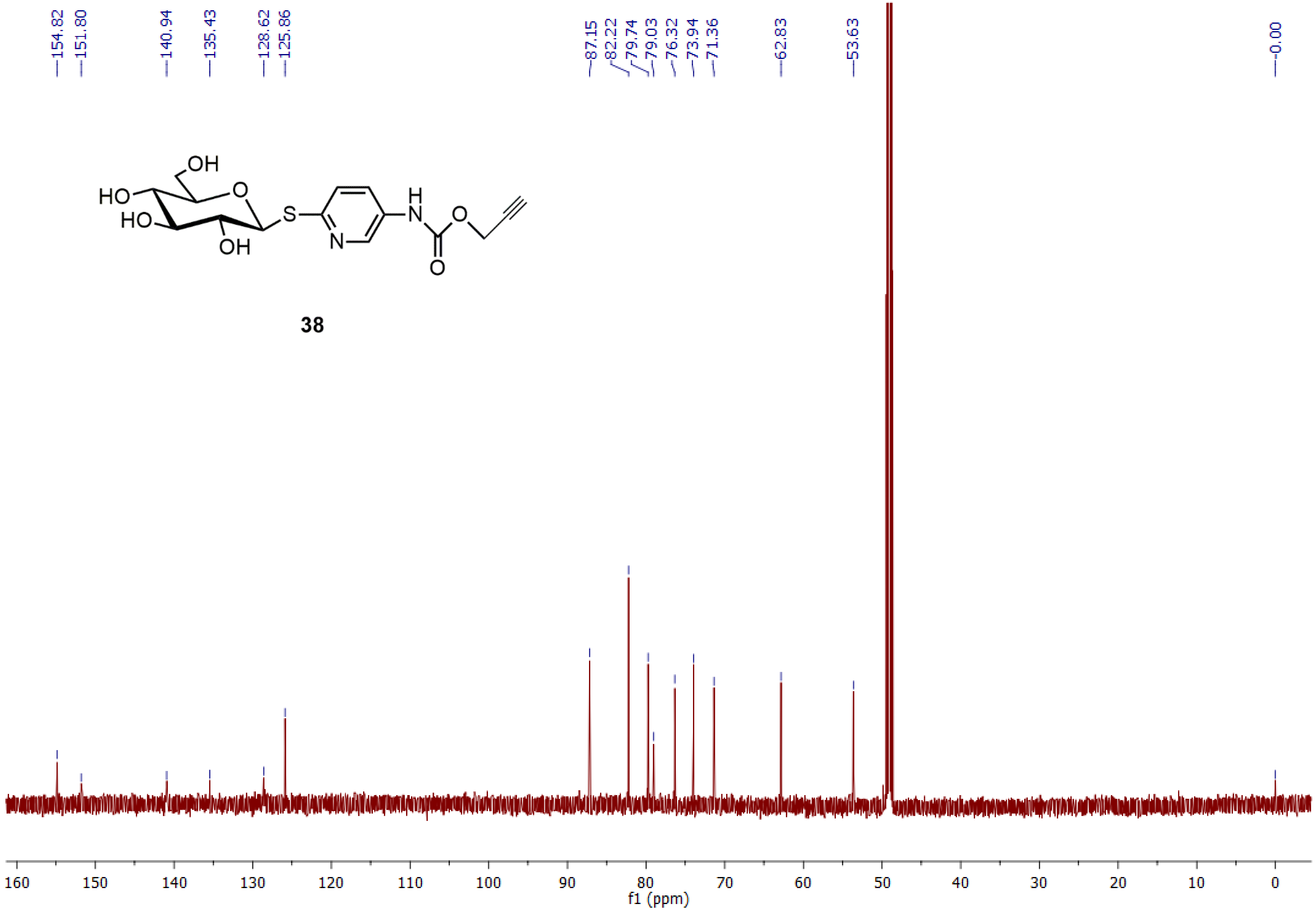

Fig. S61: ^{13}C NMR spectrum of compound **38** (100 MHz/CD_3OD/TMS; δ (ppm)).

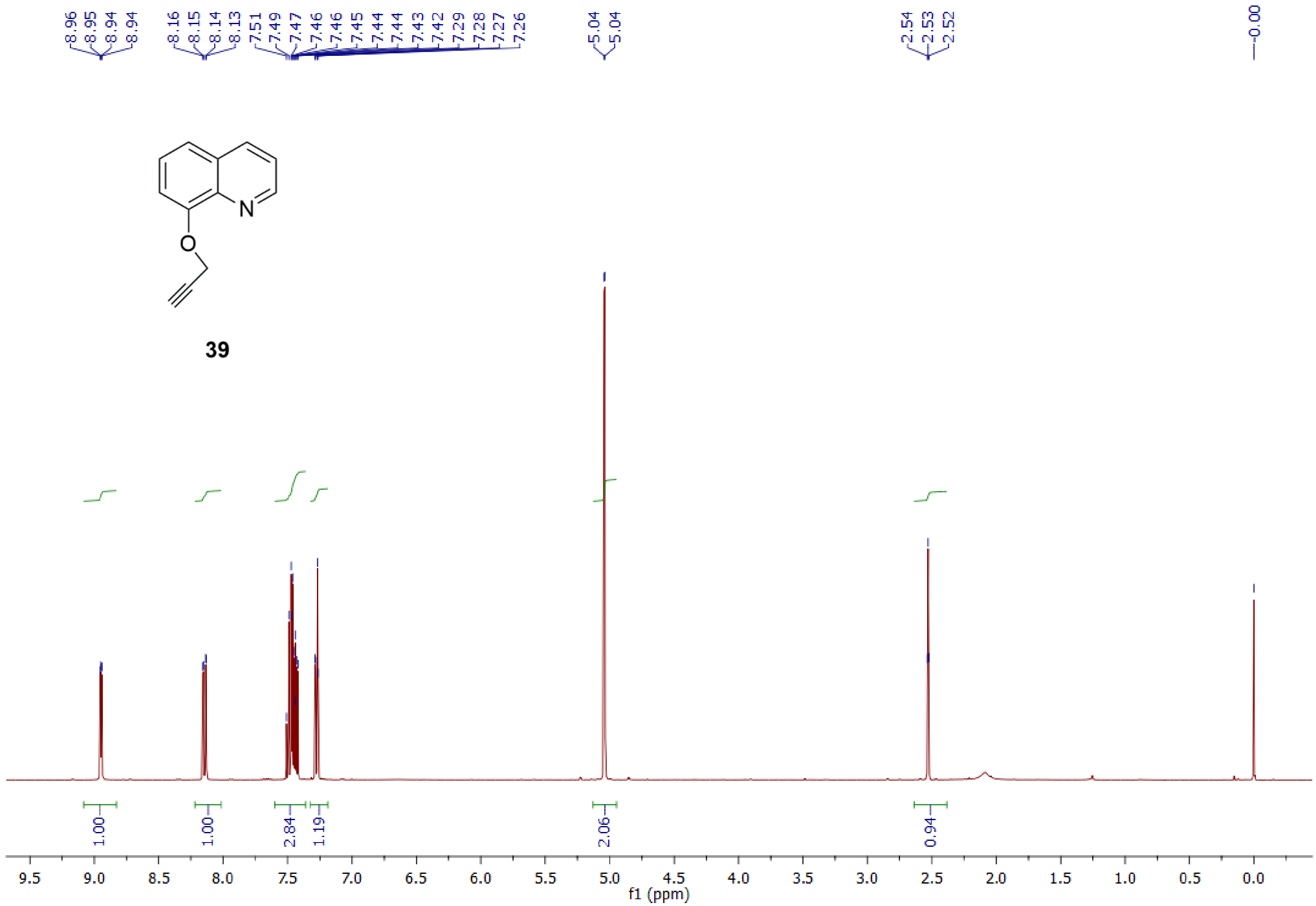

Fig. S62: ^{1}H NMR spectrum of compound **39** (400 MHz/$CDCl_3$/TMS; δ (ppm)).

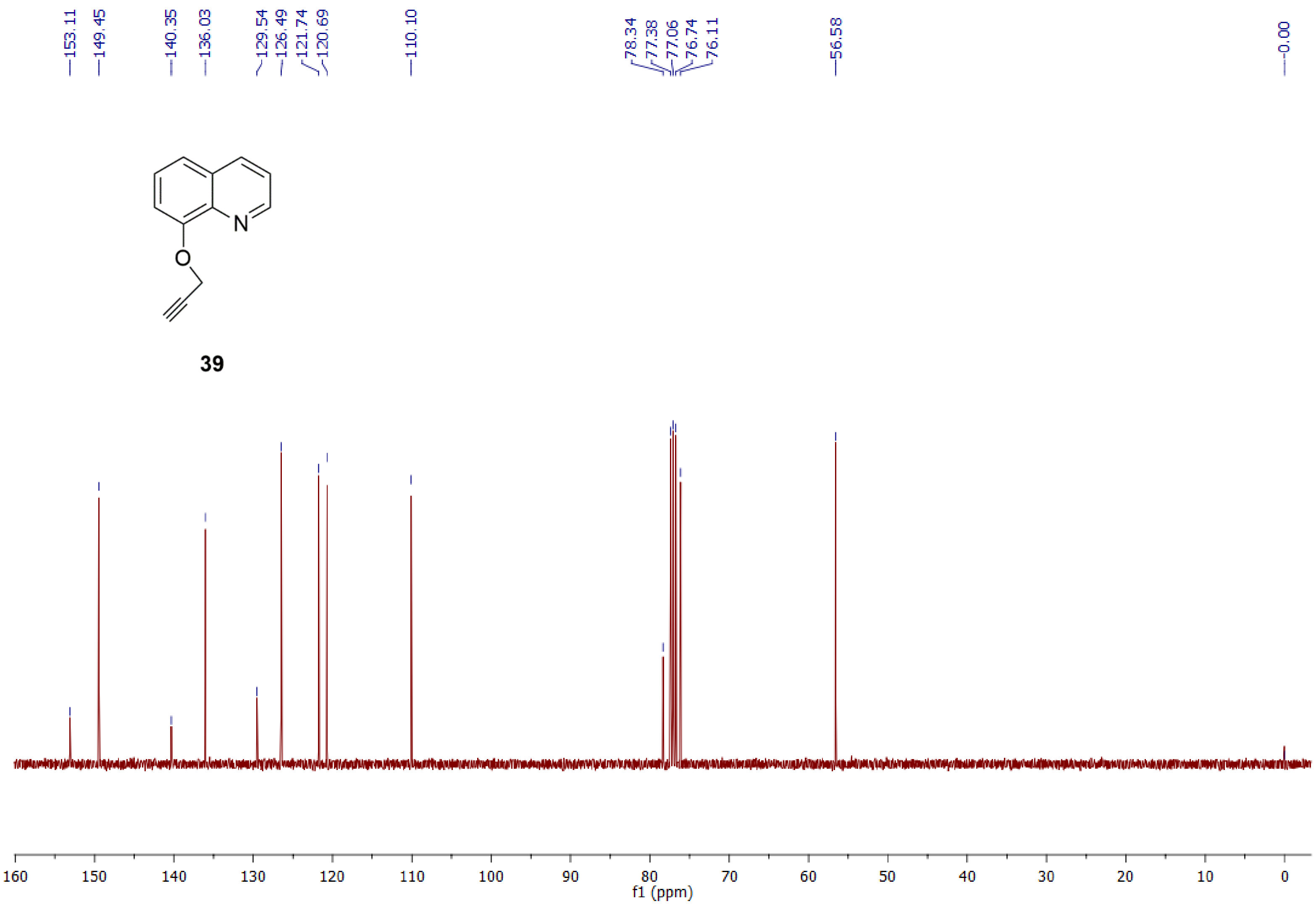

Fig. S63: ^{13}C NMR spectrum of compound **39** (100 MHz/$CDCl_3$/TMS; δ (ppm)).

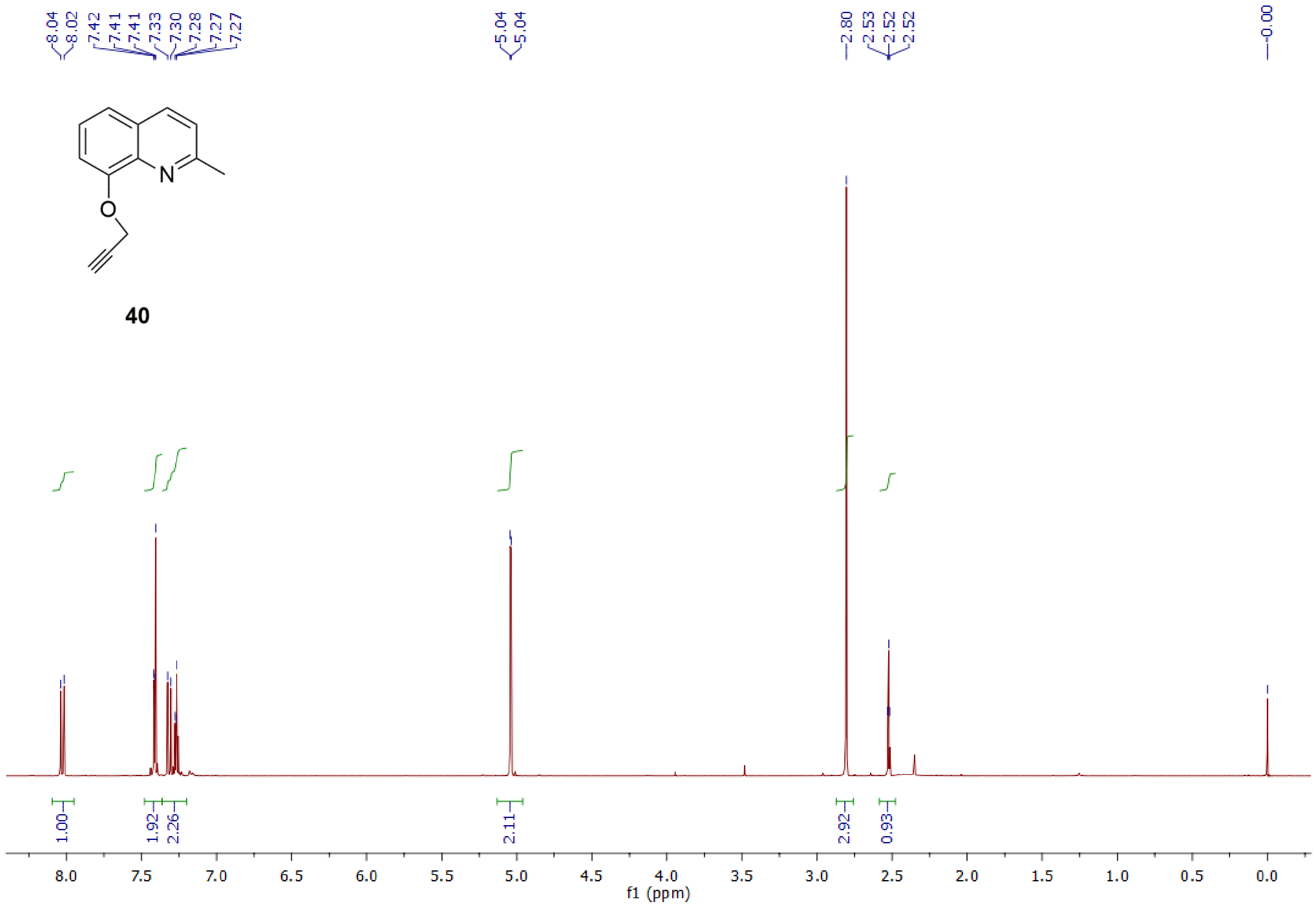

Fig. S64: ^{1}H NMR spectrum of compound **40** (400 MHz/$CDCl_3$/TMS; δ (ppm)).

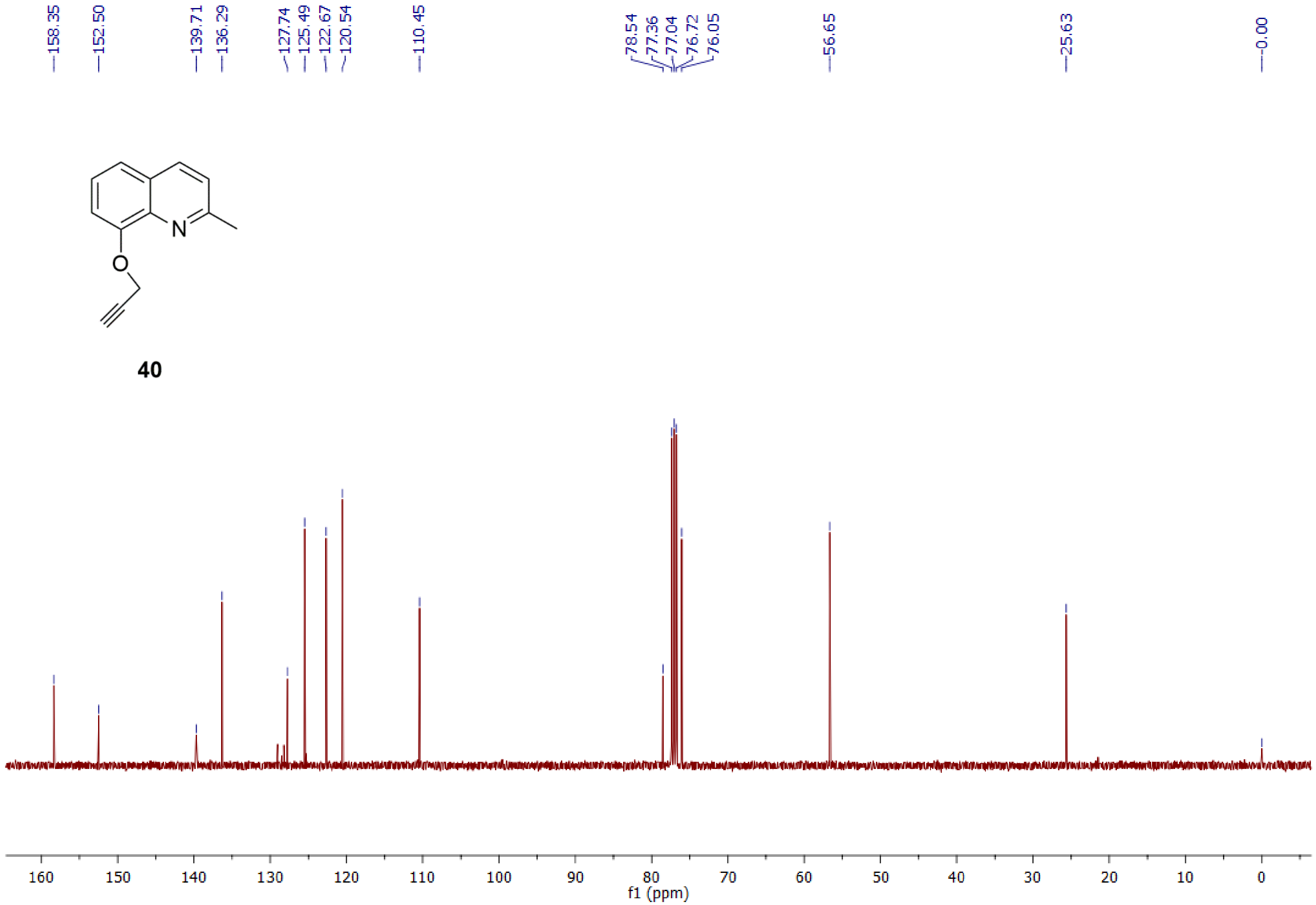

Fig. S65: ^{13}C NMR spectrum of compound **40** (100 MHz/$CDCl_3$/TMS; δ (ppm)).

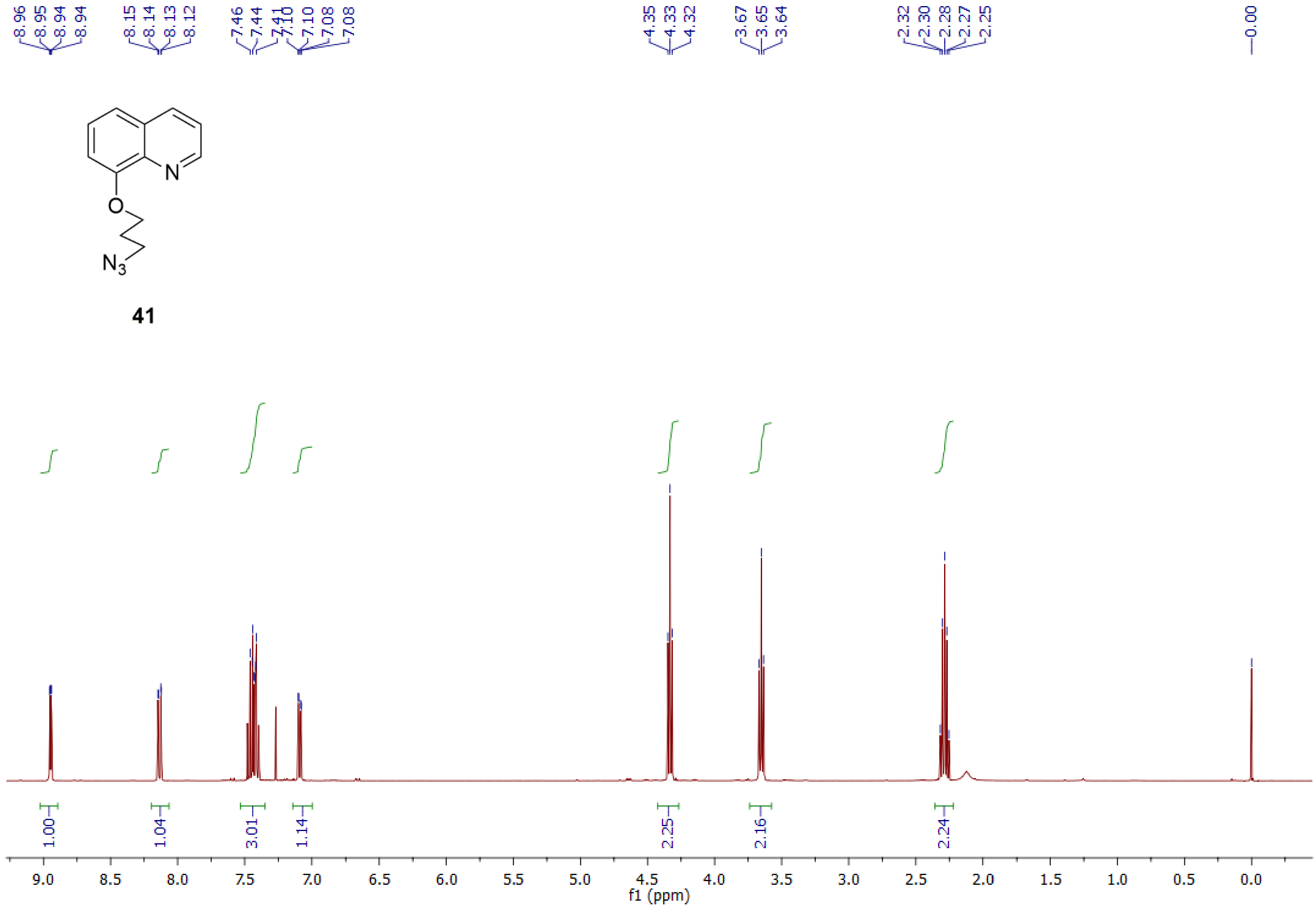

Fig. S66: ^{1}H NMR spectrum of compound **41** (400 MHz/$CDCl_3$/TMS; δ (ppm)).

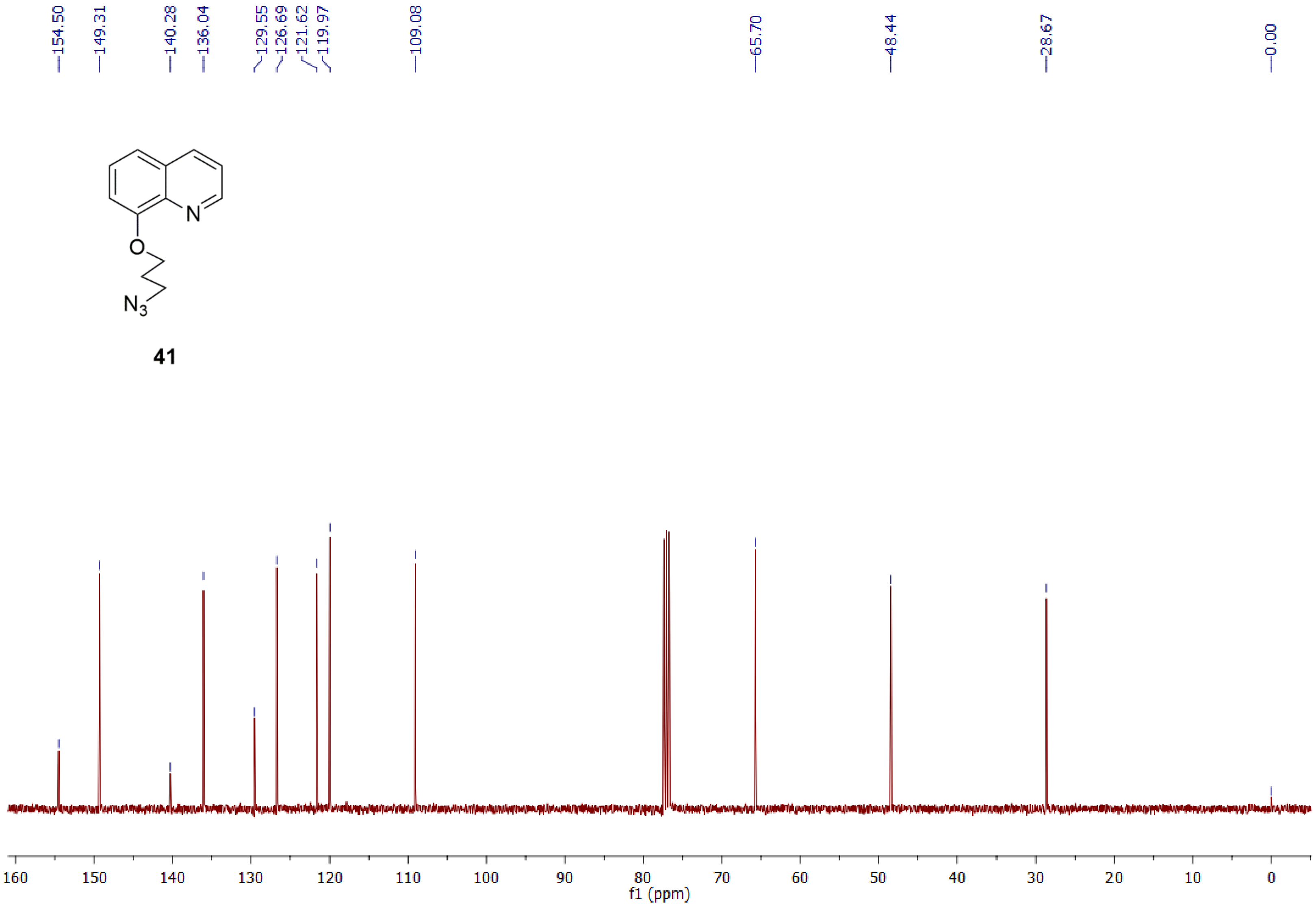

Fig. S67: ^{13}C NMR spectrum of compound **41** (100 MHz/$CDCl_3$/TMS; δ (ppm)).

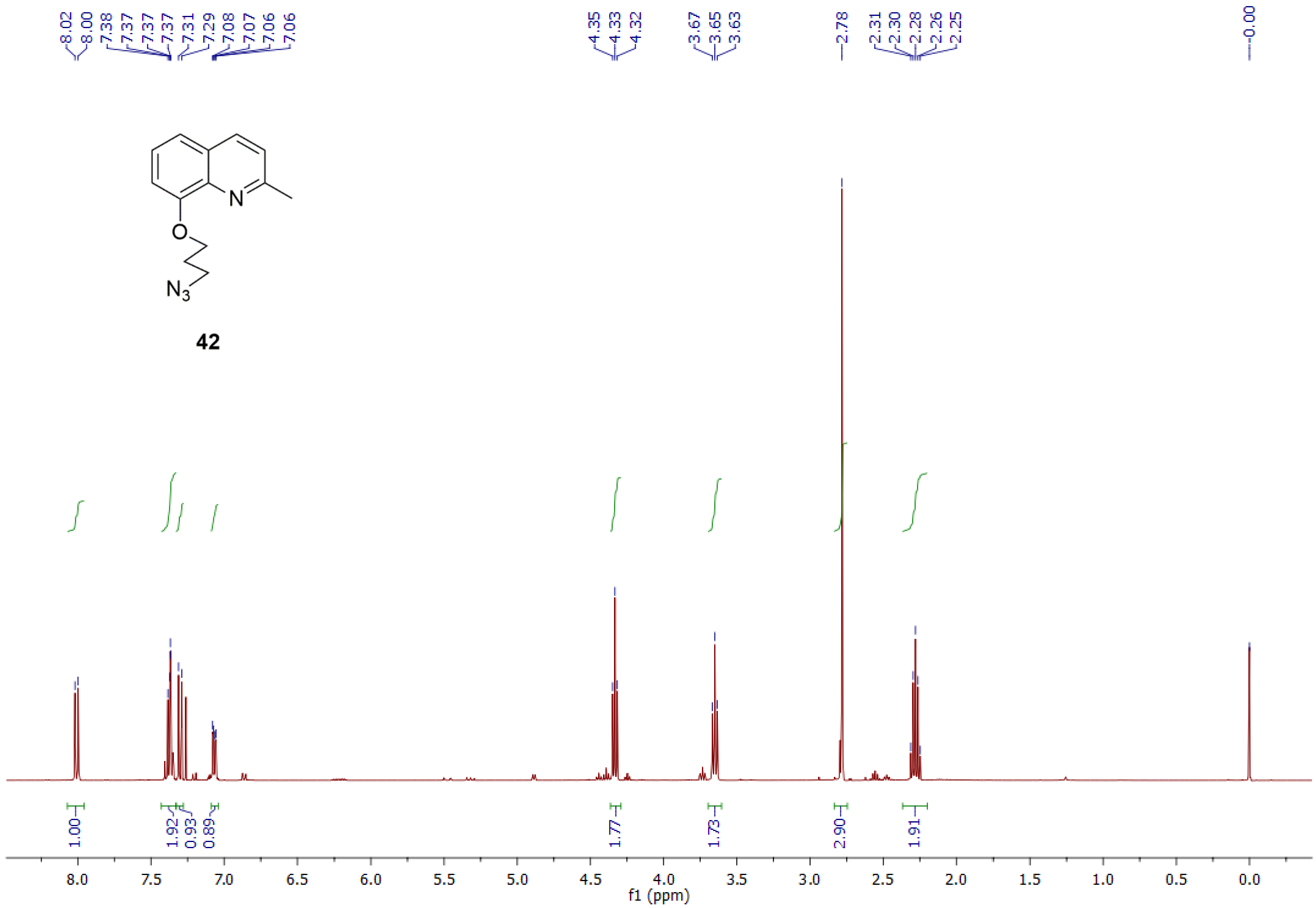

Fig. S68: ^{1}H NMR spectrum of compound **42** (400 MHz/$CDCl_3$/TMS; δ (ppm)).

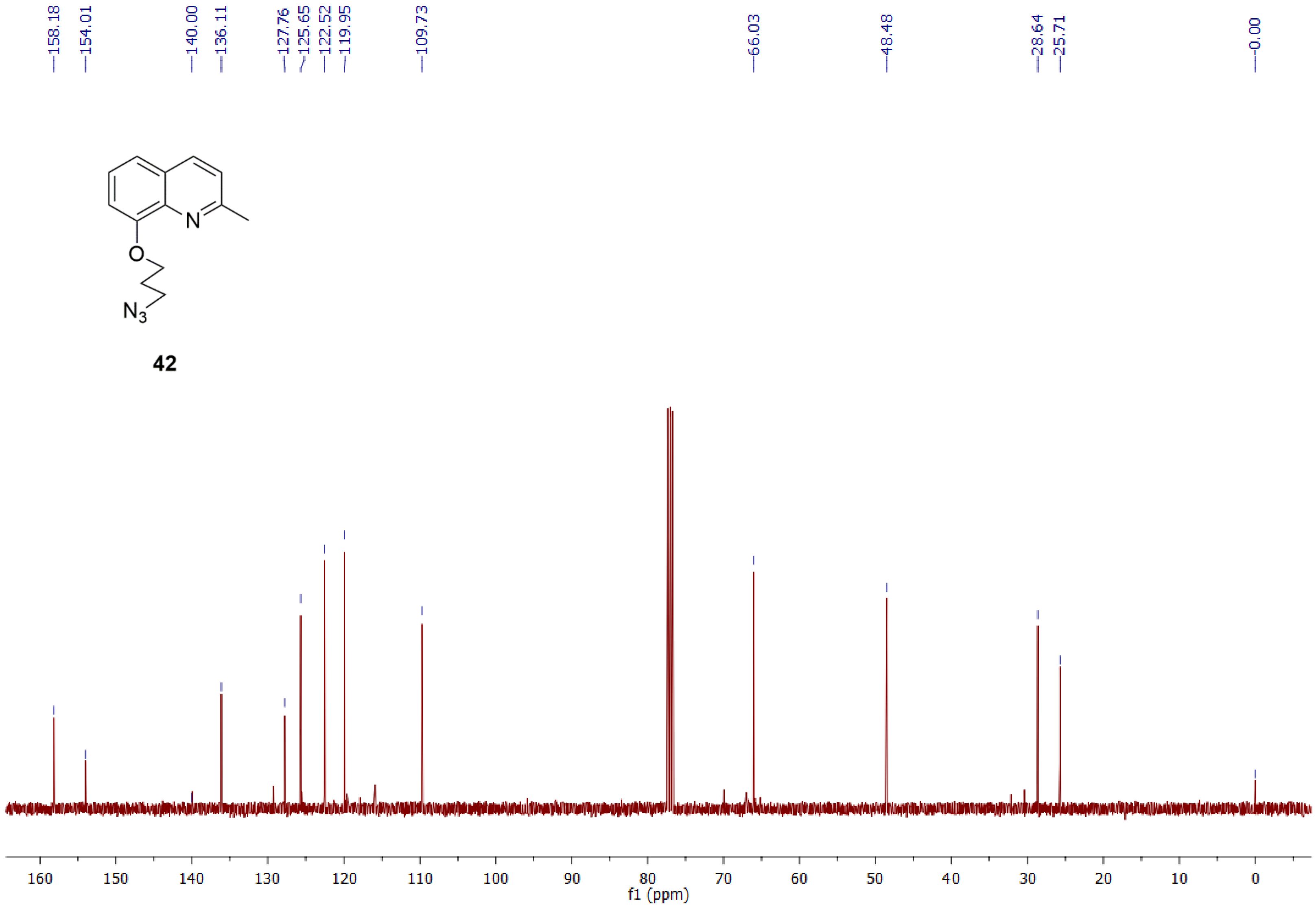

Fig. S69: ^{13}C NMR spectrum of compound **42** (100 MHz/$CDCl_3$/TMS; δ (ppm)).

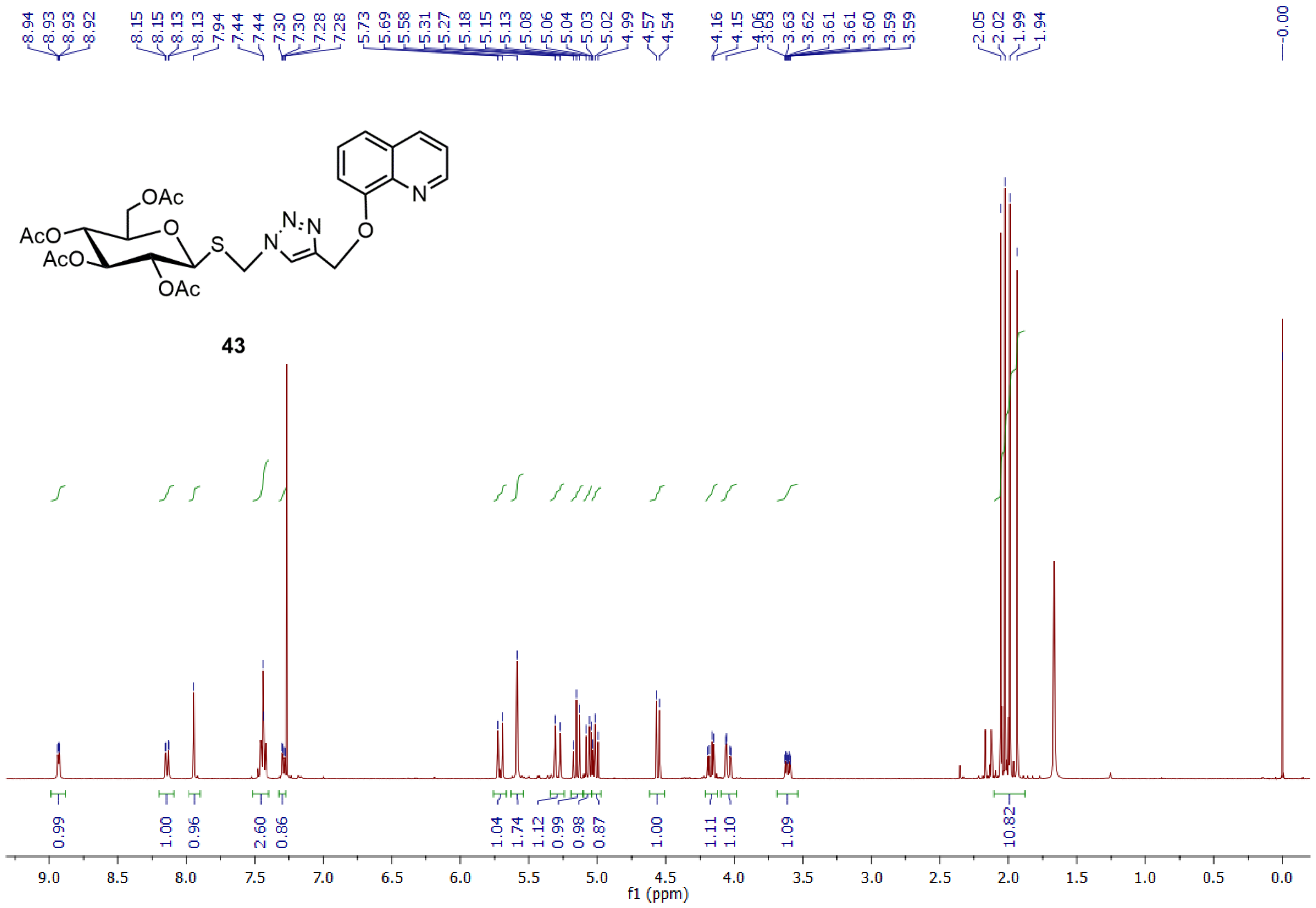

Fig. S70: ^{1}H NMR spectrum of compound **43** (400 MHz/$CDCl_3$/TMS; δ (ppm)).

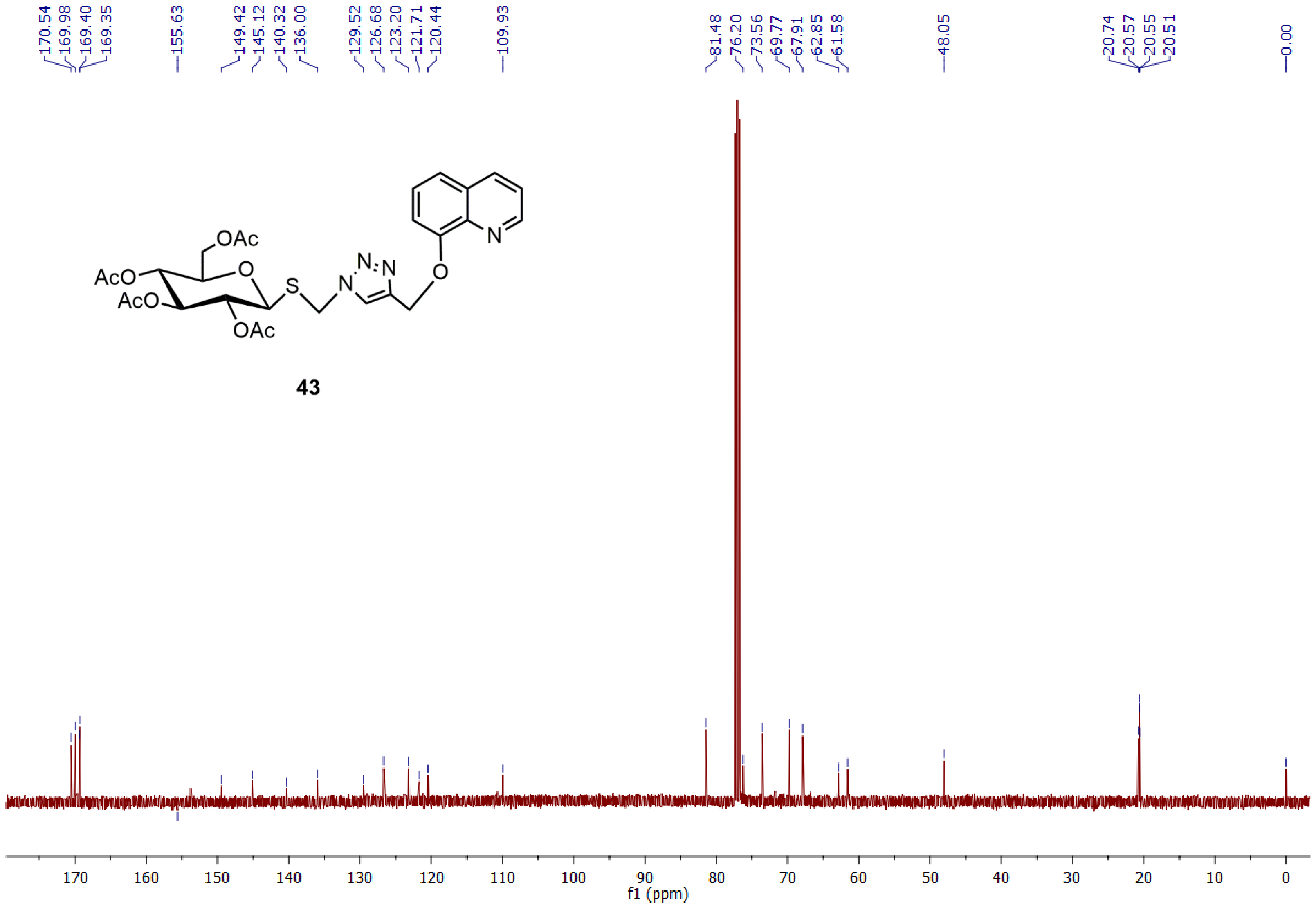

Fig. S71: ^{13}C NMR spectrum of compound **43** (100 MHz/$CDCl_3$/TMS; δ (ppm)).

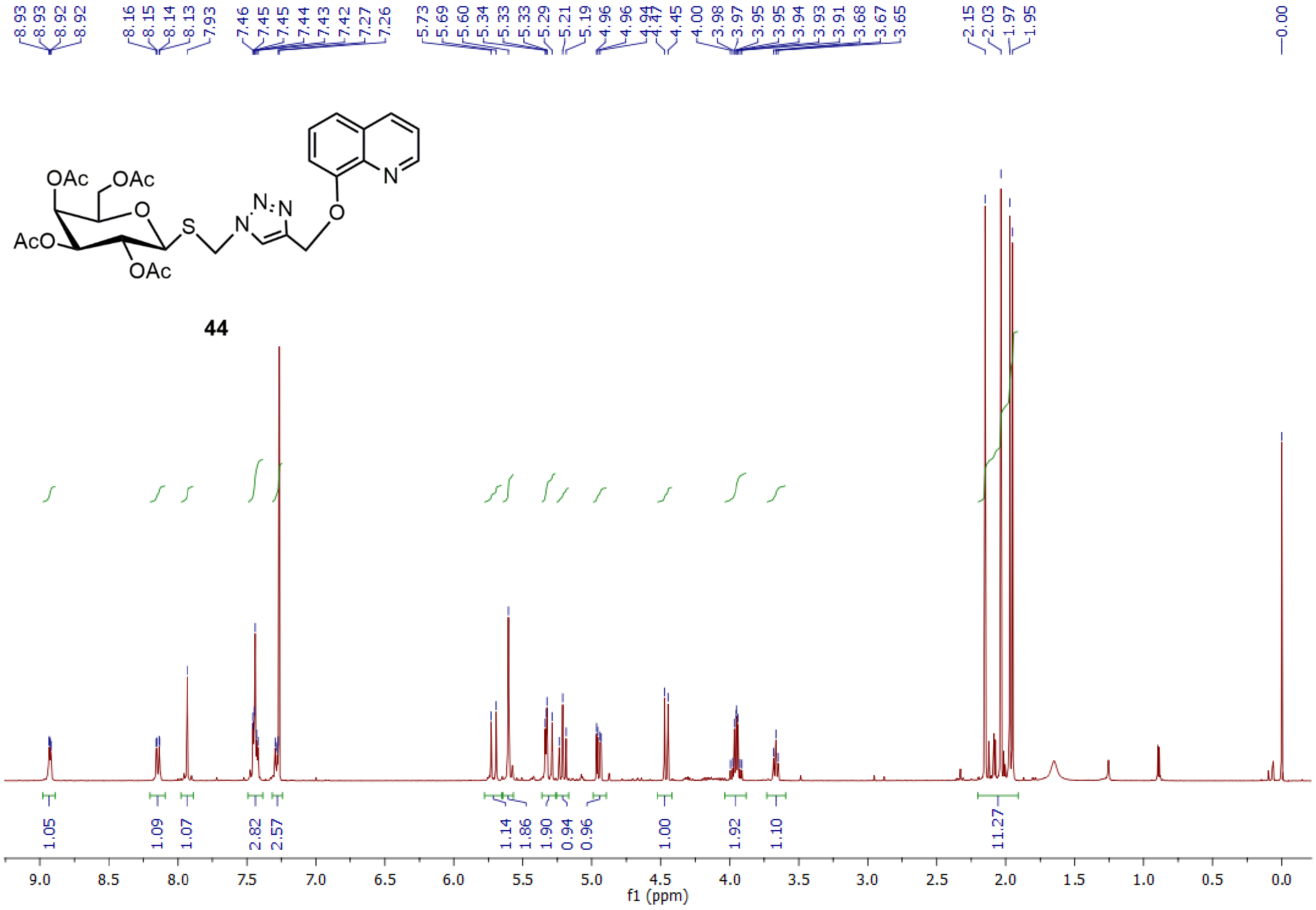

Fig. S72: ^{1}H NMR spectrum of compound **44** (400 MHz/$CDCl_3$/TMS; δ (ppm)).

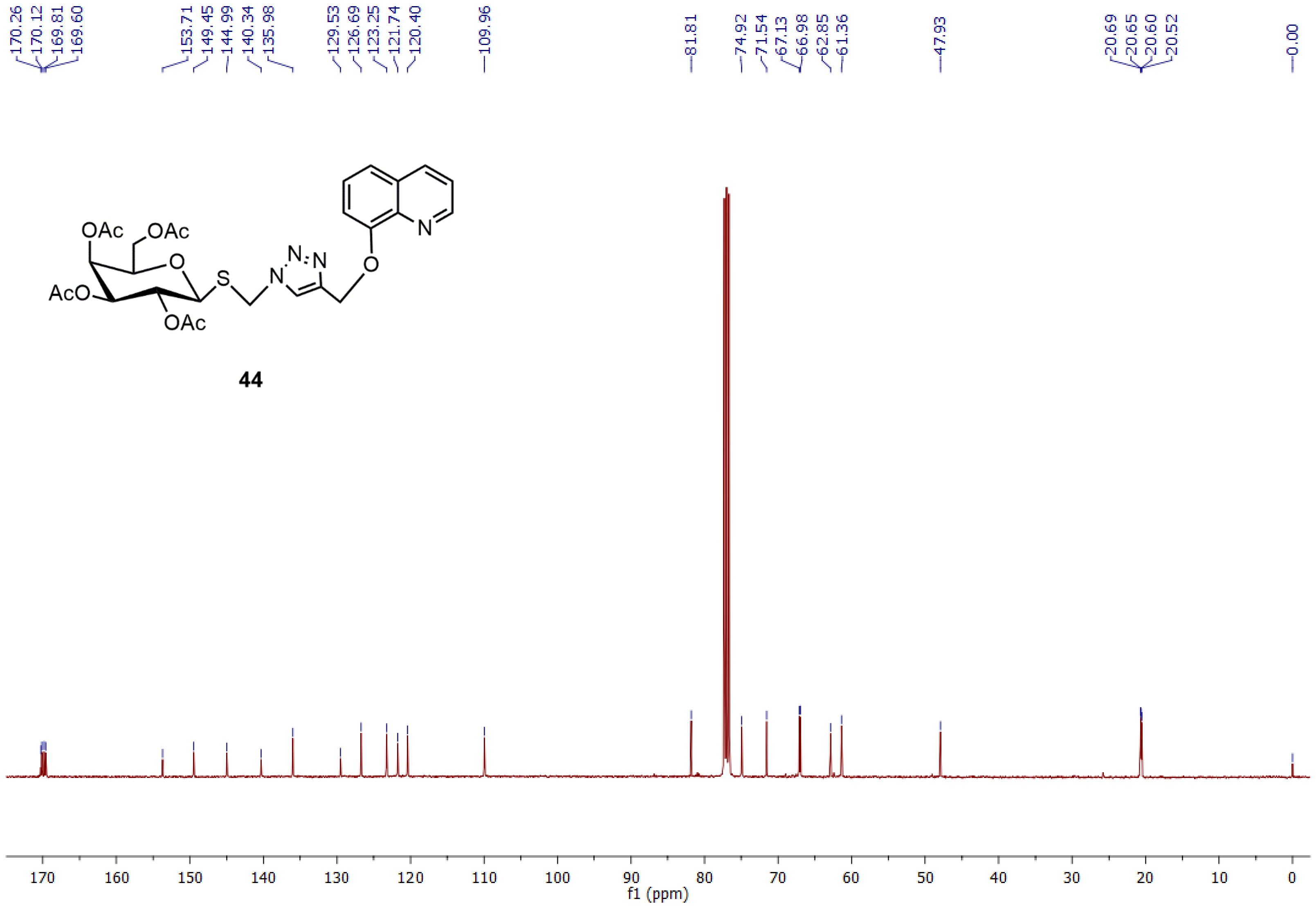

Fig. S73: ^{13}C NMR spectrum of compound **44** (100 MHz/$CDCl_3$/TMS; δ (ppm)).

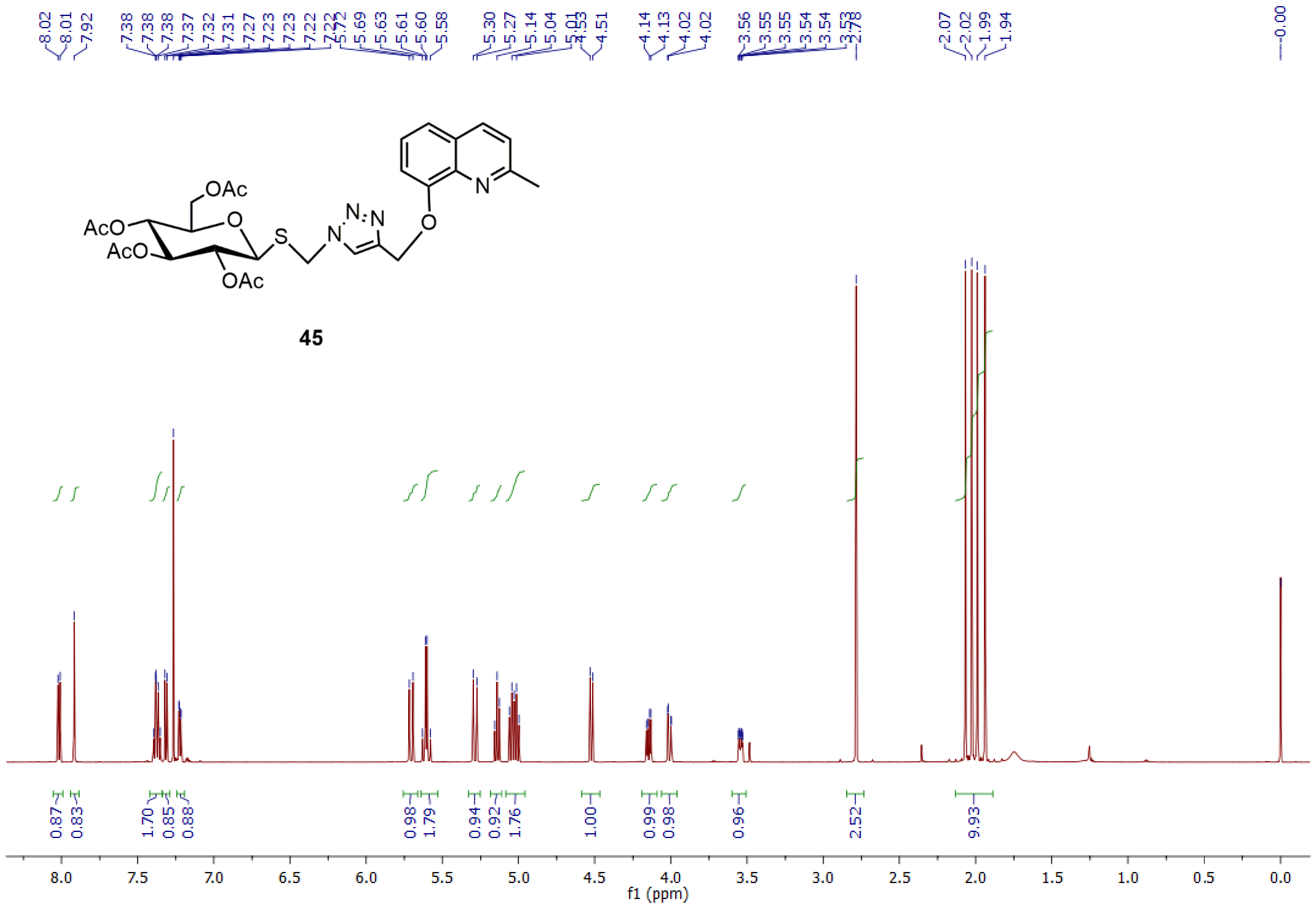

Fig. S74: ^{1}H NMR spectrum of compound **45** (400 MHz/$CDCl_3$/TMS; δ (ppm)).

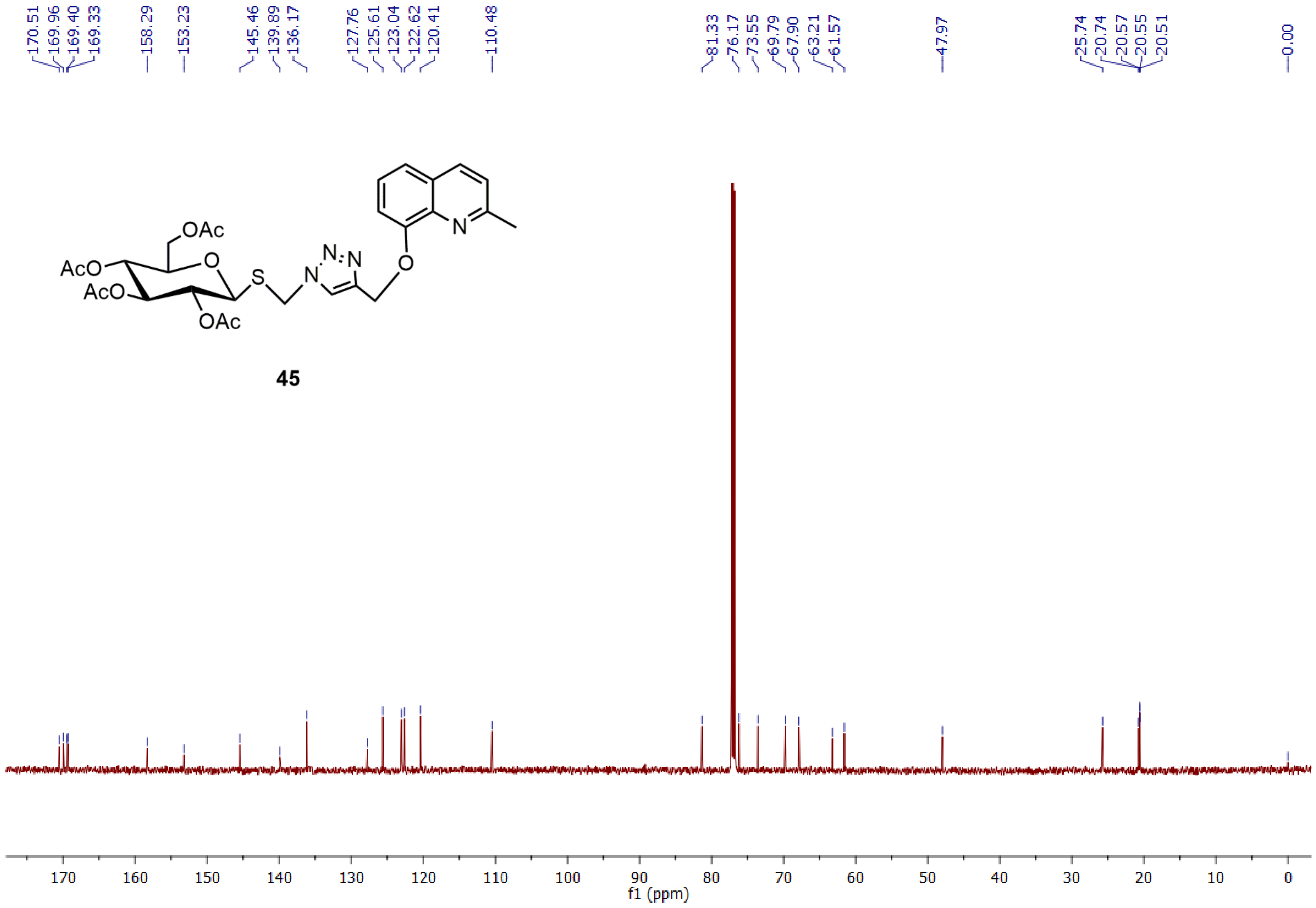

Fig. S75: ^{13}C NMR spectrum of compound **45** (100 MHz/$CDCl_3$/TMS; δ (ppm)).

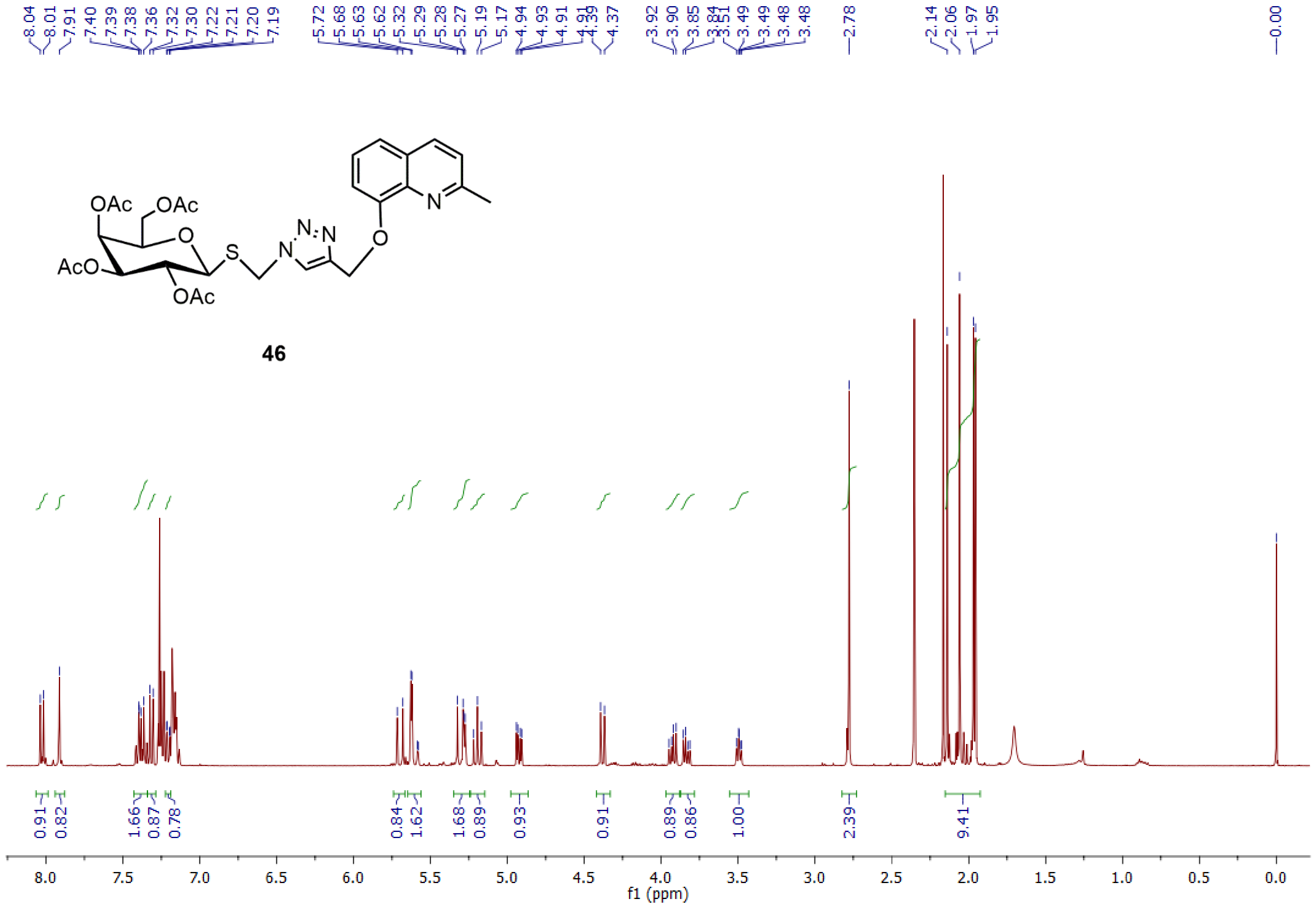

Fig. S76: ^{1}H NMR spectrum of compound **46** (400 MHz/$CDCl_3$/TMS; δ (ppm)).

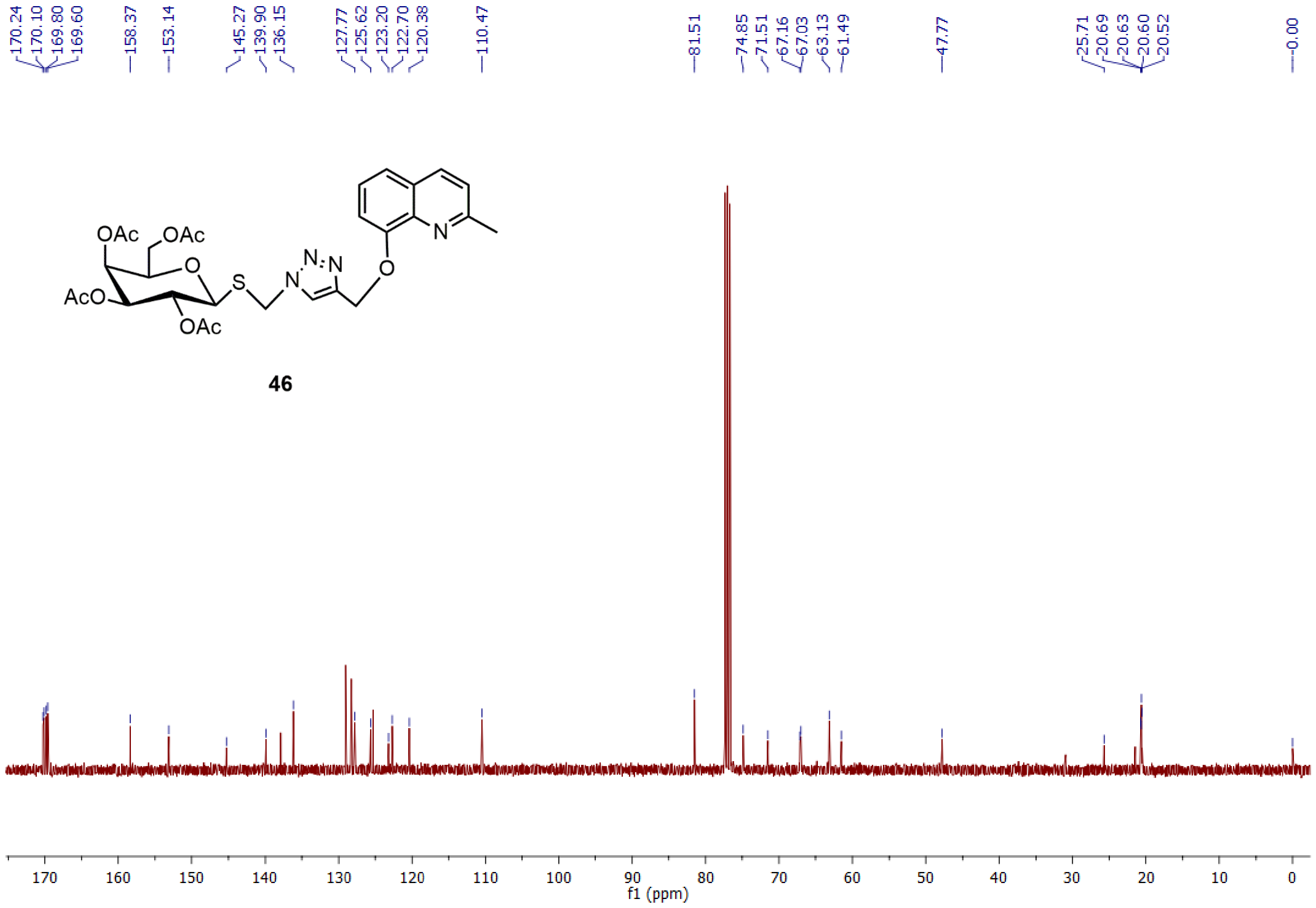

Fig. S77: ^{13}C NMR spectrum of compound **46** (100 MHz/$CDCl_3$/TMS; δ (ppm)).

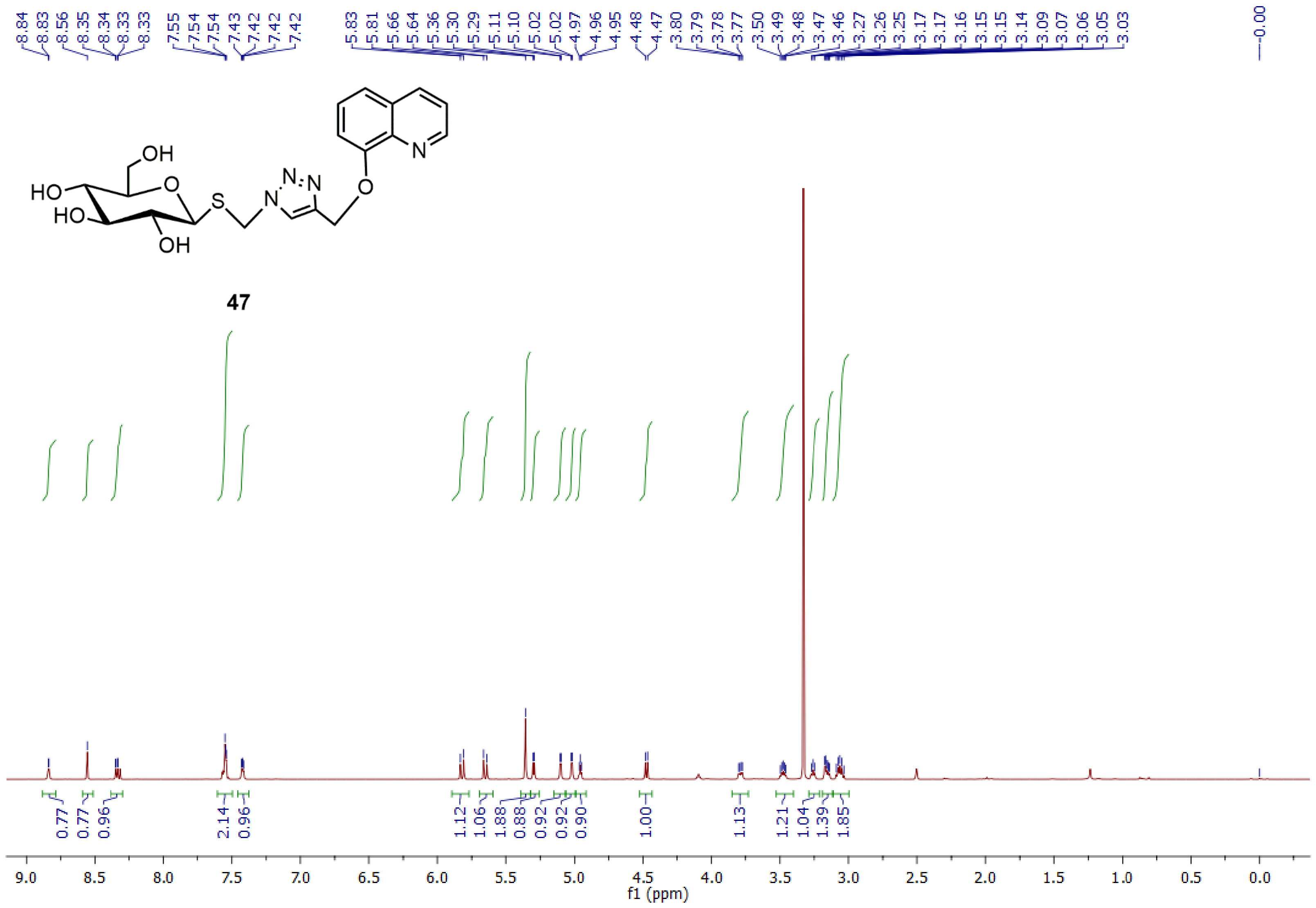

Fig. S78: ¹H NMR spectrum of compound **47** (400 MHz/DMSO/TMS; δ (ppm)).

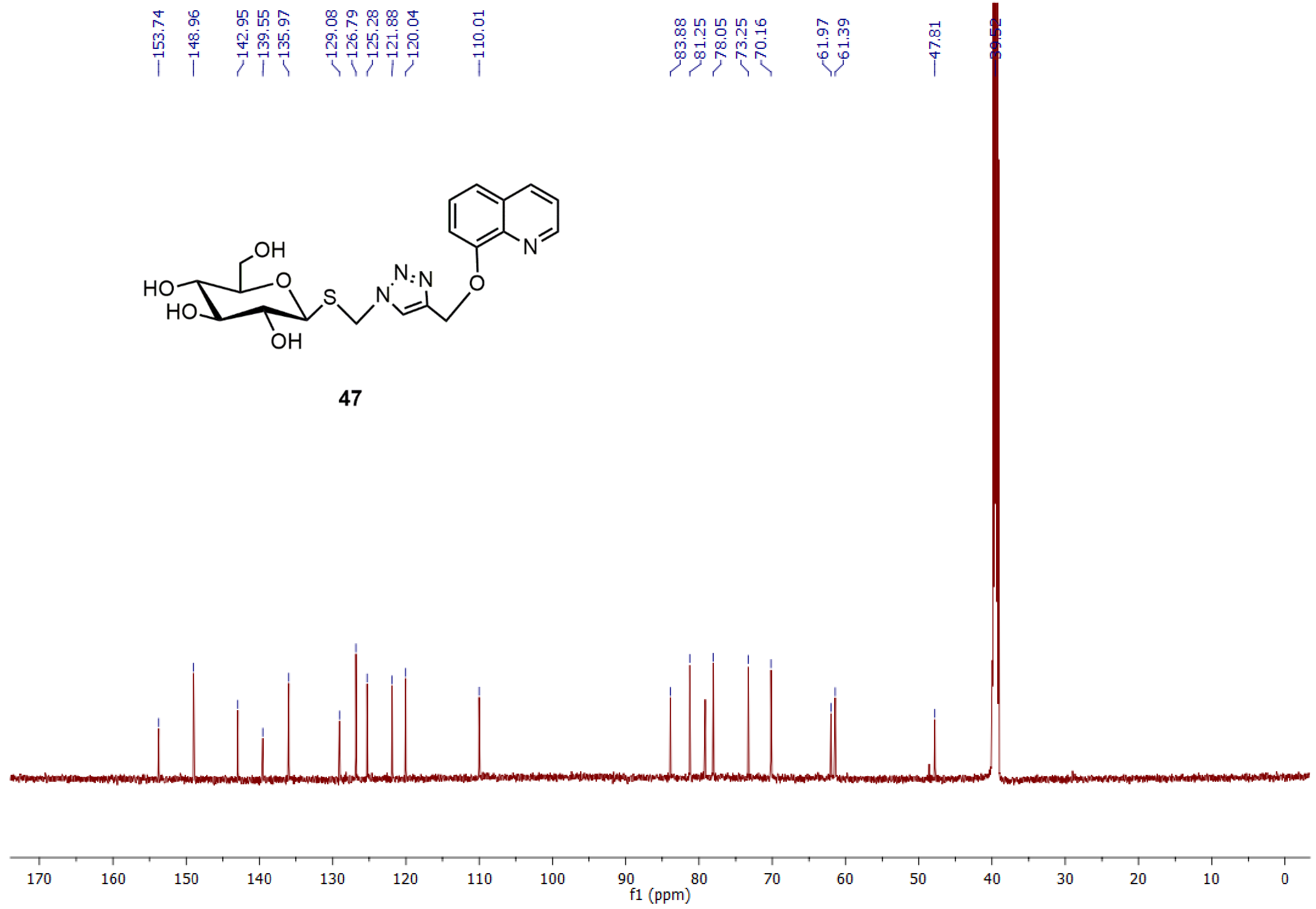

Fig. S79: ^{13}C NMR spectrum of compound **47** (100 MHz/DMSO/TMS; δ (ppm)).

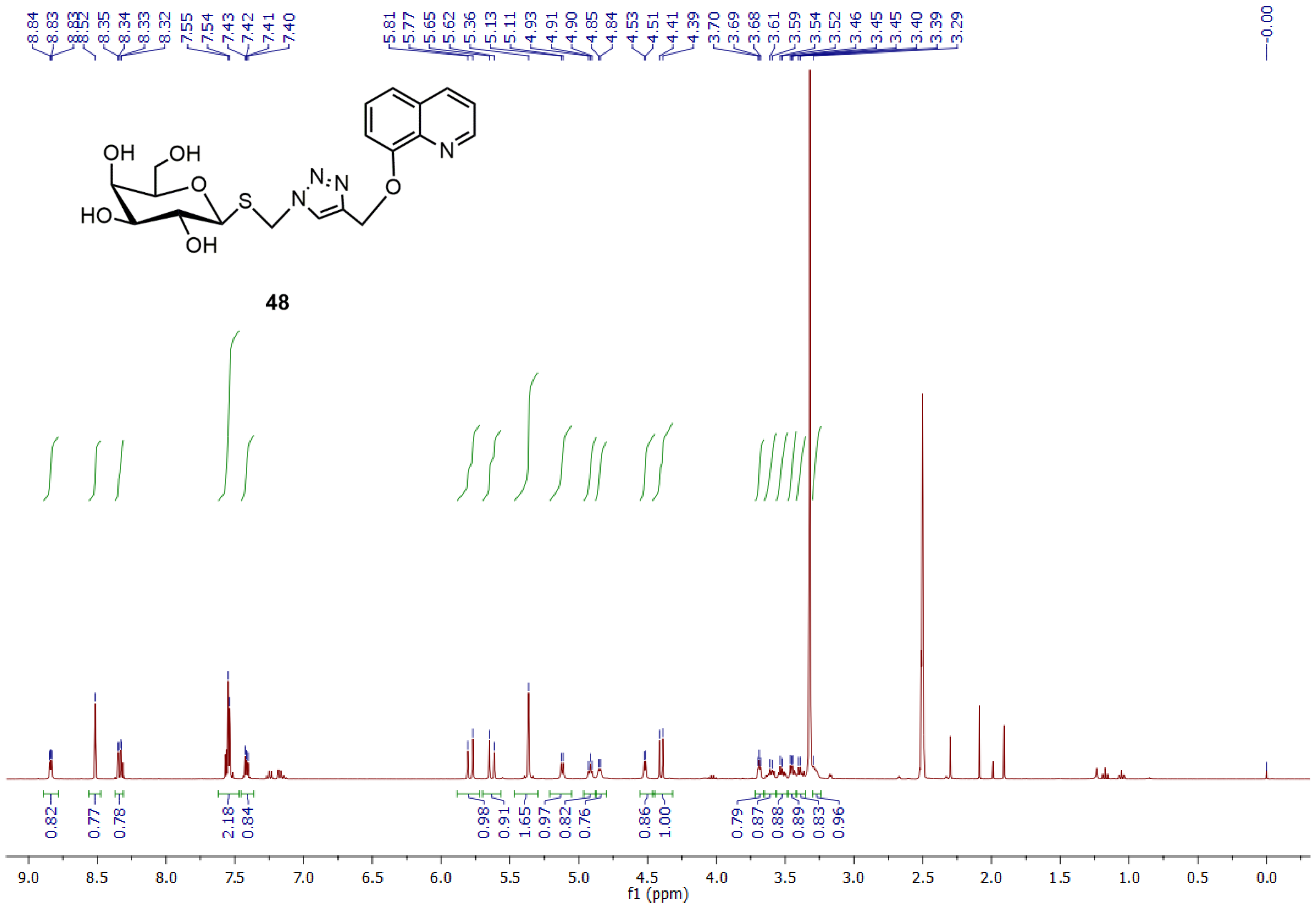

Fig. S80: ^{1}H NMR spectrum of compound **48** (400 MHz/DMSO/TMS; δ (ppm)).

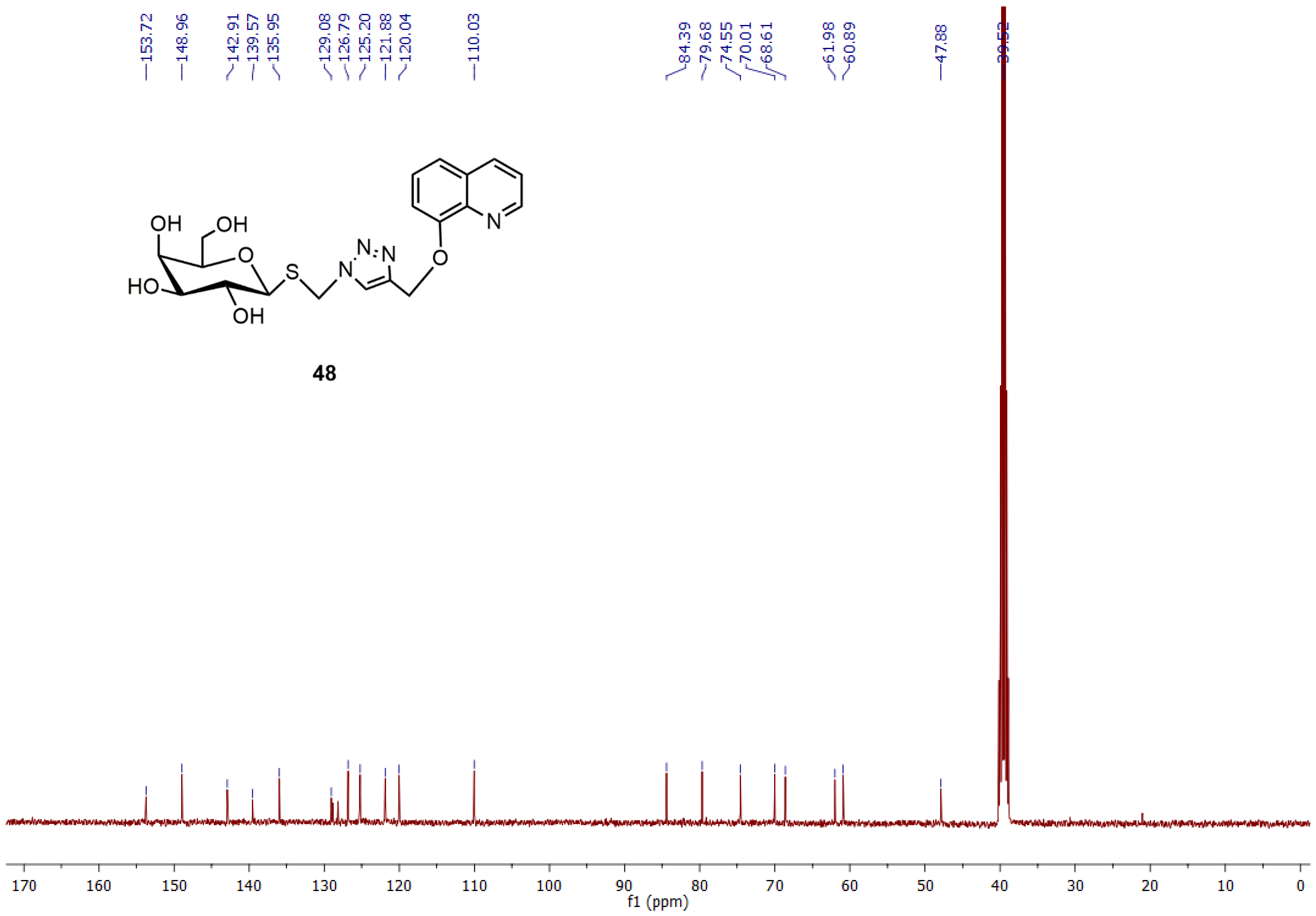

Fig. S81: ^{13}C NMR spectrum of compound **48** (100 MHz/DMSO/TMS; δ (ppm)).

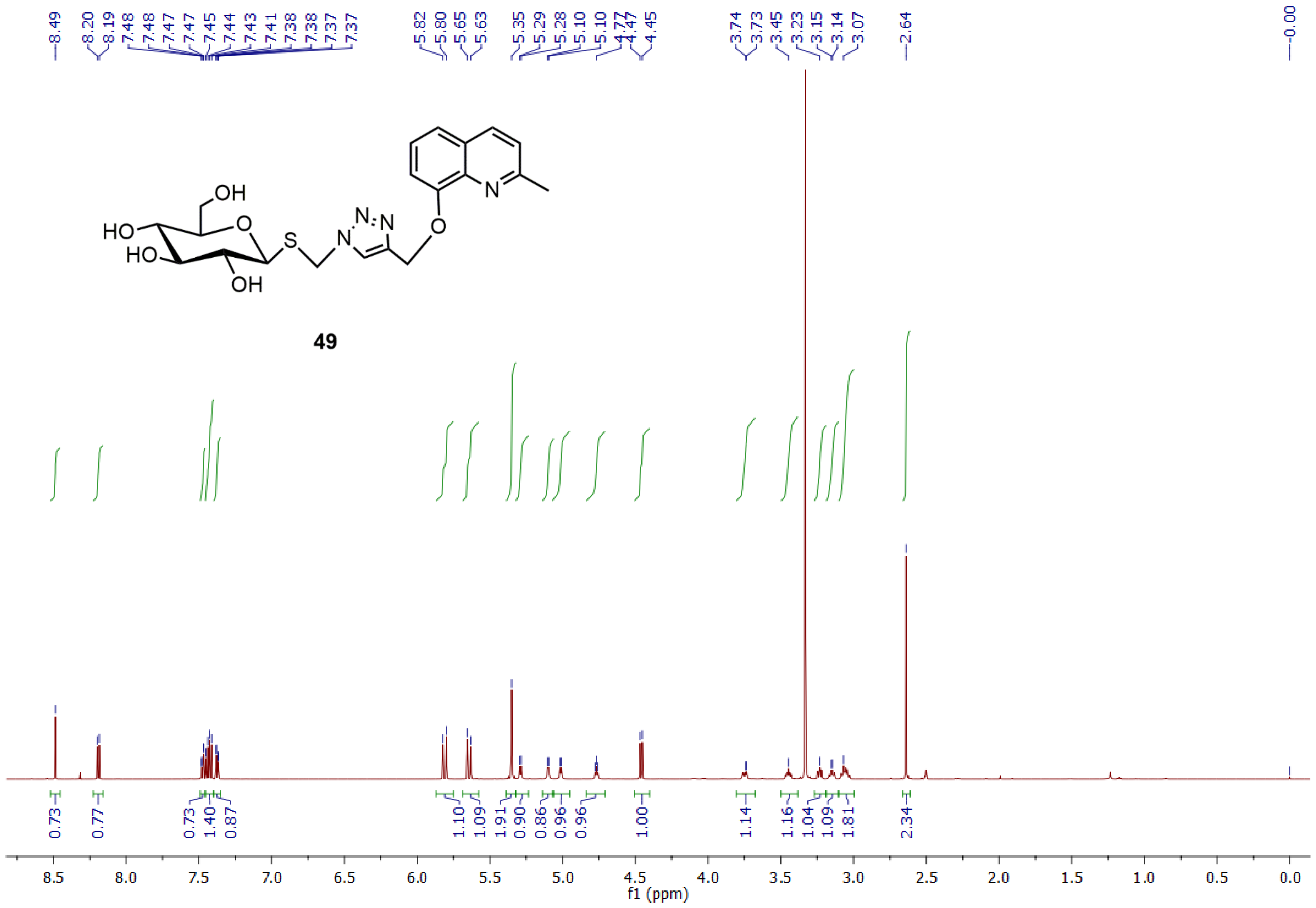

Fig. S82: ^{1}H NMR spectrum of compound **49** (400 MHz/DMSO/TMS; δ (ppm)).

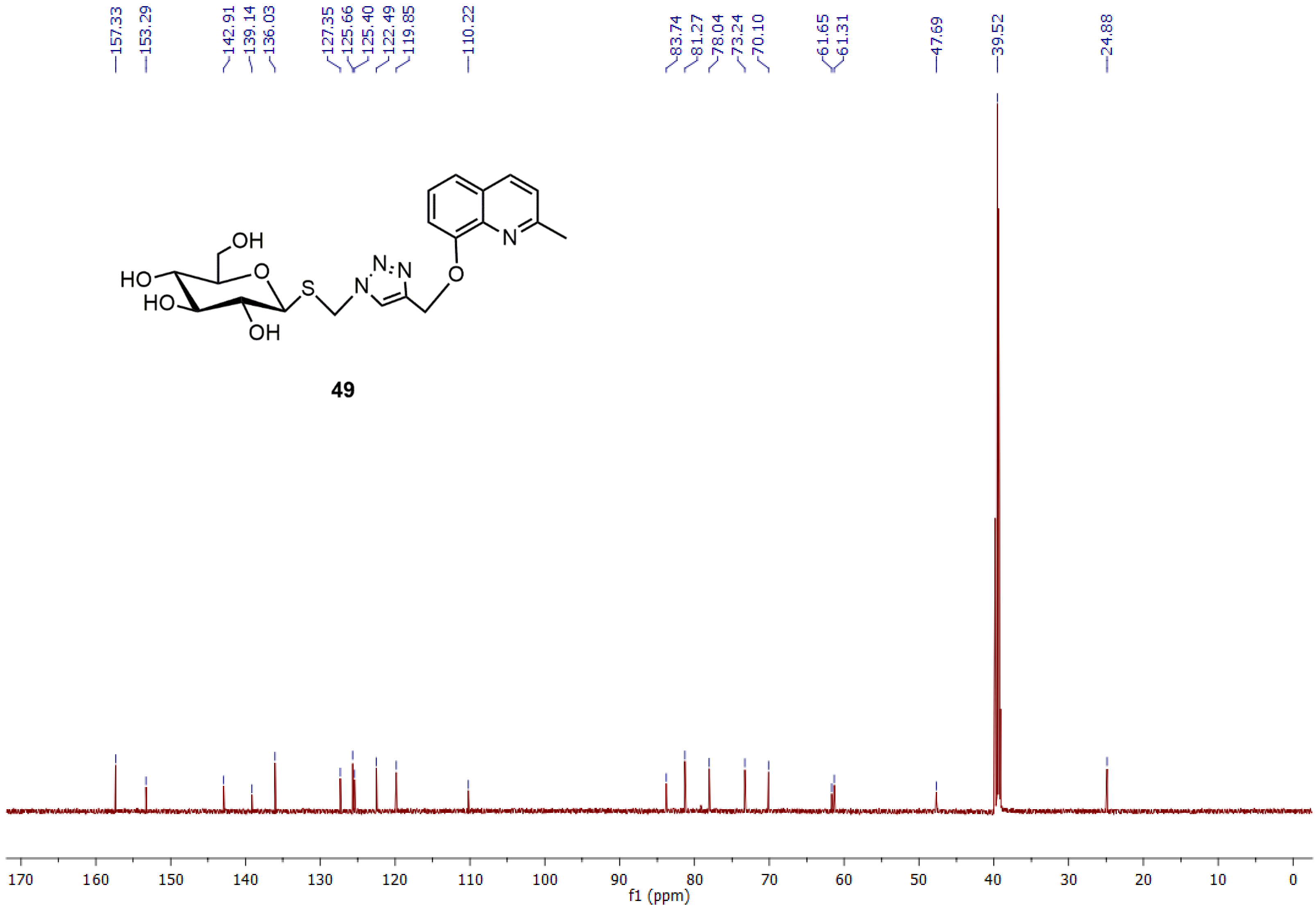

Fig. S83: ^{13}C NMR spectrum of compound **49** (100 MHz/DMSO/TMS; δ (ppm)).

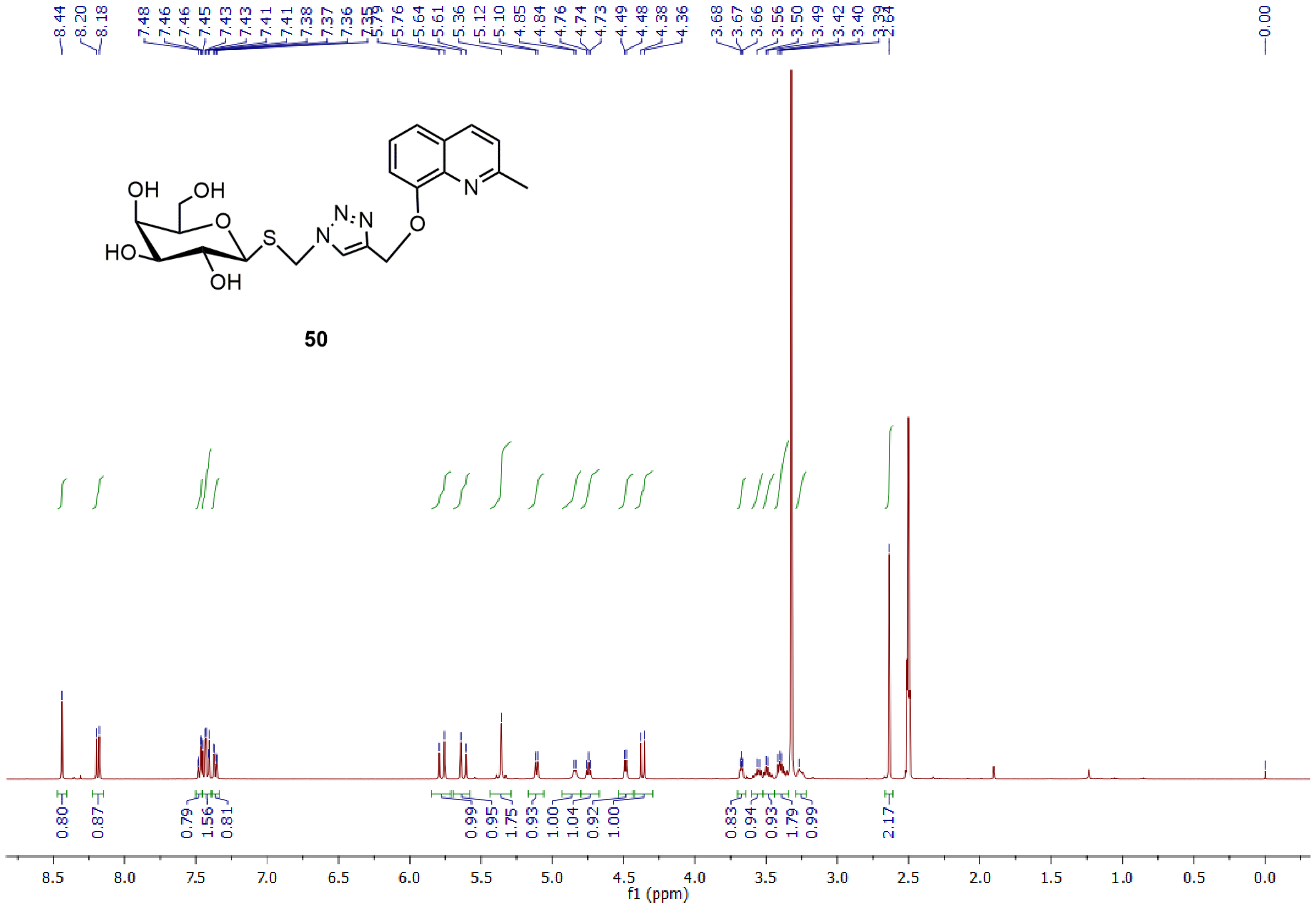

Fig. S84: ^{1}H NMR spectrum of compound **50** (400 MHz/DMSO/TMS; δ (ppm)).

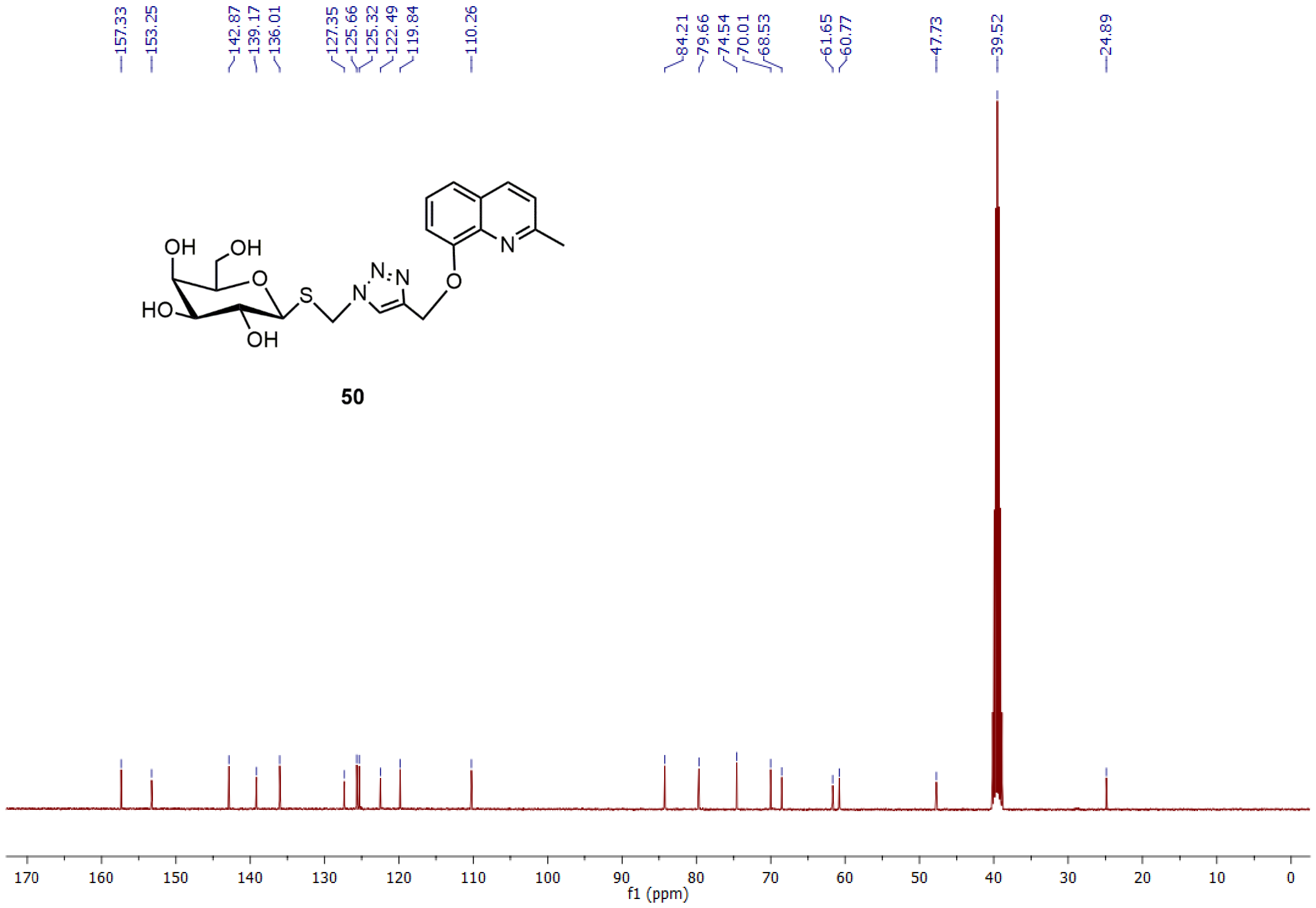

Fig. S85: ^{13}C NMR spectrum of compound **50** (100 MHz/DMSO/TMS; δ (ppm)).

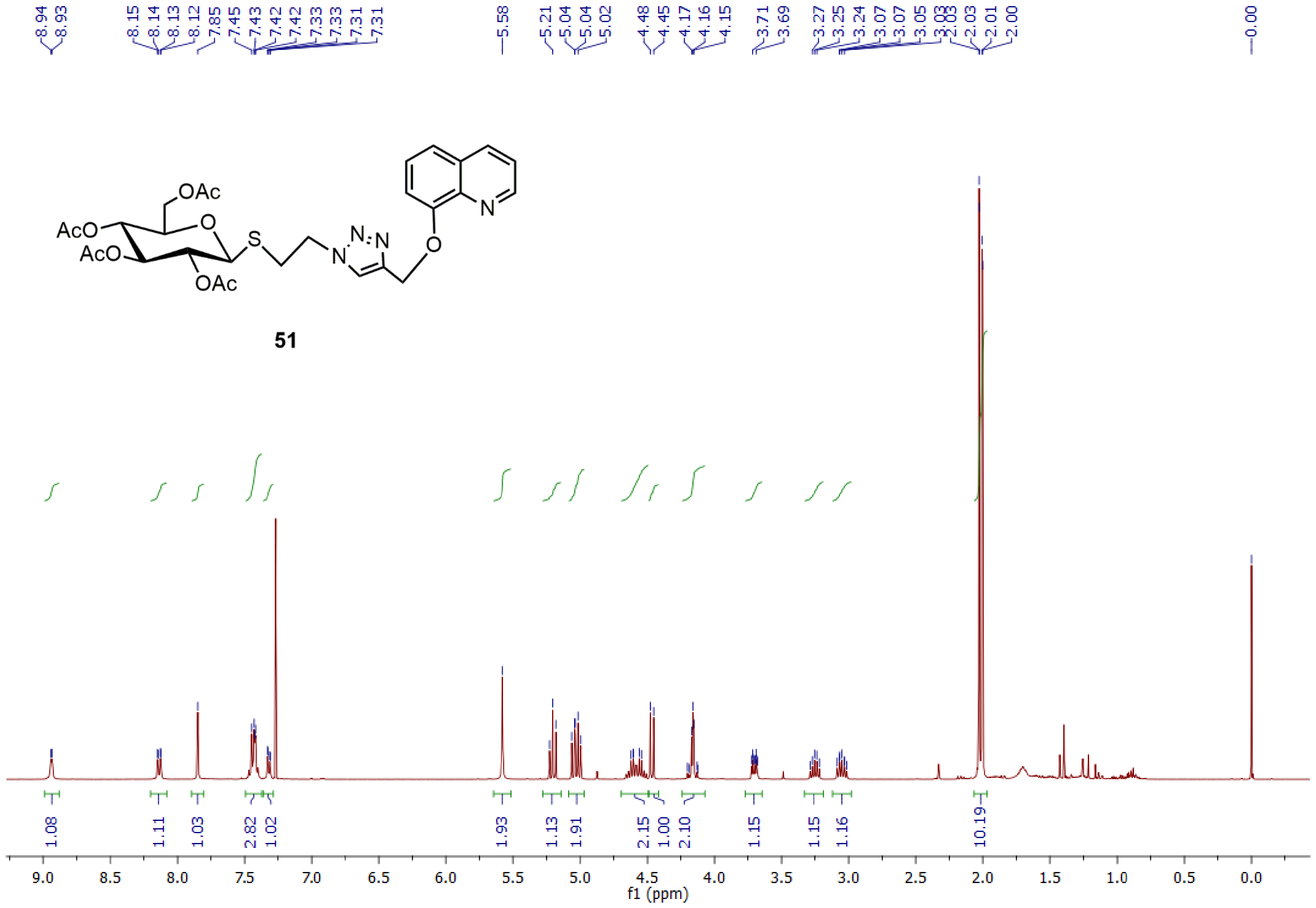

Fig. S86: ^{1}H NMR spectrum of compound **51** (400 MHz/$CDCl_3$/TMS; δ (ppm)).

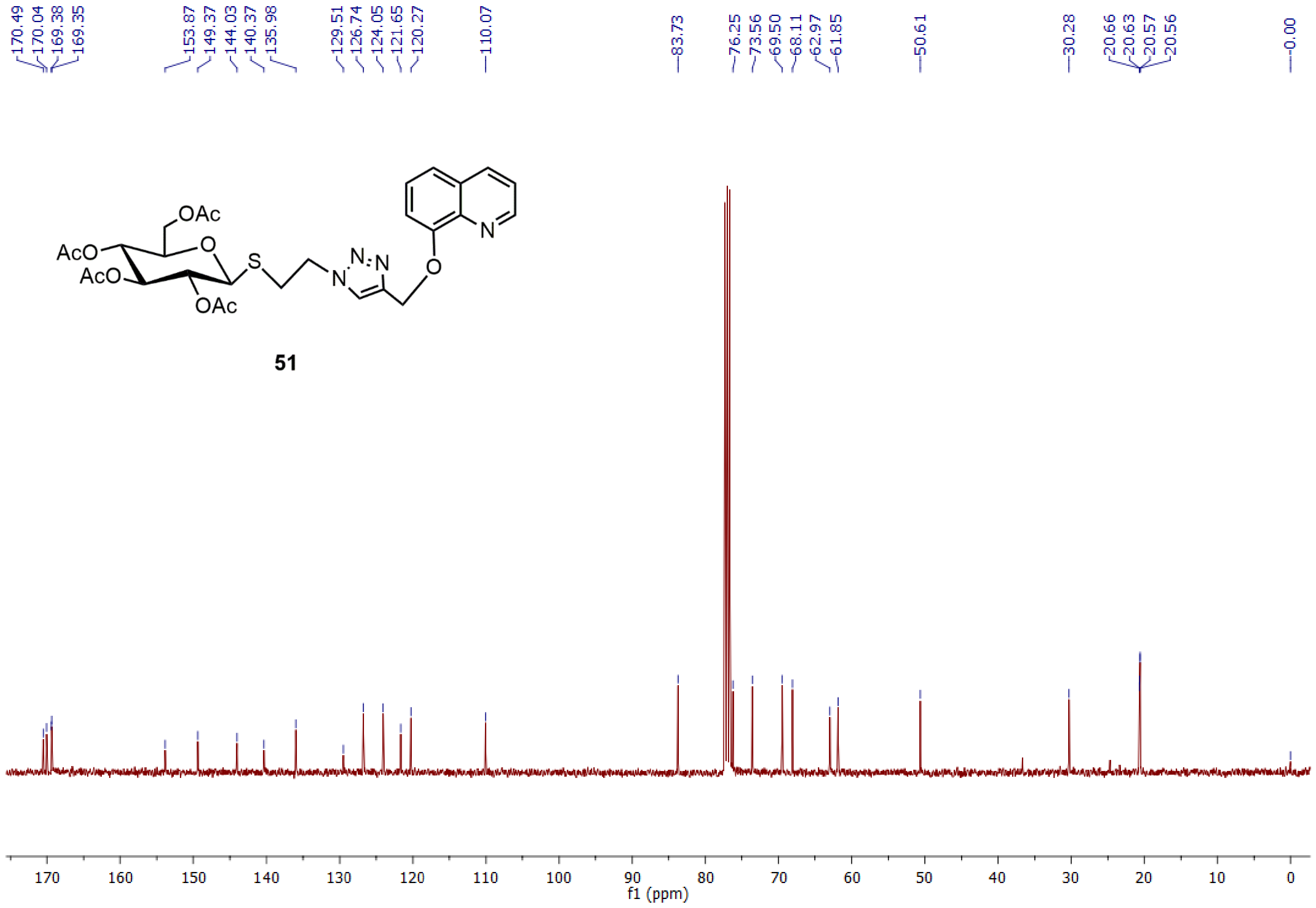

Fig. S87: ^{13}C NMR spectrum of compound **51** (100 MHz/$CDCl_3$/TMS; δ (ppm)).

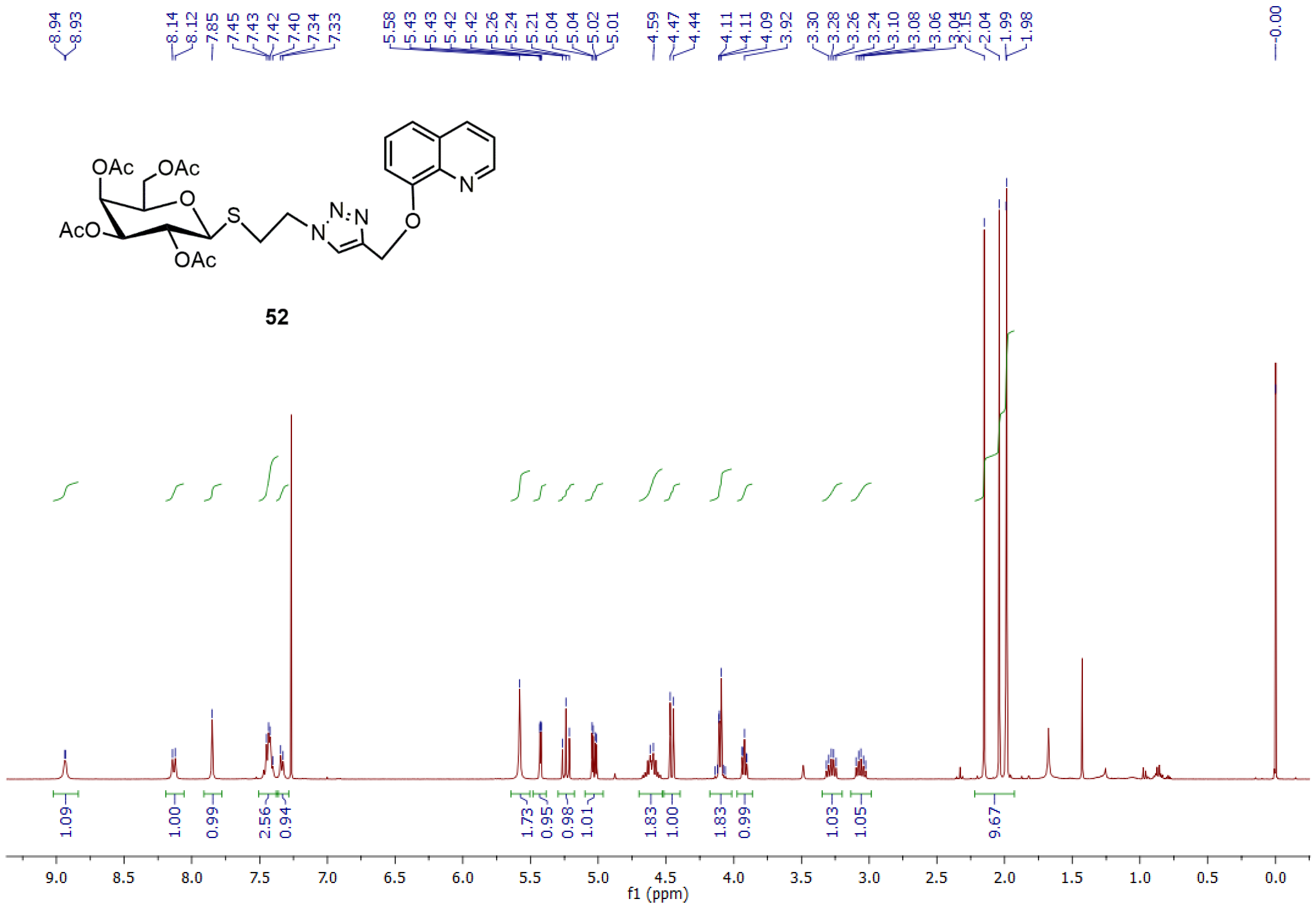

Fig. S88: ^{1}H NMR spectrum of compound **52** (400 MHz/$CDCl_3$/TMS; δ (ppm)).

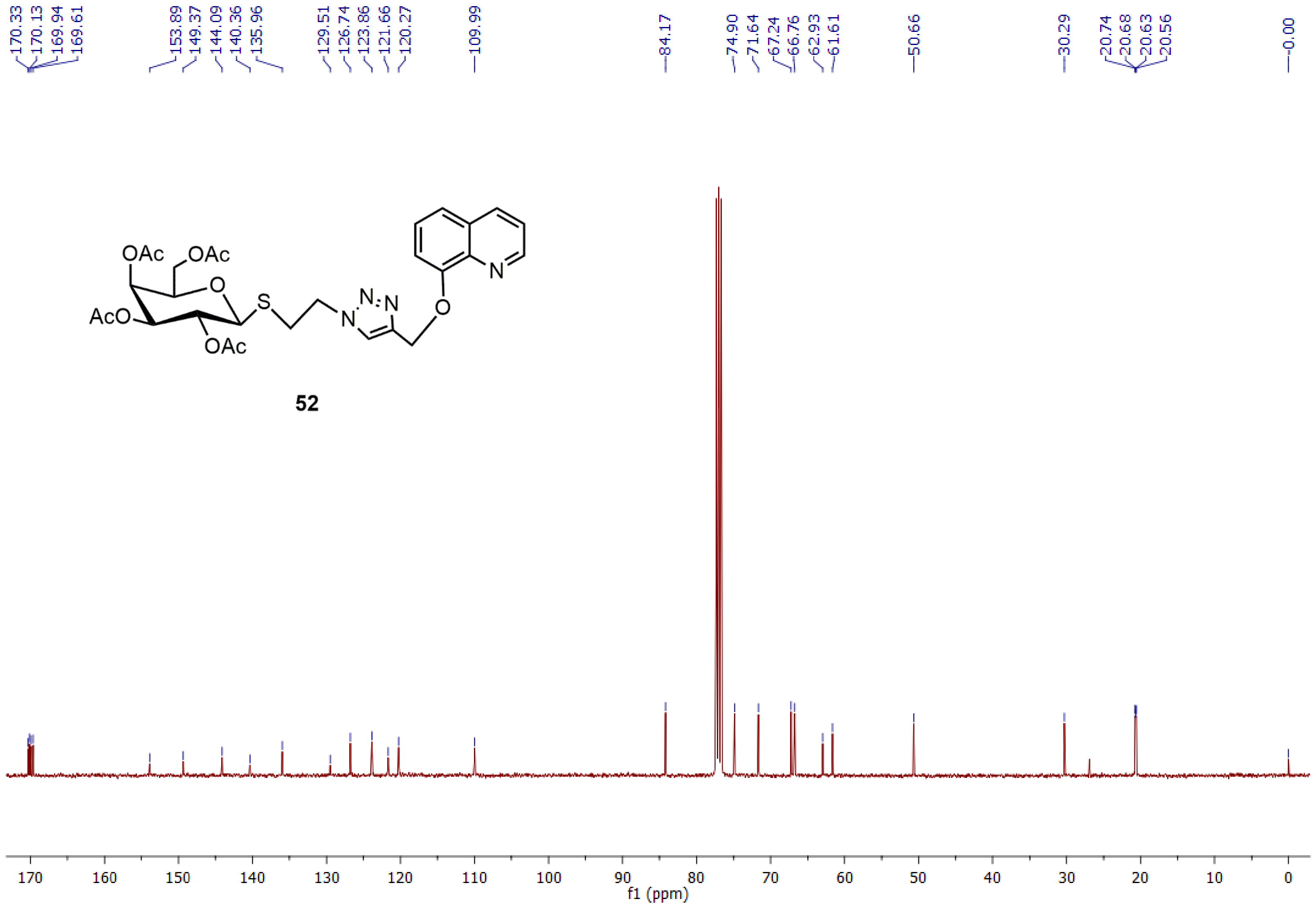

Fig. S89: ^{13}C NMR spectrum of compound **52** (100 MHz/$CDCl_3$/TMS; δ (ppm)).

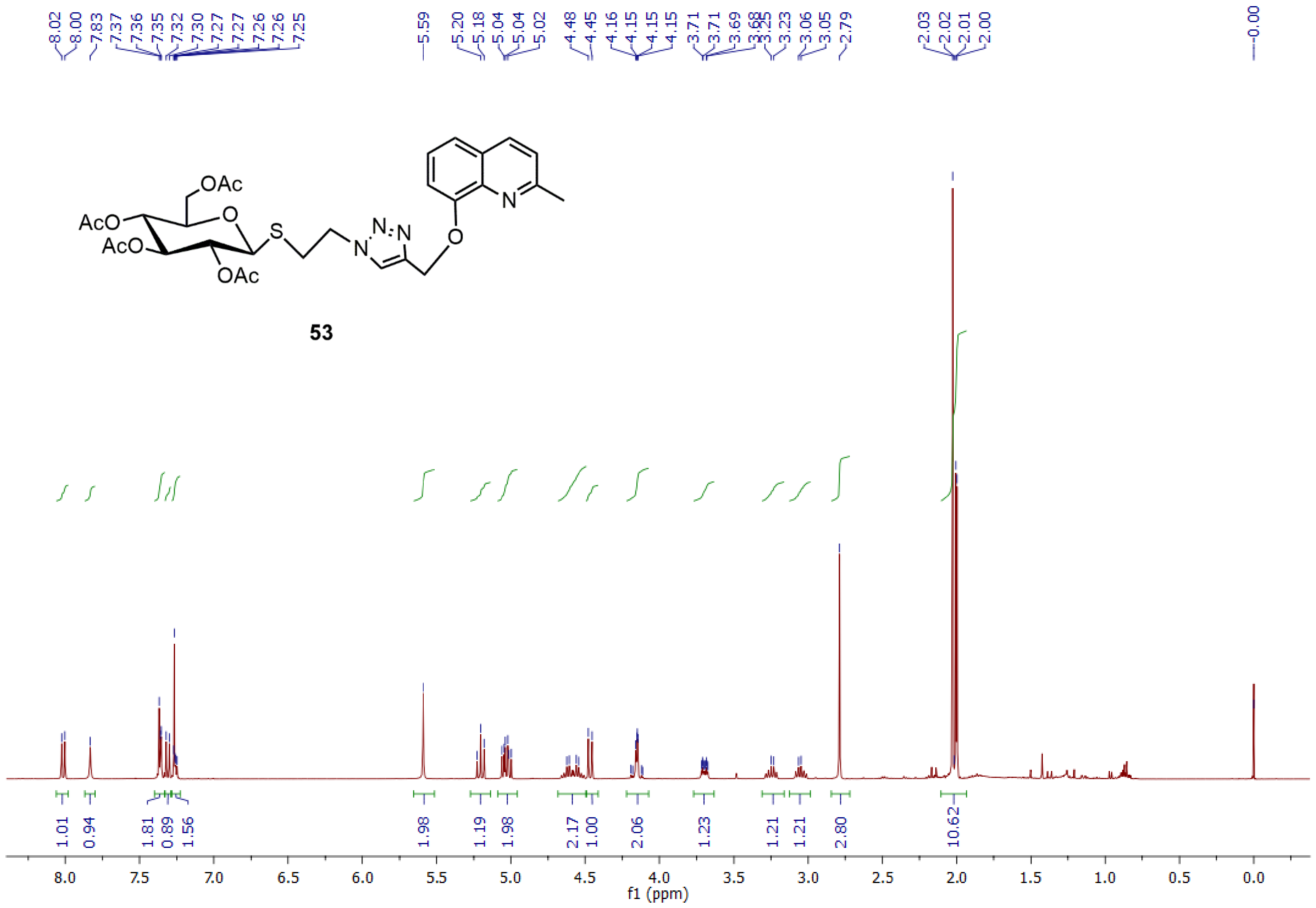

Fig. S90: ^{1}H NMR spectrum of compound **53** (400 MHz/$CDCl_3$/TMS; δ (ppm)).

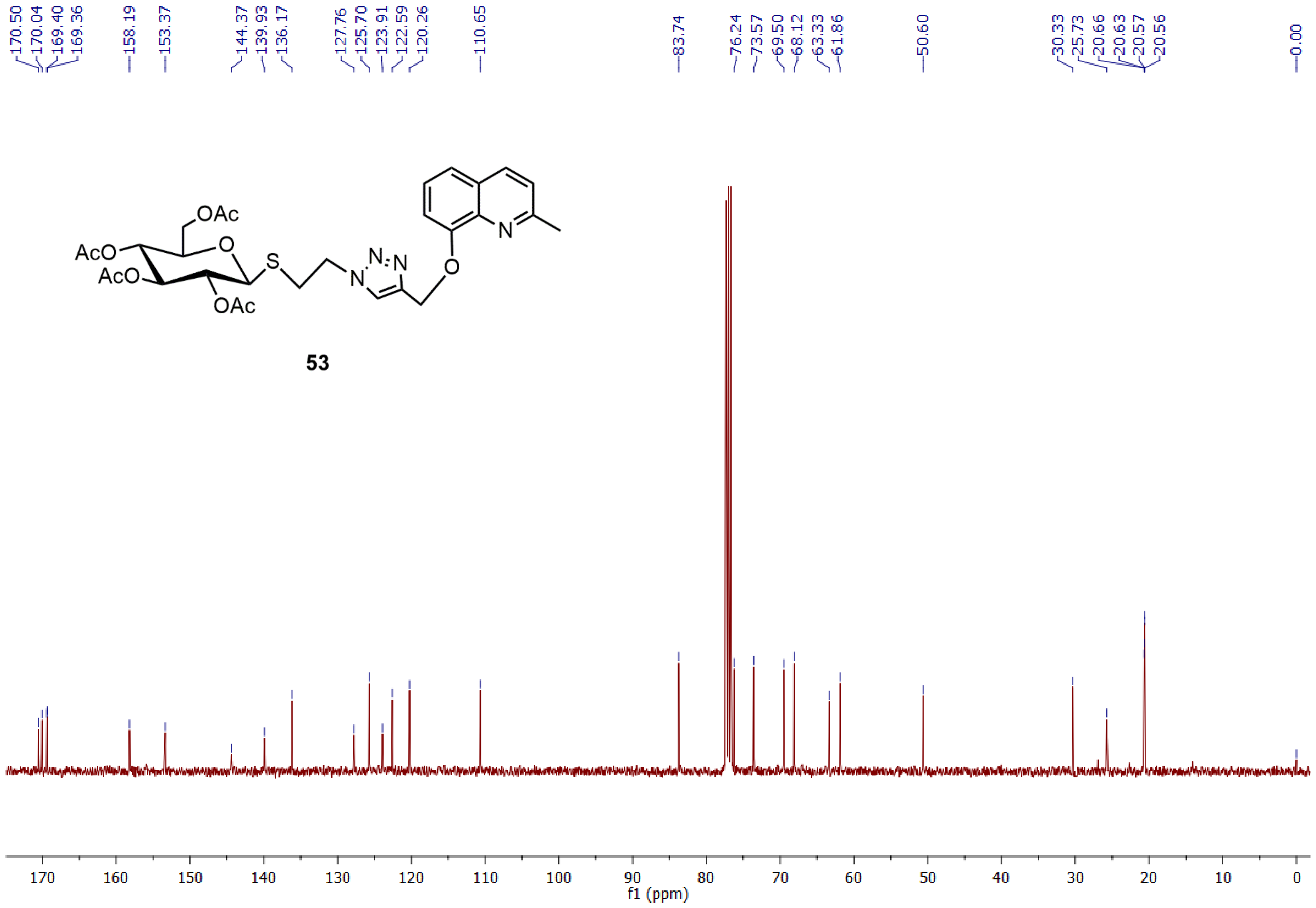

Fig. S91: ^{13}C NMR spectrum of compound **53** (100 MHz/$CDCl_3$/TMS; δ (ppm)).

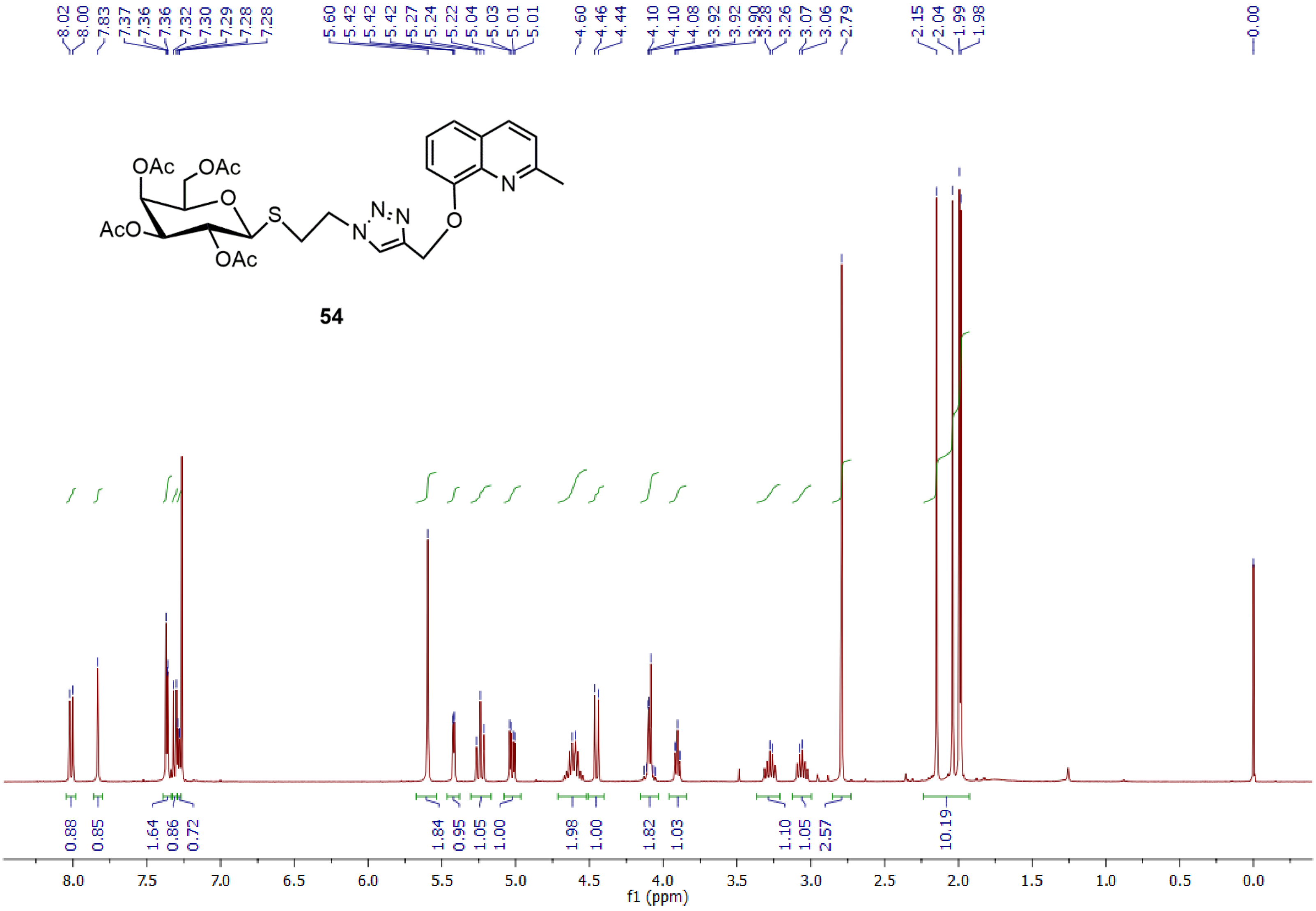

Fig. S92: ^{1}H NMR spectrum of compound **54** (400 MHz/$CDCl_3$/TMS; δ (ppm)).

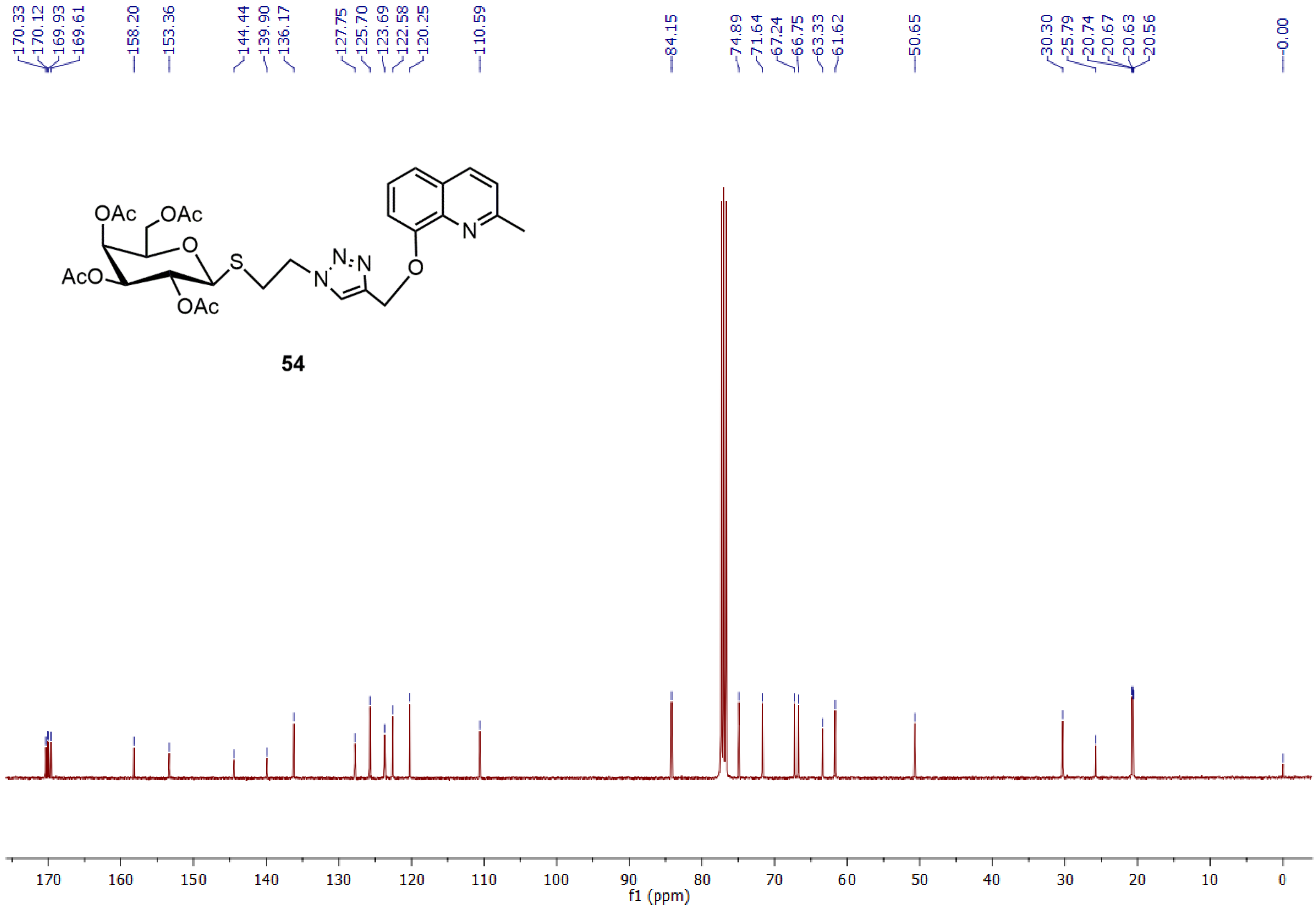

Fig. S93: ^{13}C NMR spectrum of compound **54** (100 MHz/$CDCl_3$/TMS; δ (ppm)).

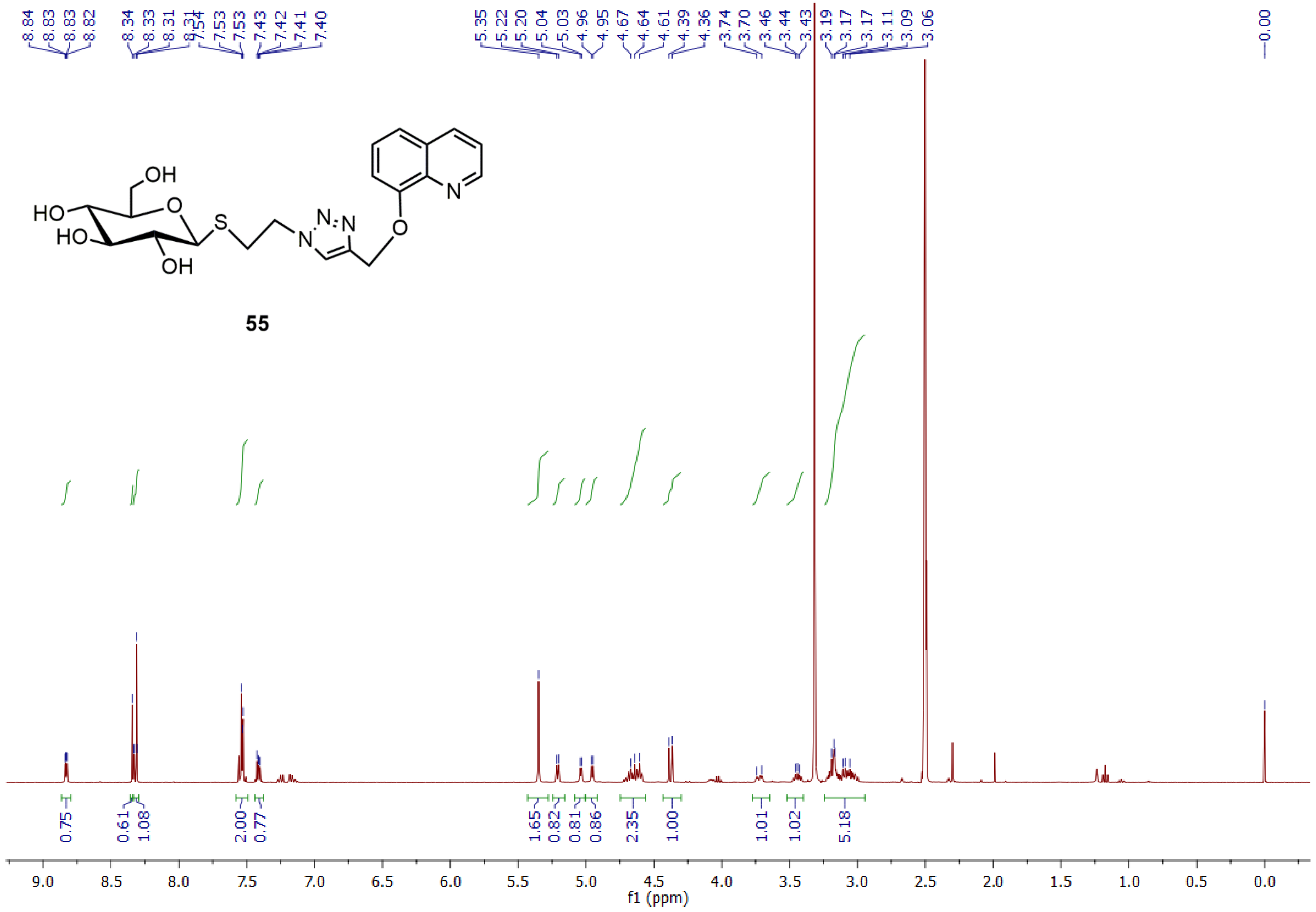

Fig. S94: ^{1}H NMR spectrum of compound **55** (400 MHz/DMSO/TMS; δ (ppm)).

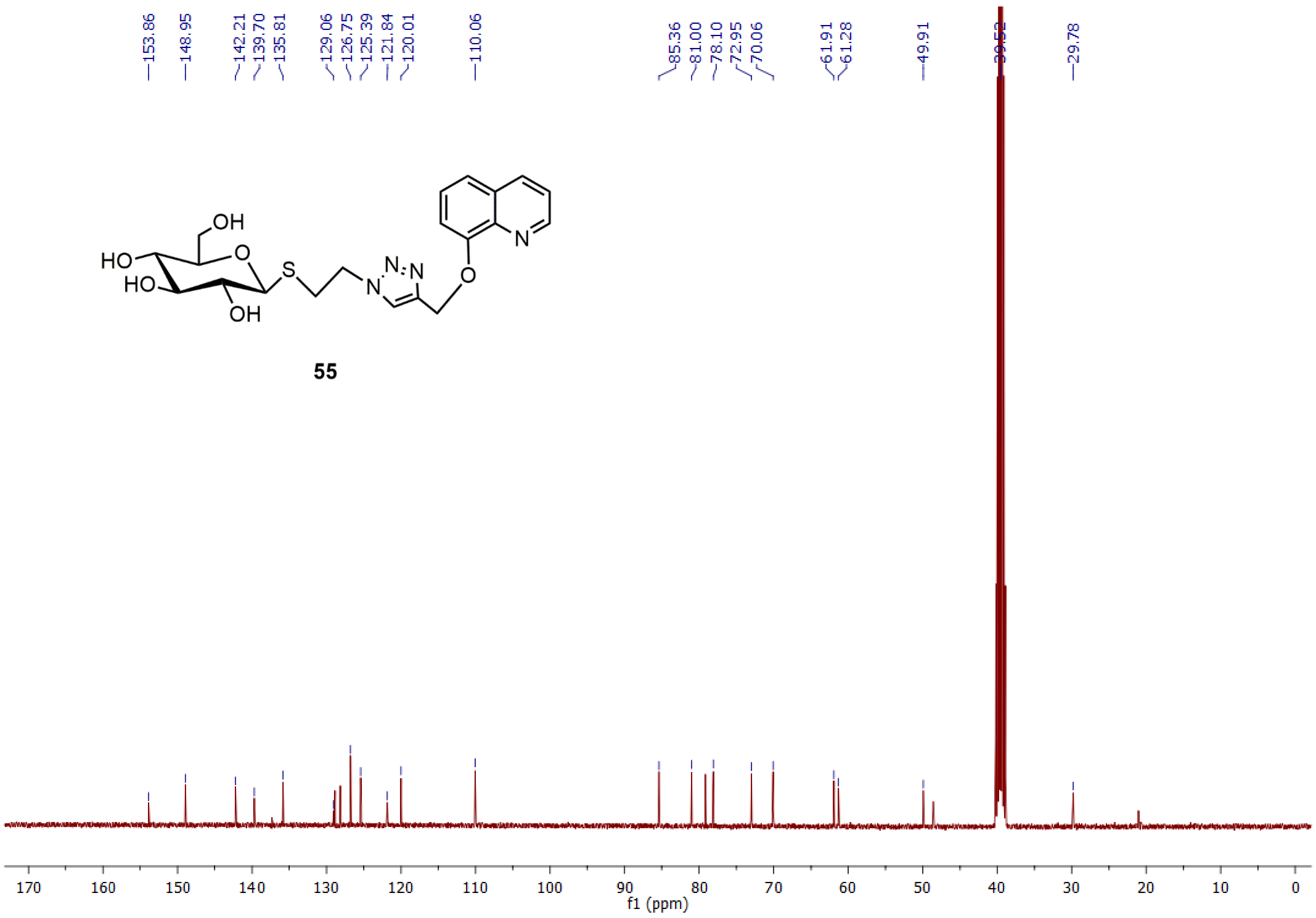

Fig. S95: ^{13}C NMR spectrum of compound **55** (100 MHz/DMSO/TMS; δ (ppm)).

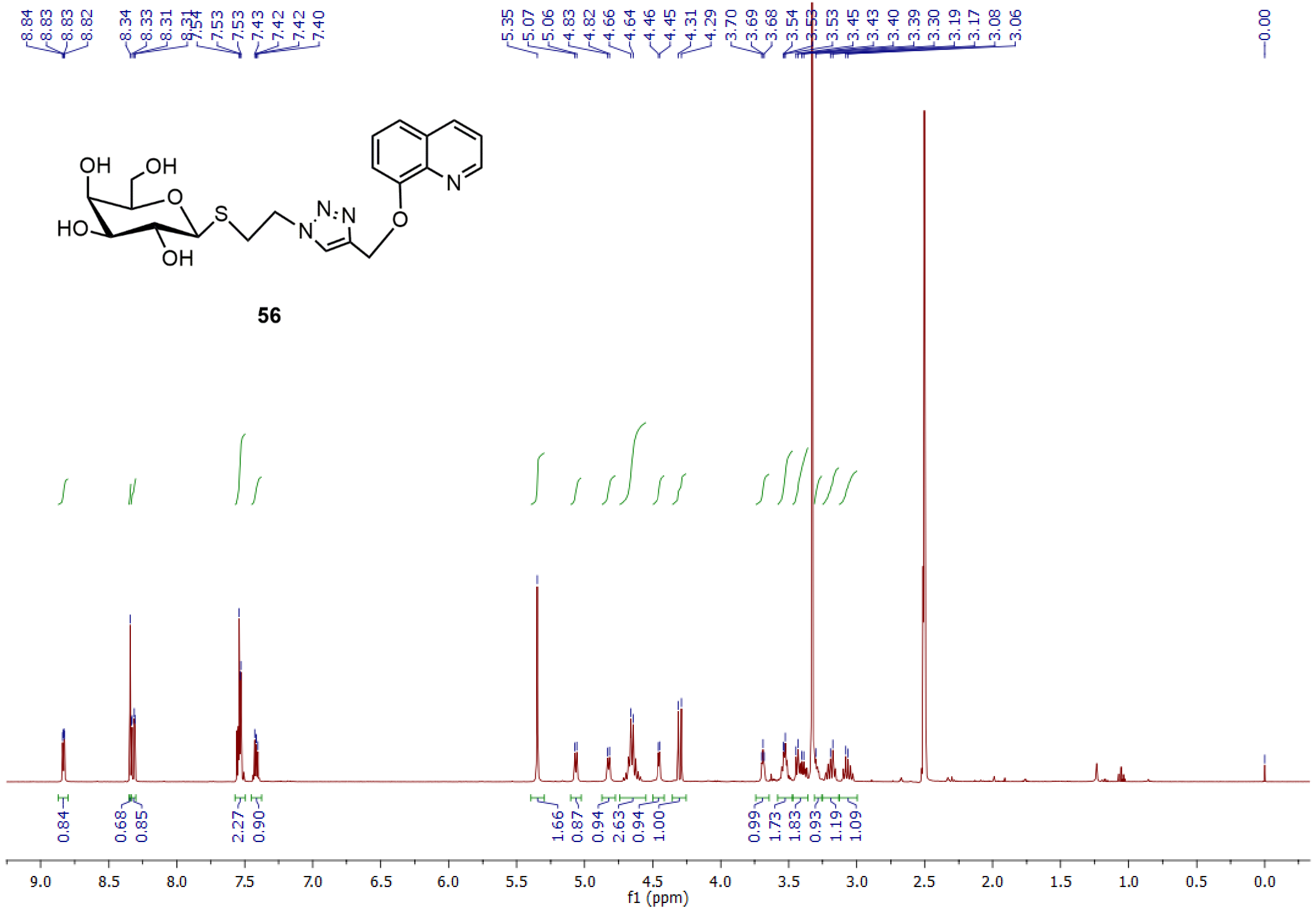

Fig. S96: ^{1}H NMR spectrum of compound **56** (400 MHz/DMSO/TMS; δ (ppm)).

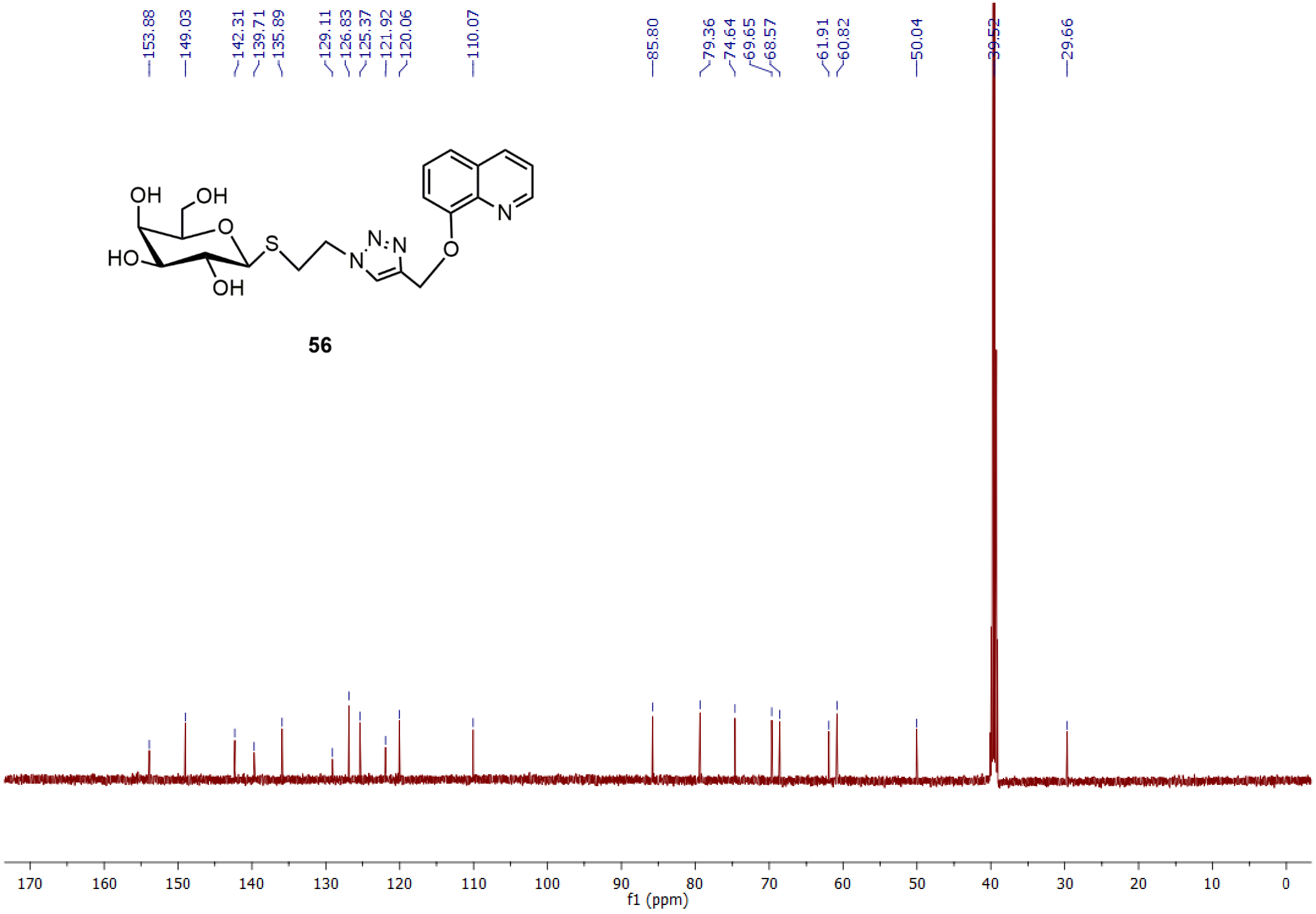

Fig. S97: ^{13}C NMR spectrum of compound **56** (100 MHz/DMSO/TMS; δ (ppm)).

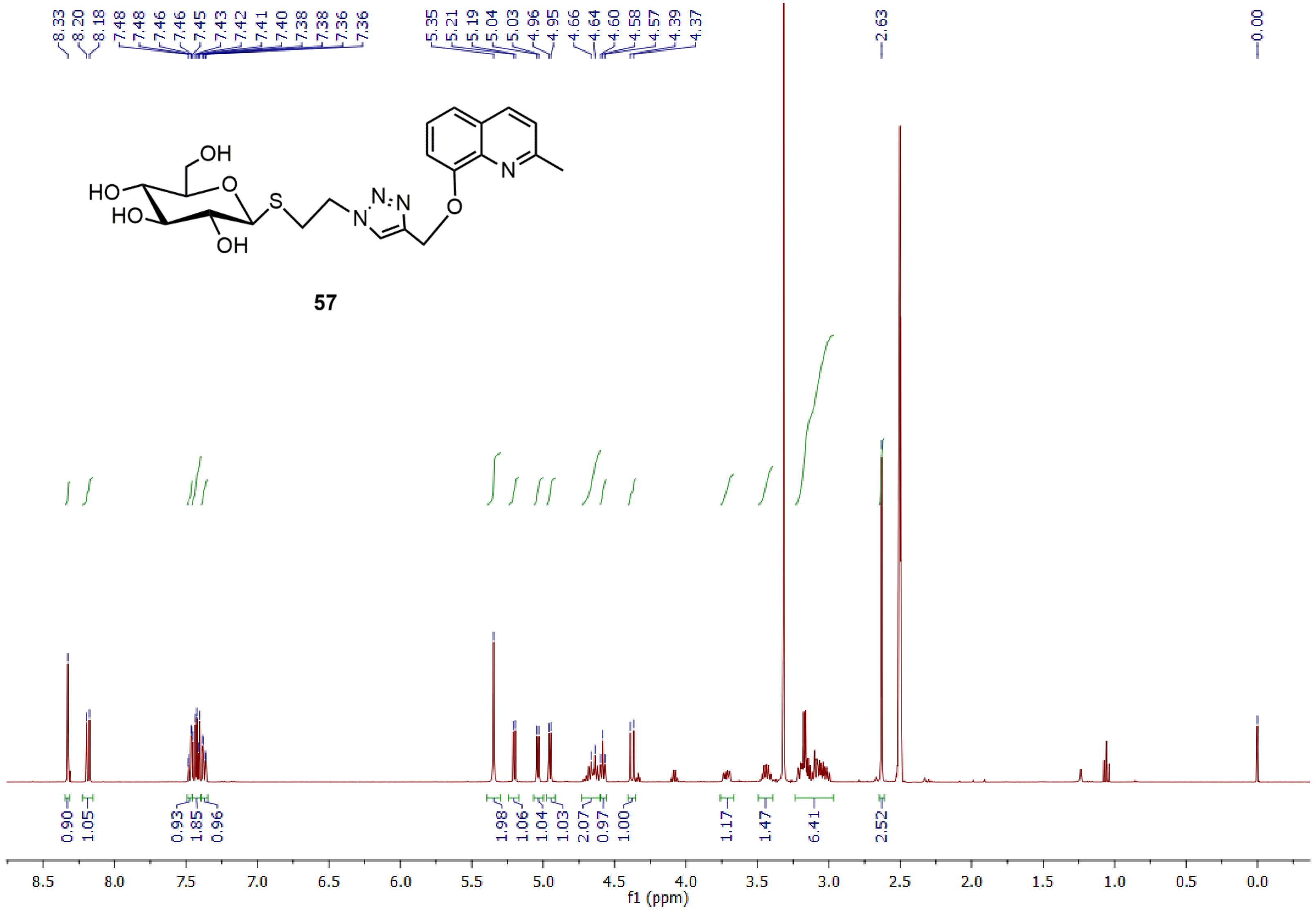

Fig. S98: ^{1}H NMR spectrum of compound **57** (400 MHz/DMSO/TMS; δ (ppm)).

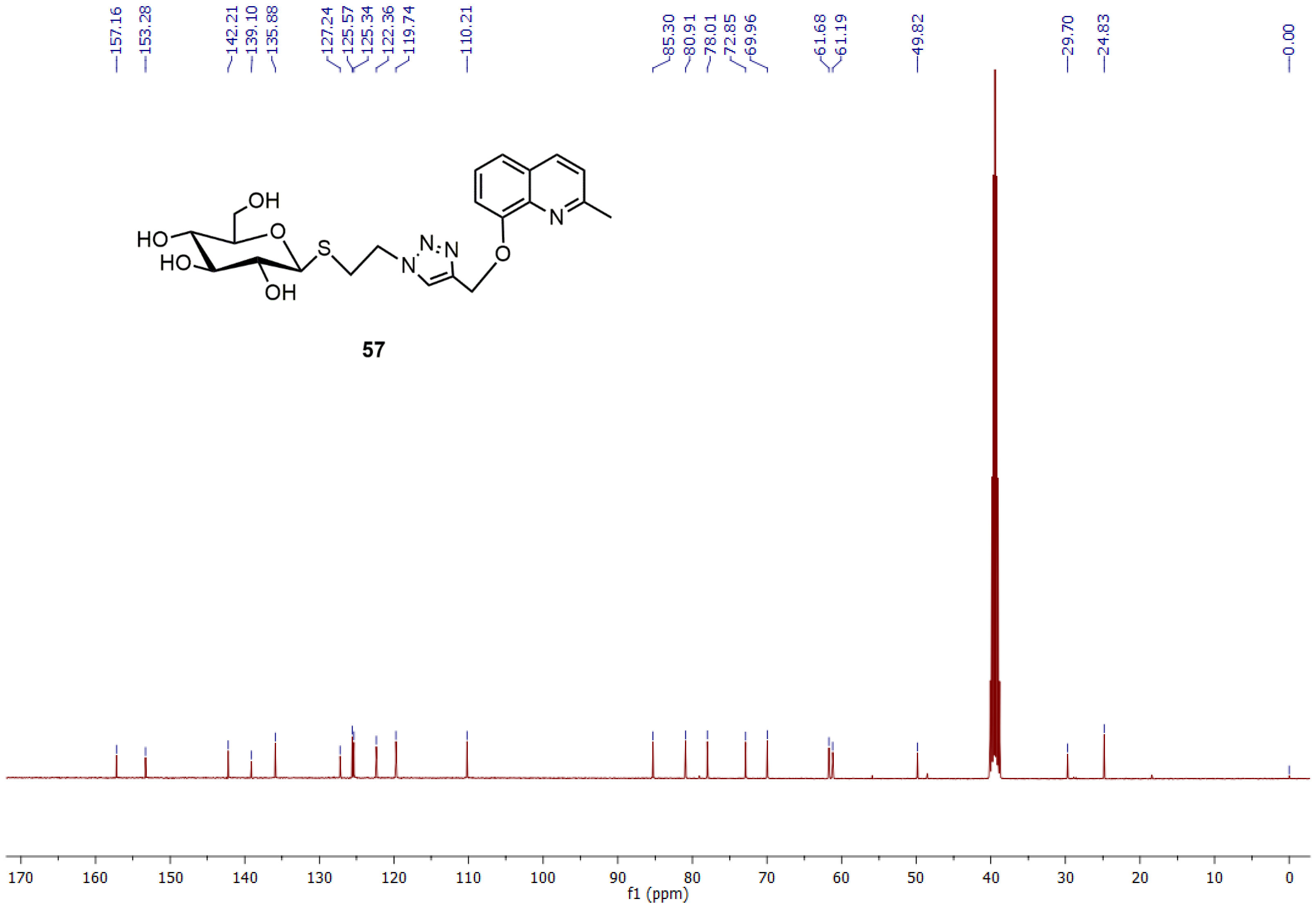

Fig. S99: ^{13}C NMR spectrum of compound **57** (100 MHz/DMSO/TMS; δ (ppm)).

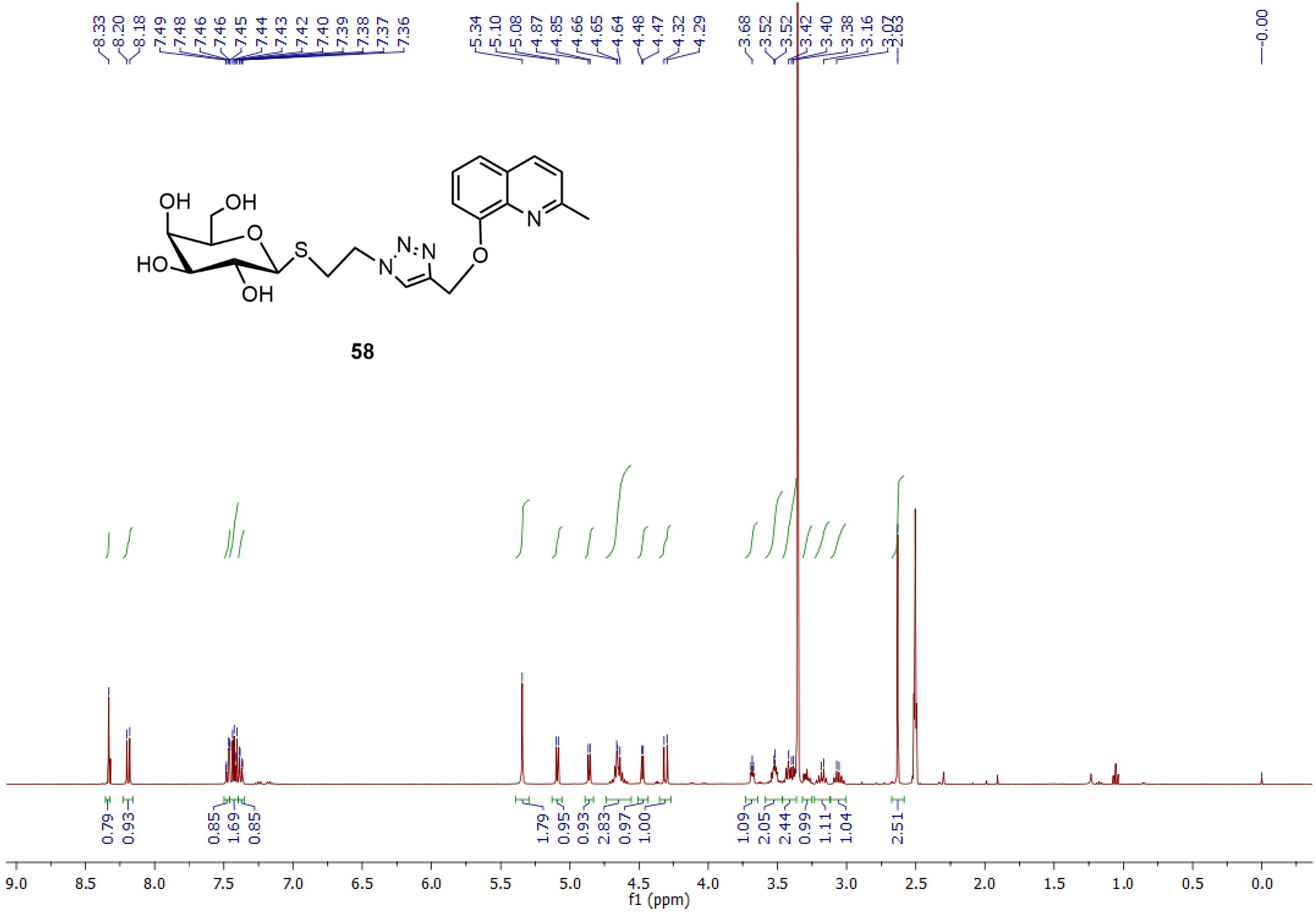

Fig. S100: ^{1}H NMR spectrum of compound **58** (400 MHz/DMSO/TMS; δ (ppm)).

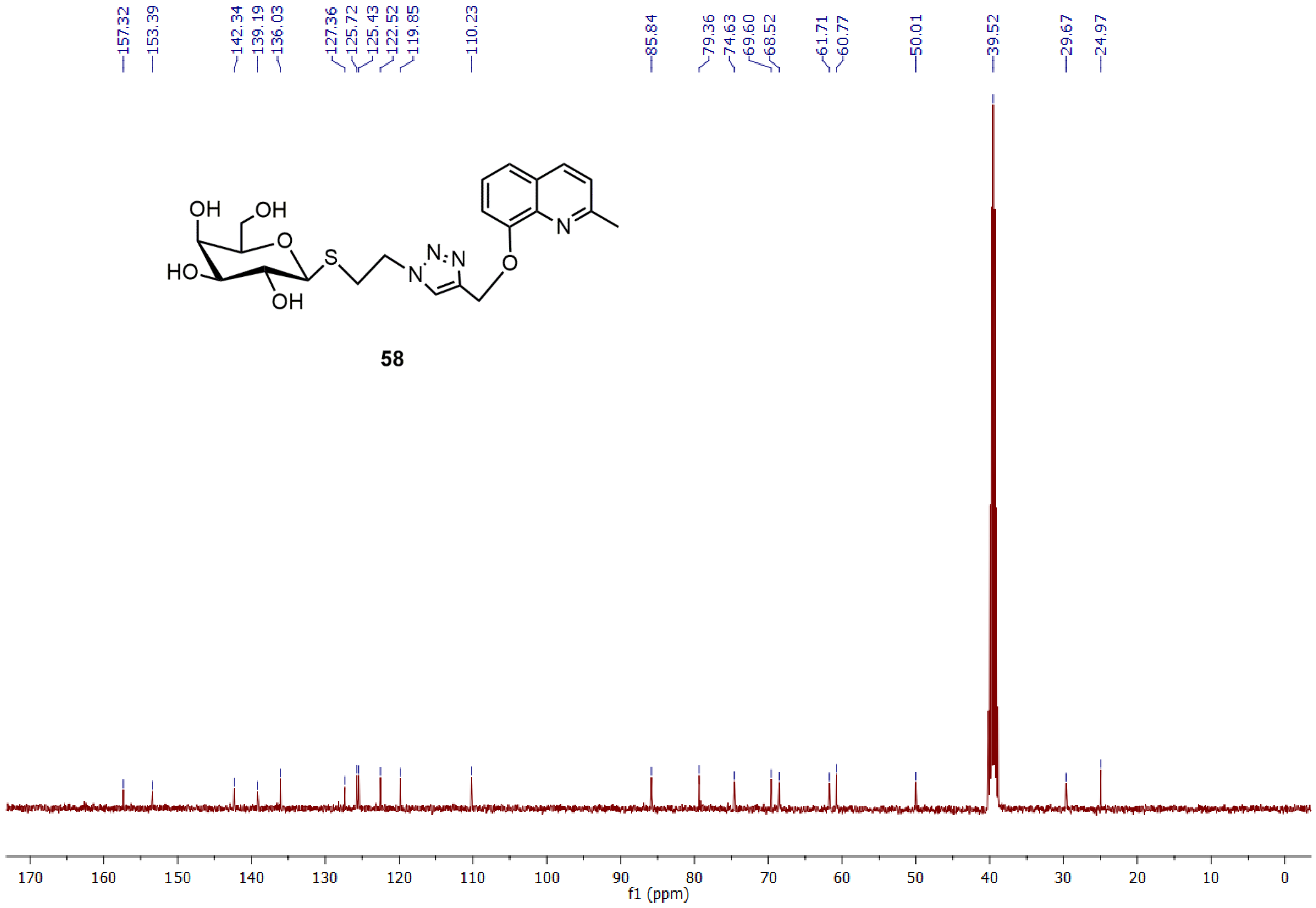

Fig. S101: ^{13}C NMR spectrum of compound **58** (100 MHz/DMSO/TMS; δ (ppm)).

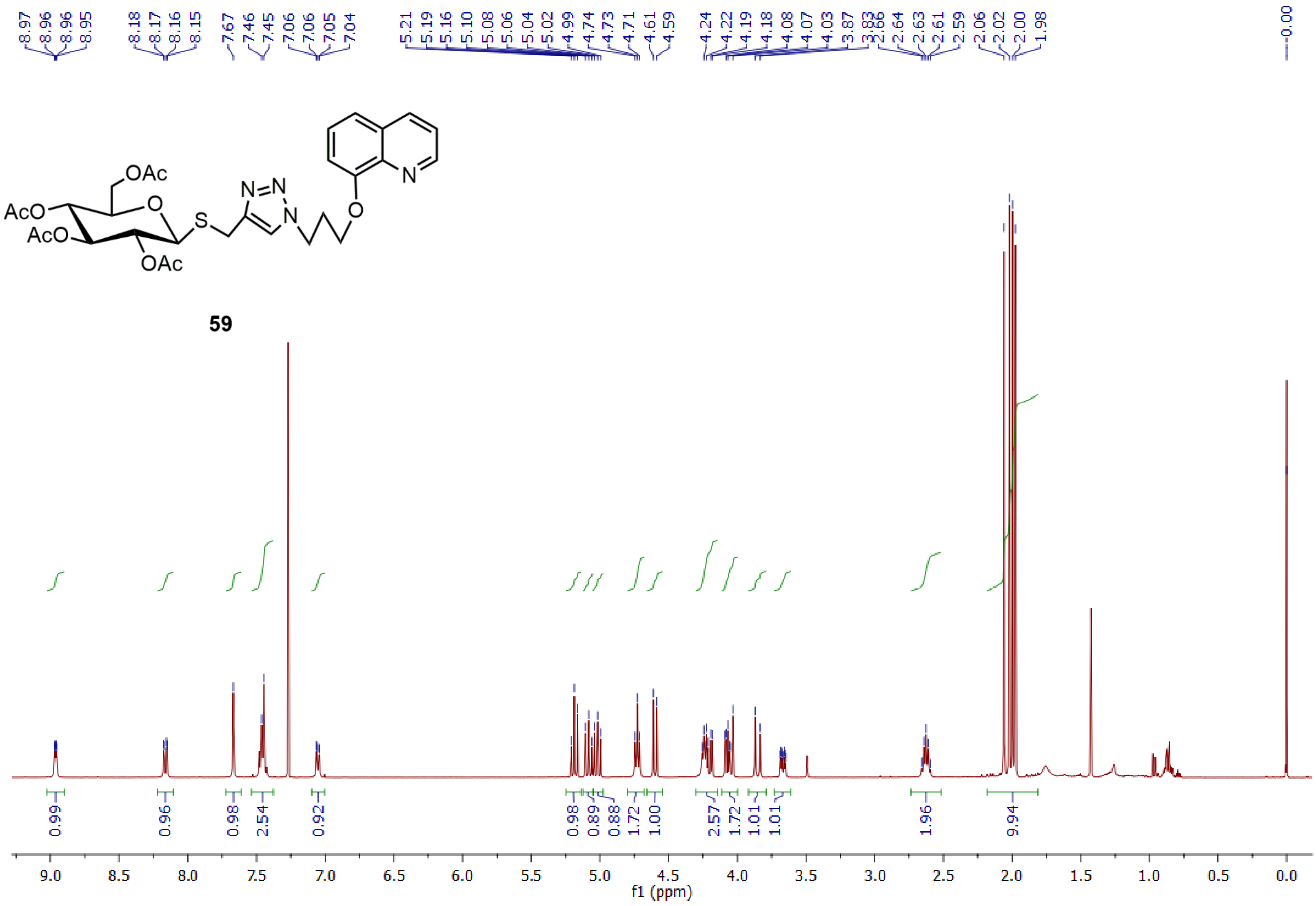

Fig. S102: ^{1}H NMR spectrum of compound **59** (400 MHz/$CDCl_3$/TMS; δ (ppm)).

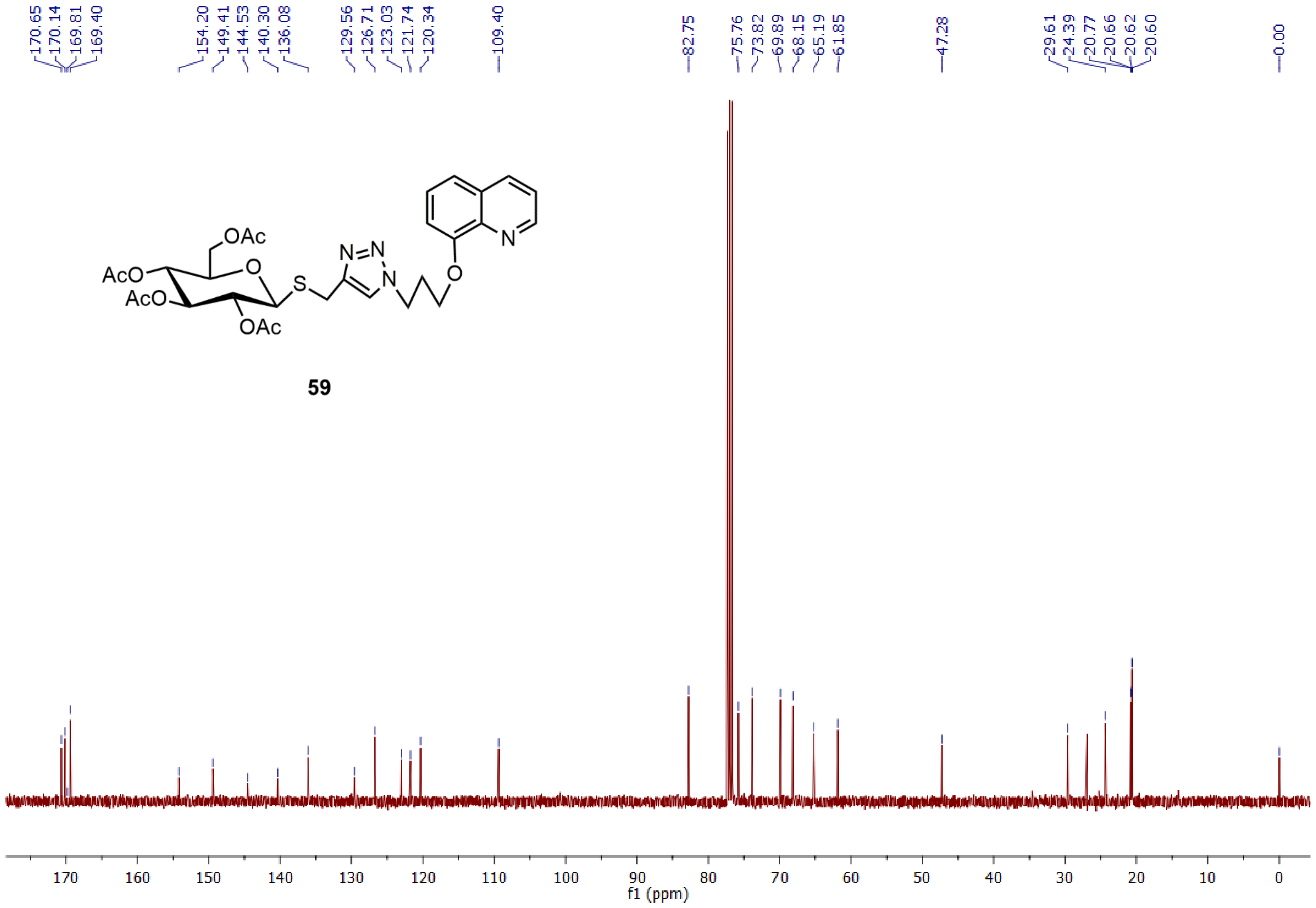

Fig. S103: ^{13}C NMR spectrum of compound **59** (100 MHz/$CDCl_3$/TMS; δ (ppm)).

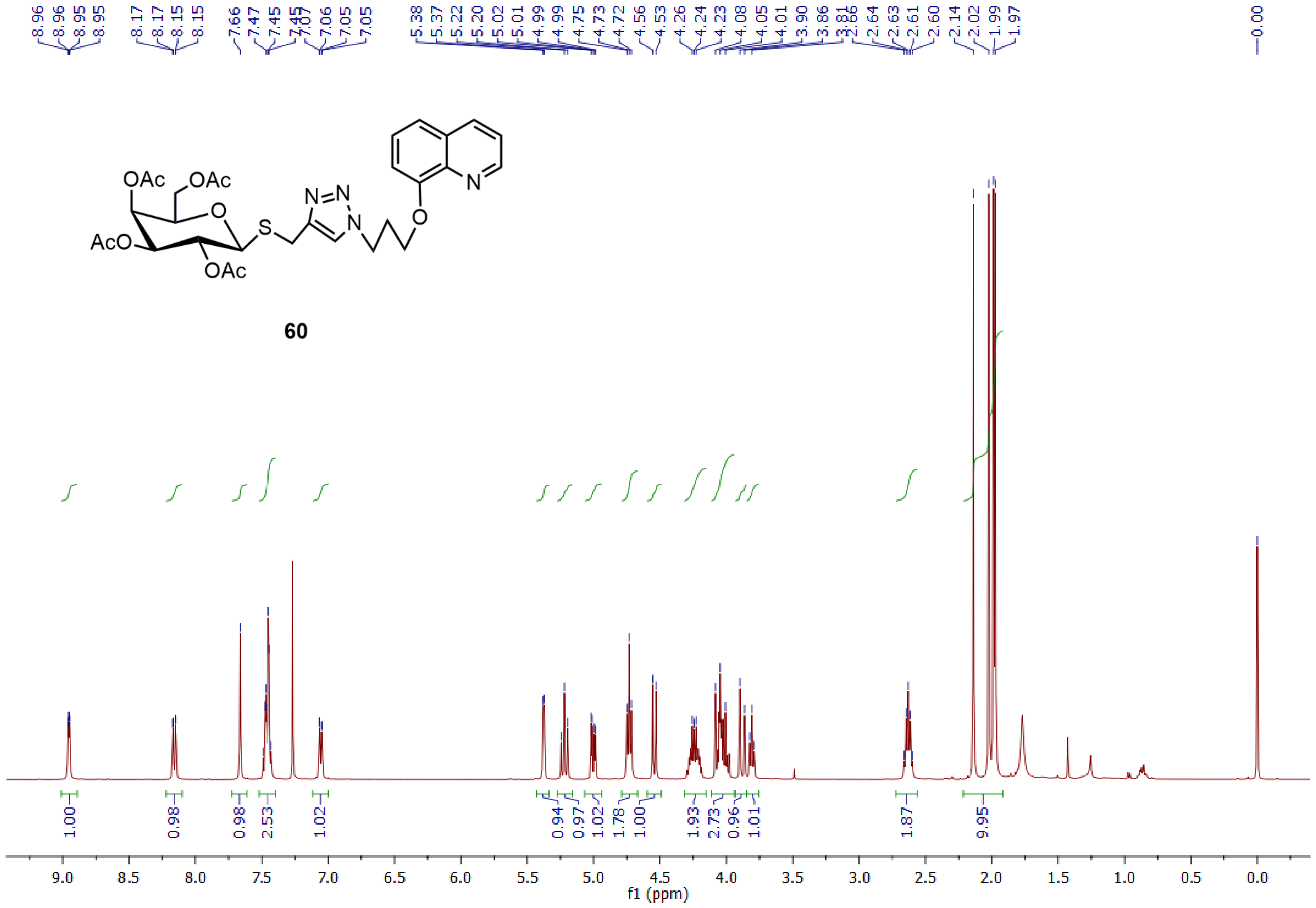

Fig. S104: ^{1}H NMR spectrum of compound **60** (400 MHz/$CDCl_3$/TMS; δ (ppm)).

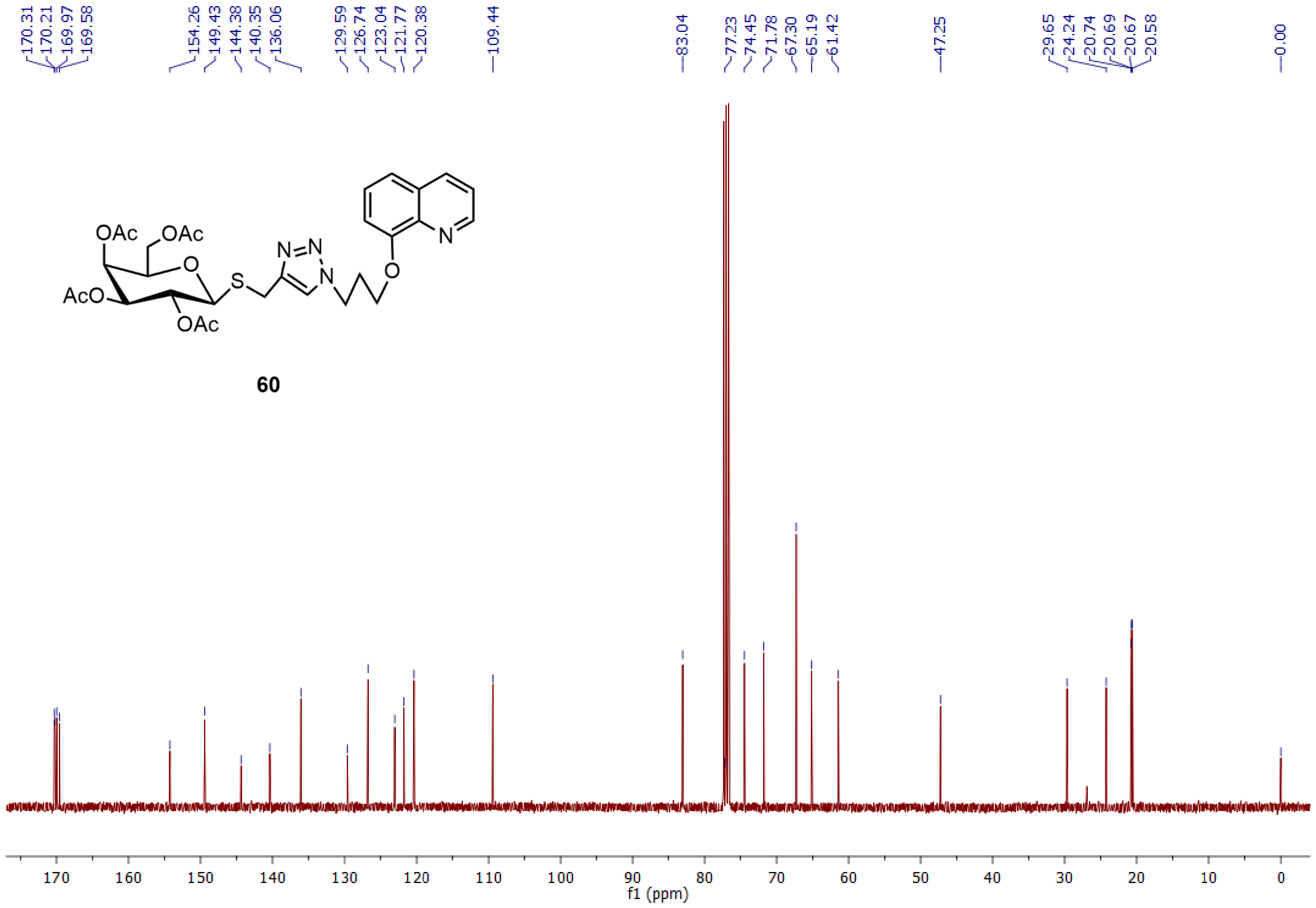

Fig. S105: ^{13}C NMR spectrum of compound **60** (100 MHz/$CDCl_3$/TMS; δ (ppm)).

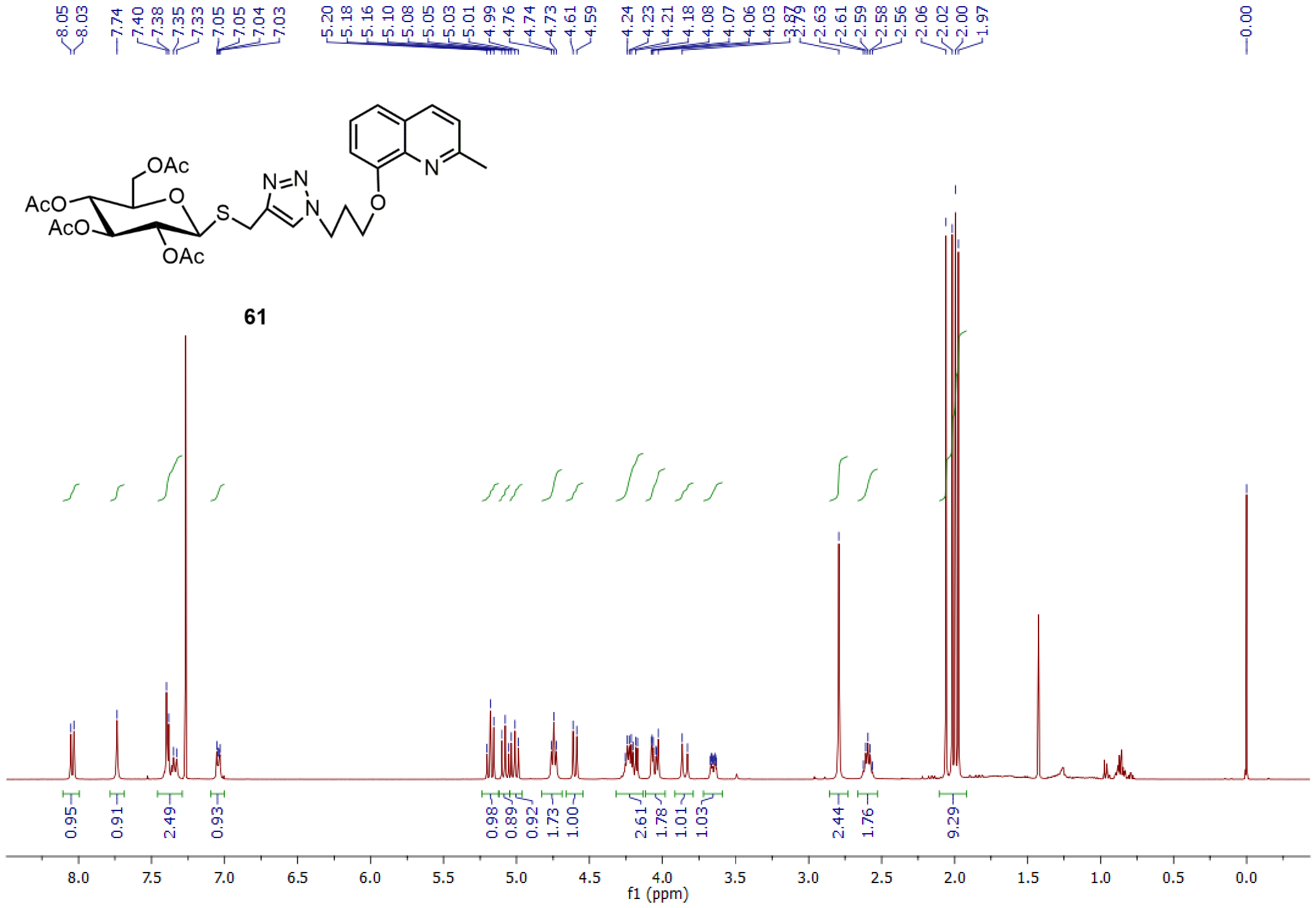

Fig. S106: ^{1}H NMR spectrum of compound **61** (400 MHz/CDCl₃/TMS; δ (ppm)).

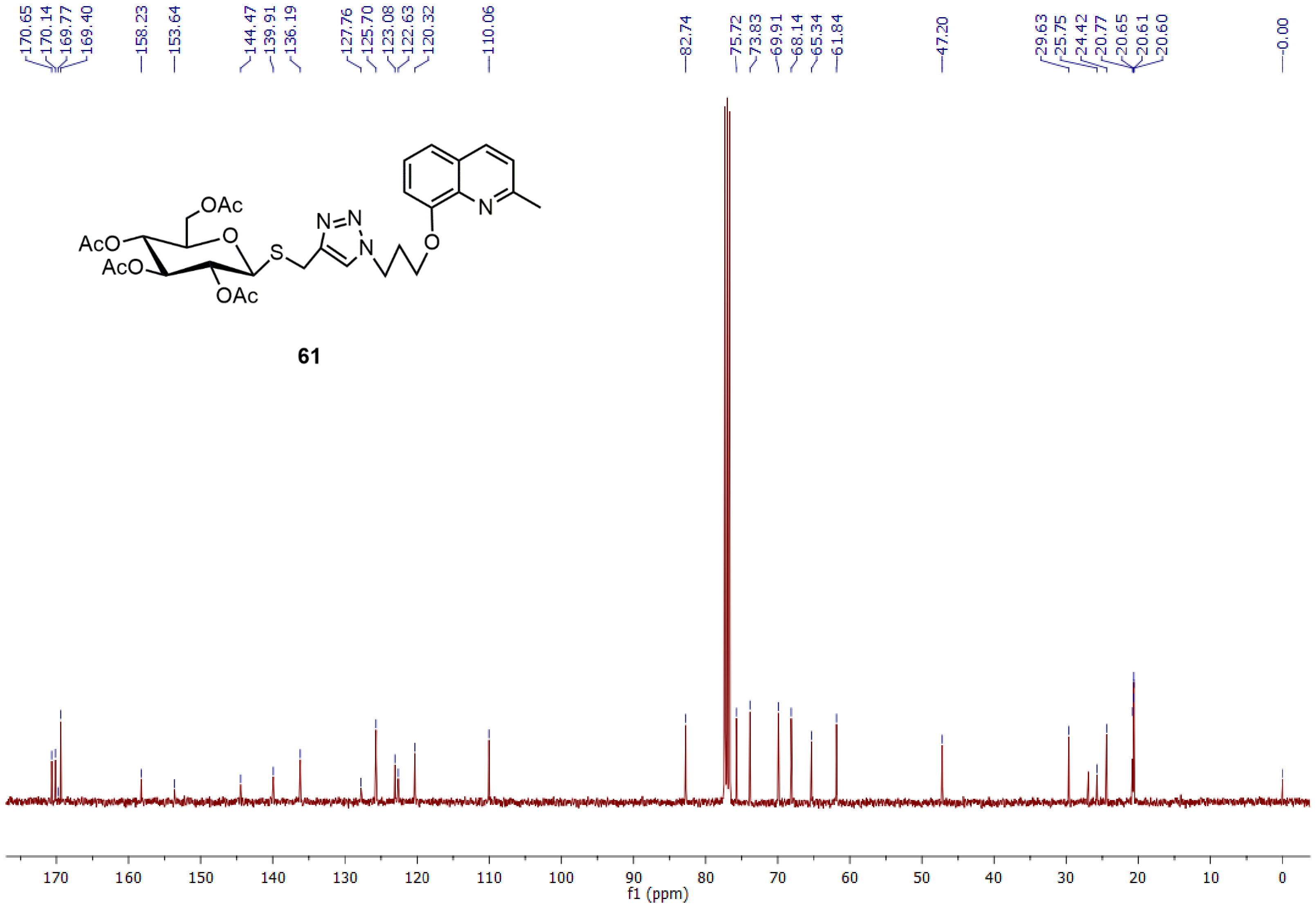

Fig. S107: ^{13}C NMR spectrum of compound **61** (100 MHz/$CDCl_3$/TMS; δ (ppm)).

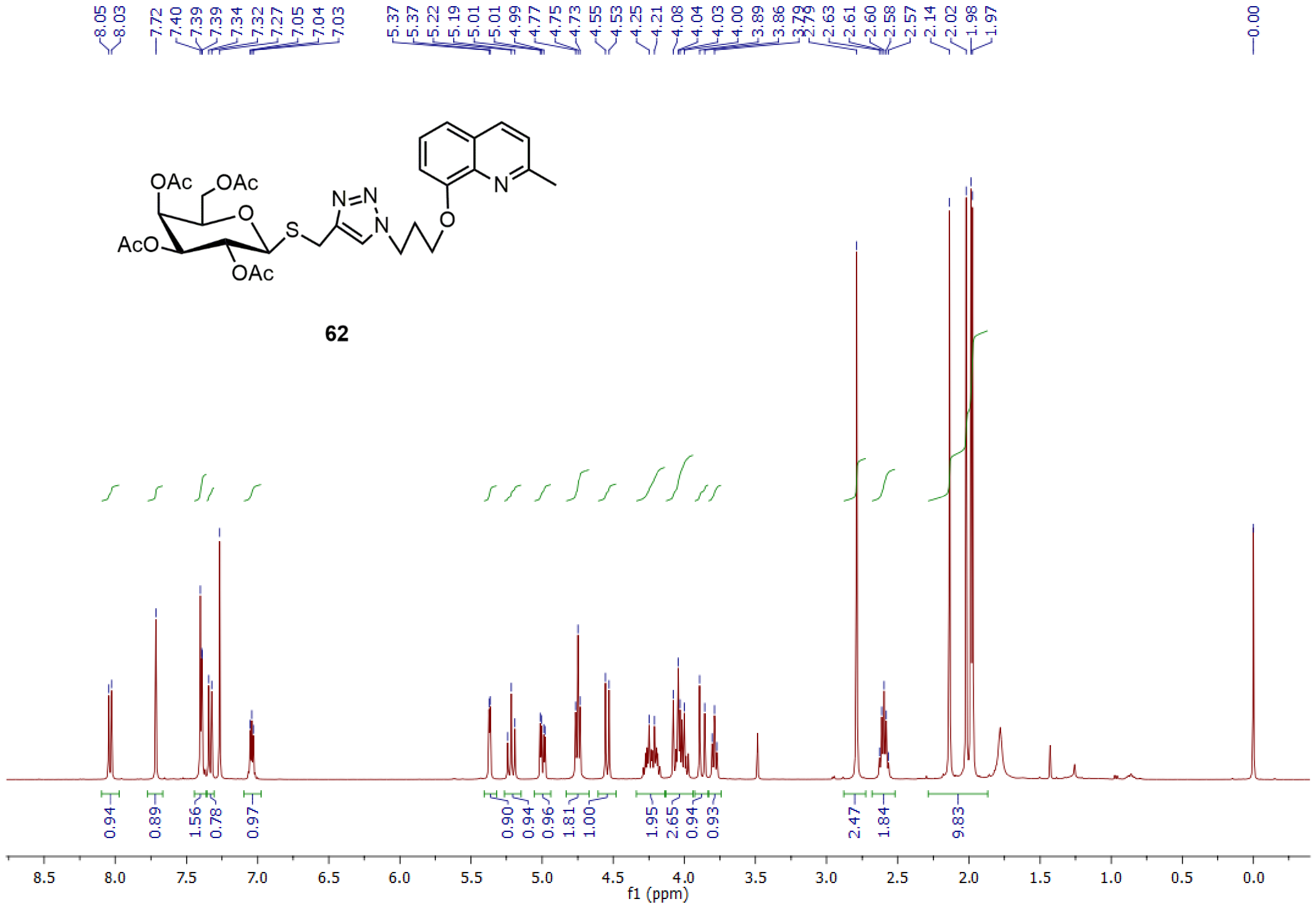

Fig. S108: ^{1}H NMR spectrum of compound **62** (400 MHz/$CDCl_3$/TMS; δ (ppm)).

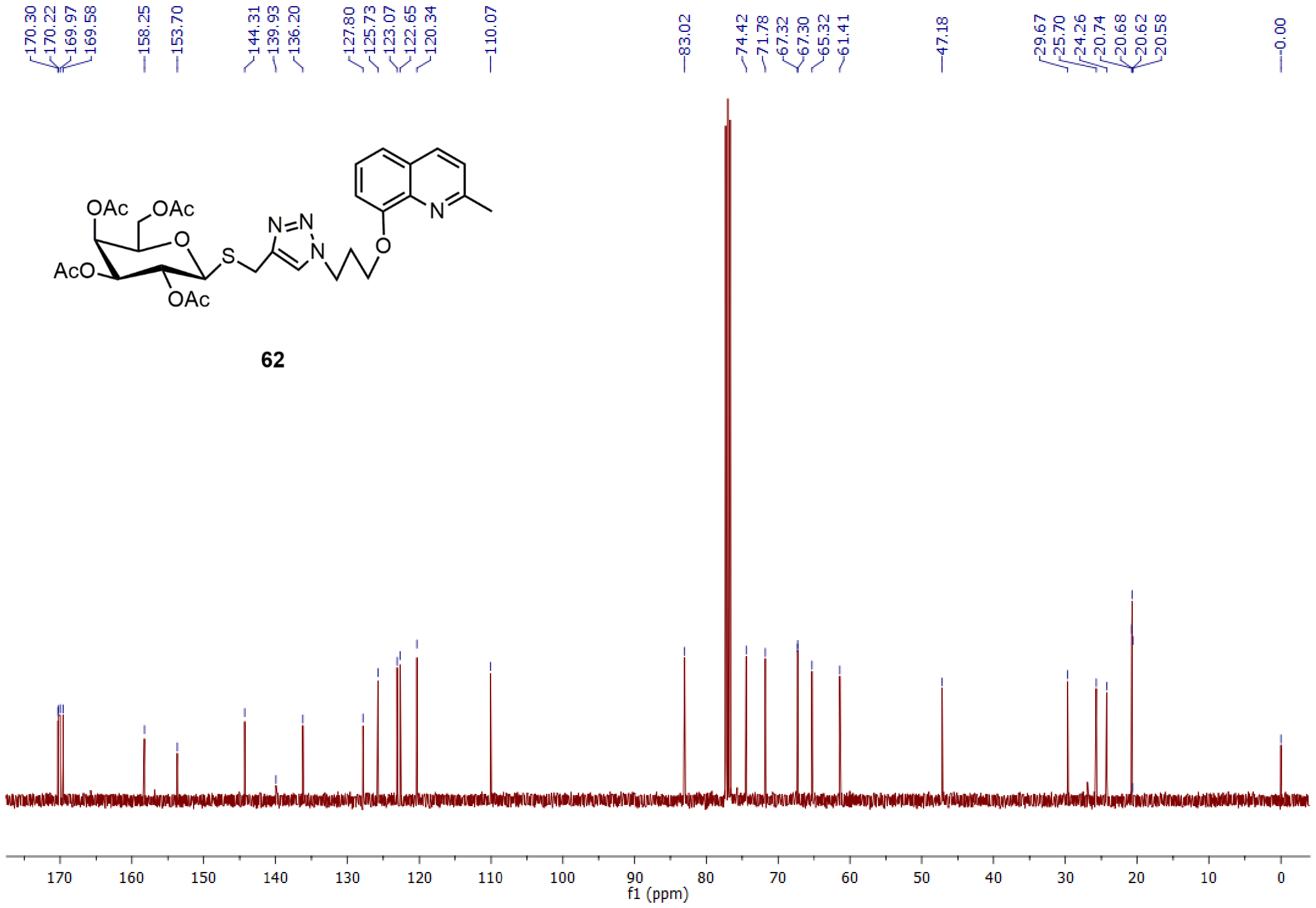

Fig. S109: ^{13}C NMR spectrum of compound **62** (100 MHz/$CDCl_3$/TMS; δ (ppm)).

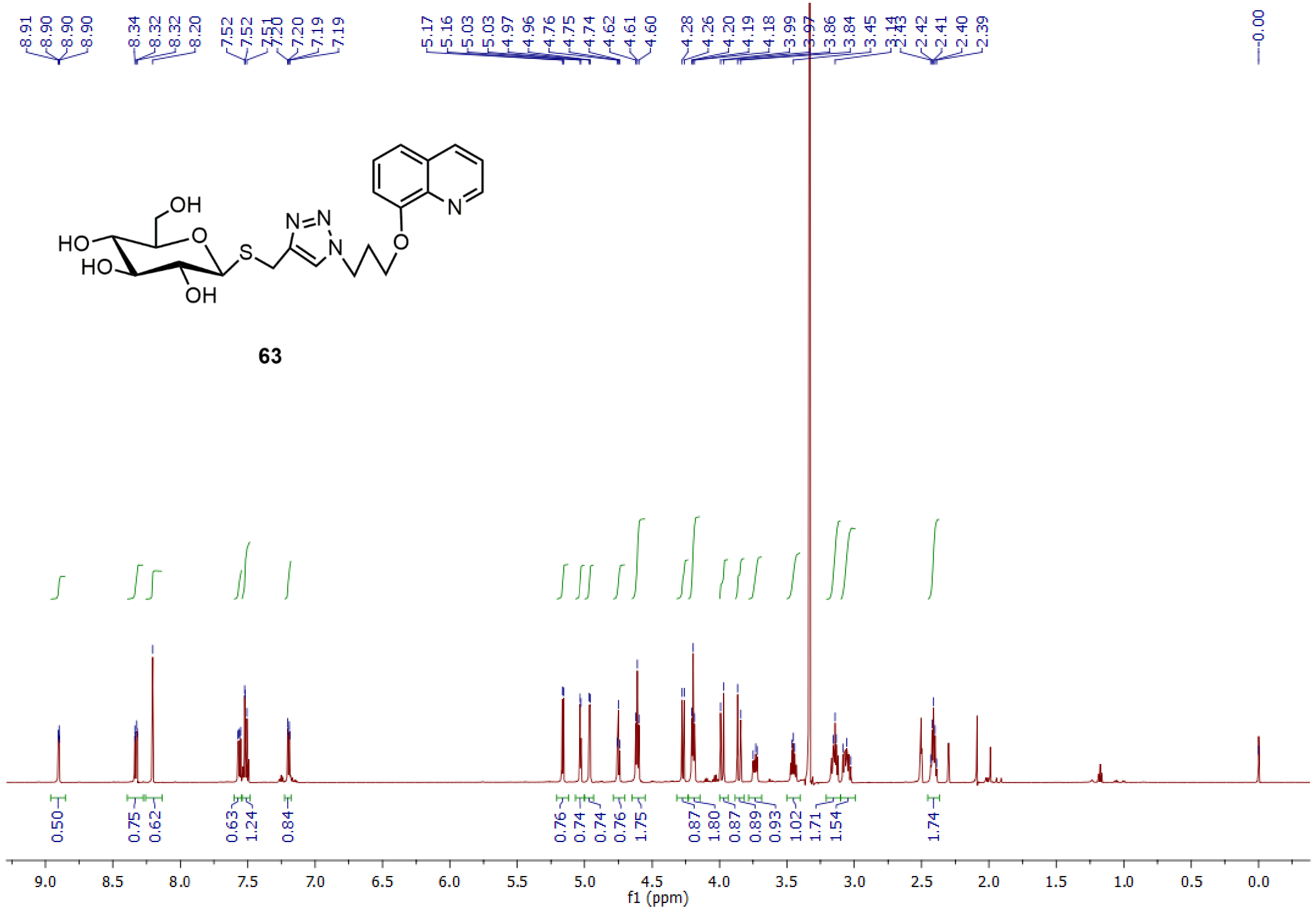

Fig. S110: ^{1}H NMR spectrum of compound **63** (400 MHz/DMSO/TMS; δ (ppm)).

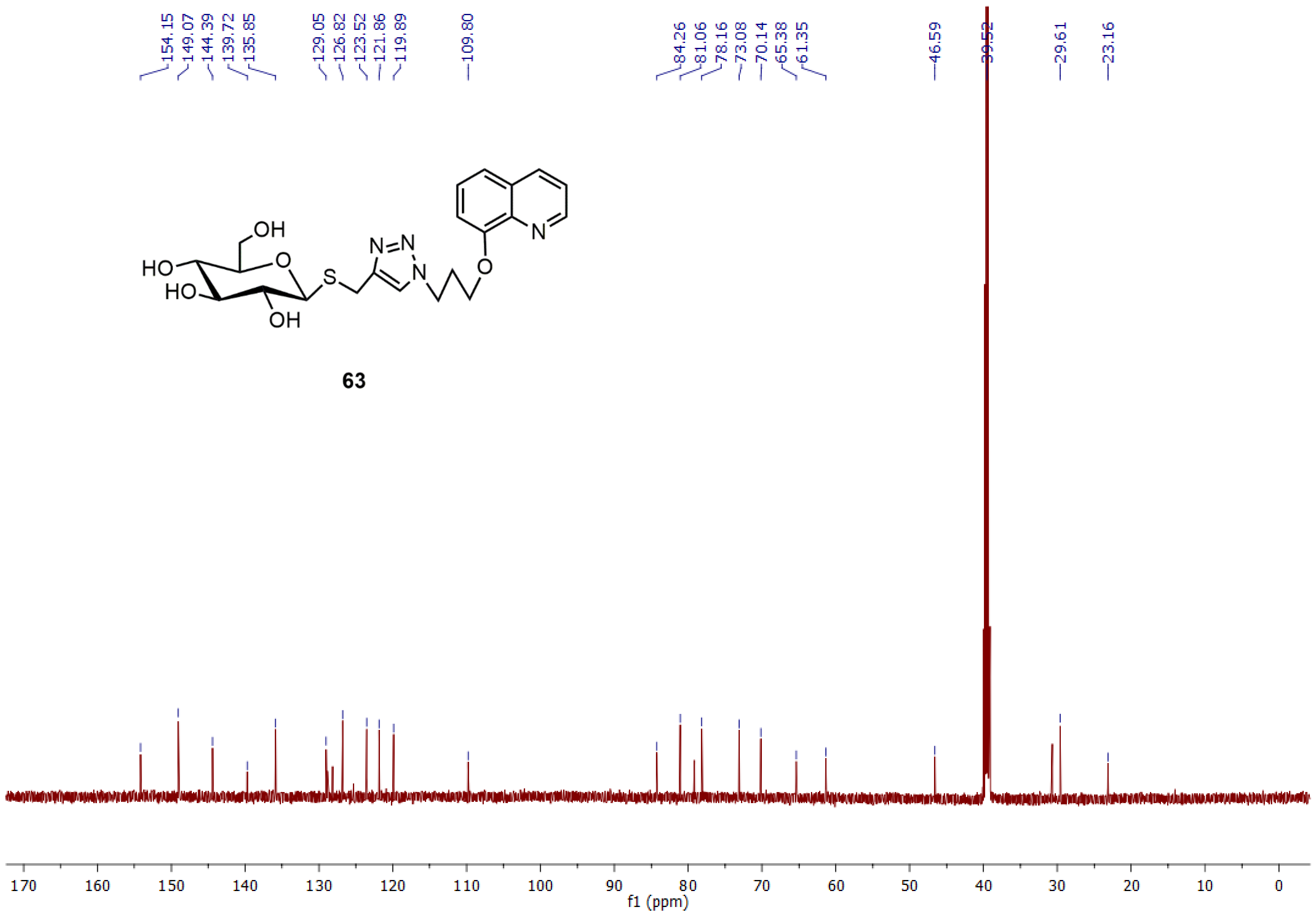

Fig. S111: ^{13}C NMR spectrum of compound **63** (100 MHz/DMSO/TMS; δ (ppm)).

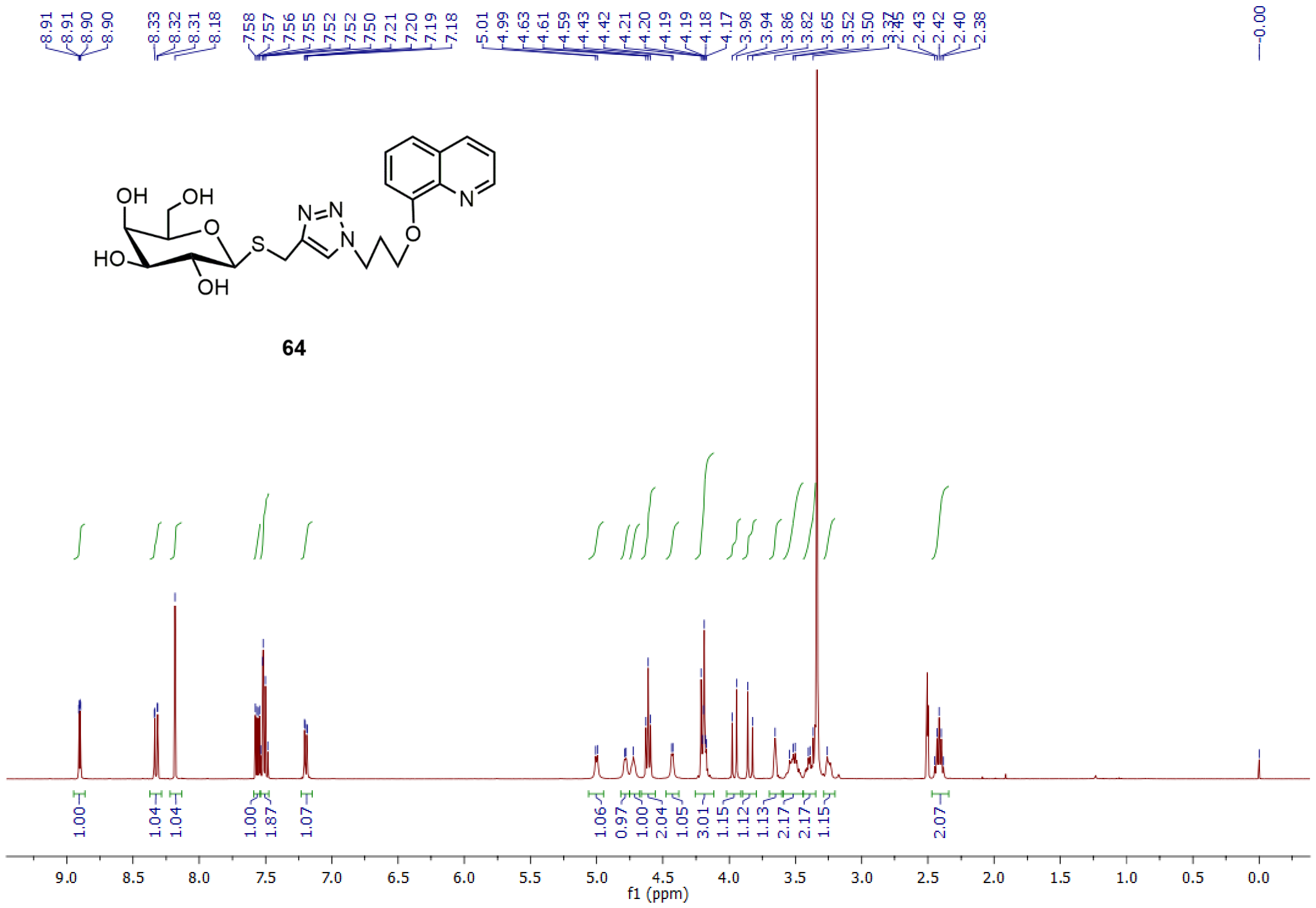

64

Fig. S112: ¹H NMR spectrum of compound **64** (400 MHz/DMSO/TMS; δ (ppm)).

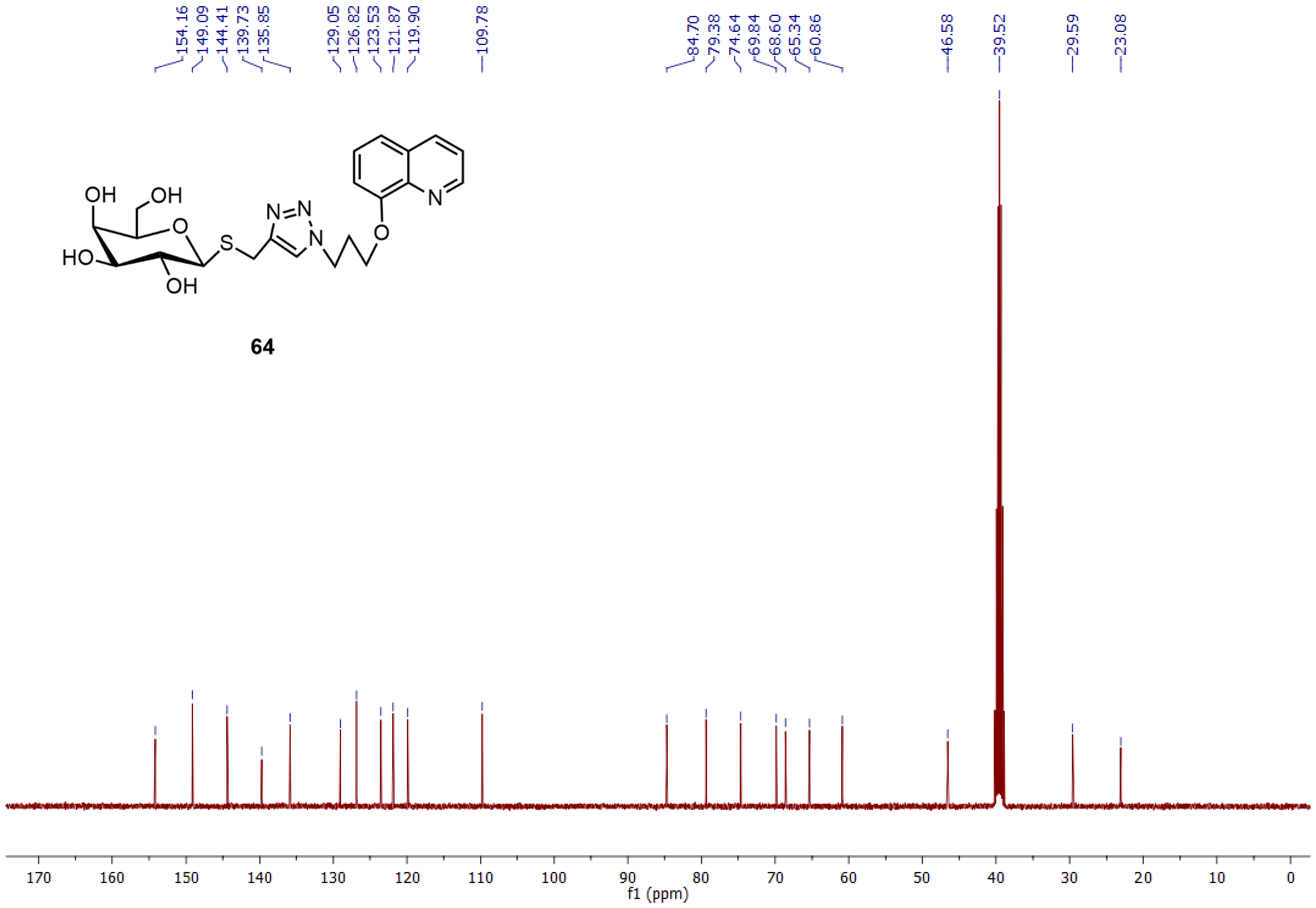

Fig. S113: ^{13}C NMR spectrum of compound **64** (100 MHz/DMSO/TMS; δ (ppm)).

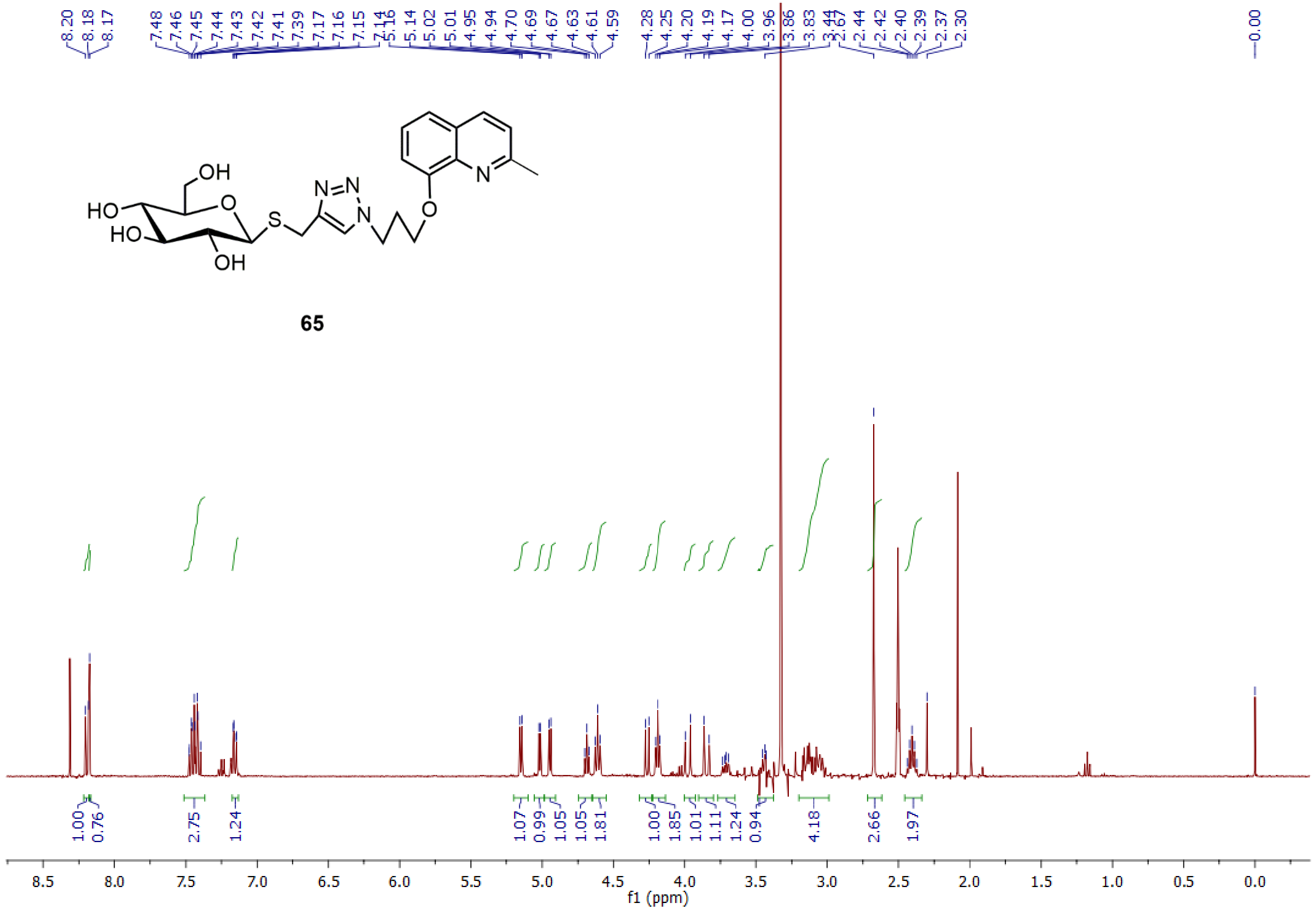

Fig. S114: ¹H NMR spectrum of compound **65** (400 MHz/DMSO/TMS; δ (ppm)).

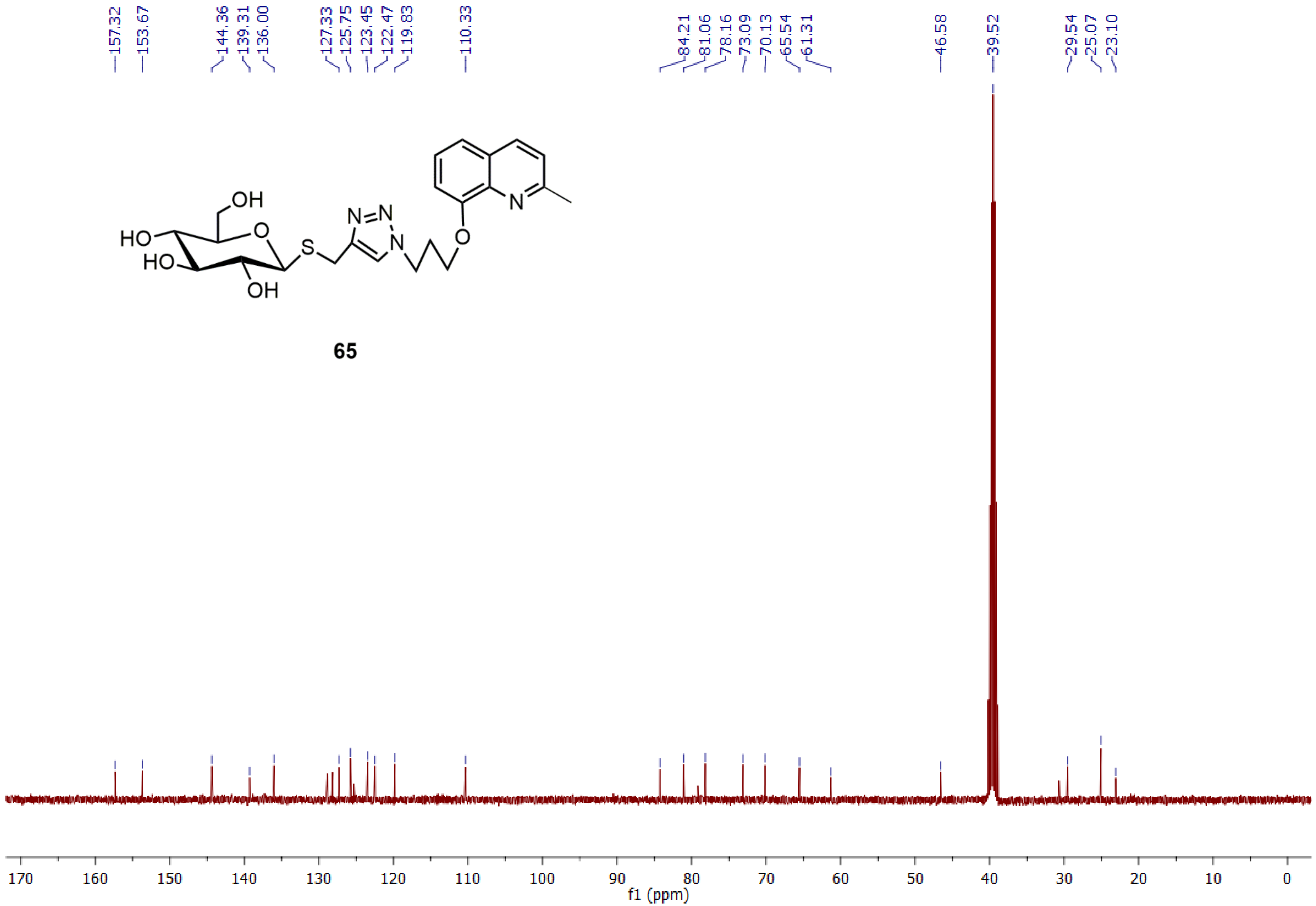

Fig. S115: ^{13}C NMR spectrum of compound **65** (100 MHz/DMSO/TMS; δ (ppm)).

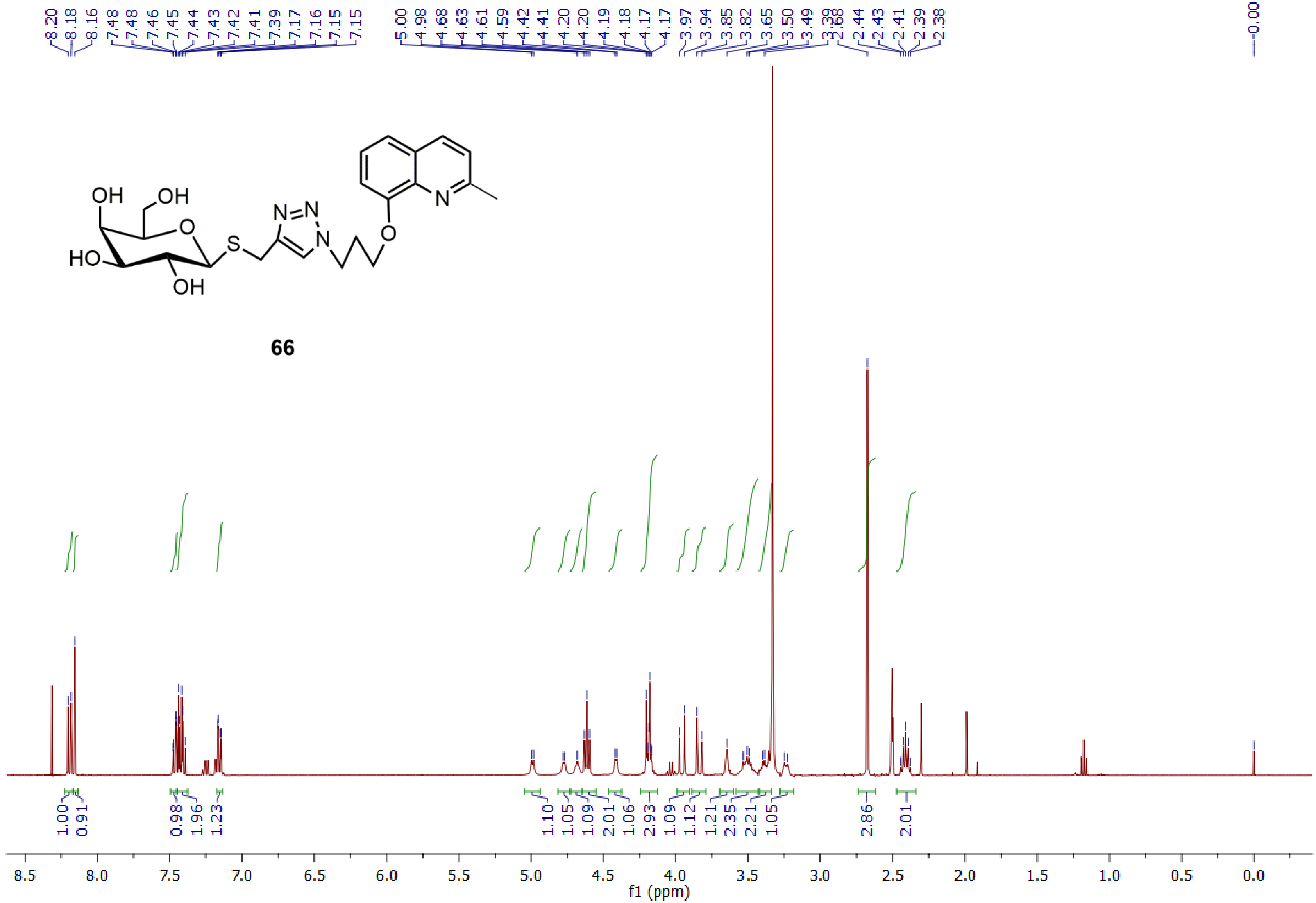

Fig. S116: ^{1}H NMR spectrum of compound **66** (400 MHz/DMSO/TMS; δ (ppm)).

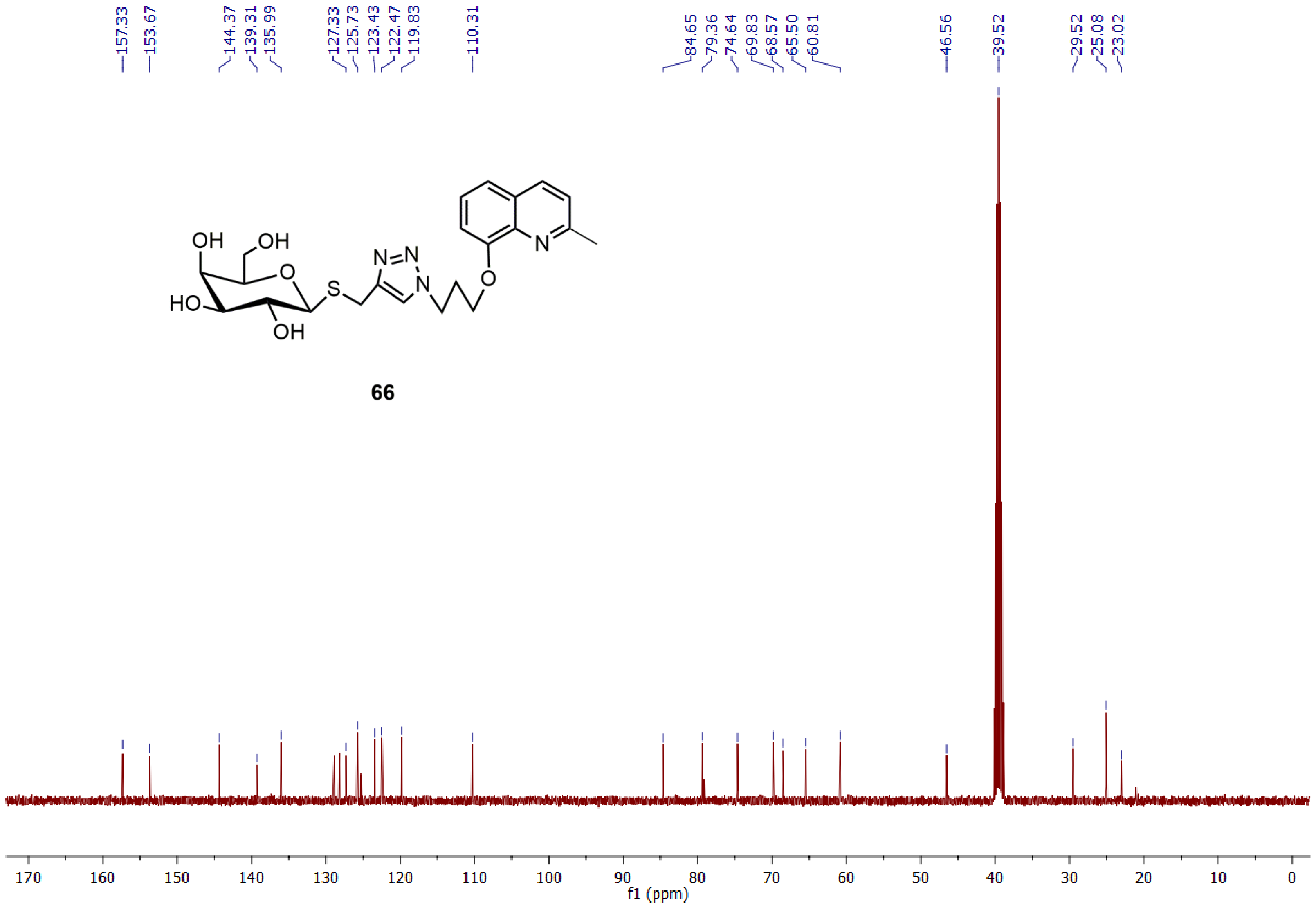

Fig. S117: ^{13}C NMR spectrum of compound **66** (100 MHz/DMSO/TMS; δ (ppm)).

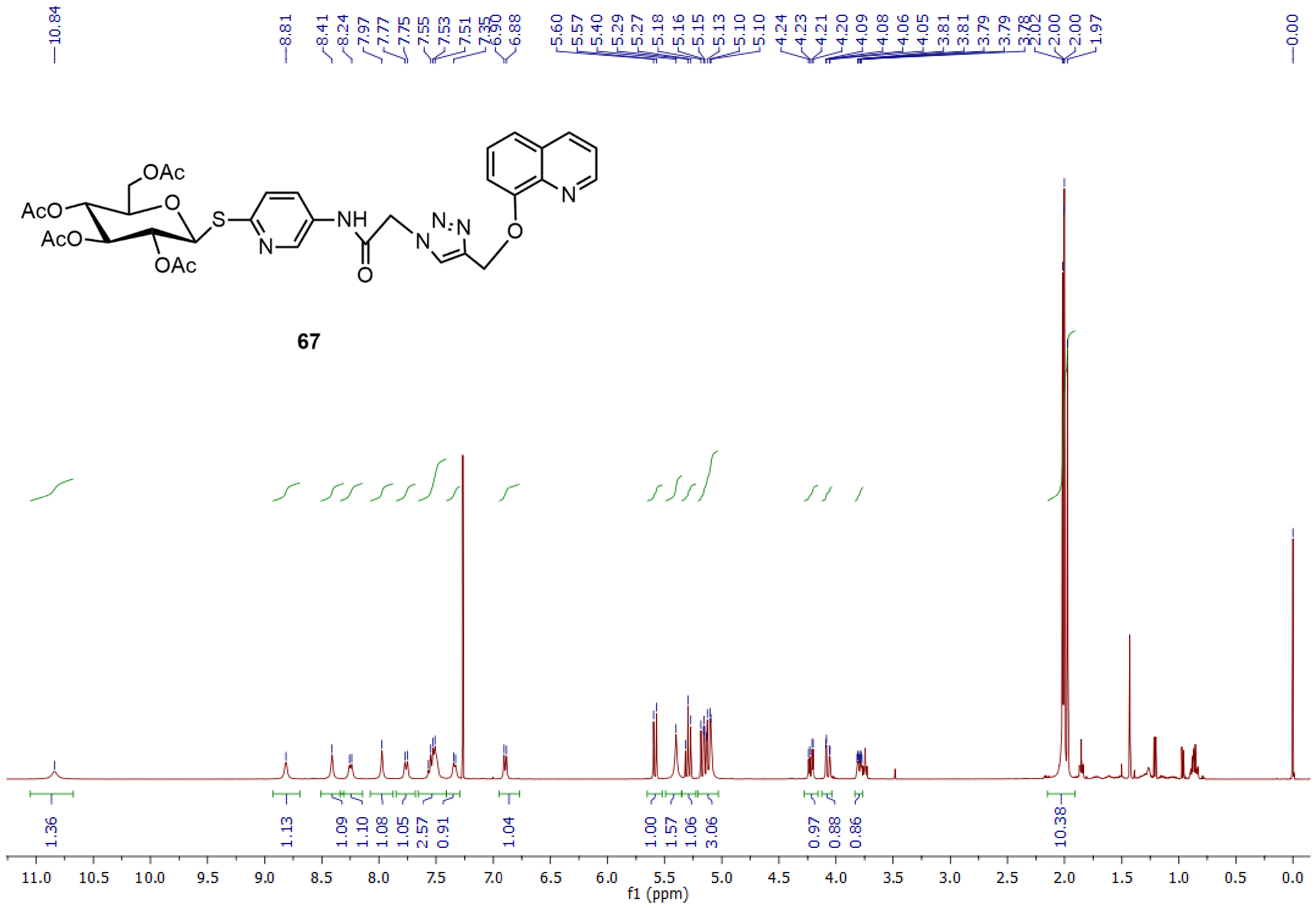

Fig. S118: ¹H NMR spectrum of compound **67** (400 MHz/CDCl₃/TMS; δ (ppm)).

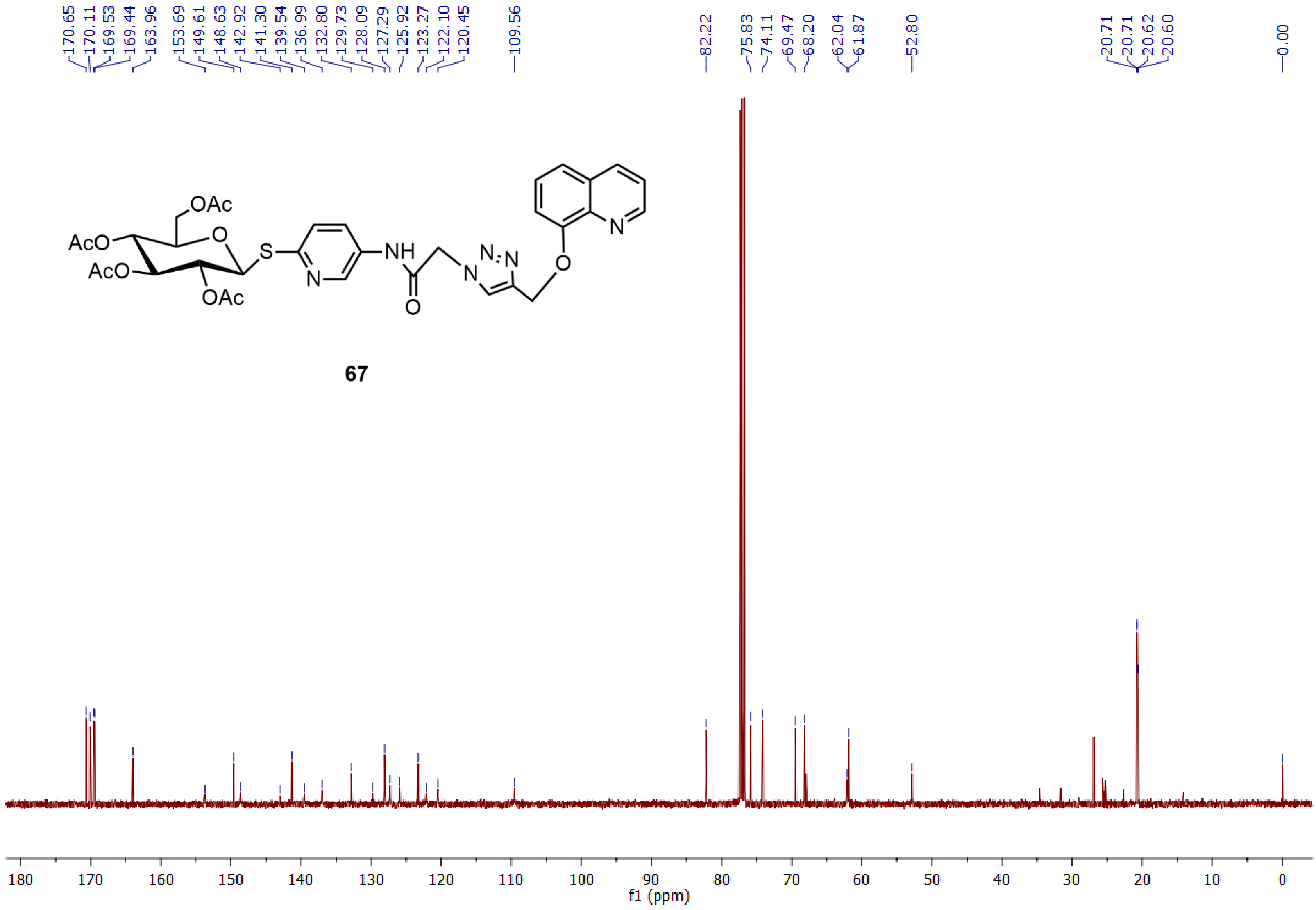

Fig. S119: ^{13}C NMR spectrum of compound **67** (100 MHz/$CDCl_3$/TMS; δ (ppm)).

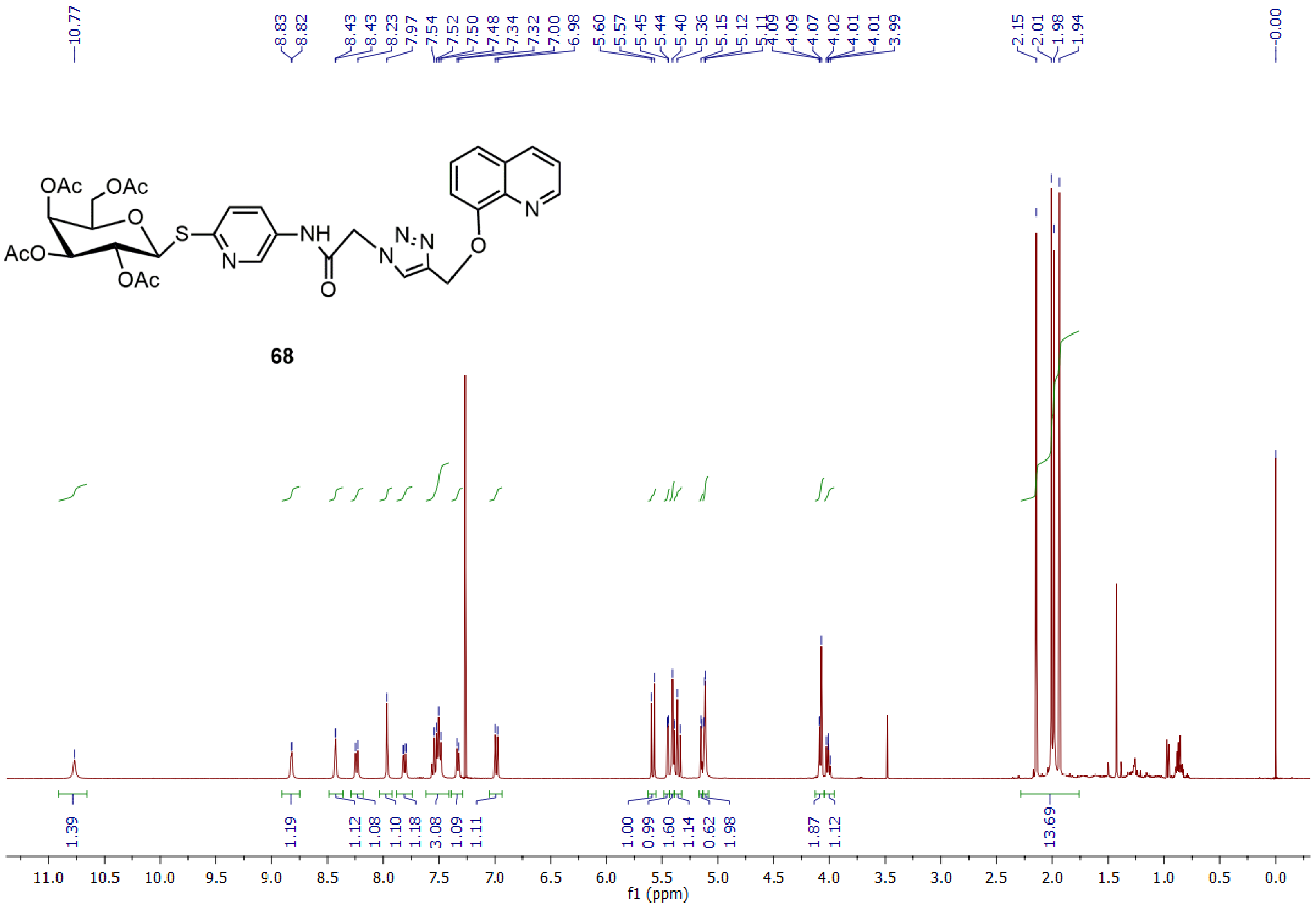

Fig. S120: ^{1}H NMR spectrum of compound **68** (400 MHz/$CDCl_3$/TMS; δ (ppm)).

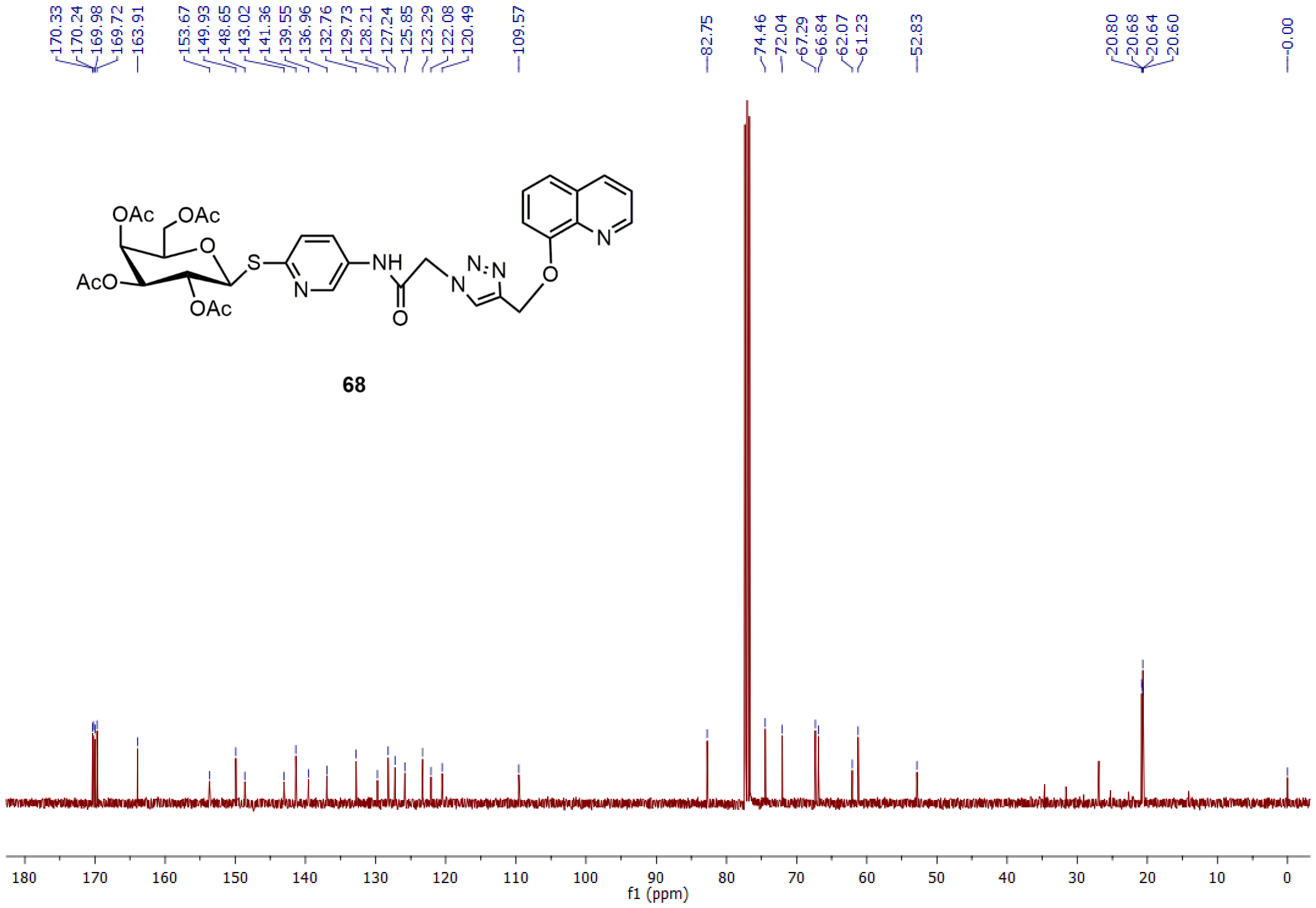

Fig. S121: ^{13}C NMR spectrum of compound **68** (100 MHz/$CDCl_3$/TMS; δ (ppm)).

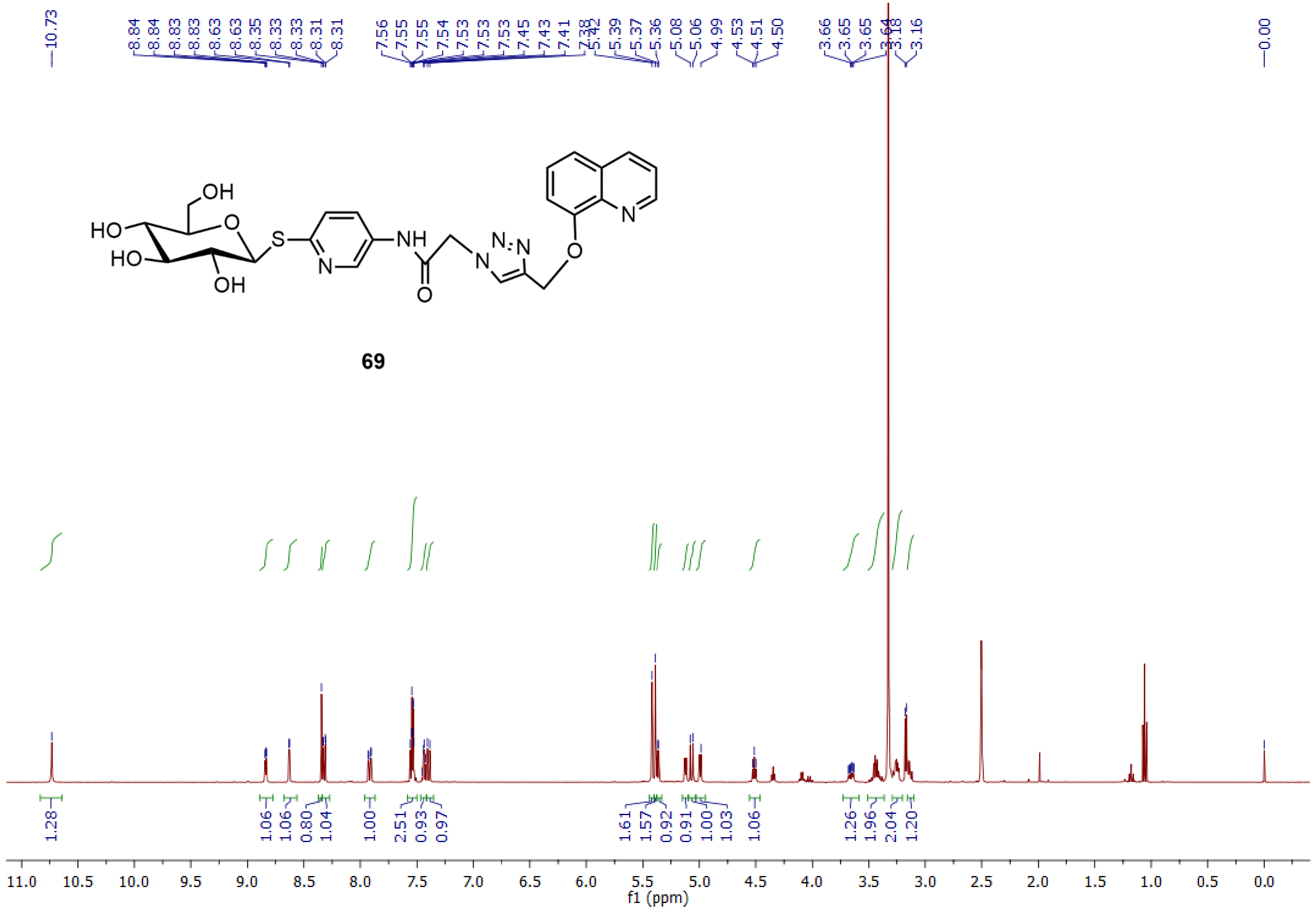

Fig. S122: ^{1}H NMR spectrum of compound **69** (400 MHz/DMSO/TMS; δ (ppm)).

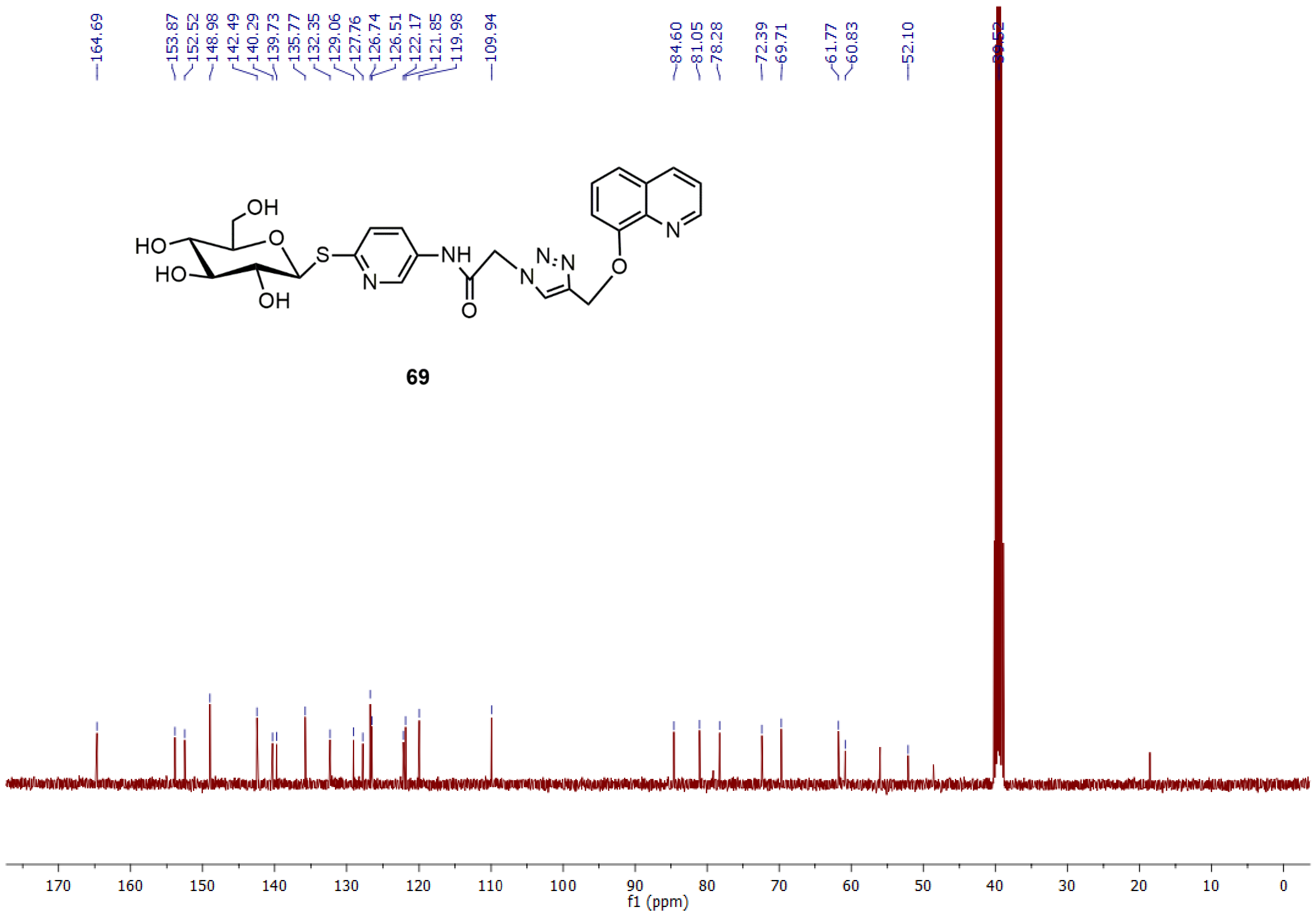

Fig. S123: ^{13}C NMR spectrum of compound **69** (100 MHz/DMSO/TMS; δ (ppm)).

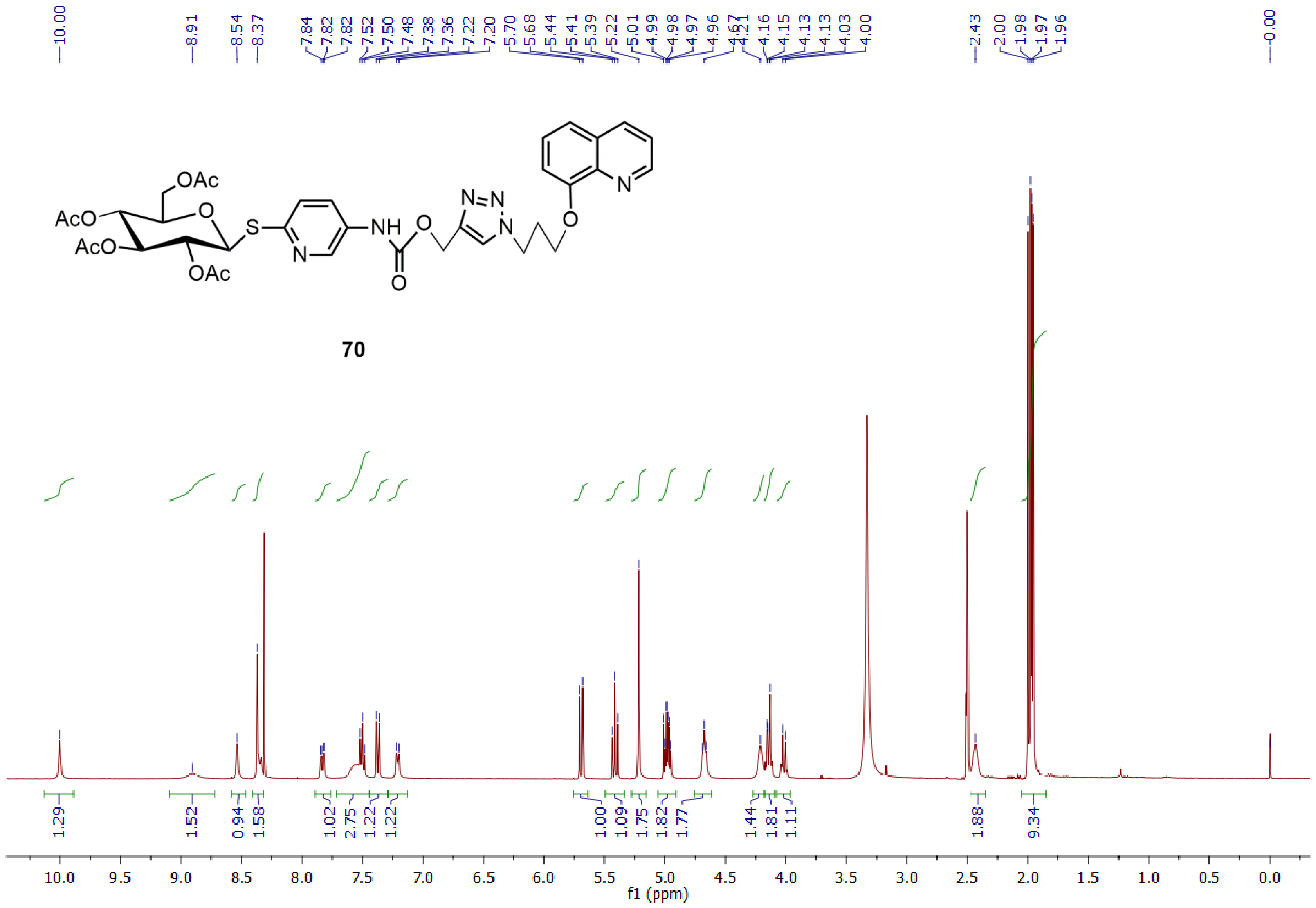

Fig. S124: ^{1}H NMR spectrum of compound **70** (400 MHz/DMSO/TMS; δ (ppm)).

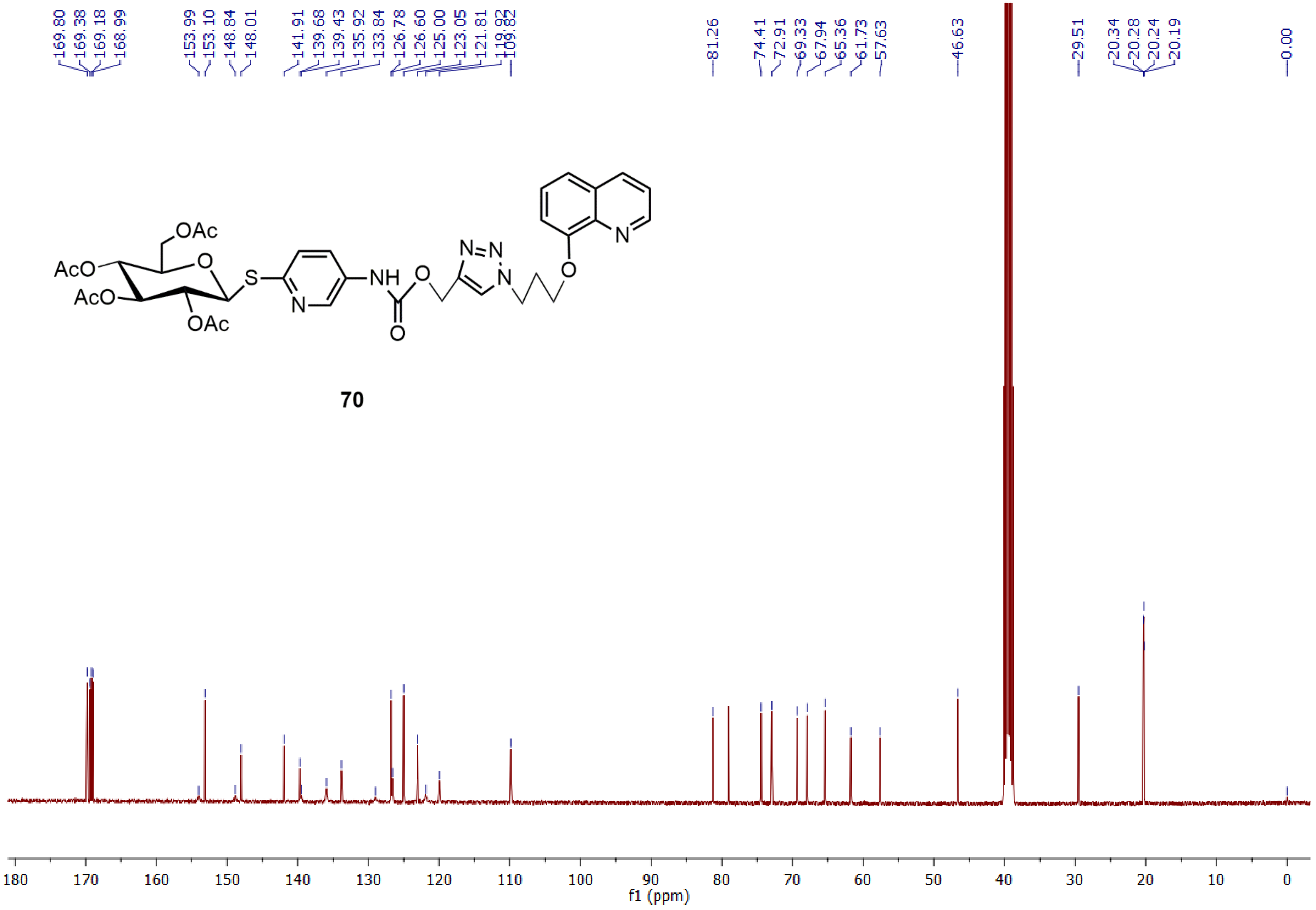

Fig. S125: ^{13}C NMR spectrum of compound **70** (100 MHz/DMSO/TMS; δ (ppm)).

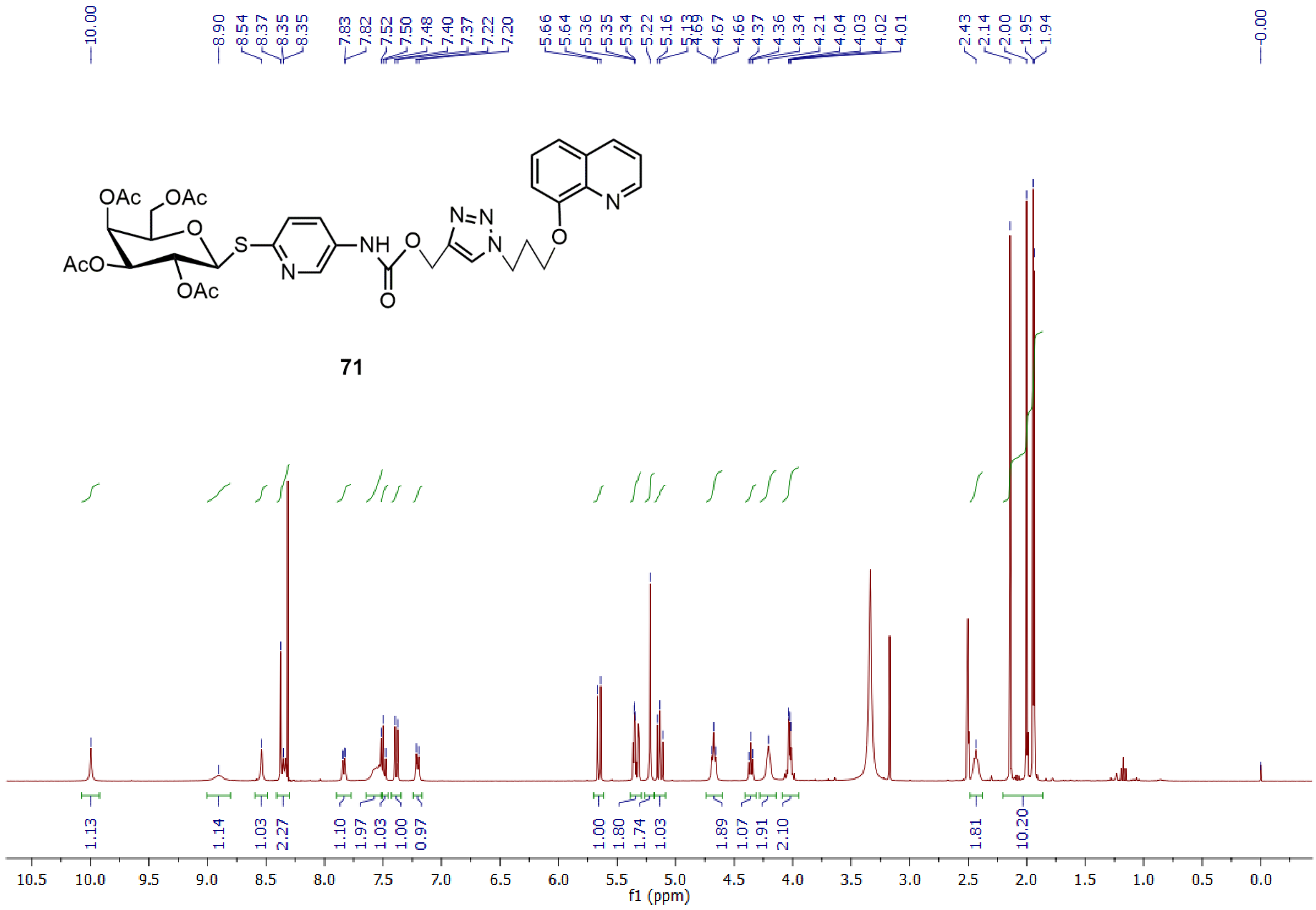

Fig. S126: ^{1}H NMR spectrum of compound **71** (400 MHz/DMSO/TMS; δ (ppm)).

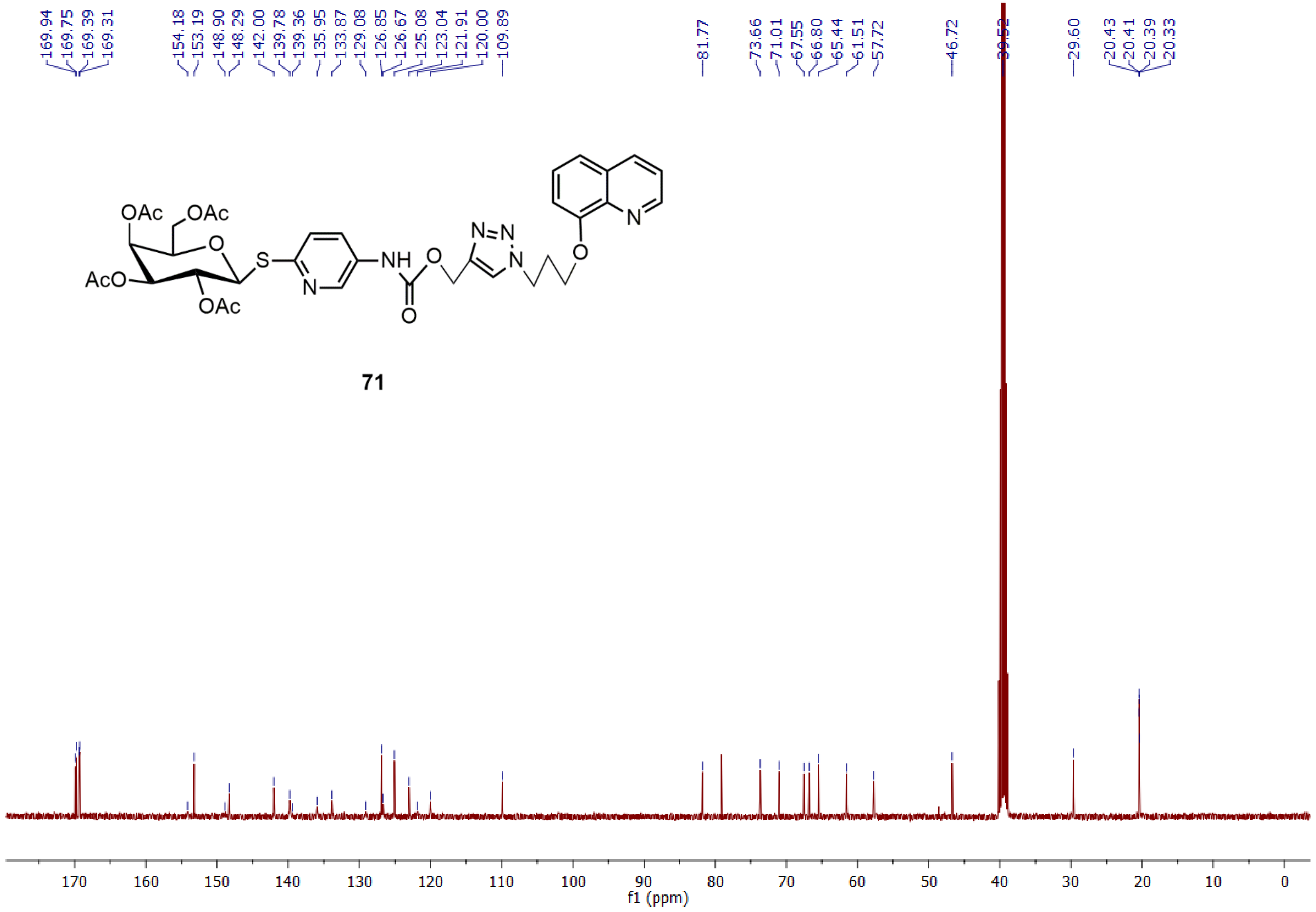

Fig. S127: ^{13}C NMR spectrum of compound **71** (100 MHz/DMSO/TMS; δ (ppm)).

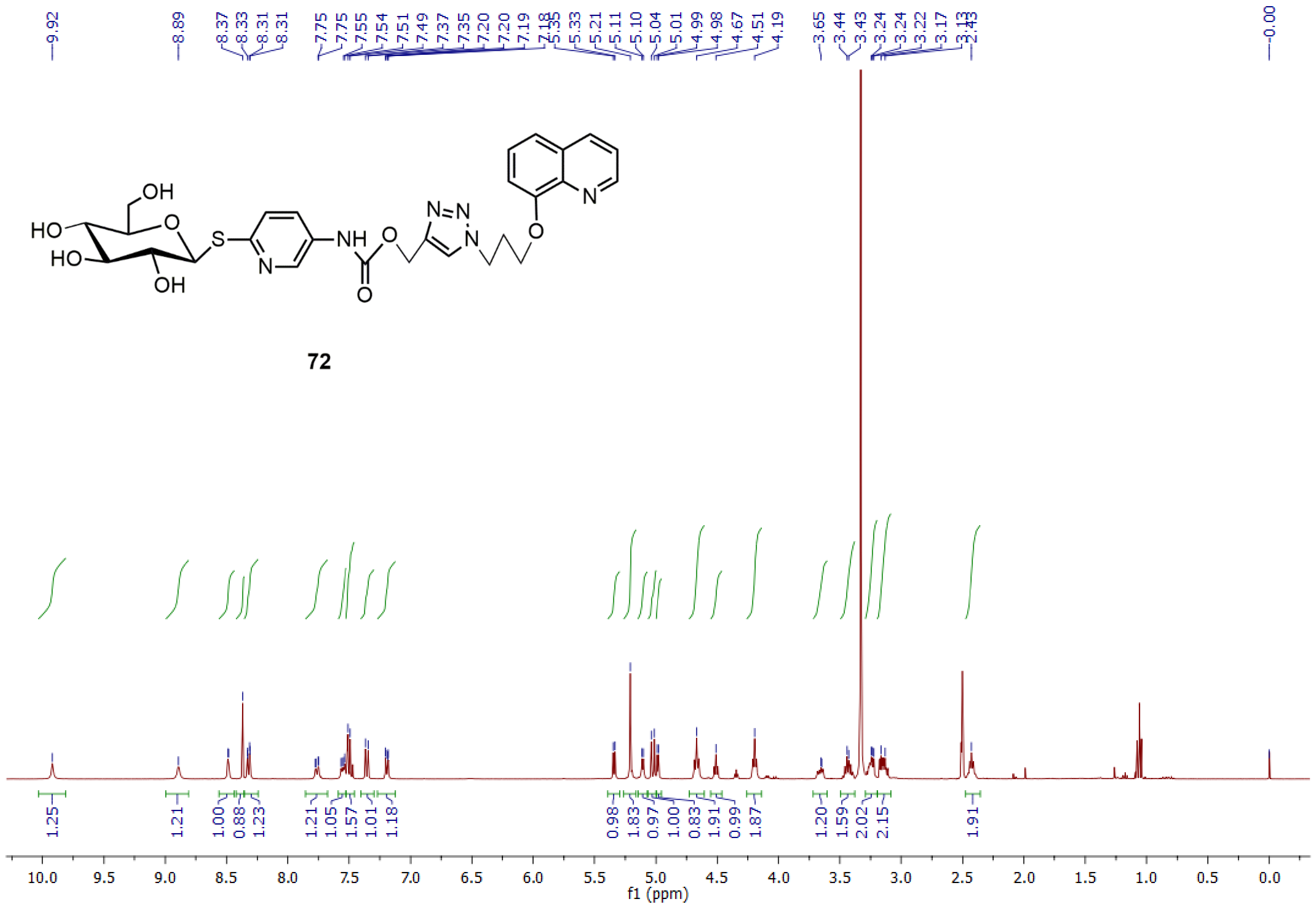

Fig. S128: ^{1}H NMR spectrum of compound **72** (400 MHz/DMSO/TMS; δ (ppm)).

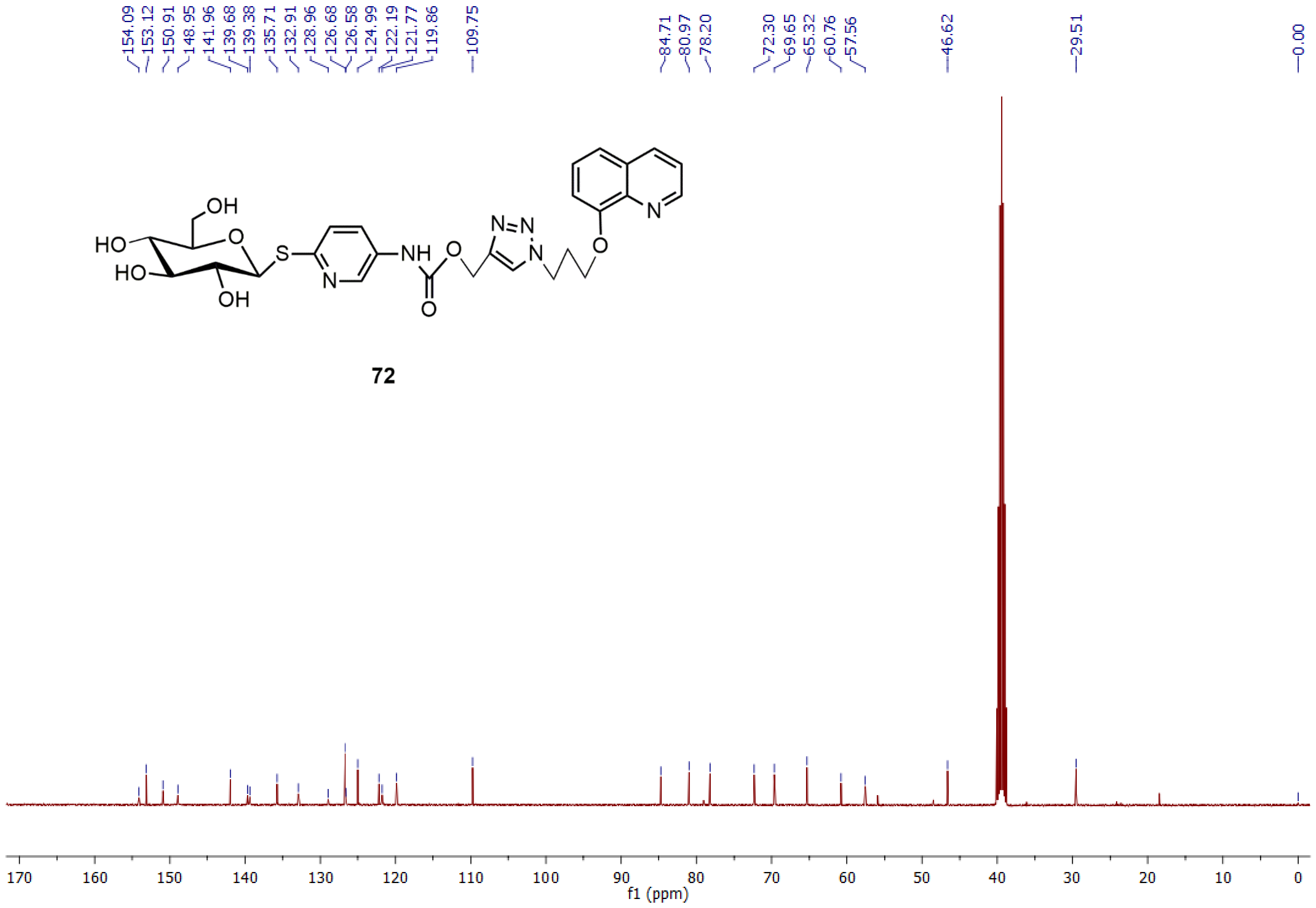

Fig. S129: ^{13}C NMR spectrum of compound **72** (100 MHz/DMSO/TMS; δ (ppm)).

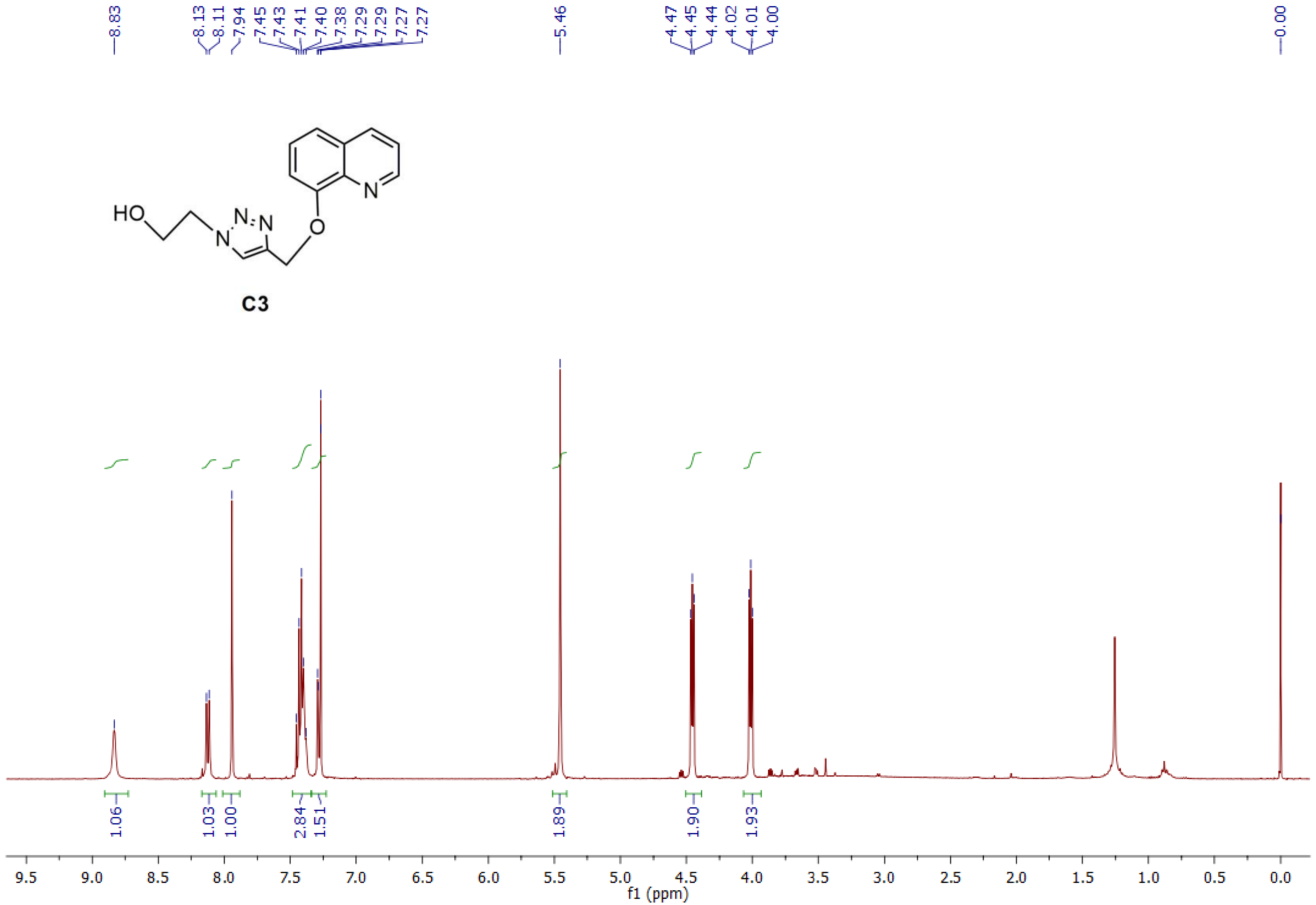

Fig. S130: ^{1}H NMR spectrum of compound **C3** (400 MHz/$CDCl_3$/TMS; δ (ppm)).

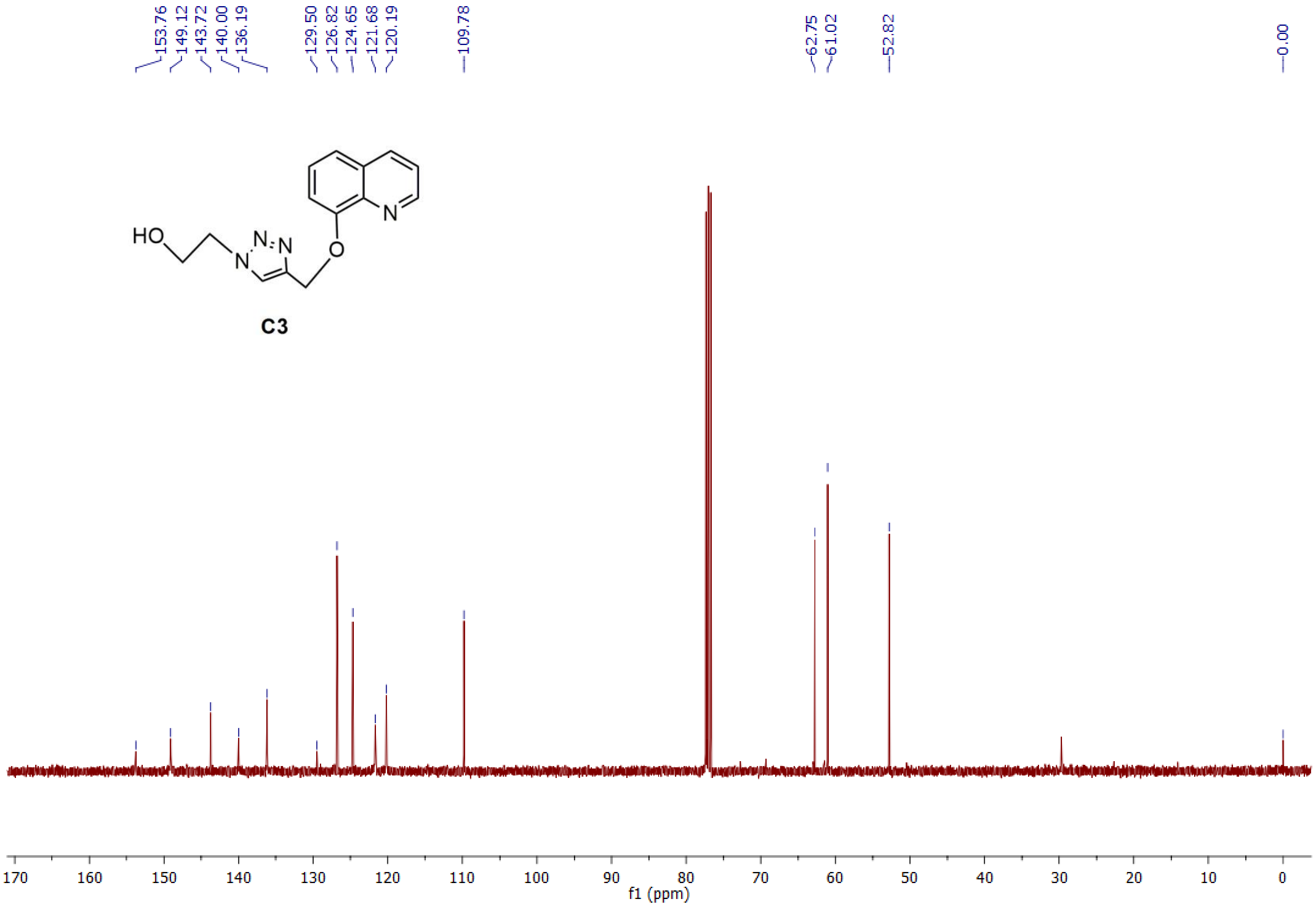

Fig. S131: ^{13}C NMR spectrum of compound **C3** (100 MHz/ $CDCl_3$/TMS; δ (ppm)).

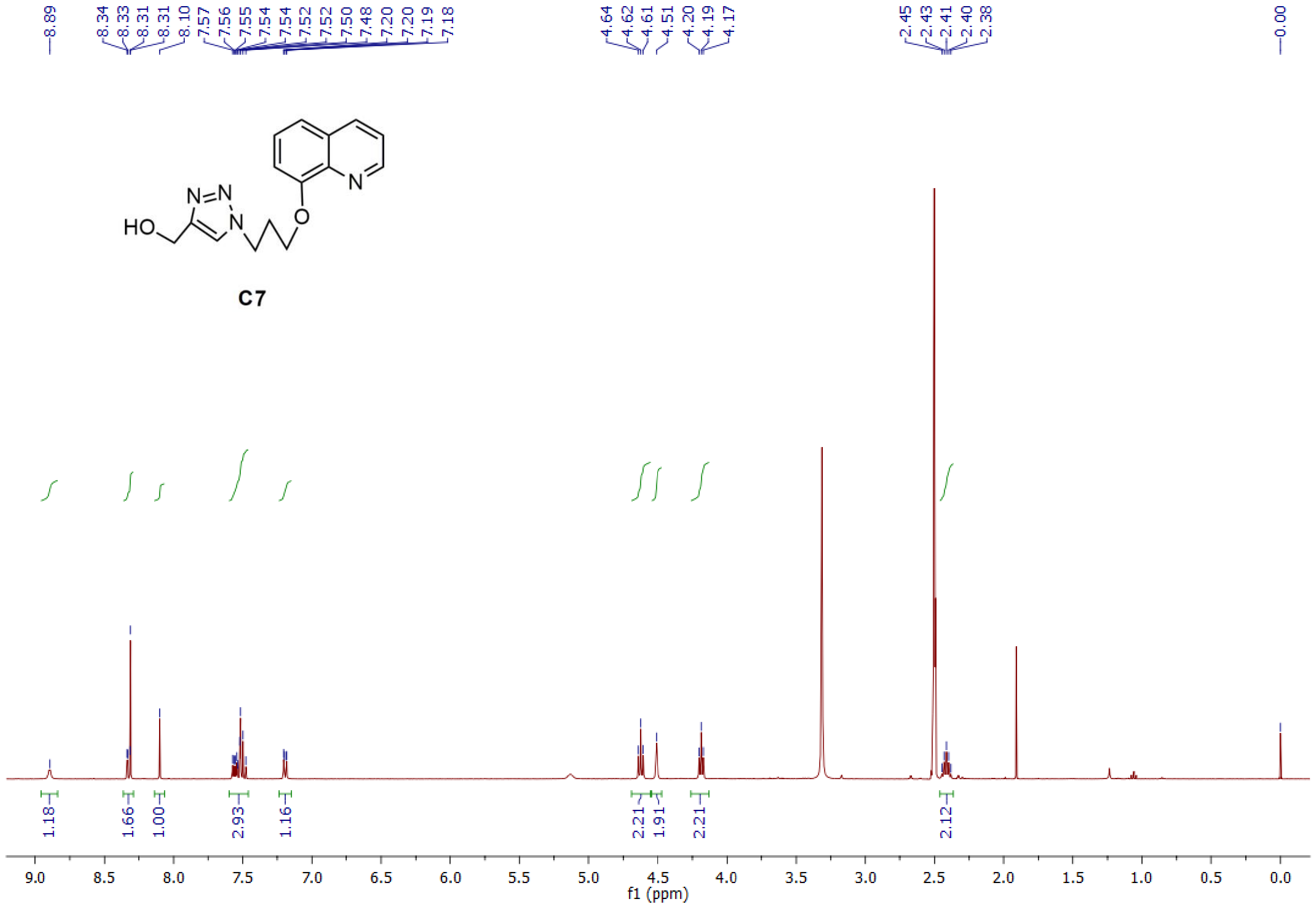

Fig. S132: ^{1}H NMR spectrum of compound **C7** (400 MHz/DMSO/TMS; δ (ppm)).

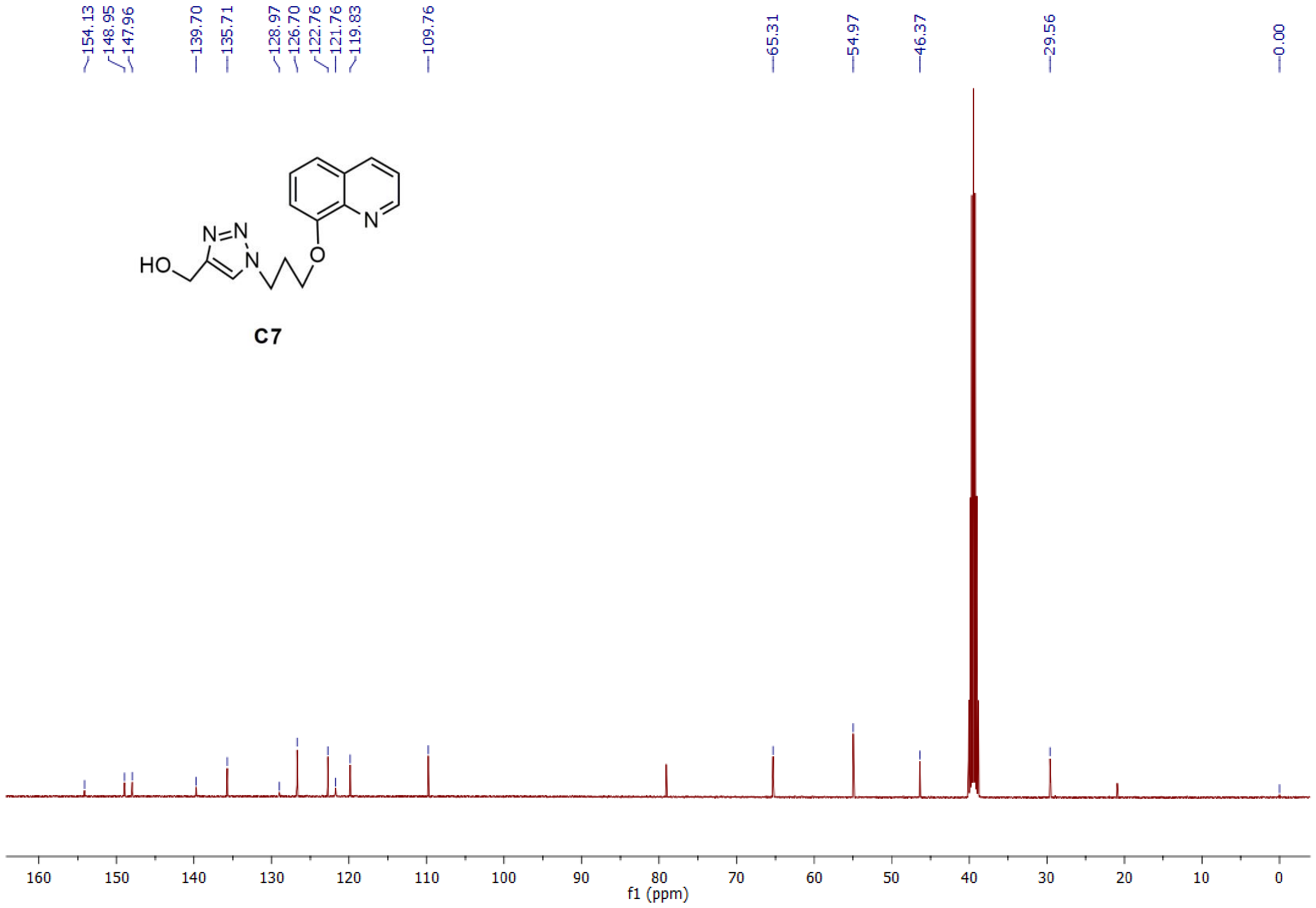

Fig. S133: ^{13}C NMR spectrum of compound **C7** (100 MHz/DMSO/TMS; δ (ppm)).
